# Understanding the Carbyne Formation from C_2_H_2_ Complexes

**DOI:** 10.1021/jacs.4c07724

**Published:** 2024-11-15

**Authors:** Miljan Z. Ćorović, Madeleine A. Ehweiner, Peter E. Hartmann, Felix Sbüll, Ferdinand Belaj, A. Daniel Boese, Jesse Lepluart, Martin L. Kirk, Nadia C. Mösch-Zanetti

**Affiliations:** † Institute of Chemistry, Inorganic Chemistry, University of Graz, Schubertstrasse 1, 8010 Graz, Austria; ‡ Institute of Chemistry, Physical and Theoretical Chemistry, University of Graz, Heinrichstrasse 28, 8010 Graz, Austria; § Department of Chemistry and Chemical Biology, The University of New Mexico, MSC03 2060, 1 University of New Mexico, Albuquerque, New Mexico 87131-0001, United States

## Abstract

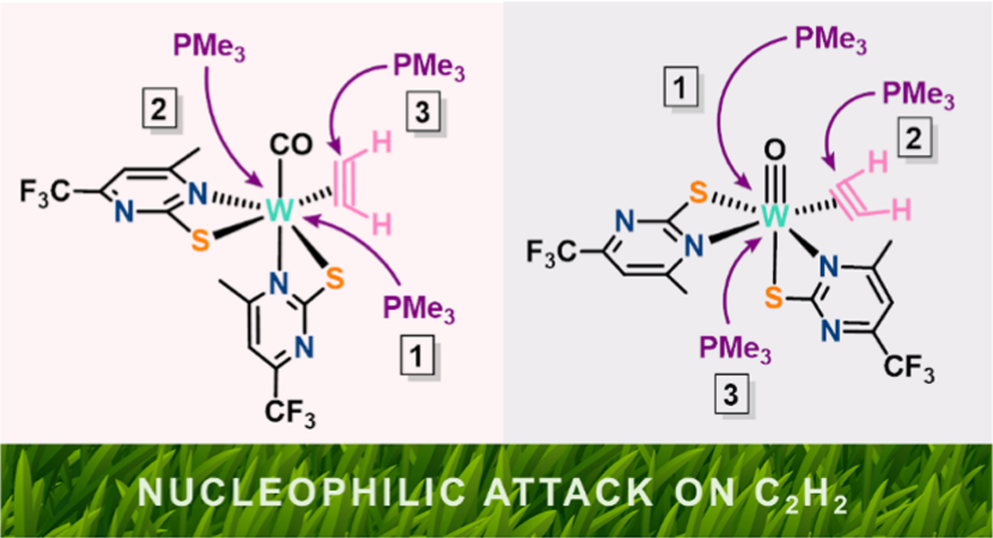

Nature chooses a high-valent tungsten center at the active site of the enzyme acetylene hydratase to facilitate acetylene hydration to acetaldehyde. However, the reactions of tungsten-coordinated acetylene are still not well understood, which prevents the development of sustainable bioinspired alkyne hydration catalysts. Here we report the reactivity of two bioinspired tungsten complexes with the acetylene ligand acting as a four-: [W(CO)(C_2_H_2_)(PymS)_2_] (**1**) and a two-electron donor: [WO(C_2_H_2_)(PymS)_2_] (**3**), with PMe_3_ as a nucleophile to simulate the enzyme’s reactivity (PymS = 4-(trifluoromethyl)-6-methylpyrimidine-2-thiolate). In dichloromethane, compound **1** was found to react to the cationic carbyne [W≡CCH_2_PMe_3_(CO)(PMe_3_)_2_(PymS)]Cl (**2-Cl**) while **3** reacts to the vinyl compound [WO(CH=CHPMe_3_)(PMe_3_)_3_(PymS)]Cl (**4-Cl**). The formation of the latter follows the common rules applied to η^2^-alkyne complexes, whereas the carbyne formation was not expected due to the challenging 1,2-H shift. To understand these differences in behavior between seemingly similar acetylene complexes, stepwise addition of the nucleophile in various solvents was investigated by synthetic, spectroscopic, and computational approaches. In this manuscript, we describe that only a four-electron donor acetylene complex can react to the carbyne over the η^1^-vinyl intermediate and that 1,2-H shift can be assisted by an H-transfer reagent (in this case, the decoordinated PymS ligand). Furthermore, to favor the attack of PMe_3_ at W coordinated acetylene, the metal center needs to be electron-poor and crowded enough to prevent nucleophile coordination. Finally, the intricate role of the anionic PymS ligand in the vicinity of the first coordination sphere models the potential involvement of amino acid residues during acetylene transformations in AH.

## Introduction

**Scheme 1:**
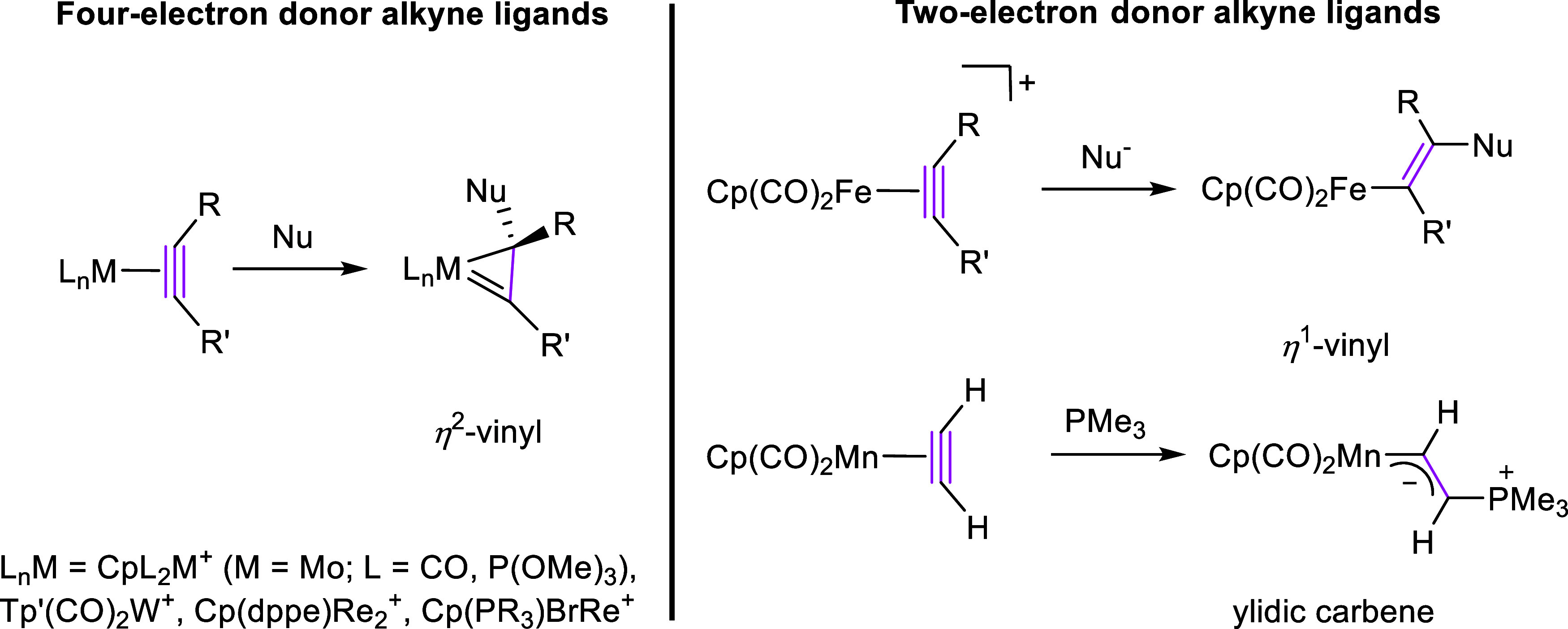
Nucleophile Addition to Alkyne Ligands^25^^,^ Reprinted from ref (25), Copyright 2000, with permission from Elsevier.

Versatile chemistry catalyzed by group VI metalloenzymes inspires the study of Mo and W biomimetic complexes.^1−7^ Biomimetic complexes assist in understanding these enzyme-catalyzed reactions and possess potential as powerful tools for achieving sustainability in small molecule activation and catalysis.^8^ Developing environmentally friendly catalysts for alkyne hydration is essential, as current methods rely on costly and toxic transition metals such as mercury.^9−11^ Furthermore, acetylene, derived from abundant coal reserves in regions such as Western China, provides a readily available and energy-rich raw material.^12^ However, direct hydration of acetylene could be reconsidered for industrial production in coal-rich areas, provided a more sustainable catalyst is available. Therefore, research efforts geared toward sustainable acetylene hydration are influenced by an increased understanding of the mechanism of the tungstoenzyme acetylene hydratase (AH).^13^ The tungsten(IV) center in the enzyme’s active site is coordinated by two pyranopterin dithiolene (PDT) moieties, a cysteine residue, and a water molecule/hydroxide. An aspartate residue is found in the vicinity of the active site.^14−16^ There are two conflicting views on the AH mechanism, suggesting either an attack of water at the coordinated acetylene,^17−19^ or an attack of coordinated hydroxide at the free acetylene.^20,21^ Well-established tungsten (and molybdenum) alkyne chemistry^22−24^ as well as AH’s high chemoselectivity^18^ for C_2_H_2_ support the idea of an organometallic intermediate. Hence, it is advantageous to continue the exploration of the fundamental principles governing acetylene activation for nucleophilic attack. Templeton and Frohnapfel have classified the reactions of η^2^-alkyne complexes with nucleophiles into two categories depending on the type of alkyne donors (Scheme 1).^25^

To maintain the initial total electron count, four-electron donor alkynes undergo nucleophilic attack to form η^2^-vinyl complexes. Conversely, metal fragments stabilizing two-electron donor alkynes yield η^1^-vinyl complexes. The latter could also be stabilized as ylidic carbenes in special cases, as shown by Alt and co-workers in the reaction between Mn(I) acetylene complex and phosphine.^26^ Although this classification explains many reactions involving η^2^-alkynes, some of the interesting transformations remain elusive, especially for the behavior of the unsubstituted C_2_H_2_ ligands. Particularly, the formation of the carbyne complex from an acetylene adduct is intriguing because of the kinetically unfavorable 1,2-H shift from C_α_ to C_β_.^27−29^

As mentioned above, a computational study^17^ suggested the involvement of a W–C_2_H_2_ intermediate in the AH catalytic cycle. We developed model acetylene complexes and upon studying their reactivity we serendipitously observed carbyne complex formation.^30^ The tungsten carbyne was obtained in the reaction of the W–C_2_H_2_ complex containing two sulfur-rich 6-methylpyridine-2-thiolates (6-MePyS) with the strong nucleophile PMe_3_. Although not biomimetic, the formation of a carbyne from the C_2_H_2_ adduct is rare and sparked our curiosity. Interestingly, Peters and co-workers reacted the mononuclear tris(phosphino)silyl-iron acetylene complex [(SiP_3_)Fe(C_2_H_2_)]BArF_24_ with TEMPOH and isolated the unusual iron(V)—carbyne as the product of reductive protonation.^29^ Overall, it is still difficult to predict the behavior of the coordinated acetylene due to the lack of thorough analysis of reaction mechanisms. This manuscript presents a comprehensive experimental and computational study on the stepwise addition of a nucleophile to the tungsten(II) and tungsten(IV) acetylene complexes bearing two bidentate 4-(trifluoromethyl)-6-methylpyrimidine-2-thiolate ligands. The S,N bidentate ligand is abbreviated with PymS for simplicity, although this label was previously attributed to the unsubstituted pyrimidine-2-thiolate.^31^ With our findings, we extend Templeton’s classification of the η^2^-alkyne reactivity and provide an explanation for the carbyne complex formation.^25^ Furthermore, we aim to understand the circumstances under which W-coordinated acetylene is attacked and expand the knowledge regarding the AH mechanism.

## Results

### Syntheses of the Complexes

**Scheme 2:**
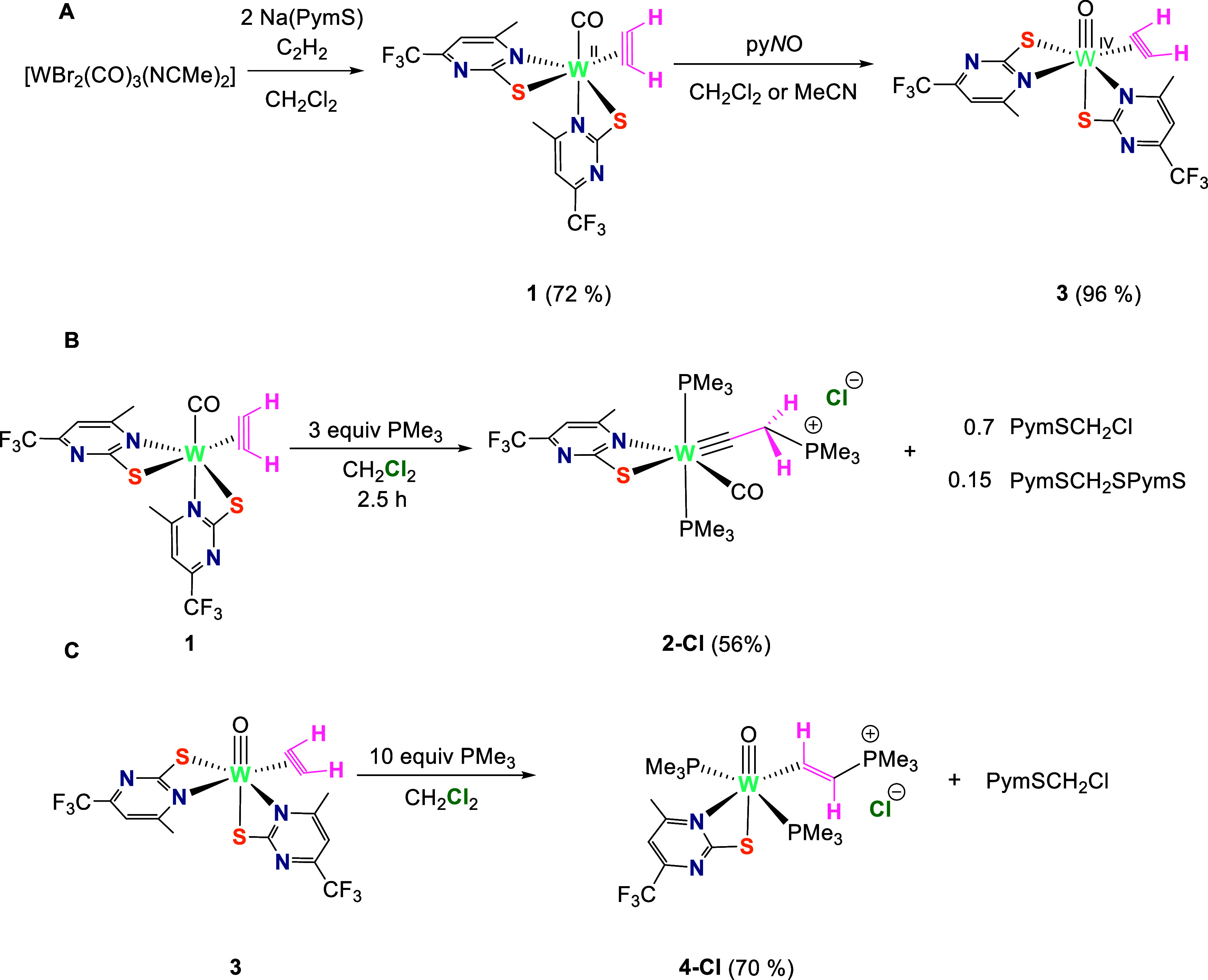
(A): Synthesis of Tungsten Carbonyl and Oxo Complexes Presented in This work. (B): Reaction of Carbonyl Acetylene Complex **1** with 3 equiv of PMe_3_ to the Carbyne Complex **2-Cl** and Organic Byproducts. (C): Reaction of Oxo Acetylene Complex **3** with Excess of PMe_3_ to the Alkenyl Complex **4-Cl** and Organic Byproduct

The W(II) carbonyl complex [W(CO)(C_2_H_2_)(PymS)_2_] (**1**) and the W(IV) oxo complex [WO(C_2_H_2_)(PymS)_2_] (**3**) were synthesized by a procedure established in our group (Scheme 2A).^22,30,32^ PymS ligands were chosen for their higher solubility in polar solvents, when compared to previously studied complexes 6-MePyS complexes. Addition of Na(PymS) to a CH_2_Cl_2_ solution of [WBr_2_(CO)_3_(NCMe)_2_] and subsequent flushing with acetylene gave [W(CO)(C_2_H_2_)(PymS)_2_] (**1**) as a brown powder in 72% yield. The carbonyl complex was further reacted with pyridine-*N*-oxide to yield [WO(C_2_H_2_)(PymS)_2_] (**3**) as light yellow crystals in 96% yield. Both compounds were subjected to the addition of PMe_3_ in CH_2_Cl_2_ leading to the carbyne complex [W(CO)(CCH_2_PMe_3_)(PMe_3_)_2_(PymS)]Cl (**2-Cl**) and η^1^-vinyl complex [WO(CHCHPMe_3_)(PMe_3_)_2_(PymS)]Cl (**4-Cl**), respectively, analogous to our previously reported 6-MePyS system (Scheme 2B,C).^30^ All compounds were fully characterized by spectroscopic means and by single crystal X-ray diffraction analyses. Detailed synthetic procedures and characterization data of the compounds are found in the Supporting Information. Compounds **1** and **3** exhibit properties similar to our previously reported compounds, except for their significantly higher solubility in polar solvents. Thus, we found them suitable for the study of the nucleophilic attack, both in dichloromethane and acetonitrile.

### Formation of the Carbyne Complex (Carbonyl Mechanism)

**Scheme 3:**
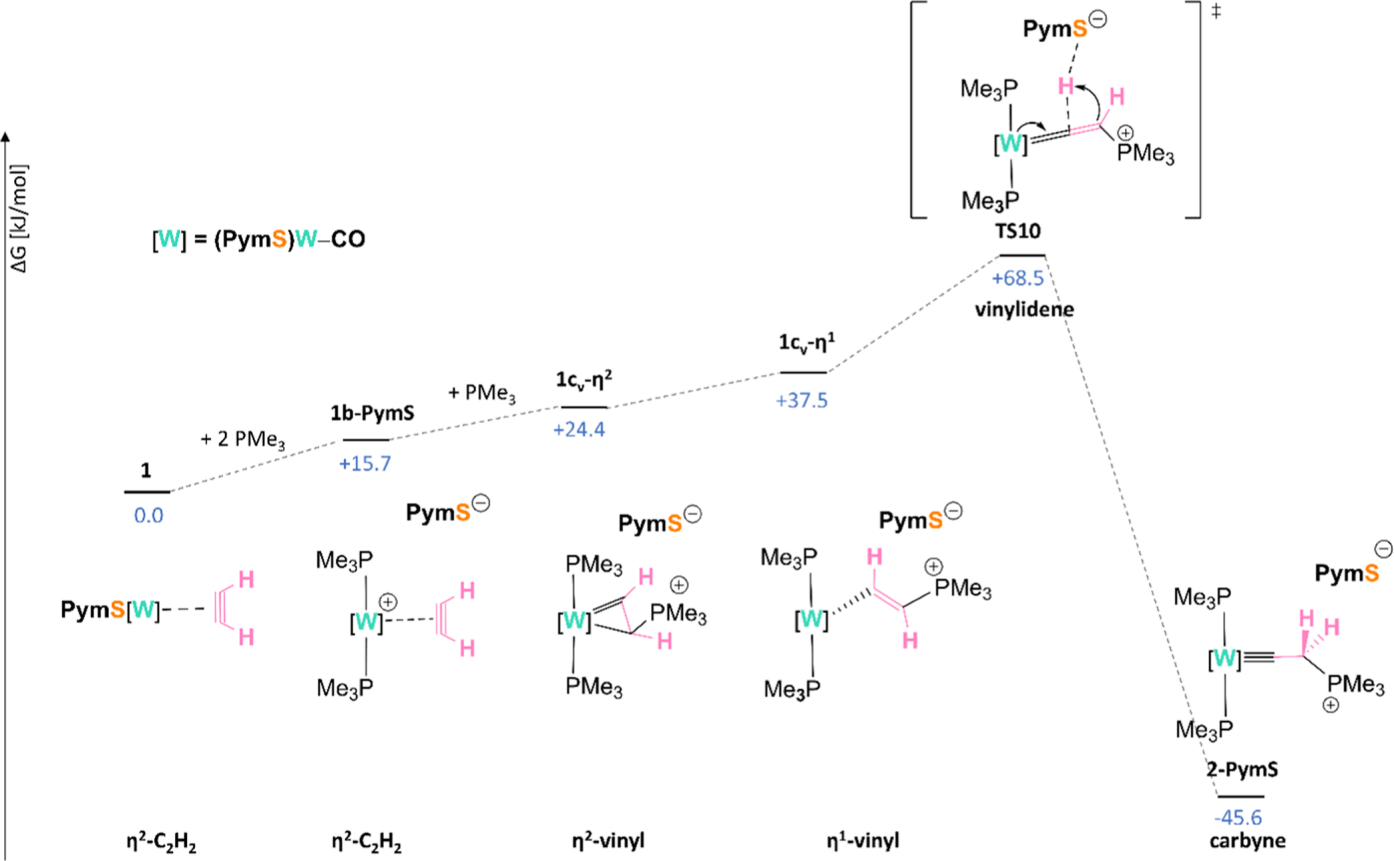
Simplified Reaction Coordinate for the Proposed Mechanism of Acetylene Activation at the W(II) Center and Subsequent Formation of the Carbyne Complex via a Ligand-Assisted 1,2-H Shift The second PymS ligand and carbonyl ligand are omitted for simplicity, as they do not change in the course of the reaction.

In this section, a thorough mechanistic analysis of the reaction depicted in Scheme 2B will be discussed. Considering all our experimental and computational findings (*vide infra*), we propose the reaction mechanism shown in Scheme 3 (simplified reaction coordinate). The full mechanism is presented in Diagram S1, Supporting Information, where all the structures are displayed with the corresponding labels. The initial coordination of two PMe_3_ molecules to the W center in **1** results in the decoordination of one ancillary PymS ligand to form cationic **1b-PymS**. This cationic acetylene intermediate is sufficiently activated for nucleophilic attack, so that the addition of the third equiv of PMe_3_ leads to the η^2^-vinyl intermediate **1c**_**v**_**-η**^**2**^. The latter rearranges to a more reactive η^1^-vinyl intermediate **1c-η**^**1**^ with increased acidity of the α–C–H. The carbyne product **2-PymS** is formed via a vinylidene transition state **TS10** with the thiolate ligand acting as an α-H acceptor. Due to π-back-donation from the W center to the vinylidene ligand, the negative charge is accumulated on the β-C of **TS10**, which (re)deprotonates PymSH, leading to a thermodynamically stable W(IV) carbyne ion pair **2-PymS**. Only in CH_2_Cl_2_, the even more stable **2-Cl** is isolated due to the reaction of the anionic ligand PymS with the solvent.

### Experimental and Computational Evidence for the Carbonyl Mechanism

**Scheme 4:**
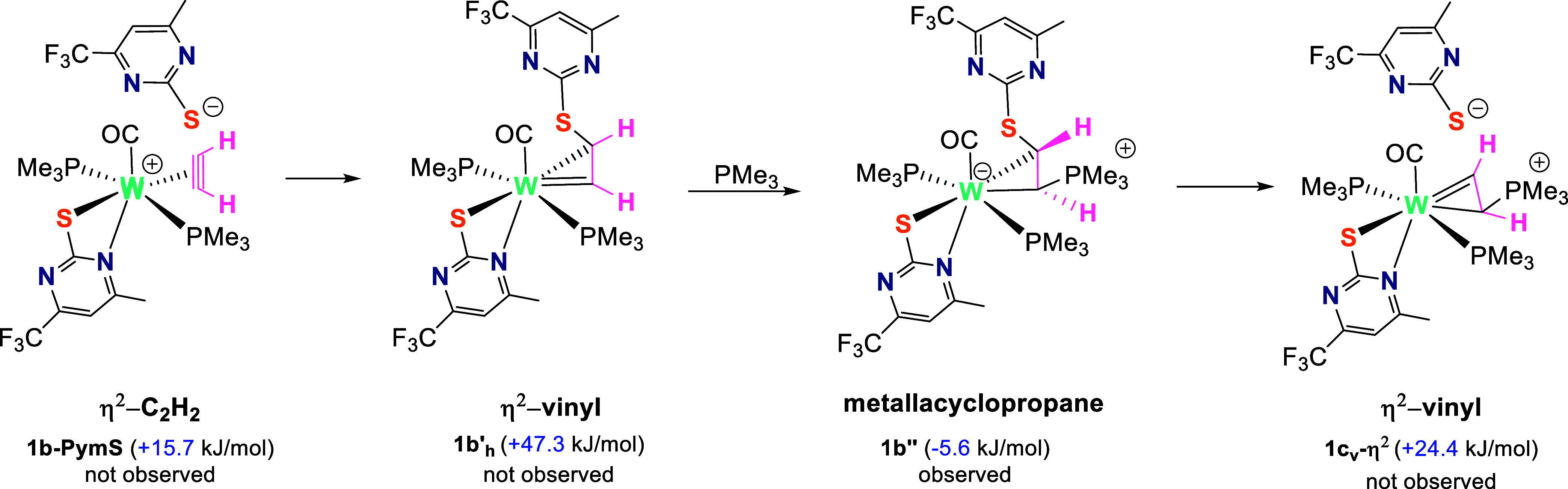
Suggested Mechanism for the Formation of the Intermediate η^2^-Vinyl Complex via the Metallacyclopropane Intermediate **1b″** Δ*G* (kJ/mol) values presented in brackets are obtained from the reaction coordinate for the reaction of 1 with 3 equiv of PMe_3_.

Here, we describe our key findings of the mechanism. More details can be found in the Supporting Information. First, 2 equiv of PMe_3_ coordinate to the metal ion, making the tungsten center cationic. Surprisingly, direct attack at the C_2_H_2_ ligand in **1** is kinetically disfavored by 5.7 kJ/mol only (ΔΔ*G*^TS^ = +66.2 vs +71.9 kJ/mol; see Section 2.1. in DFT Supporting Information). A strong indication that the nitrogen of one PymS ligand decoordinates upon PMe_3_ coordination to end up with a six-coordinate species is provided by the previously published reaction of [W(CO)(C_2_Ph_2_)(6-MePyS)_2_] with 5 equiv of PMe_3_ to selectively give [W(CO)(C_2_Ph_2_)(PMe_3_)(6-MePyS-*S*,*N*)(6-MePyS-*S*)].^22^ To obtain experimental insight into the next steps of the reaction, we increased the amount of **PMe**_**3**_ to **3 equiv**, upon which the ^1^H NMR peaks of coordinated C_2_H_2_ disappeared and a new major species [W(CO)(PymS-*S*-CHCHPMe_3_)(PMe_3_)_2_(PymS-*S*,*N*)] (**1b″**) was formed (Figures S20–S22) which could only transiently be detected at 0 °C. The NMR data suggest that the compound contains two chemically inequivalent tungsten-coordinated PMe_3_ molecules as well as a carbon-bound PMe_3_. Furthermore, the two PymS ligands also remain part of the complex. The intermediate displays two intriguing ^1^H NMR signals at 3.20 and 0.75 ppm (2× dddd) integrating to one proton each, the coupling of which was confirmed by a COSY (Figure S23). Density functional theory (DFT) analysis revealed the order of elementary steps between **1b-PymS** and **1b″** and its further transformation to the η^2^-vinyl intermediate (Scheme 4).

The attack of the free anionic PymS ligand at the coordinated acetylene in **1b-PymS** is favored by 68.5 kJ/mol (ΔΔ*G*^TS^ = +16.6 kJ/mol for PymS attack vs 85.1 kJ/mol for PMe_3_ attack; see Section 2.2.3. in DFT Supporting Information). Such an attack leads to the formation of the high energy intermediate [W(CO)(PymS-*S*-CH=CH)(PMe_3_)_2_(PymS-*S*,*N*)] (**1b′**_**h**_), which further reacts with PMe_3_ to lower the energy of intermediate **1b″**, which is observed in the NMR experiments. Structural optimization showed that **1b″** features the tungstocyclopropane moiety in a horizontally aligned fashion (perpendicular to the W–CO bond) with the two hydrogen atoms located *trans* to each other. Aside from **1b″**, a considerable amount of [W(CO)(C≡CH_2_PMe_3_)(PMe_3_)_2_(PymS)]^+^, the cation of the final product **2-Cl**, was detected in CD_2_Cl_2_ (Figure S20). Here, the formation of PymSCD_2_Cl was observed in CD_2_Cl_2_, while this species cannot be generated in CD_3_CN. Thus, we conclude that the formation of **2-Cl** proceeds via the analogous carbyne species [W(CO)(CCH_2_PMe_3_)(PMe_3_)_2_(PymS)][PymS] (**2-PymS**). This is supported by NMR data of the deprotonated ligand in both solvents (Tables S3) and explains why the carbyne cation is also observed in CD_3_CN solutions. When the CD_2_Cl_2_ reaction mixture was left at room temperature for 45 min before the next ^1^H NMR measurement, PymS fully converted to PymSCD_2_Cl. To verify that PymS can react with CH_2_Cl_2_ without being coordinated to a metal center, a mixture of Na(PymS), Bu_4_NCl, and CH_2_Cl_2_ was stirred for 24 h. The addition of Bu_4_NCl was necessary as the sodium salt alone is almost insoluble in CH_2_Cl_2_, and, hence, reacts only very slowly with the solvent. Evaporation to dryness and subsequent analysis by ^1^H NMR spectroscopy confirmed the selective formation of PymSCH_2_Cl (Figure S24).

The experimental observations are readily explained by the DFT calculations. Following the formation of **1b″**, PymS is expelled from the tungstocyclopropane moiety, yielding the high-energy η^2^-intermediate **1c**_**v**_**-η**^**2**^, which subsequently is converted to its η^1^**-**form **1c-η**^**1**^. Again, a horizontally oriented intermediate is also formed but is omitted for simplicity (see Section 2.2.3. in DFT Supporting Information). The hydrogen shift from **1c-η**^**1**^ to **2-PymS** occurs via a vinylidene transition state **TS10** corresponding to a surprisingly low energetic barrier of only +31.0 kJ/mol, which is facilitated by the basic nature of the decoordinated PymS ligand anion. The latter deprotonates the α-carbon and transfers the proton onto the β-carbon, acting as a hydrogen shuttle. Indeed, a comparable transition state where the PymS ligand anion is not in proximity to the hydrogen being transferred results in an exceedingly large energetic barrier of +127.5 kJ/mol (see Section 2.2.3. in DFT Supporting Information). Notably, attack of PMe_3_ at the C_2_H_2_ ligand of **1b-PymS** would directly lead to the formation of **1c**_**v**_**-η**^**2**^. While this route is overall kinetically disfavored (ΔΔ*G*^TS^ = +85.1 kJ/mol), it cannot totally be ruled out, leading to an additional potential route toward **1b″** (corresponding to the back-reaction of the preferred pathway from **1c**_**v**_**-η**^**2**^; for a detailed discussion, see Section 2.2.3.1. in DFT Supporting Information). Therefore, the formation of the cationic complex **1b-PymS** activates the acetylene for nucleophilic attack. Finally, the free PymS reacts with CH_2_Cl_2_, yielding the final ion pair **2-Cl**. The slow rate of this transformation is easily explained by the associated comparably large energetic barrier (ΔΔ*G*^TS^ = +85.6 kJ/mol).

### Formation of the η^1^-Vinyl Complex 4-Cl (Oxo Mechanism)

**Scheme 5:**
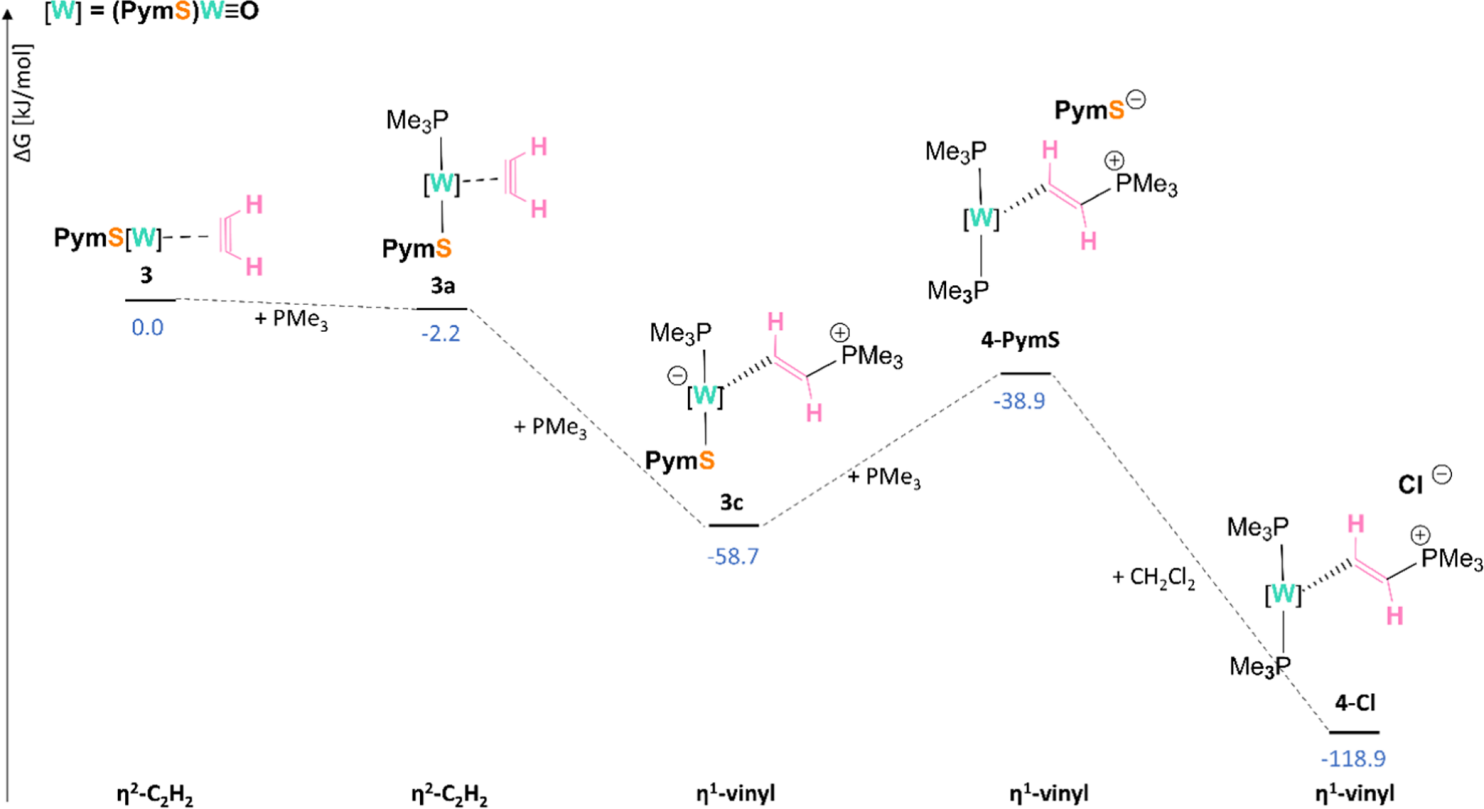
Simplified Reaction Coordinate for the Proposed Mechanism of Acetylene Activation at W(IV) Center and the Subsequent Formation of the Vinyl Complex **4-Cl** The second PymS and oxo ligands are omitted for simplicity, as they do not change in the course of the reaction.

In this section, a thorough mechanistic analysis of the reaction depicted in Scheme 2C will be discussed. Our experimental and theoretical evidence (*vide infra*) suggest the reaction mechanism as shown in Scheme 5 (simplified reaction coordinate). The full mechanism is presented in Diagram S2 in Supporting Information, where all structures are displayed with the corresponding labels. Similar to the above-described mechanism, the transformation starts with the initial coordination of one PMe_3_ molecule to the W center in **3**, yielding compound **3a**, in which acetylene is sufficiently activated for the nucleophilic attack by the phosphine leading to the η^1^-vinyl complex **3c**. Further reaction with a third equiv of phosphine leads to the cleavage of the W–S bond, forming the vinyl complex **4-PymS** and, subsequently, the chloride salt **4-Cl**. The formation of the latter occurs due to the reaction of the anionic ligand with dichloromethane, as explained above, in the carbonyl mechanism.

### Experimental and Computational Evidence for the Oxo Mechanism

**Figure 1:**
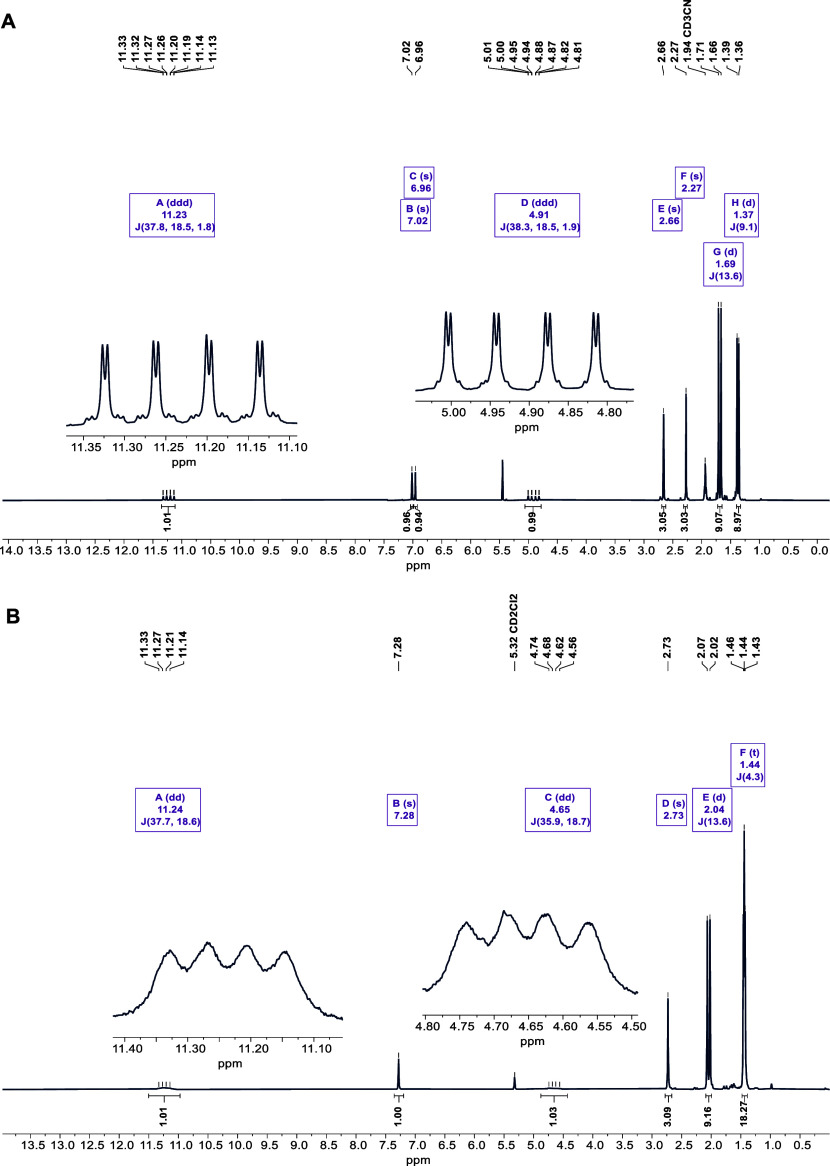
(A): ^1^H NMR spectrum of **3c** recorded in CD_3_CN. (B): ^1^H NMR spectrum of **4-Cl** recorded in CD_2_Cl_2_. Resonances of the ethenyl protons are highlighted in both spectra.

Again, the thorough mechanistic analysis of the mechanism is described in the Supporting Information. NMR spectra of CD_2_Cl_2_ or CD_3_CN solutions of **3** with **1.1 equiv of PMe**_**3**_ reveal the presence of [WO(C_2_H_2_)(PMe_3_)(PymS)_2_] (**3a**), showing that the first PMe_3_ coordinates to the tungsten center. The attack at the C_2_H_2_ ligand of **3** is both thermodynamically and kinetically highly disfavored (ΔΔ*G*^TS^ = +63.3 vs + 86.0 kJ/mol; Δ*G* = +20.8 vs +42.4 kJ/mol). The reaction mixture with **1.1 equiv of PMe**_**3**_ already contained a small share of the vinyl species [WO(η^1^-CH_α_-CH_β_PMe_3_)(PMe_3_)(PymS-*S*,*N*)(PymS-*S*)] (**3c**, Figures S25–S30). After increasing the amount of PMe_3_ to **2.2 equiv**, **3c** was formed with high selectivity in various solvents (THF, MeCN, CHCl_3_, and CH_2_Cl_2_). The reaction was most selective in CH_2_Cl_2_, from which we obtained a pure sample directly after solvent evaporation without further workup. The formation of **3c** is supported by the ^1^H NMR spectrum (CD_2_Cl_2_, Table S2), where the shifts and coupling patterns of the ethenyl protons (2× ddd, flanked with ^183^W satellites, 11.41 ppm for WCH and 4.96 ppm for PCH) are very similar to those of [IrBr(CO)(CH_3_)(−CH=CHPPh_3_)_2_(PPh_3_)]^+^.^33^ Furthermore, upon treating starting complex **3** with **3.3 equiv of PMe**_**3**_ the final product 4-Cl is selectively formed (Figure S29). Calculations support this course of the reaction, as attack of the second PMe_3_ at the tungsten center of **3a** is predicted to yield a thermodynamically extremely unstable product (**3b@W**, Δ*G* = +74.8 kJ/mol). Overall, the formation of **3c** is driven by the exergonicity of the overall reaction (Δ*G* = −58.7 kJ/mol). Moreover, upon the reaction of **3** with **3.3 equiv of PMe**_**3**_ in CD_3_CN, besides major product **3c**, resonances consistent with those of [WO(CHCHPMe_3_)(PMe_3_)_2_(PymS)][PymS] (**4-PymS**) were observed in the ^1^H NMR spectrum after 20 min of reaction which increase with time (Figure S30). This is also supported by the fact that the resonances of the free PymS are well in accordance with NMR data of Na(PymS) (Tables S2 and S3). Furthermore, the reaction of **3** with **3.3 equiv of PMe**_**3**_ in CD_2_Cl_2_ was followed by ^1^H NMR spectroscopy at specific time intervals revealing no full conversion of **3c** to **4-Cl** even after 22 h (Figure S29). ^1^H NMR spectra of **3c** and **4-Cl** are shown in Figure 1. The relative energies of **3c** and **4-PymS** (Δ*G* = −58.7 and −38.9 kJ/mol) support the formation of a mixture of both **3c** and **4-PymS** in the reaction vessel. Using CH_2_Cl_2_ as a solvent, **4-PymS** can subsequently undergo further reaction to the ion pair **4-Cl** and PymSCH_2_Cl, again driven by the corresponding energy gain of the reaction (ΔΔ*G* = −60.2 kJ/mol vs **3c**).

### Influence of PR_3_

To fine-tune the electrophilic reactivity of acetylene complexes **1** and **3**, respectively, we investigated the attack of three additional phosphines with variable steric size and electronic properties, namely PMe_2_Ph, PMePh_2_, and PPh_3_. Excess of the respective phosphine (4 equiv) was added to CD_3_CN solutions of complexes **1** and **3**. NMR spectra of the corresponding reactions are presented in the Supporting Information (Figures S35–S46). The general outcome is summarized in Table 1.

**Table 1:** Outcome of the Reactions of **1** and **3**, Respectively, with 4 Different Phosphine Nucleophiles in CD_3_CN

outcome	PMe_3_	PMe_2_Ph	PMePh_2_	PPh_3_
**1 + PR**_**3**_	carbyne	carbyne	complex mixture	no reaction
**3 + PR**_**3**_	η^1^-vinyl	η^1^-vinyl	η^1^-vinyl	no reaction

NMR spectra (Figures S35–S42) of the reactions employing PMe_2_Ph revealed the formation of largely analogous complexes as compared to PMe_3_, namely the carbyne compound [W(CO)(CCH_2_PMe_2_Ph)(PMe_2_Ph)_2_(PymS)](PymS) and η^1^-vinyl compound [WO(η^1^-CHCHPMe_2_Ph)(PMe_2_Ph)(PymS-*S*,*N*)(PymS-*S*)]. The chloride salt of the carbyne complex could be isolated after workup in 67% yield, as red microcrystalline powder (see Supporting Information, synthetic procedures). ^31^P{^1^H} NMR spectroscopy of the W oxo η^1^-vinyl compound confirms the presence of only one W coordinated phosphine as the second phosphine coordination is sterically prevented.

Interestingly, bulkier PMePh_2_ showed significantly different reactivity with compounds **1** and **3**. Reaction with **1** led to an inconclusive reaction mixture with several acetylene complexes and a carbyne complex as a minor product (Figures S43 and S44) which is presumably due to the weaker nucleophilicity of PMePh_2_, in comparison to PMe_3_ and PMe_2_Ph. Another reason for lower selectivity is sterically prevented coordination at the W center, which slows down formation of the cationic complex which would be more reactive with the nucleophile, as described in the previous chapter. However, tungsten(IV) acetylene complex **3** reacted selectively with PMePh_2_ to [WO(η^1^-CHCHPMePh_2_)(PMePh_2_)(PymS-*S*,*N*)(PymS-*S*)], an analog of **3c** (Figures S45 and S46). Further coordination of the phosphine to obtain an analog of **4-PymS** is again prevented for steric reasons. Finally, reactions involving bulky and electron-deficient PPh_3_ have not led to the observation of new compounds. These comparative studies reveal that the complex with the W(IV) center in **3** is significantly more reactive toward nucleophilic attack.

### Vinyl vs Carbyne Complex Formation

This chapter discusses the thermodynamic differences between vinyl and carbyne complexes in two different reaction mechanisms. The aim is to explain why the carbyne formation is favored in the carbonyl mechanism but not in the oxo mechanism, focusing on Gibbs energy differences, bond orders, and electronic structures.

**Figure 2:**
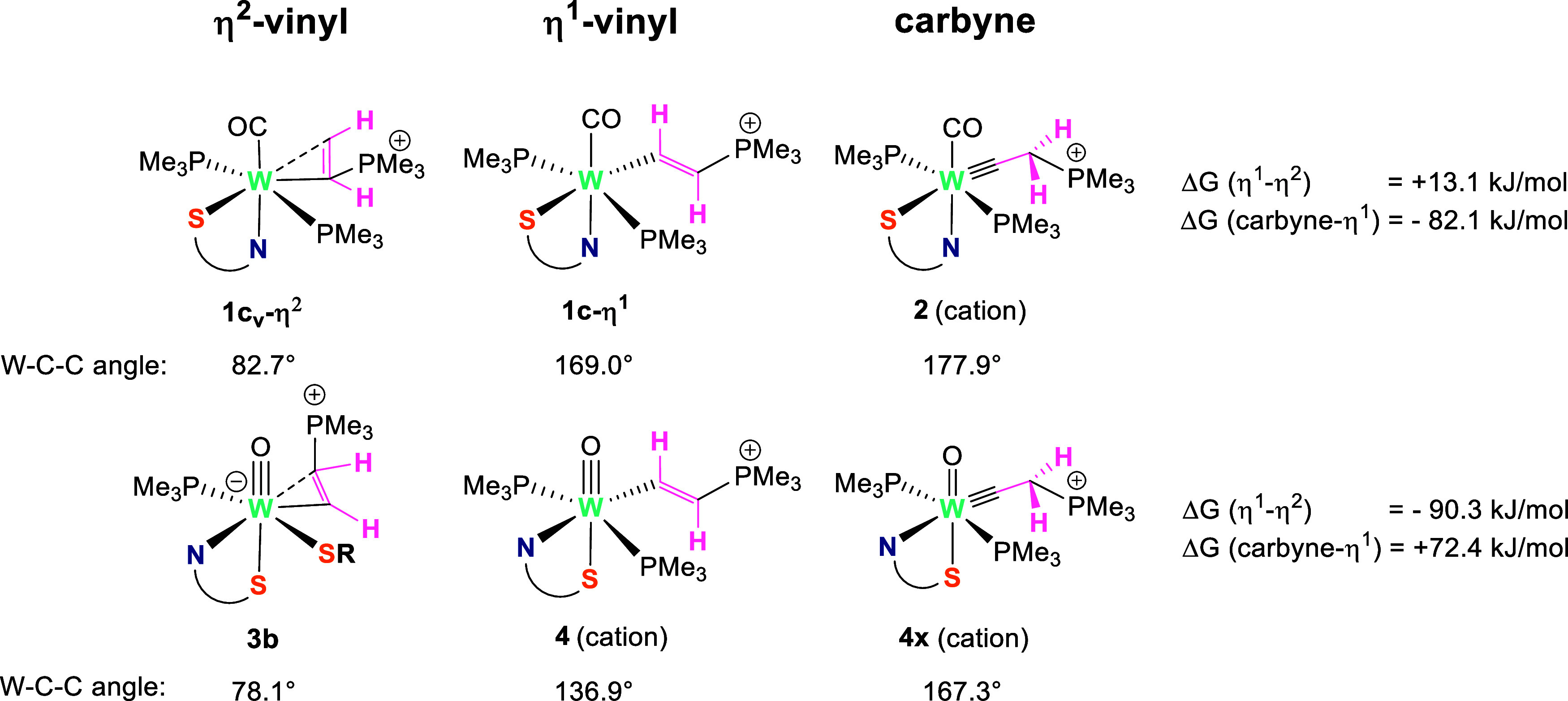
Comparison of the relative Gibbs energies and angles of the η^2^-vinyl, η^1^-vinyl and the carbyne species in both carbonyl and oxo mechanisms. For the ion pairs, only the cations are considered. The species **4x** is virtual, as no carbyne complex is observed in the oxo mechanism.

We now look at **thermodynamic differences** between vinyl and carbyne ligands in our complexes. The different nature of the tungsten acetylene binding in complexes **1** and **3**, highly influences the reaction outcome upon nucleophilic attack. Notably, an η^1^-vinyl species is formed in both cases, but only with the carbonyl complex, it reacts further to the carbyne species. To understand such a distinct behavior, we calculated the Gibbs energies for the η^1^-vinyl, η^2^-vinyl and the carbyne species in both mechanisms, alongside the angles between the W center and two acetylene-deriving carbon atoms (Figure 2). Since the carbyne complex does not appear in the oxo mechanism, a virtual species has been computed for better comprehension. We only consider the cationic part of the η^1^-vinyl and carbyne complexes to eliminate any influence of the geometric arrangement of the counterions and the reaction energy of the PymS with the solvent.

In the carbonyl mechanism the η^2^-vinyl complex is favored over its η^1^-isomer by 13.1 kJ/mol, while in the oxo mechanism, the η^1^-oxo cation **4** is favored over the η^2^-vinyl complex **3b** by 90.3 kJ/mol. As described above, the carbonyl mechanism proceeds to the final carbyne product **2-Cl**, strongly favored over the η^1^-isomer **1c-η**^**1**^ by 82.1 kJ/mol. In contrast, the oxo reaction stops at the η^1^-complex **4-Cl** without forming the potential carbyne product **4x**. Energetically, **4x** is markedly disfavored over **4-Cl** by +71.2 kJ/mol. Hence, the relative stabilities of the η^2^- and η^1^-vinyl and of the η^1^-vinyl and carbyne complexes are reversed in the two mechanisms.

This trend can be explained by the calculated NBO charges (for more details see Section 4 in DFT Supporting Information): The tungsten in **4-PymS** bears a positive partial charge of +1.000, while that in **1c-η**^**1**^ a negative partial charge of −0.187 (Table 7 in DFT Supporting Information). The latter exhibits a negative charge due to the donating character of PMe_3_ ligands and the lower oxidation state in comparison to the former.^37^ This leads to a **different tendency toward electron donation onto the η**^**1**^**-ligand**, causing increased W–C_α_ bond order in **1c-η**^**1**^ as compared to **4-PymS** (1.562 vs 1.098), a concomitant reduction of the C_α_=C_β_ double bond character (1.480 vs 1.532) and linearization of the W–C_α_-C_β_ bond angle (169.0 vs 136.9°). Additionally, while in **4-PymS**, the W=O bond order is 1.994, in the hypothetical carbyne complex, it markedly drops to 1.736. On the contrary, in the carbonyl mechanism, upon going from **1c-η**^**1**^ to the carbyne product, the W–C=O bond order displays no significant change (1.275 vs 1.293). This hints that the tungsten in the oxo mechanism is too electron-deficient to stabilize simultaneously both an oxo and a carbyne ligand.

## Discussion

### Factors Governing the PMe_3_ Attack

This chapter highlights the critical steric and electronic factors that govern the attack at tungsten-coordinated acetylene, leading to distinct differences in the reactivity of compounds **1** and **3**.

#### Steric Effects

Upon reactions with the first equivalent of PMe_3_, both compounds **1** and **3** first undergo associative substitution of the nitrogen atom of the equatorial PymS ligand by PMe_3_, leading to compounds **1a** and **3a**. In the case of the carbonyl mechanism, the tungsten center of **1a** (see Section 2.1. in DFT Supporting Information) subsequently coordinates a second molecule of PMe_3_, while in the oxo mechanism, C_2_H_2_ ligand in **3a** gets attacked. The energy of the addition of PMe_3_ to W increases in both mechanisms with each PMe_3_ due to **steric crowding**. Whereas attack of the first PMe_3_ at **1** and **3** exhibits an energy barrier of +66.2 and +63.3 kJ/mol, respectively, attaching the second PMe_3_ to **1a** requires +74.2 kJ/mol, and attaching the second PMe_3_ to **3a** is energetically unreasonable. Hence, in **3a** the second PMe_3_ attacks the acetylene. In addition, compounds **1a** and **3a** exhibit different **orientations of the acetylene ligand** vs W-CO and W–O bonds. Namely, **1a** features the C_2_H_2_ ligand oriented in a vertically aligned fashion (to the W–CO), while in **3a** it is aligned horizontally (to the W–O). Therefore, the latter has less space to accommodate an additional tungsten-bound PMe_3_, as reflected in the high energy of the corresponding hypothetical product (Δ*G* = +74.7 kJ/mol).

#### Electronic Effects

As previously mentioned, addition of the second equivalent of PMe_3_ in the carbonyl mechanism causes cation formation (**1b-PymS**), which finally allows for the attack at the acetylene ligand. This is supported by the NBO charges at the acetylenic carbon atoms which increase from −0.230 in neutral **1** to −0.074 in cationic **1b-PymS** rendering the acetylene ligand more electrophilic. In the oxo mechanism the metal is more electron-deficient leading to an electrophilic acetylenic ligand, and allowing for nucleophilic attack without the need of cation formation. These findings underscore the critical role of an **electron-poor metal center** for the activation of acetylene in the enzyme AH.

#### Role of the Metal Oxidation State

**Scheme 6:**
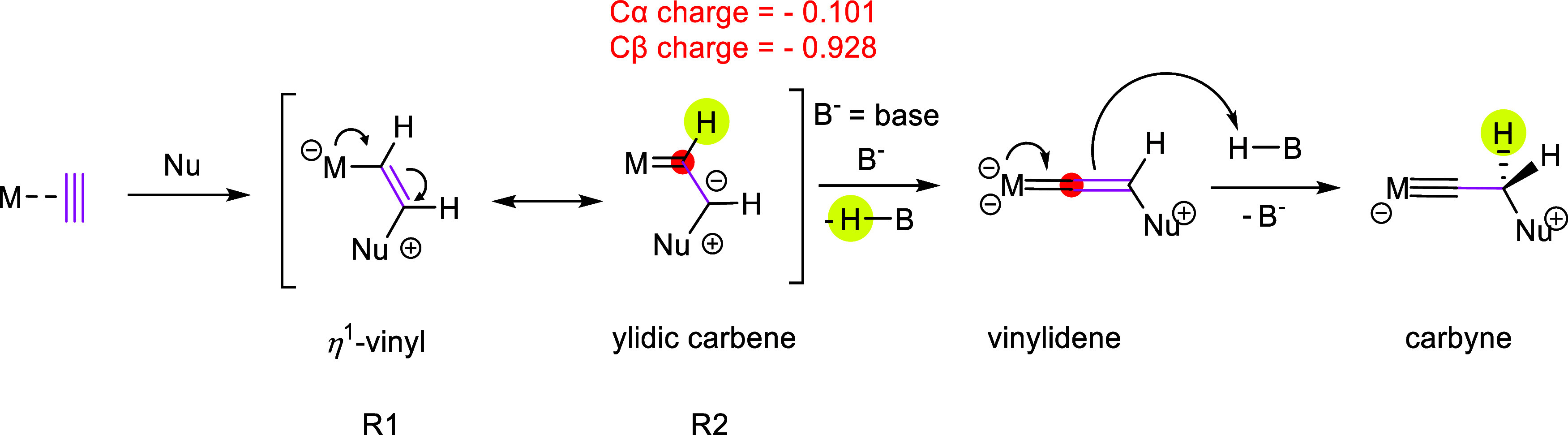
Reactivity of Acetylene Complexes Described with Relevant Resonance Structures

Upon nucleophilic attack, the η^1^-vinyl species is formed in both mechanistic cases, but only with the carbonyl complex, it reacts further to the carbyne species. Furthermore, due to the strong electron donation of the central tungsten atom in the η^1^-vinyl species (represented via resonance structures R1 and R2 in Scheme 6), the associated partial double bond character of the W–C_α_ bond, and the accompanying W–C_α_–C_β_ linearization, the C_α_ exhibits only a weak negative partial charge (−0.101), rendering the attached hydrogen acidic (Scheme 6). This enables the nearby free PymS to deprotonate the C_α_, again highlighting the important role of the formation of the cation, and the in situ generation of the required base. The cation formation enhances the charge separation between C_α_ and C_β_. Furthermore, the negative partial charge localized on the C_β_ (−0.928) is markedly greater than that on the C_α_ (−0.101), which may be interpreted as an increased basicity. This drives the proton transfer from the thiolate base to the C_β_ leading to the formation of the observed carbyne product (Scheme 6), thereby providing a rationale for the associated low energetic barrier. Thus, in the oxo mechanism due to the absence of π-back-donation, the final product remains the η^1^-vinyl species.

The carbene contribution (R2) to the R1-R2 resonance hybrid in the carbonyl system allows for negative charge buildup on the β-carbon atom attached to the phosphine relative to the α-carbon coordinated to tungsten. However, the direct 1,2-H shift to yield the carbyne product is inhibited by a very high energy computed transition state. This high energy transition state is consistent with the Bell–Evans–Polanyi principle,^38^ whereby the reorganizational energy for the direct 1,2-H shift is large, coupled with a small energy difference between the vinyl and carbyne species. The degree of mixing in the R1-R2 resonance hybrid in Scheme 6 also contributes to the partial double bond character between the W ion and the α carbon of the ligand, leading to an increased linearization of the W–C_α_–C_β_ bond angle (Figure 2) relative to the vinyl (resonance structure R1), resulting in a more acidic H located on the α carbon.

In summary, a sterically crowded and electron-deficient metal center is necessary to facilitate nucleophilic attack on the coordinated acetylene. The final product of the nucleophilic attack at the metal-coordinated acetylene depends on the capability of the metal to stabilize the initially formed vinyl species, as presented in Scheme 6 in the form of Lewis structures.

### Revisited Nucleophile Attack at Coordinated Acetylene

**Scheme 7:**
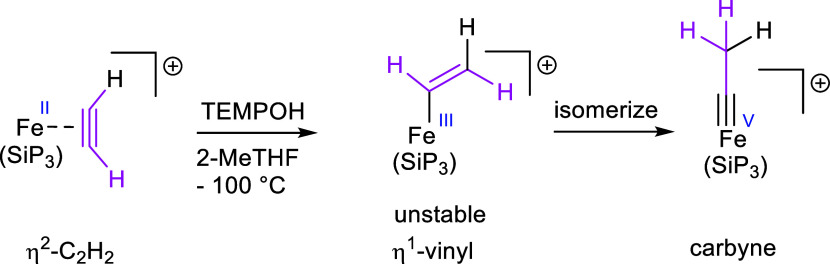
Formation of the Carbyne Complex Starting from an Fe(II)–C_2_H_2_ Adduct^29^^,^ Reproduced or adapted with permission from ref (29), Copyright [2019] American Chemical Society.

Limitations were encountered in understanding carbyne formation based on the reactivity classification presented in Scheme 1. Here we present an extended classification of the η^2^-alkyne adducts reactivity, additionally including carbyne complexes. The formation of the carbyne species from complex **1**, was only possible due to tungsten’s ability to stabilize the intermediate vinyl complexes via back-donation. Thus, it is noteworthy that in the case of complex **1**, acetylene acts as a four-electron donor, contrasting the two-electron donor C_2_H_2_ in complex **3**. Chemical shifts of acetylenic carbons in ^13^C NMR spectra are good predictors for distinguishing the amount of donated electrons, but the method is limited to diamagnetic compounds.^34^ Interestingly, M–C bond lengths were found to be more affected by the type of the alkyne donor than the corresponding elongation of the C–C bond. Precisely, two-electron donor alkynes are exhibiting nearly 0.1 Å longer M–C bonds than the corresponding four-electron donor alkynes.^35^ Four-electron donor alkynes in Mo and W complexes usually exhibit M–C bonds at 2.03 ± 0.03 Å.^23^ A similar trend was also observed for other metals, for example, in [CpOs(Cl{η^2^-HC≡CC(OH)Ph_2_}(P^i^Pr_3_))] with Os–C1 2.142(7) Å and Os–C2 2.163(6) Å. Upon dissociation of chloride the increase in electron donation by acetylene leads to contraction of the Os–C bonds to 1.992(9) and 1.981(8) Å.^36^ In their fascinating work on an Fe–C_2_H_2_ adduct, Peters and co-workers observed that the paramagnetic Fe(II)-C_2_H_2_ adduct reacts with TEMPOH to a carbyne species via reductive protonation as presented in Scheme 7.^29^

**Scheme 8:**
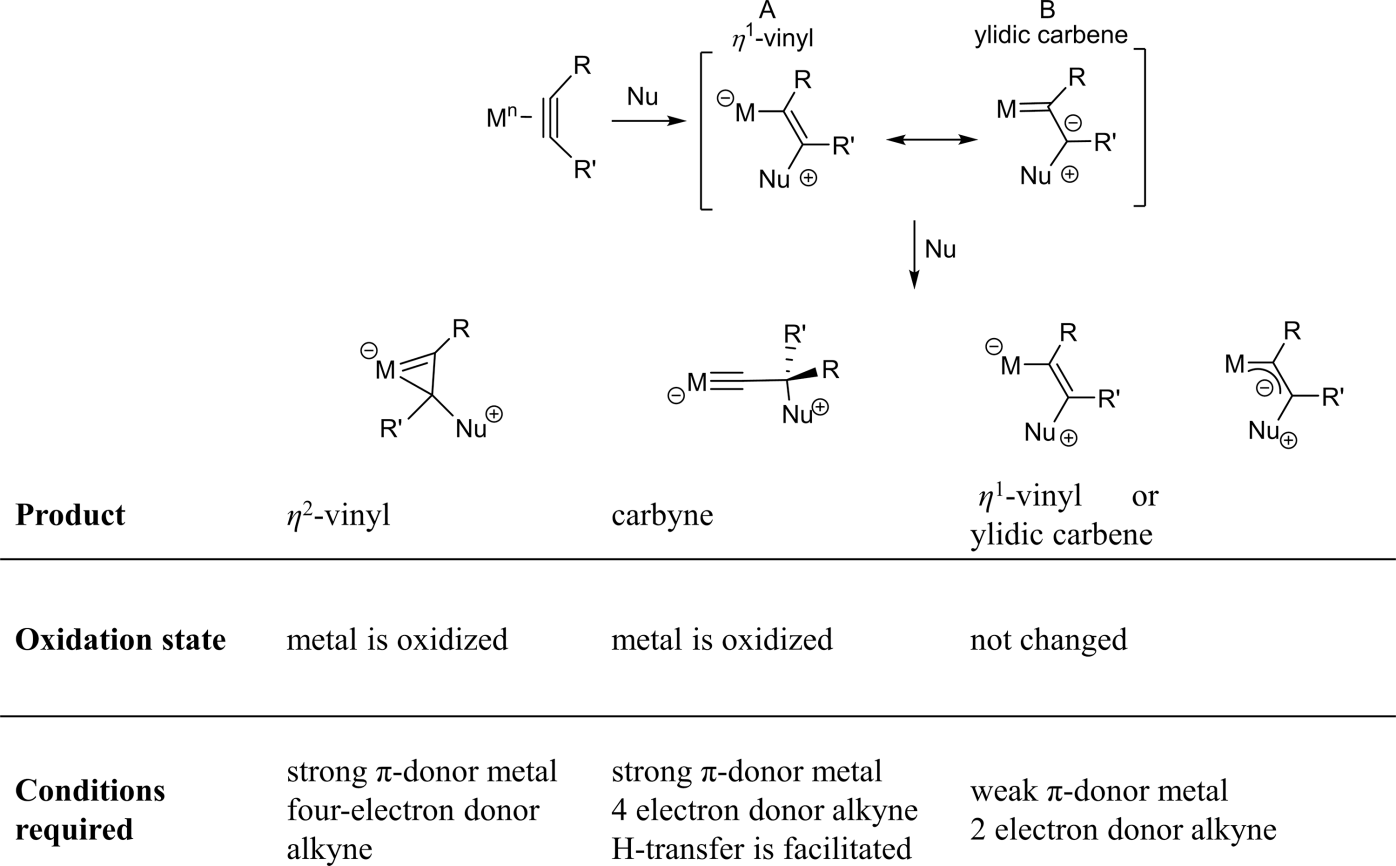
Revisited Nucleophile Attack at Coordinated Alkynes

The Fe-to-acetylene back-bonding in the η^2^-adduct is described to be moderate according to the C≡C bond length of 1.25 vs 1.20 Å in free acetylene. The paramagnetism of the species prevented the discussion of alkyne bonding via ^13^C NMR spectroscopy. As previously mentioned, the type of the alkyne donor is more accurately determined via the M-C bond, which are 1.9649(1) and 1.9972(1) Å in the Fe(II)–C_2_H_2_ adduct. Such bond length points toward a strong back-bonding and characterizes the starting acetylene ligand rather as a four-electron donor. In the presence of TEMPO, isomerization from the η^1^-vinyl to the carbyne species occurs via a radical mechanism, contrasting the ionic mechanism found in this work. Although two different mechanisms are suggested for the same transformation, in both cases, the hydrogen transfer is facilitated. Based on the analysis in this manuscript, we propose the extensions of Templeton’s rules for nucleophilic attack on coordinated alkynes as presented in Scheme 8. It is worth emphasizing that the rules only apply to stable η^2^-alkyne adducts and not other cases where the addition of acetylene as a reagent directly leads to vinylidene complexes.

In short, starting from isolated η^2^-alkyne complexes, carbyne species are formed from four-electron donor alkyne ligands where R-group transfer is allowed. The type of the alkyne donor (two- or four-electrons) is best described by the M-C bond lengths in η^2^-alkyne complexes and not with the C≡C bond length.

## Conclusions

In conclusion, this study provides new insights into the mechanisms governing nucleophilic attack on W-acetylene adducts by a combination of spectroscopic and DFT analyses. Our findings highlight the critical role of both steric and electronic factors in facilitating such attacks. Specifically, steric congestion at the tungsten center is required to shield the metal from the incoming phosphine, allowing the acetylene ligand to be targeted instead. Additionally, an electron-deficient tungsten center, as found in W(IV) compounds, is essential for activation of the acetylene ligand. This was further corroborated by the higher reactivity of a weaker nucleophile toward the here investigated W(IV) vs W(II) acetylene species. Regardless of the tungsten oxidation state, nucleophilic attack results in the formation of an η^1^-vinyl complex. Only in the W(II) case, reaction to the carbyne occurs due to stabilization by π-back-donation. This reaction is accompanied by an unusual ligand-assisted 1,2-H shift, highlighting the importance of the second coordination sphere in this organometallic transformation. Moreover, considering the nature of the acetylene ligand, only a four-electron donor acetylene can form the carbyne species. The activation of acetylene at the biologically relevant oxidation state +IV supports the potential tungsten(IV) acetylene intermediate in the AH mechanism. Moreover, further studies on the reactivity of bioinspired W-acetylene model compounds should consider the involvement of thiolate and carboxylate moieties in the vicinity of the metal center as they may simulate the role of cysteine and aspartate residues in AH.

## Methodology

All synthetic manipulations and NMR experiments were performed under nitrogen atmosphere using standard Schlenk and glovebox techniques.

All DFT calculations were performed with TURBOMOLE 7.4.1.^39−41^ Geometries were optimized employing the PBE^42^ functional together with the D3 dispersion correction using Becke–Johnson damping^43,44^ and the dhf-SVP basis set.^45^ To account for relativistic effects occurring for the central tungsten atom, the corresponding dhf-ecp (effective core potential) was utilized.^46^ Further details are described in the Supporting Information.

## Supporting Information Available

Full experimental procedures; basic computational data; NMR spectra and crystallographic data CCDC 2294570–2294573 (PDF)
Detailed description of the final mechanisms and alternative scenarios (PDF)
*XYZ* coordinates (ZIP)
