# Supplementary material for: Understanding the Carbyne Formation from C2H2 Complexes
Source: J Am Chem Soc. 2024 Nov 15;146(47):32392–402. doi: 10.1021/jacs.4c07724 (PMC11613314; doi:10.1021/jacs.4c07724)
Supplement: Supplementary file 1 — ja4c07724_si_001.pdf [file ja4c07724_si_001.pdf]

Supporting Information

Understanding the Carbyne Formation From C_2H_2 Complexes

Miljan Z. Ćorović, [‡,a] *Madeleine A. Ehweiner,* [‡,a] *Peter E. Hartmann,* [‡,b] *Felix Sbüll,* [b] *Ferdinand Belaj,* [a] *A. Daniel Boese,* [b] *Jesse Lepluart,*[c] *Martin L. Kirk,*[c] *Nadia C. Mösch-Zanetti**[,a]

[a]Institute of Chemistry, Inorganic Chemistry, University of Graz, Schubertstrasse 1, 8010, Graz, Austria

[b]Institute of Chemistry, Physical and Theoretical Chemistry, University of Graz, Heinrichstrasse 28, 8010, Graz, Austria

[c]Department of Chemistry and Chemical Biology, The University of New Mexico, MSC03 2060, 1 University of New Mexico, Albuquerque, NM 87131-0001, United States

[‡]These authors contributed equally

Table of Contents

1 General Considerations 3

2 Synthetic Procedures 3

3 DFT Calculations 7

4 Mechanistic Elucidation 8

5 NMR Spectra of Isolated Compounds 19

6 NMR Data of All Relevant Compounds 36

6.1 Carbonyl Complexes 36

6.2 Oxo Complexes 39

6.3 Organic Compounds 42

7 NMR Experiments 43

8 X-Ray Crystallographic Data 73

9 References 81

1 General Considerations

All synthetic manipulations and NMR experiments were performed under a nitrogen atmosphere using standard Schlenk and glovebox techniques. Solvents were purified via a Pure Solv Solvent Purification System. Chemicals were purchased from commercial sources, and apart from acetylene, sodium hydride, and pyridine *N*-oxide, all were used without further purification. Acetylene 2.6 was purified by bubbling it through water and conc. H_2SO_4 and subsequently dried by passing it through $CaCl_2$ and KOH. Pyridine *N*-oxide was purified by recrystallization from Et_2O and subsequent sublimation. NaH 60% in mineral oil was washed with pentane to give pure NaH. Celite was dried at 100 °C prior to use, and silica gel was washed with Et_3N and subsequently dried in vacuo under heating. The metal precursor complex $[WBr_2(CO)_3(NCMe)_2]$[1] was synthesized according to established procedures. NMR spectra were recorded on Bruker Avance III 300 MHz and 700 MHz spectrometers. Chemical shifts δ are given in ppm. ^1H NMR spectra are referenced to residual protons in the solvent and ^{13}C NMR spectra to the deuterated solvent peak. The multiplicity of peaks is denoted as singlet (s), doublet (d), triplet (t), quadruplet (q), doublet of doublets (dd), doublet of doublets of doublets (ddd), doublet of triplets (dt), triplet of doublets (td), or multiplet (m). NMR solvents were stored over molecular sieves in the glovebox. NMR experiments were performed in J. Young NMR tubes. Solid-state IR spectra were measured on a Bruker ALPHA ATR-FT-IR spectrometer at 2 cm^{-1} resolution. The relative intensity of signals is declared as strong (s), medium (m), weak (w), and very weak (vw). Elemental analyses (C, H, N, S) were performed at the Department of Inorganic Chemistry at the University of Technology in Graz. Values for elemental analyses are given as percentages.

2 Synthetic Procedures

Ligand Synthesis. The ligand 4-(trifluoromethyl)-6-methylpyrimidine-2-thiol (PymSH) was synthesized according to a literature procedure.[2] It was then deprotonated with pure NaH in THF to yield Na(PymS) in quantitative yield.[3] The S,N bidentate ligand is abbreviated with PymS for simplicity, although this label was previously attributed to the unsubstituted pyrimidine-2-thiolate.

Carbonyl Complexes. Complex **1** is infinitely stable at ambient conditions in the solid state and solution. Compound **2-Cl** must be stored and handled under a nitrogen atmosphere.

$[W(CO)(C_2H_2)(PymS)_2]$ (1): Na(PymS) (1.36 g, 6.30 mmol) was added portion-wise to a stirred solution of $[WBr_2(CO)_3(NCMe)_2$ (1.53 g, 3.00 mmol) in 30 mL of CH_2Cl_2. After 40 min, the resulting suspension was purged with acetylene for 20 min and then stirred under an acetylene atmosphere for 2 h. Thereafter, all volatiles were removed in vacuo. The resulting brown solid was suspended in 100 mL of CH_2Cl_2, and this suspension was then filtrated through a pad of Celite/silica gel 1:1. The volume of the filtrate was reduced to 50 mL, whereupon 25 mL of heptane was added. After slow evaporation to approx. 20 mL, brown crystals were isolated by filtration, washed with 10 mL of pentane and eventually dried in vacuo to give pure $W(CO)(C_2H_2)(PymS)_2$ (1.35 g, 72%). ^{1}H NMR (CD_2Cl_2, 300 MHz, major isomer): δ 14.00 (s, 1H, C≡CH), 12.65 (s, 1H, C≡CH), 7.35 (s, 1H, pymH), 6.97 (s, 1H, pymH), 2.13 (s, 3H, CH_3), 1.39 (s, 3H, CH_3) ppm. $^{13}C\{^{1}H\}$ NMR (CD_2Cl_2, 75 MHz, major isomer): δ 229.32 (CO), 207.87 (C≡C), 204.06 (C≡C), 186.91 (CS), 177.50 (CS), 169.66 (CCH_3), 165.94 (CCH_3), 156.72 (q, $^{2}J_{CF}$ = 36.47 Hz, CCF_3), 154.62 (q, $^{2}J_{CF}$ = 36.3 Hz, CCF_3), 120.73 (q, $^{1}J_{CF}$ = 275.7 Hz, CF_3), 120.41 (q, $^{1}J_{CF}$ = 275.4 Hz, CF_3), 113.85 (q, $^{3}J_{CF}$ = 2.7 Hz, pymCH), 111.66 (q, $^{3}J_{CF}$ = 2.8 Hz, pymCH), 26.99 (CH_3), 23.13 (CH_3) ppm. ^{19}F NMR (CD_2Cl_2, 282 MHz, major isomer): δ –70.72 (CF_3), –70.83 (CF_3) ppm. IR (cm^{-1}): 3076 (w), 1952 (m, C≡O), 1935 (s, C≡O), 1897 (w, C≡O), 1593 (w), 1581 (m), 1545 (m), 1531 (m), 1440 (m), 1386 (s), 1365 (m), 1273 (s), 1230 (s), 1193 (m), 1161 (m), 1115 (s), 998 (m), 928 (m), 858 (m), 835 (m), 728 (m), 711 (s). EI-MS (70 eV) *m/z*: M^+ 624.0, $[M – CO]^+$ 596.0, $[M – CO – C_2H_2]^+$ 570.0. Anal. Calcd. For $C_{15}H_{10}N_4OS_2F_6W$: C, 28.86; H, 1.61; N, 8.98. Found: C, 28.88; H, 1.51; N, 9.06.

$[W(CO)(CCH_2PMe_3)(PMe_3)_2(PymS)]Cl$ (2-Cl): A 25 mL Schlenk flask was charged with $[W(CO)(C_2H_2)(PymS)_2]$ (156 mg, 0.25 mmol), PMe_3 (93 μL, 0.90 mmol), and 6 mL of CH_2Cl_2. The resulting black suspension was stirred for 3 h before all volatiles were removed in vacuo. Then, 6 mL of MeCN and 5 mL of toluene were added to the dark magenta solid to give a deep pink solution which was reduced in vacuo to approx. 6 mL. The saturated solution was then left at –25 °C overnight. The resulting red crystals were isolated by filtration, washed with Et_2O (2x 2 mL) and pentane (2x 5 mL), and eventually dried in vacuo to yield pure $[W(CO)(CCH_2PMe_3)(PMe_3)_2(PymS)]Cl$ (106 mg, 56%). ^{1}H NMR (CD_3CN, 300 MHz): δ 7.29 (s, 1H, pymH), 3.66 (dt, J = 19.6, 4.7 Hz, 2H, CH_2), 2.43 (s, 3H, CH_3), 1.94 (d, $^{2}J_{PH}$ = 14.5 Hz, 9H, PCH_3), 1.41 (t, 18 H, $WPCH_3$) ppm. $^{13}C\{^{1}H\}$ NMR (CD_3CN, 176.15 MHz): δ 255.33 (m, W≡C), 247.80 (q(dq), J = 165.8, 4.4 Hz, CO), 183.40 (q, CS), 168.34 (s, CCH_3), 153.55 (q, $^{2}J_{CF}$ = 35.2

Hz, CCF_3), 121.77 (q, $^1J_{CF}$ = 274.4 Hz, CF_3), 111.36 (s, pymCH), 46.84 (d(dd), J = 49.8, 45.5 Hz, CH_2), 25.20 (s, CH_3), 18.25 (t, $WPCH_3$), 8.95 (d, $^1J_{CP}$ = 54.6 Hz, PCH_3) ppm. $^{31}P\{^1H\}$ NMR (CD_3CN, 121 MHz): δ 21.30 (t, $^4J_{PP}$ = 5.6 Hz, $CPMe_3$), –19.15 (d(dd), J = 277.9, 4.4 Hz, $WPMe_3$) ppm. IR (cm^{-1}): 3028 (vw), 2969 (w), 2905 (w), 2788 (vw), 2780 (vw), 2719 (vw), 1874 (s, C≡O), 1573 (w), 1529 (w), 1495 (vw), 1421 (m), 1388 (m), 1367 (m), 1297 (m), 1286 (m), 1270 (m), 1232 (m), 1194 (m), 1173 (m), 1132 (m), 1117 (m), 969 (s), 940 (s), 888 (m), 850 (m), 779 (w), 760 (w), 726(m), 712 (m), 697 (w), 668 (m), 578 (w), 559 (w), 462 (m). Anal. Calcd. For $C_{18}H_{33}N_2OF_3P_3SClW \cdot 0.4C_7H_8$: C, 34.15; H, 4.99; N, 3.83. Found: C, 33.83; H, 4.62; N, 3.93.

[W(CO)(CCH$_2$PMe$_2$Ph)(PMe$_2$Ph)$_2$(PymS)]Cl: A 25 mL Schlenk flask was charged with [W(CO)(C_2H_2)(PymS)$_2$] (100 mg, 0.16 mmol), PMe_2Ph (82 µL, 0.58 mmol), and 10 mL of CH_2Cl_2. The resulting brown suspension was stirred for 2 h before all volatiles were removed in vacuo. The residue was dissolved in CH_2Cl_2, filtrated through celite and the filtrate was overlayed with the eqaul volume of PhMe. Slow solvent removal led to formation of red crystaline solid, which was isolated by filtration, washed with pentane and dried in vauceo to yield pure [W(CO)(CCH$_2$PMe$_2$Ph)(PMe$_2$Ph)$_2$(PymS)]Cl (97 mg, 67 %). MW: 880.95 g/mol.

^1H NMR (CD_2Cl_2, 300 MHz): δ 8.06 – 7.92 (m, 2H, PhH), 7.82 – 7.65 (m, 3H, PhH), 7.32 – 7.06 (m, 10H, PhH), 4.41 (dt, J = 19.5, 4.6 Hz, 2H, CH_2), 2.28 (d, $^2J_{PH}$ = 14.0 Hz, 6H, $CPMe_2$), 1.93 (s, 3H, CH_3), 1.78 – 1.72 (m, 12H, $WPMe_2$) ppm. $^{31}P\{^1H\}$ NMR (CD_2Cl_2, 121 MHz): δ 16.67 (t, $^4J_{PP}$ = 5.7 Hz, $CPMe_2Ph$), -5.06 (d, J = 6.1 Hz, $WPMe_2Ph$) ppm. ^{19}F NMR (CD_2Cl_2, 282 MHz) δ -70.47 ppm. IR (cm^{-1}): 3485 (w), 3407 (w), 2972 (w), 2908 (w), 1872 (s, C≡O), 1271 (m), 1133 (m), 1112 (m), 942 (m), 905 (m), 743 (m), 695 (m), 490 (m), 479 (m).

Oxo Complexes. Complex **3** is infinitely stable under ambient conditions in the solid state. Solutions should be handled under a N_2 atmosphere. Complex **3c** is temperature-sensitive and slowly decomposes if not stored at –35 °C in a glovebox. Compound **4-Cl** decomposes within 30 min at ambient conditions and should be stored in a glovebox, preferably at –35 °C.

[WO(C_2H_2)(PymS)$_2$] (3): A solution of [W(CO)(C_2H_2)(PymS)$_2$] (624 mg, 1.00 mmol) and pyridine *N*-oxide (105 mg, 1.15 mmol) in 18 mL of CH_2Cl_2 was stirred for 1 h. After evaporation to dryness, the yellow solid was suspended in 30 mL CH_2Cl_2, and the resulting suspension was filtrated through Celite. The volume of the yellow filtrate was reduced to 20 mL before 15 mL of

heptane was added. Further evaporation to 10 mL gave light yellow crystals, which were isolated by filtration, washed with Et_2O (4x 2 mL) and pentane (2x 2 mL), and eventually dried in vacuo to yield $WO(C_2H_2)(PymS)_2$ (586 mg, 96%). ^1H NMR (CD_2Cl_2, 300 MHz, major isomer): δ 11.39 (s, 1H, C≡CH), 11.02 (s, 1H, C≡CH), 7.42 (s, 1H, pymH), 7.39 (s, 1H, pymH), 2.75 (s, 3H, CH_3), 2.26 (s, 3H, CH_3) ppm. $^{13}C\{^1H\}$ NMR (CD_2Cl_2, 75 MHz, major isomer): δ 183.76 (CS), 180.80 (CS), 170.74 (CCH_3), 167.13 (CCH_3), 157.80–156.39 (m, CCF_3, 2C), 157.12 (C≡C), 156.60 (C≡C), 120.61 (q, $^1J_{CF}$ = 276.0 Hz, CF_3), 120.42 (q, $^1J_{CF}$ = 275.9 Hz, CF_3), 114.90 (q, $^3J_{CF}$ = 2.0 Hz, pymCH), 112.46 (q, $^3J_{CF}$ = 1.6 Hz, pymCH), 25.64 (CH_3), 22.24 (CH_3) ppm. ^{19}F NMR (CD_2Cl_2, 282 MHz, major isomer): δ –70.87 (CF_3), –71.00 (CF_3) ppm. IR (cm^{-1}): 3090 (w), 3074 (w), 1586 (m), 1544 (m), 1415 (m), 1389 (m), 1277 (m), 1230 (m), 1147 (s), 1113 (s), 944 (s), 856 (m), 843 (m), 712 (s), 561 (m). EI-MS (70 eV) *m/z*: M^+ 612.0, $[M - C_2H_2]^+$ 586.1. Anal. Calcd. For $C_{14}H_{10}N_4OF_6S_2W$: C, 27.47; H, 1.65; N, 9.15. Found: C, 27.63; H, 1.44; N, 9.41.

$[WO(CHCHPMe_3)(PMe_3)(PymS)_2]$ (3c): A 10 mL Schlenk flask was charged with $[WO(C_2H_2)(PymS)_2]$ (92 mg, 0.15 mmol), PMe_3 (33 µL, 0.32 mmol), and 3 mL of CH_2Cl_2. The resulting deep blue solution was stirred for 15–60 min before it was evaporated to dryness to give pure $[WO(CHCHPMe_3)(PMe_3)(PymS)_2]$ in quantitative yield. The deep blue solid was immediately transferred to a glovebox and stored at –35 °C. ^1H NMR (CD_3CN, 300 MHz): δ 11.23 (ddd(dddd), *J* = 37.8, 18.5, 1.8 Hz, 1H, WCH), 7.02 (s, 1H, pymH), 6.96 (s, 1H, pymH), 4.91 (ddd(dddd), *J* = 38.3, 18.5, 1.9 Hz, 1H, PCH), 2.66 (s, 3H, CH_3), 2.27 (s. 3H, CH_3), 1.69 (d, $^2J_{PH}$ = 13.6 Hz, 9H, PCH_3), 1.37 (d, $^2J_{PH}$ = 9.1 Hz, 9H, $WPCH_3$) ppm. $^{13}C\{^1H\}$ NMR (CD_2Cl_2, 75 MHz): δ 225.02 (dd, *J* = 9.9, 5.4 Hz, WCH), 185.72 (CS), 175.25 (CS), 169.53 (CCH_3), 165.07 (CCH_3), 154.63 (q, $^2J_{CF}$ = 34.3 Hz, CCF_3), 151.71 (q, $^2J_{CF}$ = 34.7 Hz, CCF_3), 121.98 (q, $^1J_{CF}$ = 274.7 Hz, CF_3), 121.44 (q, $^1J_{CF}$ = 273.7 Hz, CF_3), 111.23 (q, $^3J_{CF}$ = 2.6 Hz, pymCH), 108.58 (q, $^3J_{CF}$ = 2.6 Hz, pymCH), 97.88 (dd, *J* = 80.9, 2.6 Hz, CHP), 25.21 (CH_3), 24.34 (CH_3), 15.33 (d, $^1J_{CP}$ = 30.3 Hz, $WPCH_3$), 12.72 (d, $^1J_{CP}$ = 58.0 Hz, PCH_3) ppm. $^{31}P\{^1H\}$ NMR (CD_3CN, 121 MHz): δ 3.86 (d(dd), *J* = 45.2, 4.9 Hz, $CPMe_3$), –23.52 (d(dd), *J* = 375.8, 4.5 Hz, $WPMe_3$) ppm. IR (cm^{-1}): 2976 (w), 2908 (w), 1578 (m), 1542 (m), 1429 (m), 1389 (m), 1363 (m), 1276 (m), 1256 (m), 1222 (m), 1191 (m), 1168 (m), 1132 (s), 1107 (s), 966 (s), 937 (s, W=O), 839 (m), 730 (m), 708 (s), 582 (m), 546 (m), 478 (m). Anal. Calcd. For $C_{20}H_{28}N_4OF_6P_2S_2W$: C, 31.43; H, 3.69; N, 7.33. Found: C, 31.21; H, 3.30; N, 7.42.

[WO(CHCHPMe3)(PMe3)2(PymS)]Cl (4-Cl): A scintillation vial was charged with $WO(C_2H_2)(PymS)_2$ (92 mg, 0.15 mmol), PMe_3 (93 µL, 0.90 mmol), and 4 mL of CH_2Cl_2. The resulting deep blue solution was stirred for 8 h before 2 mL of heptane was added to the now black solution which was then left open at –35 °C for 4 weeks. The resulting black crystals were isolated by filtration, washed with Et_2O (2x 1 mL) and pentane (2x 2 mL), and eventually dried in vacuo to yield $[WO(CHCHPMe_3)(PMe_3)_2(PymS)]Cl$ (72 mg, 70%). ^{1}H NMR (CD_2Cl_2, 300 MHz): δ 11.24 (dd, J = 37.7, 18.6 Hz, 1H, WCH), 7.28 (s, 1H, pymH), 4.65 (dd, J = 35.9, 18.7 Hz, 1H, PCH), 2.73 (s, 3H, CH_3), 2.04 (d, $^2J_{PH}$ = 13.6 Hz, 9H, PCH_3), 1.44 (t, $^2J_{PH}$ = 4.3 Hz, 18H, $WPCH_3$) ppm. ^{13}C{^{1}H} NMR (CD_2Cl_2, 176.15 MHz): δ 219.62 (bs, 1H, WCH), 172.77 (s, CS), 165.52 (s, CCH_3), 153.22 (q, $^2J_{CF}$ = 35.7 Hz, CCF_3), 120.51 (q, $^1J_{CF}$ = 274.8 Hz, CF_3), 110.68 (q, $^3J_{CF}$ = 3.2 Hz, pymCH), 102.08 (d, $^1J_{CP}$ = 79.6 Hz, CHP), 25.08 (s, CH_3), 14.37 (t, $^2J_{CP}$ = 15.3 Hz, $WPCH_3$), 12.64 (d, $^2J_{CP}$ = 57.5 Hz, PCH_3) ppm. ^{31}P{^{1}H} NMR (CD_2Cl_2, 121 MHz): δ 7.28 (bs, $CPMe_3$), –23.19 (bs, $WPMe_3$) ppm. IR (cm^{-1}): 2971 (w), 2900 (w), 1571 (w), 1516 (vw), 1452 (w), 1432 (m), 1422 (m), 1411 (m), 1391 (m), 1371 (m), 1278 (m), 1230 (m), 1189 (m), 1161 (m), 1143 (m), 1115 (m), 982 (s), 968 (m), 942 (s, W=O), 898 (m), 846 (m), 812 (w), 777 (w), 759 (w), 737 (m), 712 (m), 668 (m), 558 (m). Anal. Calcd. For $C_{17}H_{33}N_2OF_3P_3SClW$: C, 29.91; H, 4.87; N, 4.10. Found: C, 30.27; H, 4.61; 4.27.

3 DFT Calculations

All DFT calculations were performed with TURBOMOLE 7.4.1.[4–6] Geometries were optimized employing the PBE[7] functional together with the D3 dispersion correction using Becke-Johnson damping[8,9] and the dhf-SVP basis set.[10] To account for relativistic effects occurring for the central tungsten atom, the corresponding dhf-ecp (effective core potential) was utilized.[11] For speeding up the calculations, the resolution of identity (RI) approximation was utilized.[12–15] Transition states were located by using TURBOMOLE's woelfling-program,[16] followed by subsequent geometry optimization. Analytical normal modes were determined using TURBOMOLE's aoforce-program for confirmation of the stationary points and transition state search. After scaling of the frequencies,[17] the rigid-rotor-harmonic-oscillator (RRHO) approximation was used to calculate zero-point vibrational energies and thermal properties at room temperature (298 K).

To model solvent effects, geometries were reoptimized at the RI-PBE-D3BJ/dhf2-SVP level employing the COSMO-solvation model for dichloromethane (ε_r = 8.930).[18,19]

Single points of the transition states and minima were calculated with RI-PBE-D3BJ/dhf-TZVPP[10] and RIJK-B3LYP[20–22]-D3BJ/dhf2-TZVPP (with and without computing solvation effects), making use of the RIJK approximation for the B3LYP calculations.[21,23,24] Zero-point energies and thermal corrections for the COSMO-reoptimized structures and the single point calculations were taken from the RI-PBE-D3BJ/dhf-SVP gas phase calculations. The data reported in the main text and the remainder of this SI are the B3LYP-D3BJ/dhf2-TZVPP+COSMO data except otherwise noted. NBO-charges and Wiberg bond indices were calculated with the NBO implementation of TURBOMOLE[25] at the RIJK-B3LYP-D3BJ/dhf-TZVPP@RI-PBE-D3BJ/dhf-SVP level. NMR shifts were calculated using TURBOMOLE's mpshift program[26] at the RI-PBE-D3BJ/dhf-SVP level. This method has additionally been verified to yield consistent results with calculations performed at both the RI-PBE-D3BJ/dhf-TZVPP and RIJK-B3LYP-D3BJ/dhf2-TZVPP level.

4 Mechanistic Elucidation

Formation of the Carbyne Complex 2-Cl (Carbonyl Mechanism). Considering all our experimental and computational findings, we propose the reaction mechanism shown in Scheme 1. After the initial coordination of two PMe_3 molecules to the W center in **1**, the electron-rich d^4 W(II) carbonyl compound **1b** releases one ancillary ligand, thereby forming **1b-PymS**. This cationic acetylene intermediate is sufficiently activated for nucleophilic attack by the anionic thiolate ligand giving the metallacyclopropane compound **1b''**. The attack of the third equivalent of PMe_3 leads to dissociation of the C-bound thiolate yielding the η^1-vinyl species **1c-η^1**. The latter rearranges *via* a vinylidene transition state **TS10** with the thiolate ligand acting as a α-H acceptor. Due to π-backdonation from the W center to the vinylidene ligand, the negative charge now accumulates on the β-C of **TS10**, which deprotonates PymSH, leading to a thermodynamically stable W(IV) carbyne ion pair **2-PymS**. The final product **2-Cl** is isolated due to the reaction of the anionic ligand with CH_2Cl_2. The overall reaction diagram is presented in Diagram S1.

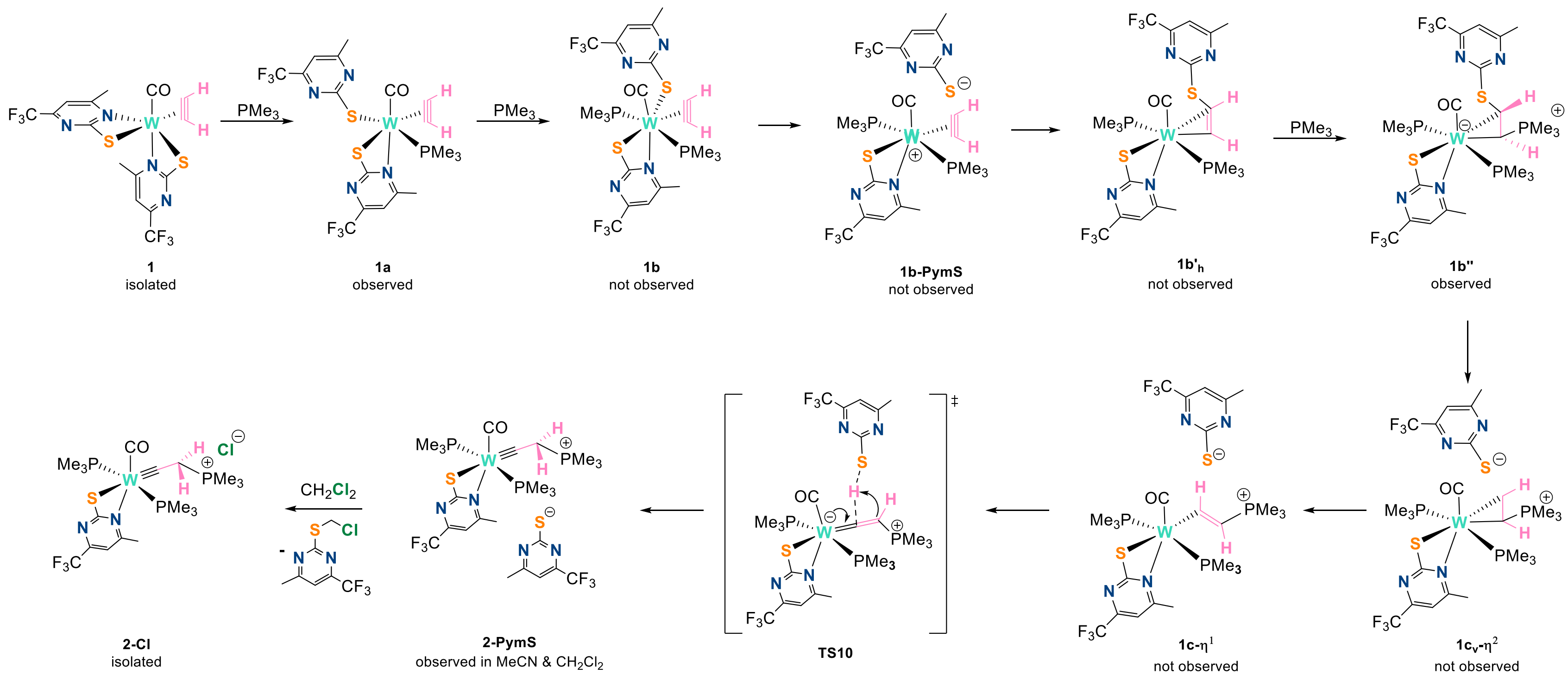

Scheme 1. Suggested reaction mechanism for the reaction of **1** to a carbyne complex **2-Cl**

Experimental and Computational Evidence for the Cabonyl Mechanism. To investigate the first step of the overall reaction, **1.3 equiv of PMe_3** were added to a precooled CD_2Cl_2 solution of **1** which was kept below 0 °C until ^1H and $^{31}P\{^1H\}$ NMR spectra were recorded. The ^1H NMR spectrum showed almost full conversion of the starting materials and the formation of one major species with two resonances for the coordinated C_2H_2 at 12.50 and 12.14 ppm as well as a doublet at 1.08 ppm resulting from a W-coordinated PMe_3 molecule (Figure S18). The corresponding $^{31}P\{^1H\}$ NMR spectrum showed a singlet at –27.43 ppm with ^{183}W satellites (Figure S19, $J_{W–P}$ = 236.2 Hz). Spectral data demonstrate almost full conversion of the starting materials and the formation of one major species [W(CO)(C_2H_2)(PMe_3)(*S*-PymS)(PymS)] (**1a**), which is also observed in CD_3CN, albeit less selectively. This provides a strong indication that coordination of PMe_3 to the tungsten center of **1** constitutes the first step of the reaction mechanism. Since a seven-coordinate tungsten complex would be sterically extremely crowded, we presume that **1a** contains both a bidentate and a monodentate PymS ligand, with the latter coordinated only *via* the thiolate moiety. Indeed, our DFT calculations corroborate this assumption, which predict formation of **1a** to occur by an associative substitution mechanism *via* hepta-coordinate **1a'** (Diagram S1). Surprisingly, attack at the C_2H_2 ligand is kinetically disfavored by 5.7 kJ/mol only ($\Delta\Delta G^{TS}$ = +66.2 *vs.* +71.9 kJ/mol). A strong hint that the nitrogen of one PymS ligand decoordinates to end up with a six-coordinate species is provided by the previously published reaction of [W(CO)(C_2Ph_2)(6-MePyS)$_2$] with five equiv of PMe_3 to selectively give [W(CO)(C_2Ph_2)(PMe_3)(6-MePyS-*S*,*N*)(6-MePyS-*S*)].[27] In both solvents, acetonitrile and dichloromethane, **1a** decomposes within 1 h at room temperature. Overall, we found in our group that seven-coordinate tungsten centers coordinated by this type of ligands are indeed rare, with only one such species reported,[28] in contrast to six-coordinate tungsten centers.[3,28–30]

While adding **2 equiv of PMe_3** to solutions of **1** did not give conclusive spectral information, DFT calculations predict attack of the second molecule of PMe_3 to also occur at the tungsten center of **1a**, yielding high energy intermediate [W(CO)(C_2H_2)(PMe_3)$_2$(PymS-*S*,*N*)(PymS-*S*)] (**1b**). Again, the potential attack of phosphine at the coordinated acetylene in **1a** is kinetically disfavored by 9.6 kJ/mol ($\Delta\Delta G^{TS}$ = +75.2 *vs.* +84.8 kJ/mol).

To obtain experimental insight into the next steps of the reaction, we increased the amount of **PMe_3** to **3 equiv**, upon which the ^1H NMR peaks of coordinated C_2H_2 disappeared and a new

major species [W(CO)(PymS-*S*-CHCHPMe$_3$)(PMe$_3$)$_2$(PymS-*S,N*)] (**1b''**) was formed (Figure S20-S22) which could only transiently be detected at 0 °C. The NMR data suggest that the compound contains two chemically inequivalent tungsten coordinated PMe$_3$ molecules as well as a carbon bound PMe$_3$, while the two PymS ligands also remain attached. The intermediate displays two intriguing signals at 3.20 and 0.75 ppm (2x dddd) integrating to one proton each, the coupling of which was confirmed by a COSY (Figures S23).

DFT analysis revealed the order of elementary steps between **1b** and **1b''**. Decoordination of the monodentate anionic ligand PymS from **1b** leads to the barrierless formation of the cationic complex **1b-PymS**. The attack of the free anionic PymS ligand at the coordinated acetylene in **1b-PymS** is favored by 68.5 kJ/mol ($\Delta\Delta G^{TS}$ = +16.6 kJ/mol for PymS$^-$ attack *vs.* 85.1 kJ/mol for PMe$_3$ attack). Such an attack leads to the formation of the high energy intermediate [W(CO)(PymS-*S*-CH=CH)(PMe$_3$)$_2$(PymS-*S,N*)] (**1b'**$_v$), which further reacts with PMe$_3$ to the low energy intermediate **1b''** observed in NMR experiments. Structure optimization showed that **1b''** features the tungstocyclopropane moiety in a horizontally aligned fashion (perpendicular to the W-CO bond) with the two hydrogen atoms located *trans* to each other.

Aside from **1b''**, a considerable amount of [W(CO)(C≡CH$_2$PMe$_3$)(PMe$_3$)$_2$(PymS)]$^+$, the cation of the final product **2-Cl**, was detected in CD_2Cl_2 (Figure S20). Here, the formation of PymSCD$_2$Cl was observed in CD_2Cl_2, while this species cannot be generated in CD_3CN. Thus, we conclude that the formation of **2-Cl** proceeds *via* the analogous carbyne species [W(CO)(CCH$_2$PMe$_3$)(PMe$_3$)$_2$(PymS)][PymS] (**2-PymS**). This is supported by NMR data of the deprotonated ligand in both solvents (Tables S3) and explains why the carbyne cation is also observed in CD_3CN solutions. When the CD_2Cl_2 reaction mixture was left at room temperature for 45 minutes before the next measurement, PymS fully converted to PymSCD$_2$Cl. To verify that PymS can react with CH_2Cl_2 without being coordinated to a metal center, a mixture of Na(PymS), Bu$_4$NCl, and CH_2Cl_2 was stirred for 24 h. Addition of Bu$_4$NCl was necessary as the sodium salt alone is almost insoluble in CH_2Cl_2, and, hence, reacts only very slowly with the solvent. Evaporation to dryness and subsequent analysis by ^{1}H NMR spectroscopy confirmed the selective formation of PymSCH$_2$Cl (Figure S24).

The experimental observations are readily explained by the DFT calculations. Following the formation of **1b''**, PymS$^-$ is expelled from the tungstocyclopropane moiety yielding the high-

energy η^2-intermediate **$1c_v$-η^2** which subsequently is converted to its η^1-form **$1c$-η^1**. Again, a horizontally oriented intermediate is also formed but is omitted for simplicity. The hydrogen shift from **$1c$-η^1** to **2-PymS** occurs *via* a vinylidene transition state **TS10** corresponding to a surprisingly low energetic barrier of only +31.0 kJ/mol which is facilitated by the basic nature of the decoordinated PymS ligand anion. The latter deprotonates the α-carbon and transfers the proton onto the β-carbon, acting as a hydrogen shuttle. Indeed, a comparable transition state where the PymS ligand anion is not in proximity to the hydrogen being transferred boosts an exceedingly large energetic barrier of +127.5 kJ/mol. Notably, attack of PMe_3 on the C_2H_2 ligand of **1b-PymS** would directly lead to the formation of **$1c_v$-η^2**. While this route is overall kinetically disfavored ($\Delta\Delta G^{TS}$ = +85.1 kJ/mol), it cannot totally be ruled out, leading to an additional potential route toward **1b''** (corresponding to the backreaction of the preferred pathway from **$1c_v$-η^2**; for a detailed discussion, see the SI). Therefore, formation of the cationic complex **1b-PymS** activates the acetylene for a nucleophilic attack. Finally, the $PymS^-$ counterion reacts with CH_2Cl_2 yielding the final ion pair **2-Cl**. The slow rate of this transformation is easily explained by the associated comparably large energetic barrier ($\Delta\Delta G^{TS}$ = +85.6 kJ/mol).

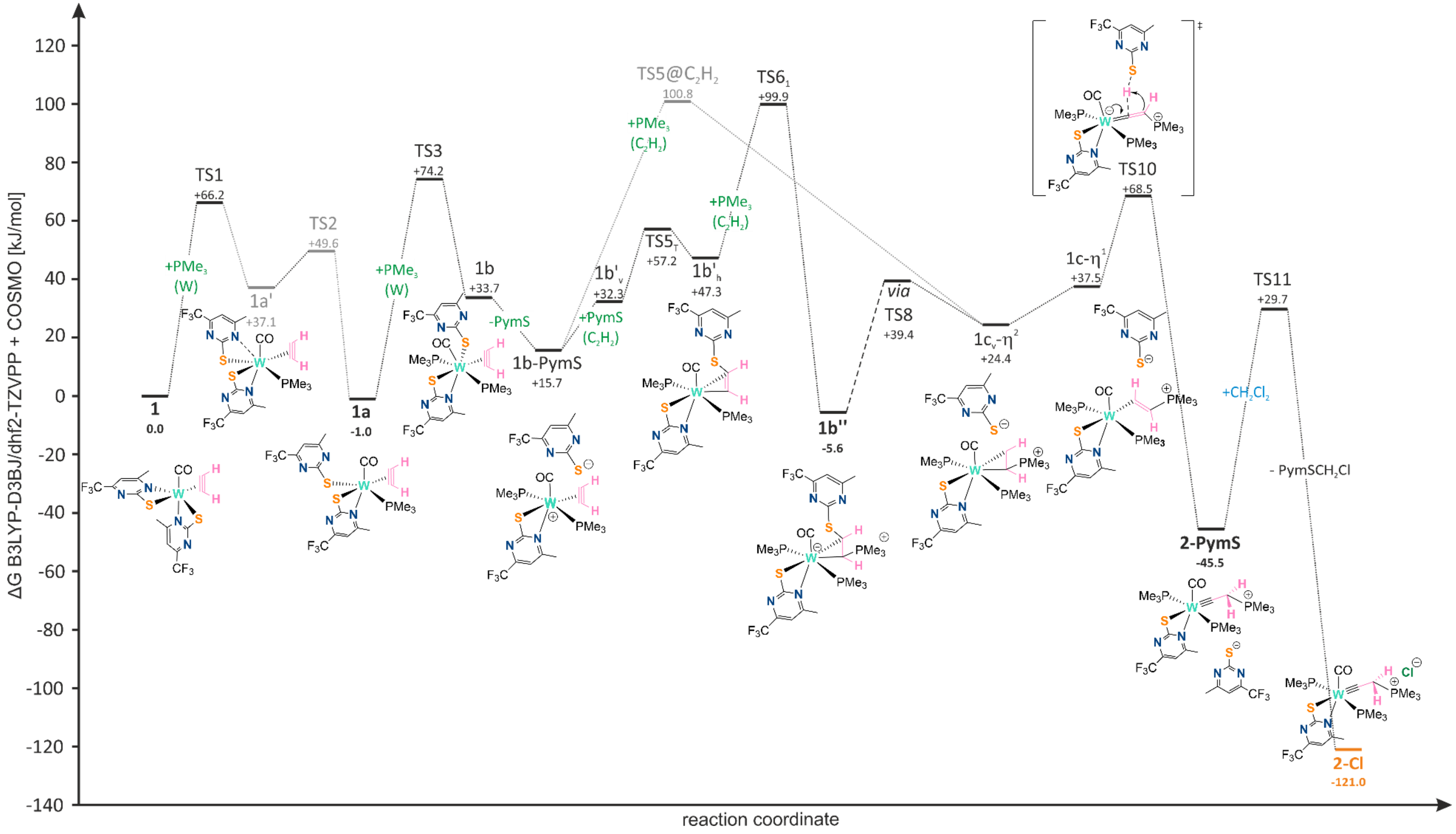

Diagram S1. Suggested energy profile for carbyne species formation starting from compound **1** and PMe_3

.

Formation of the η[1]-Vinyl Complex 4-Cl (Oxo Mechanism). Our experimental and theoretical evidence (*vide infra*) suggest the reaction mechanism as shown in Scheme 2. Similar to the above described mechanism, the transformation starts with the initial coordination of one PMe_3 molecule to the W center to **3** yielding compound **3a**, in which acetylene is sufficiently activated for the nucleophilic attack by the phosphine leading to the η^1-vinyl complex **3c**. Further reaction with a third equivalent of phosphine leads to the cleavage of the ancillary ligand forming to the carbyne complex **4-PymS** and subsequently the chloride salt **4-Cl**. The formation of the latter occurs due to the reaction of the anionic ligand with dichloromethane, as explained above.

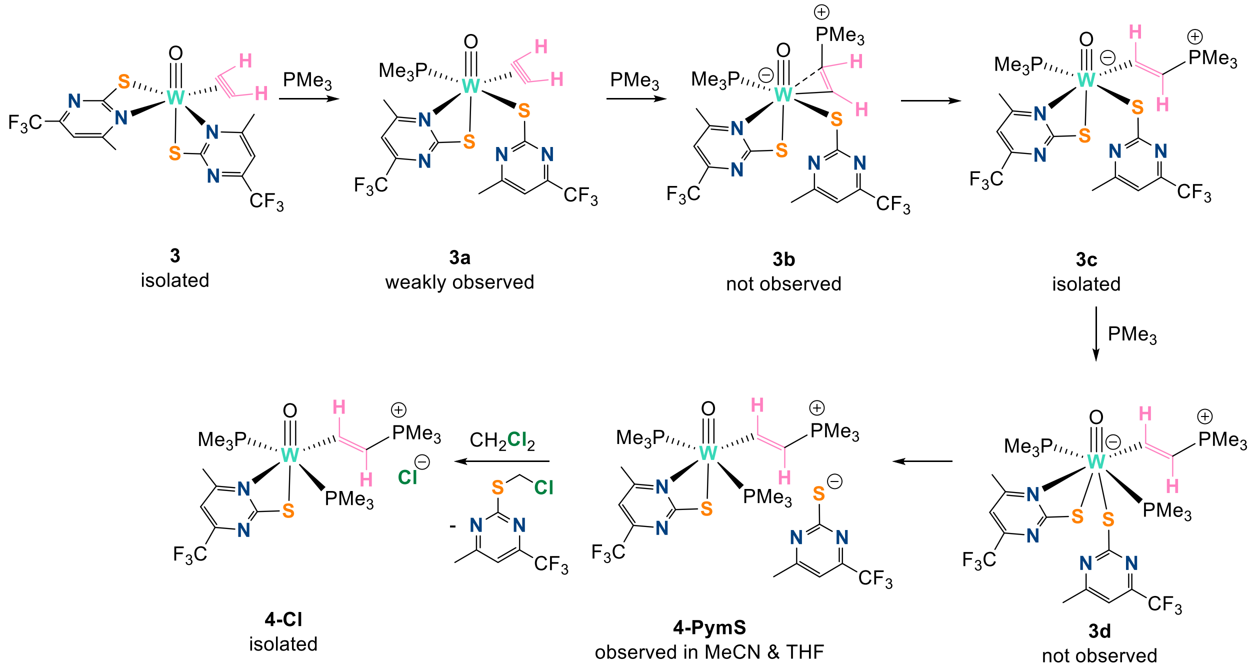

Scheme 2. Suggested reaction mechanism for the reaction of **3** with 3 equiv of PMe_3

Experimental and Computational Evidence for the Oxo Mechanism. NMR spectra of **3** with **1.1 equiv of PMe_3** in CD_2Cl_2 or CD_3CN solutions reveal the presence of $WO(C_2H_2)(PMe_3)(PymS)_2$ (**3a**) (Figure S25), again showing that the first PMe_3 coordinates to the tungsten center. This is further corroborated by the DFT studies, which predict formation of **3a** to occur by an associative substitution mechanism *via* hepta-coordinate **3a'**. Attack at the C_2H_2 ligand of **3** is both thermodynamically and kinetically highly disfavored ($\Delta\Delta G^{TS} = +63.3$ *vs.* +86.0 kJ/mol; $\Delta G = +20.8$ *vs.* +42.4 kJ/mol). The reaction mixture with **1.1 equiv of PMe_3** already contained a small share of the vinyl species $[WO(\eta^1\text{-}CH_\alpha\text{-}CH_\beta PMe_3)(PMe_3)(PymS\text{-}S,N)(PymS\text{-}S)]$ (**3c**,

Figures S25 to S28). After increasing the amount of PMe_3 to **2.2 equiv**, **3c** was formed with high selectivity in various solvents (THF, MeCN, $CHCl_3$ and CH_2Cl_2). The reaction was most selective in CH_2Cl_2, from which we obtained a pure sample directly after solvent evaporation without further work-up. The formation of **3c** is supported by the ^1H NMR spectrum (CD_2Cl_2, Table S2), where the shifts and coupling patterns of the ethenyl protons (11.41 ppm; 2x ddd, flanked with ^{183}W satellites, WCH and 4.96 ppm PCH) are very similar to those of $[IrBr(CO)(CH_3)(\text{-}CH{=}CHPPh_3)_2(PPh_3)]^+$.[31] Furthermore, upon treating starting complex **3** with **3.3 equiv of PMe_3** the final product 4-Cl is selectively formed (Figure S29). However, monitoring by ^1H NMR spectroscopy reveals a minor species in the early phase of the reaction assignable to the intermediate $WO(\eta^2\text{-}CH_\alpha{=}CH_\beta PMe_3)(PMe_3)(PymS)_2$ (**3b**). This is demonstrated by H–P coupling constants greater than those in **3c**, hinting at a shorter C–C distance, and a shift of the H_α and H_β resonances to lower and higher field, respectively, indicating a stronger W–CH_α bond and W–CH_β bond breaking (Figure S29).

Calculations support this course of the reaction, as attack of the second PMe_3 at the tungsten center of **3a** is predicted to yield a thermodynamically extremely unstable product (**3b@W**, $\Delta G = +74.8$ kJ/mol) compared to **3b** ($\Delta G = +31.6$ kJ/mol). Subsequently, **3b** is further transformed into the η^1-vinyl compound **3c**. The actual transformation involves a couple of conformational changes regarding the orientation of the vinyl ligand, which are omitted for clarity (for detailed information, see the SI). Overall, the formation of **3c** is driven by the exergonicity of the overall reaction ($\Delta G = -58.7$ kJ/mol).

Moreover, upon reaction of **3** with **3.3 equiv of PMe_3** in CD_3CN, besides major product **3c**, resonances consistent with those of $[WO(CHCHPMe_3)(PMe_3)_2(PymS)][PymS]$ (**4-PymS**) were observed in the ^1H NMR spectrum after 20 min of reaction which increase with time (Figure S30). This is also supported by the fact that the resonances of the PymS counteranion are well in accordance with NMR data of Na(PymS) (Tables S2 and S3). Furthermore, the reaction of **3** with **3.3 equiv of PMe_3** in CD_2Cl_2 was followed by ^1H NMR spectroscopy at specific time intervals revealing no full conversion of **3c** to **4-Cl** even after 22 h (Figure S29). Computationally, the experimental data for further reactivity of **3c** are readily explained by an associative mechanism. Precisely, a third molecule of PMe_3 coordinates to the tungsten center of **3c** first, forming the short-lived high-energy intermediate **3d** ($\Delta G = +57.5$ kJ/mol) without an additional energetic barrier.

Once formed, **3d** can either undergo loss of PMe_3 to restore **3c** or loss of the thiolate ligand, thereby forming the ion pair **4-PymS**. The relative energies of **3c** and **4-PymS** (ΔG = -58.7 and -38.9 kJ/mol) support the formation of a mixture of both **3c** and **4-PymS** in the reaction vessel. Using CH_2Cl_2 as a solvent, **4-PymS** can subsequently undergo further reaction to the ion pair **4-Cl** and $PymSCH_2Cl$, again driven by the corresponding energy gain of the reaction ($\Delta\Delta G$ = -60.2 kJ/mol *vs.* **3c**). The overall reaction diagram is presented in **Diagram S2**.

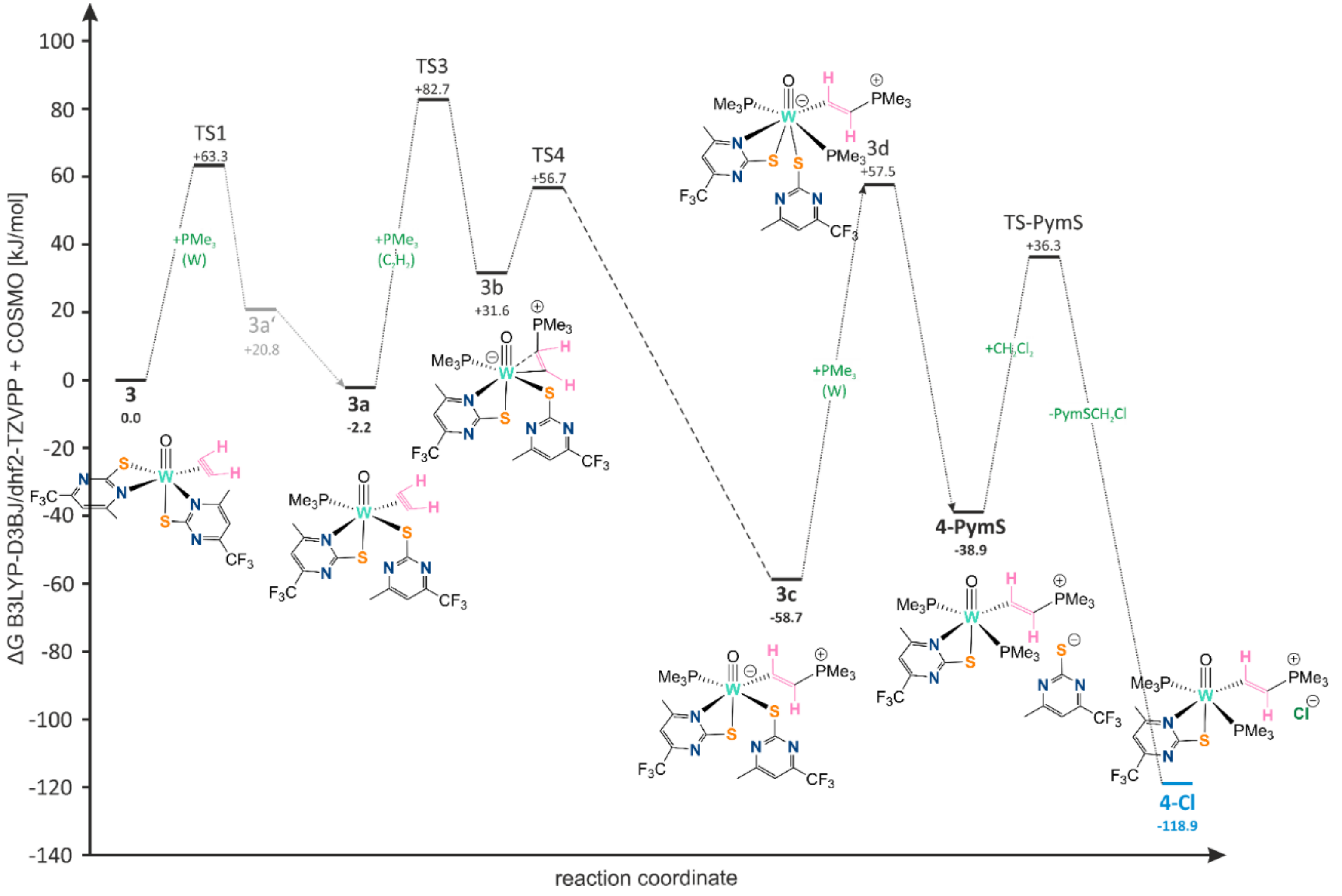

Diagram S2. Energy profile of the possible reaction mechanism for η[1]-vinyl species formation

Alternative possibilities for PMe_3 attack (DFT)

If acetylene ligand is attacked by PMe_3 in the following compounds, the reaction were calculated to be disfavored according to sequences presented below. A detailed description of the calculations can be found in the DFT Supporting Information. @ points the target of the computed nucleophilic attack; "agg" stands for agglomerate formed with PMe_3, prior to transition state. All the coordinates are provided as XYZ files in the accompanying documents. Relative energies are given in kJ/mol.

Attack at the C_2H_2 in compound **1**:

options 1: 1 (0.0) → $agg1_1@C_2H_2$ (+21.4) → $TS1_1@C_2H_2$ (+71.9) → $1a'_1@C_2H_2$ (+9.0) → $1b'_1@C_2H_2$ (+88.0)

Option 2: 1 (0.0) → $agg1_2@C_2H_2$ (+22.3) → $TS1_2@C_2H_2$ (+73.3) → $1a'_2@C_2H_2$ (+15.1) → $1b'_2@C_2H_2$ (+75.4)

Attack at the C_2H_2 in compound **1a**: 1a (-1.0) → $agg3_4@C_2H_2$ (+23.9) → $TS3_4@C_2H_2$ (+83.8) → $1b_4@C_2H_2$ (+22.8)

Attack at the C_2H_2 in compound **1b-PymS** 1b-PymS (+15.7) → $TS5@C_2H_2$ (+100.8) → $1c_v$-η^2 (+24.4) → $1c_{ago}$-η^1(+37.5) → TS10(+68.5) → 2-PymS(-45.5) → TS11(+40.1) → 2-Cl(-121.0)

Attack at the C_2H_2 in compound **3**: 3 (0.0) → $agg1_3@C_2H_2$ (+22.7) → $TS1_3@C_2H_2$ (+86.0) → $3a'_3@C_2H_2$ (+42.4)

Attack at the W center in compound **3a** leads to the high energy species **3b@W** (+74.8).

$TS10_{direct}$: A comparable transition state to TS10 (CO mechanism) where the PymS ligand anion is not in proximity to the hydrogen being transferred boosts an exceedingly large energetic barrier of +127.5 kJ/mol.

5 NMR Spectra of Isolated Compounds

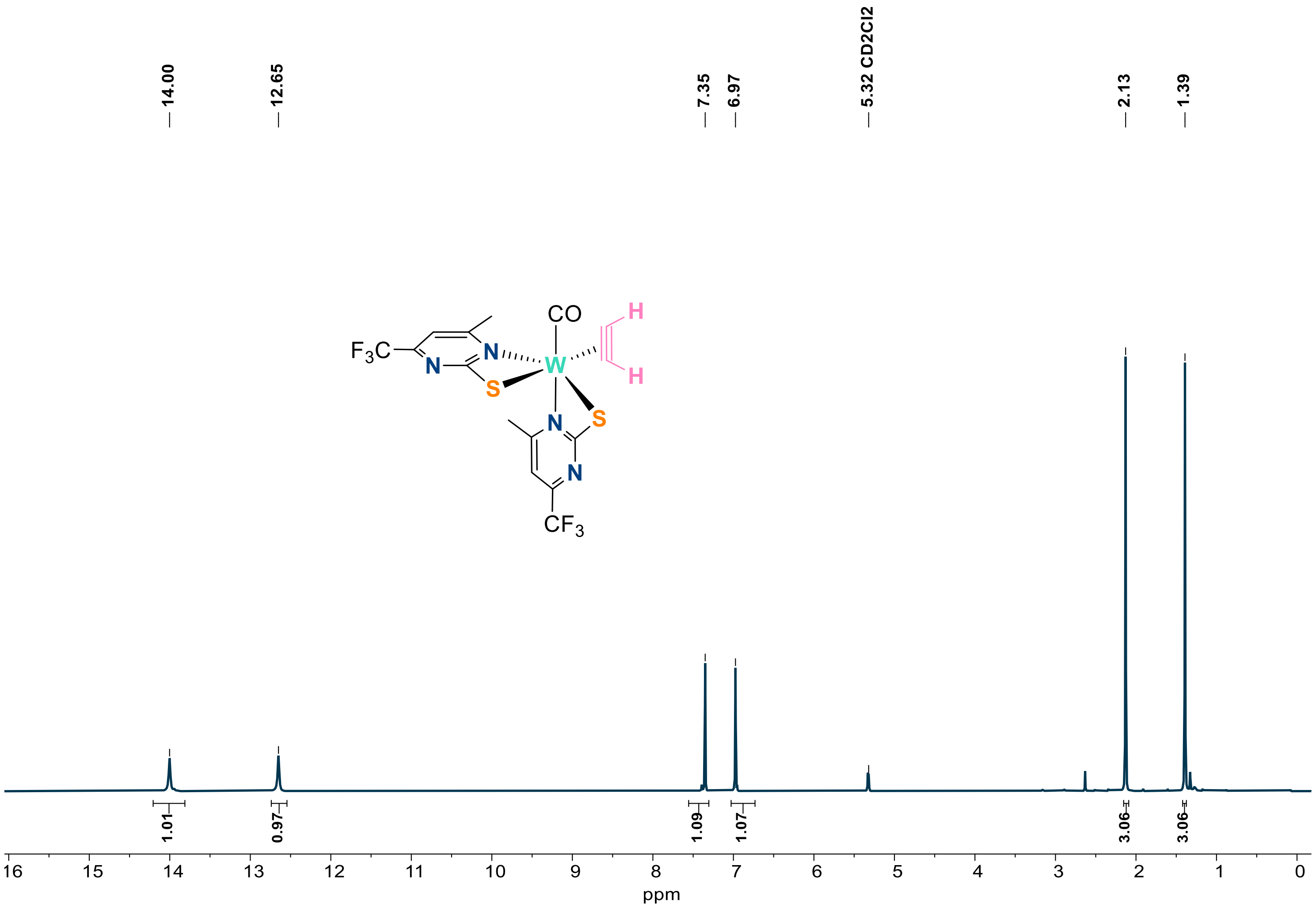

Figure S1. ^{1}H NMR spectrum of **1** in CD_2Cl_2.

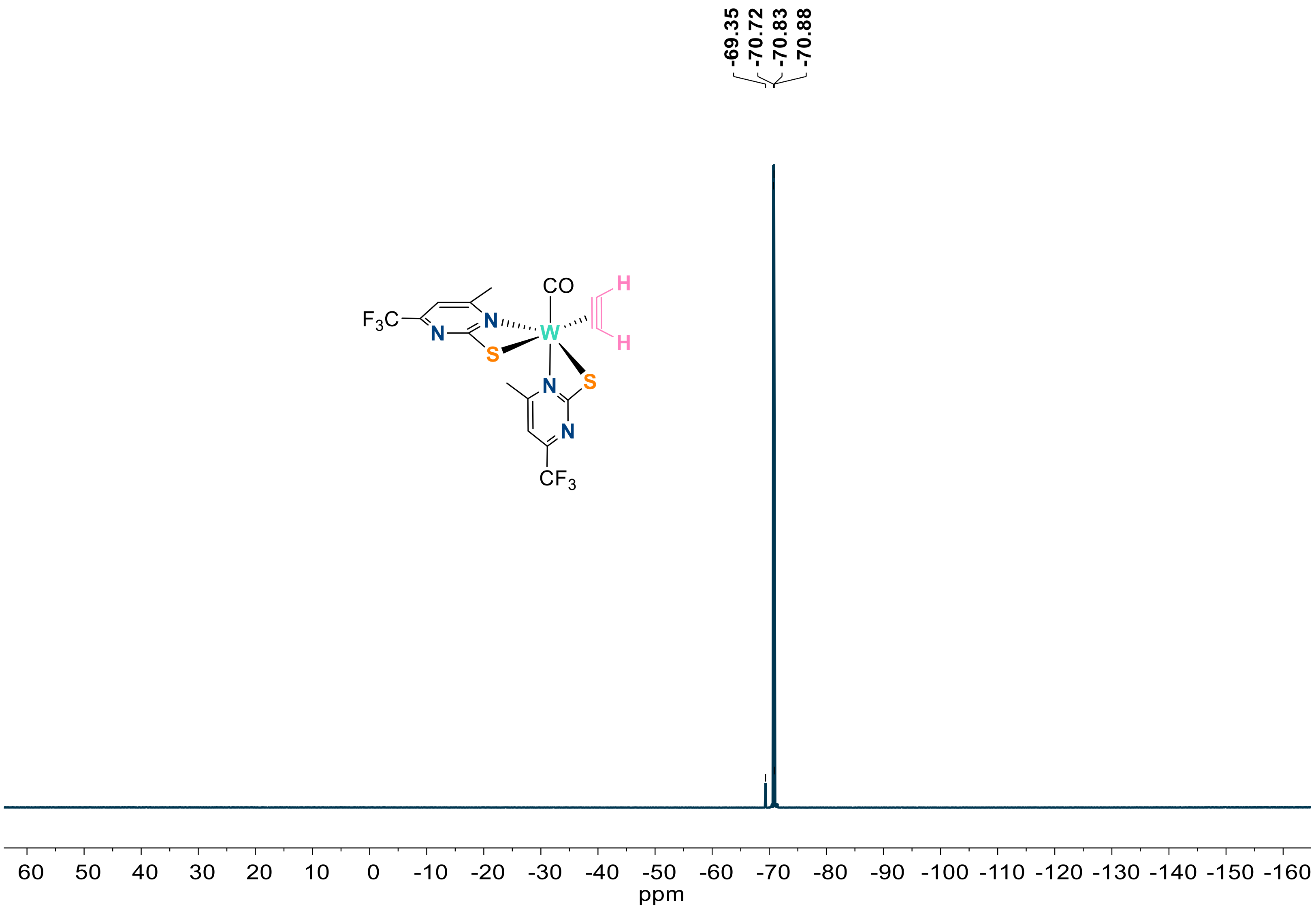

Figure S2. ^{19}F NMR spectrum of **1** in CD_2Cl_2.

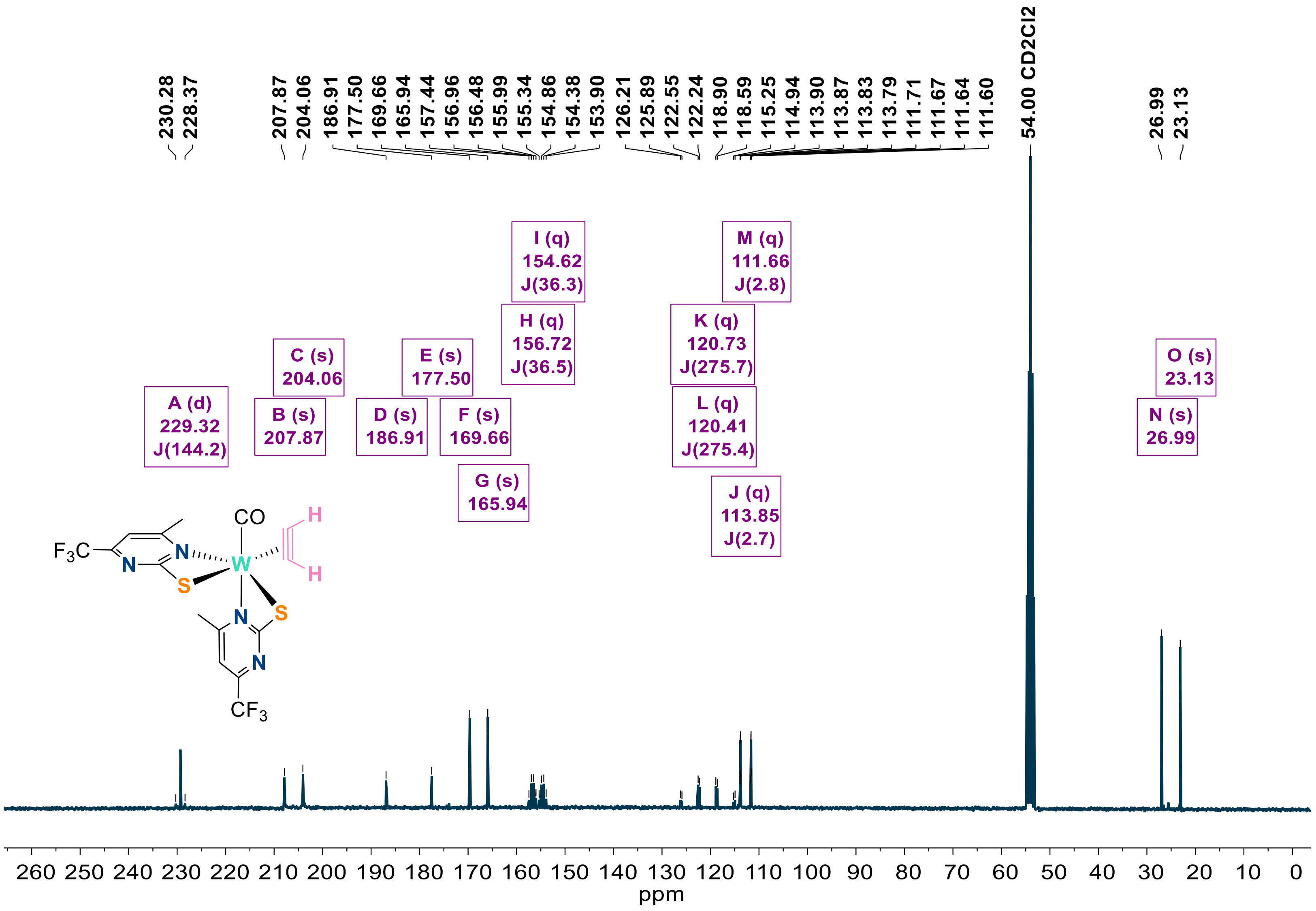

Figure S3. ^{13}C NMR spectrum of **1** in CD_2Cl_2.

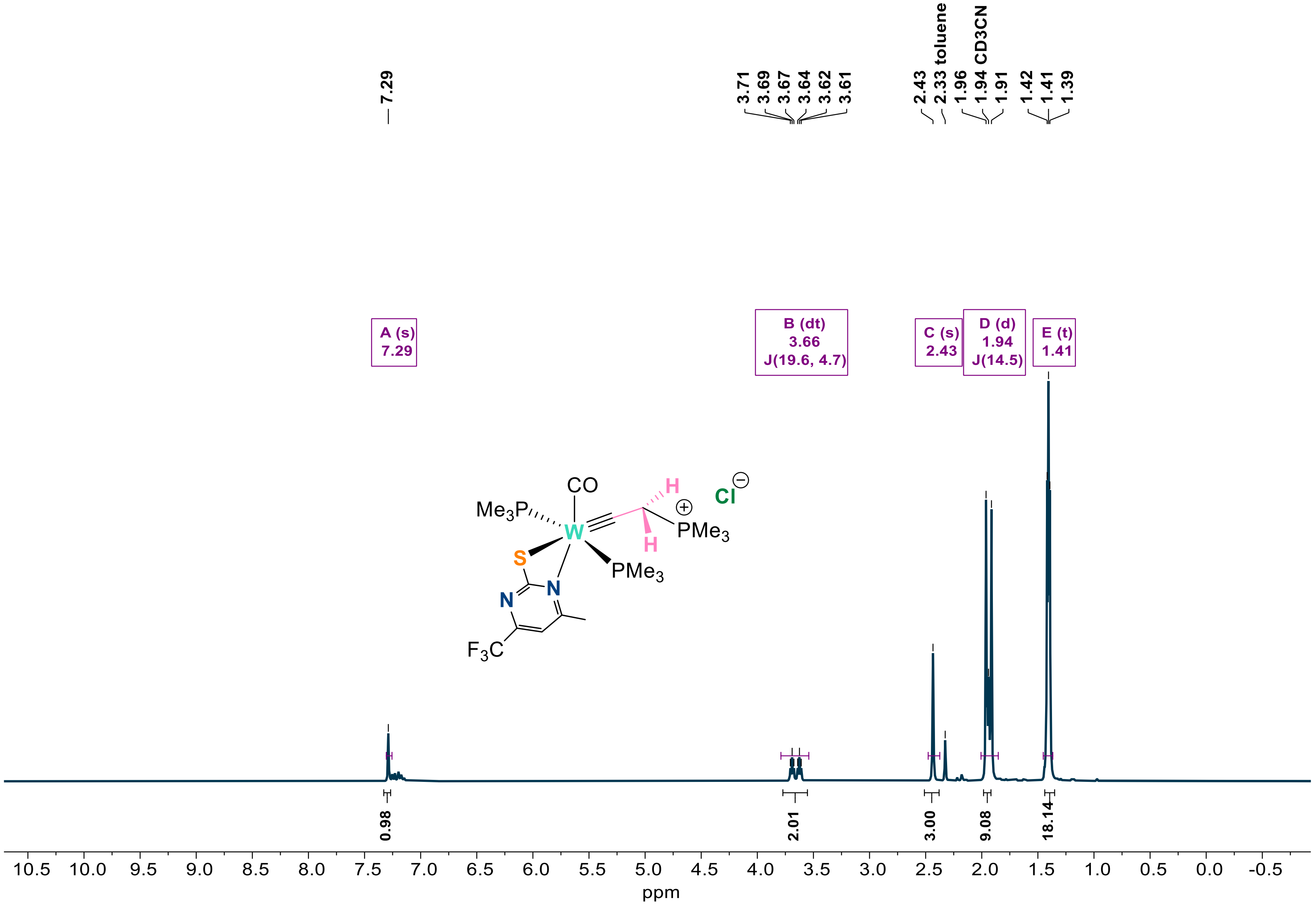

Figure S4. ^{1}H NMR spectrum of **2-Cl** in CD_3CN.

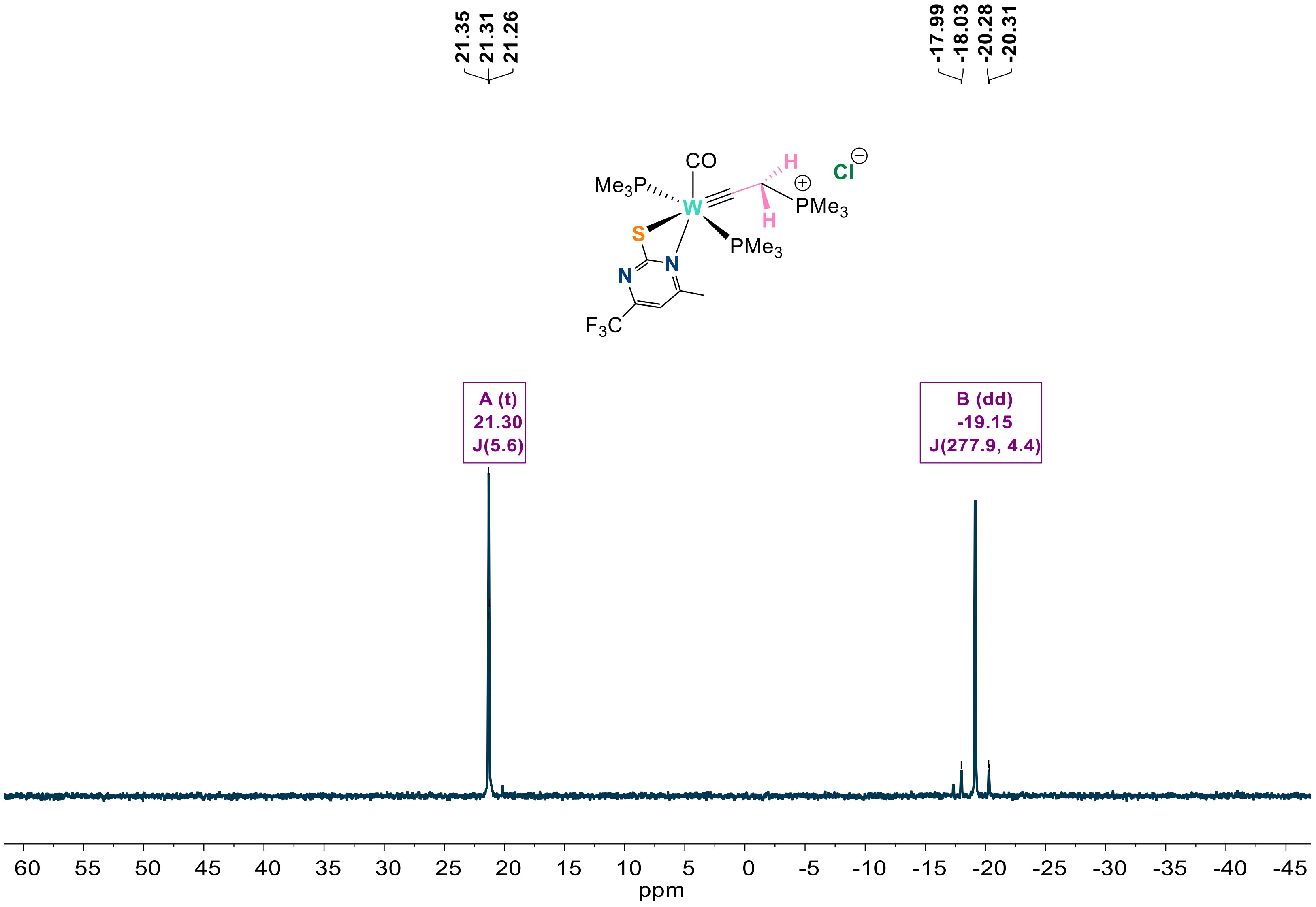

Figure S5. $^{31}P\{^{1}H\}$ NMR spectrum of **2-Cl** in CD_3CN.

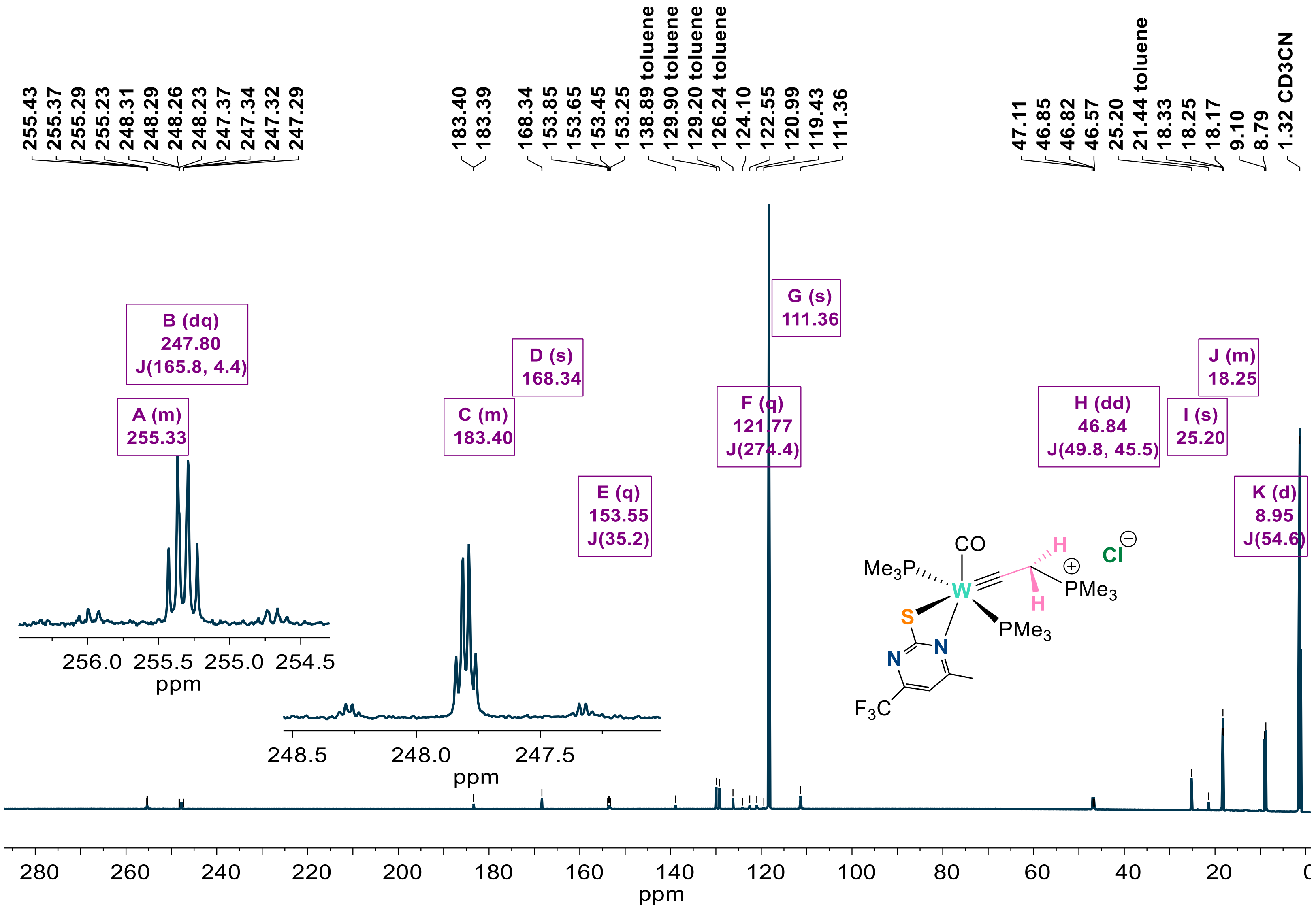

Figure S6. ^{13}C NMR spectrum of **2-Cl** in CD_3CN.

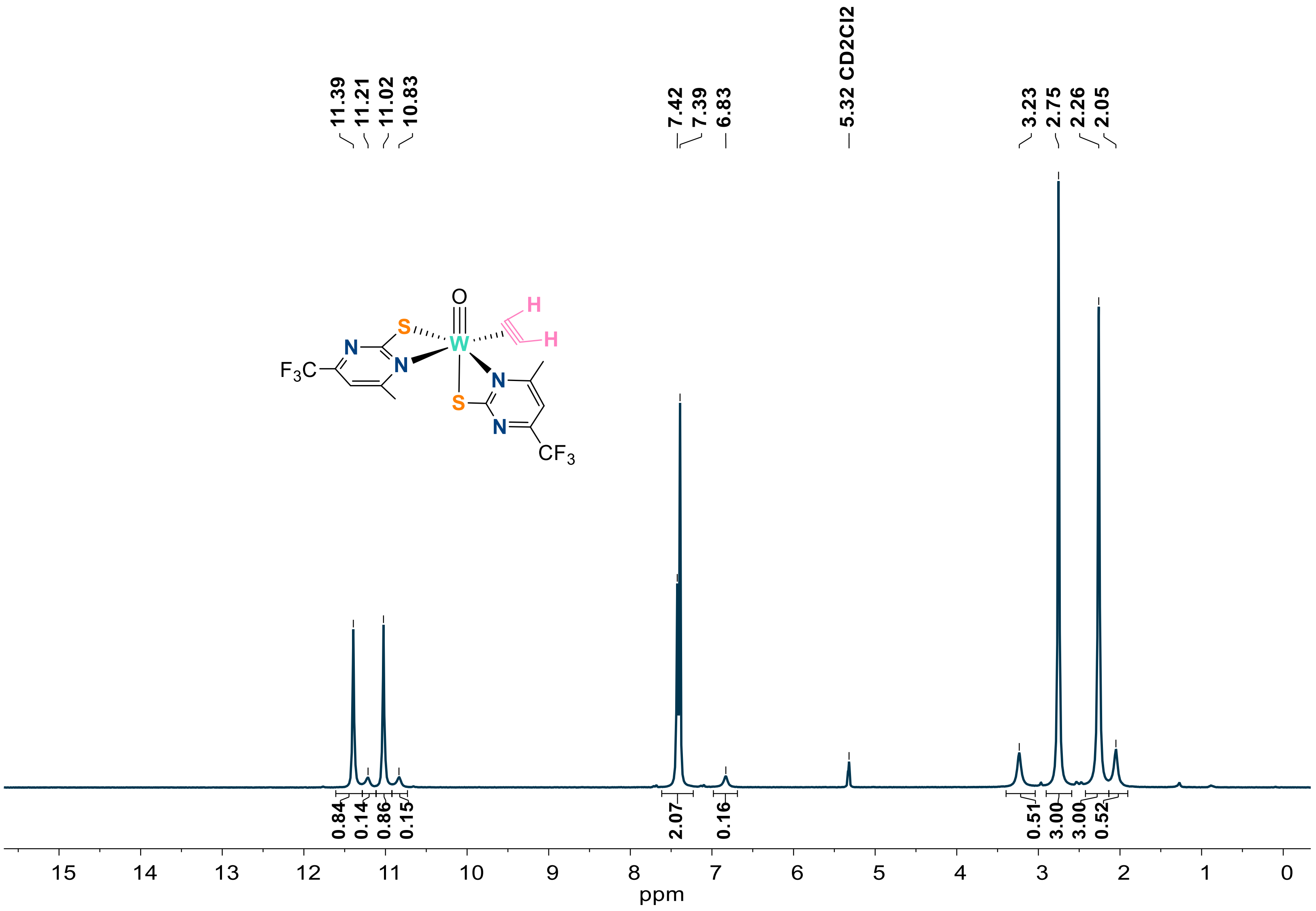

Figure S7. ^{1}H NMR spectrum of **3** in CD_2Cl_2.

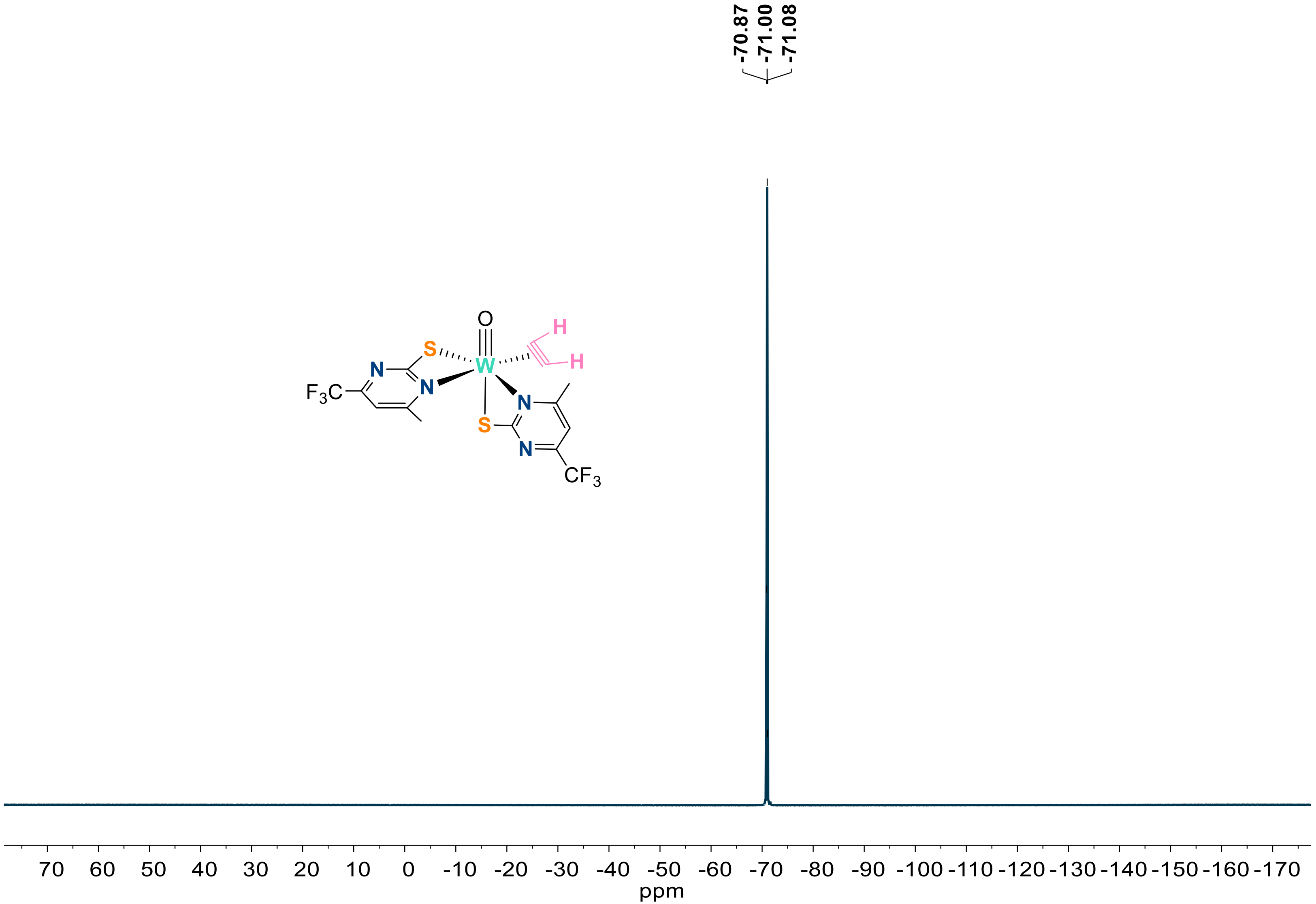

Figure S8. ^{19}F NMR spectrum of **3** in CD_2Cl_2.

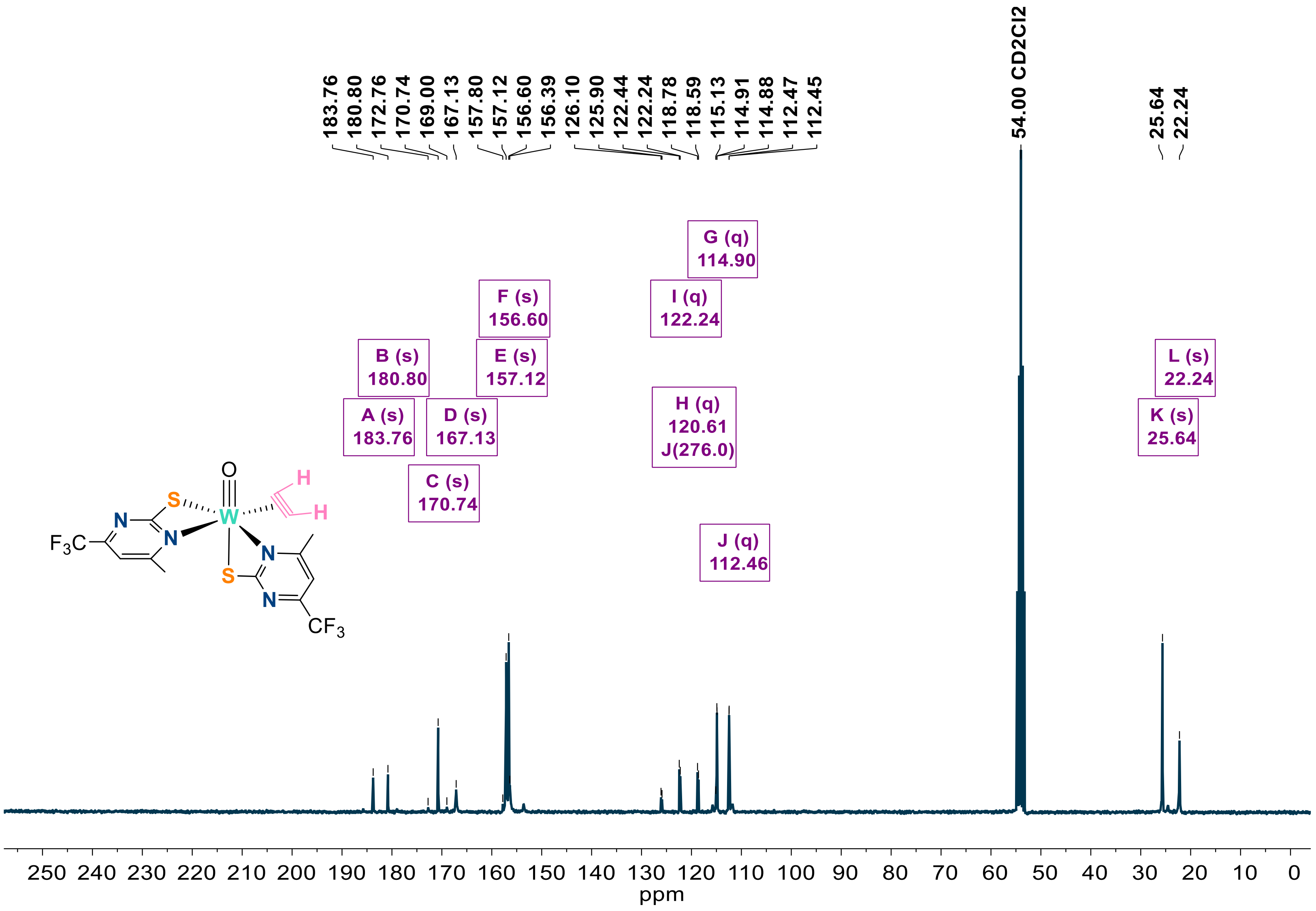

Figure S9. ^{13}C NMR spectrum of **3** in CD_2Cl_2.

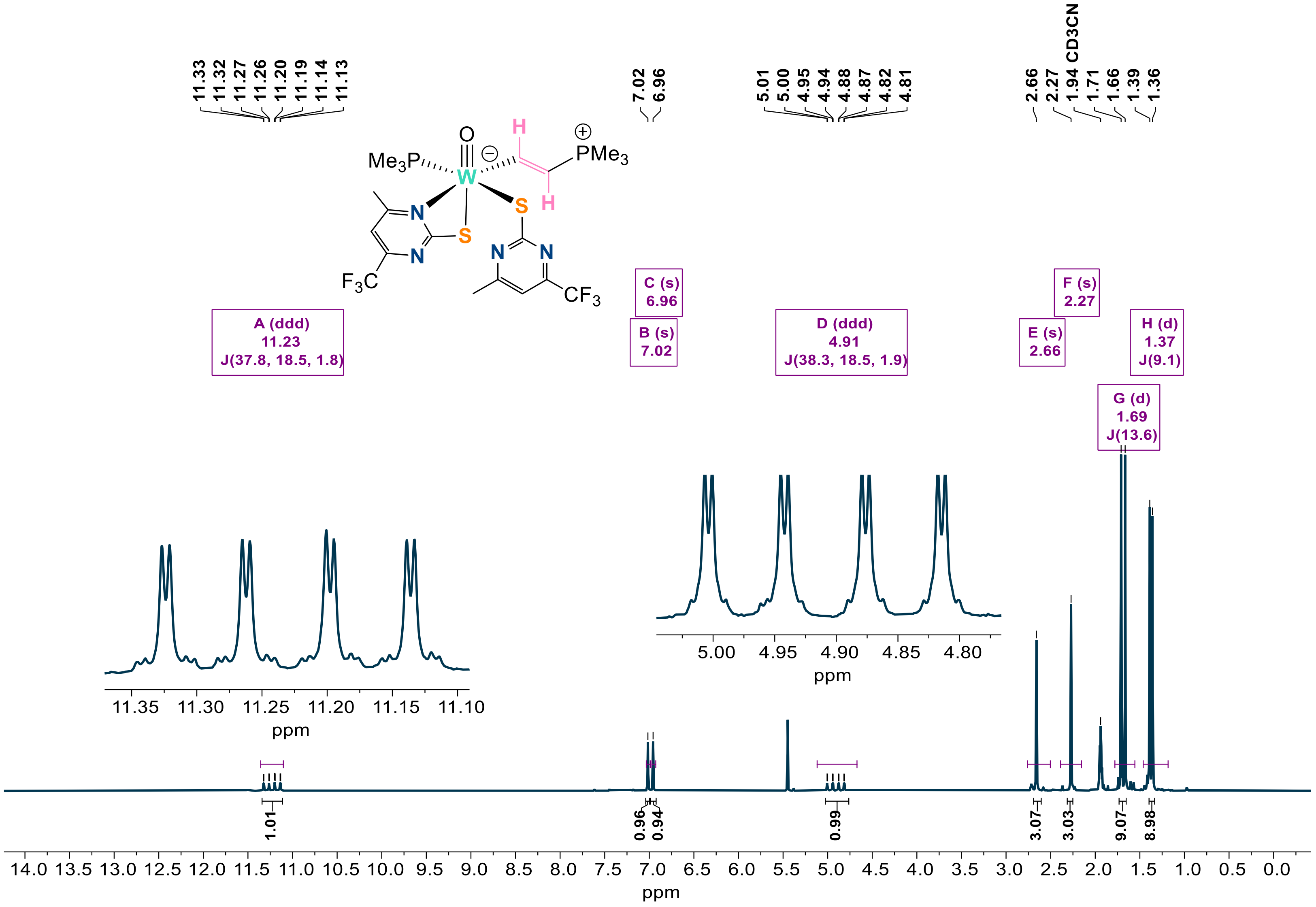

Figure S10. ^{1}H NMR spectrum of **3c** in CD_3CN.

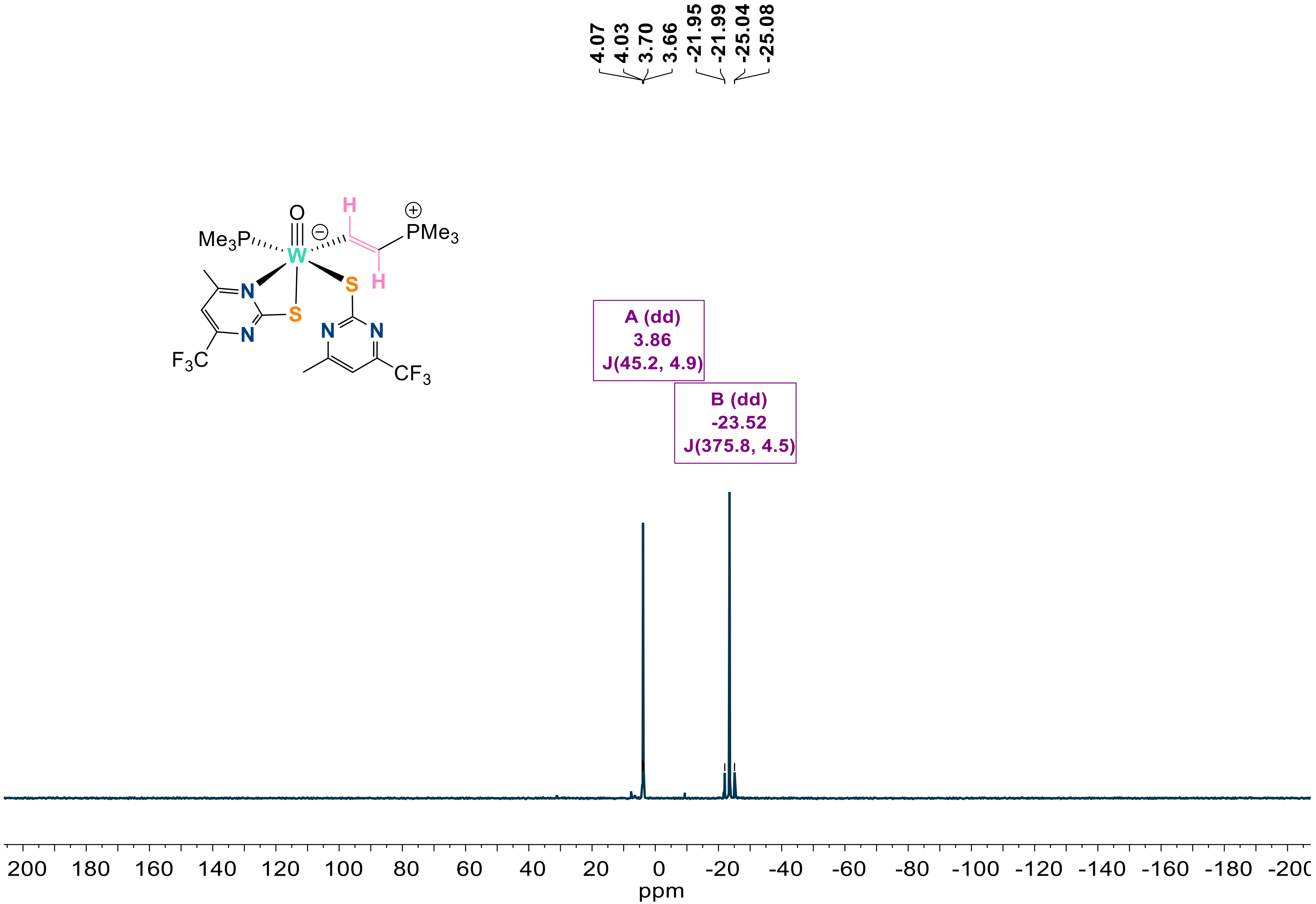

Figure S11. $^{31}P\{^{1}H\}$ NMR spectrum of **3c** in CD_3CN.

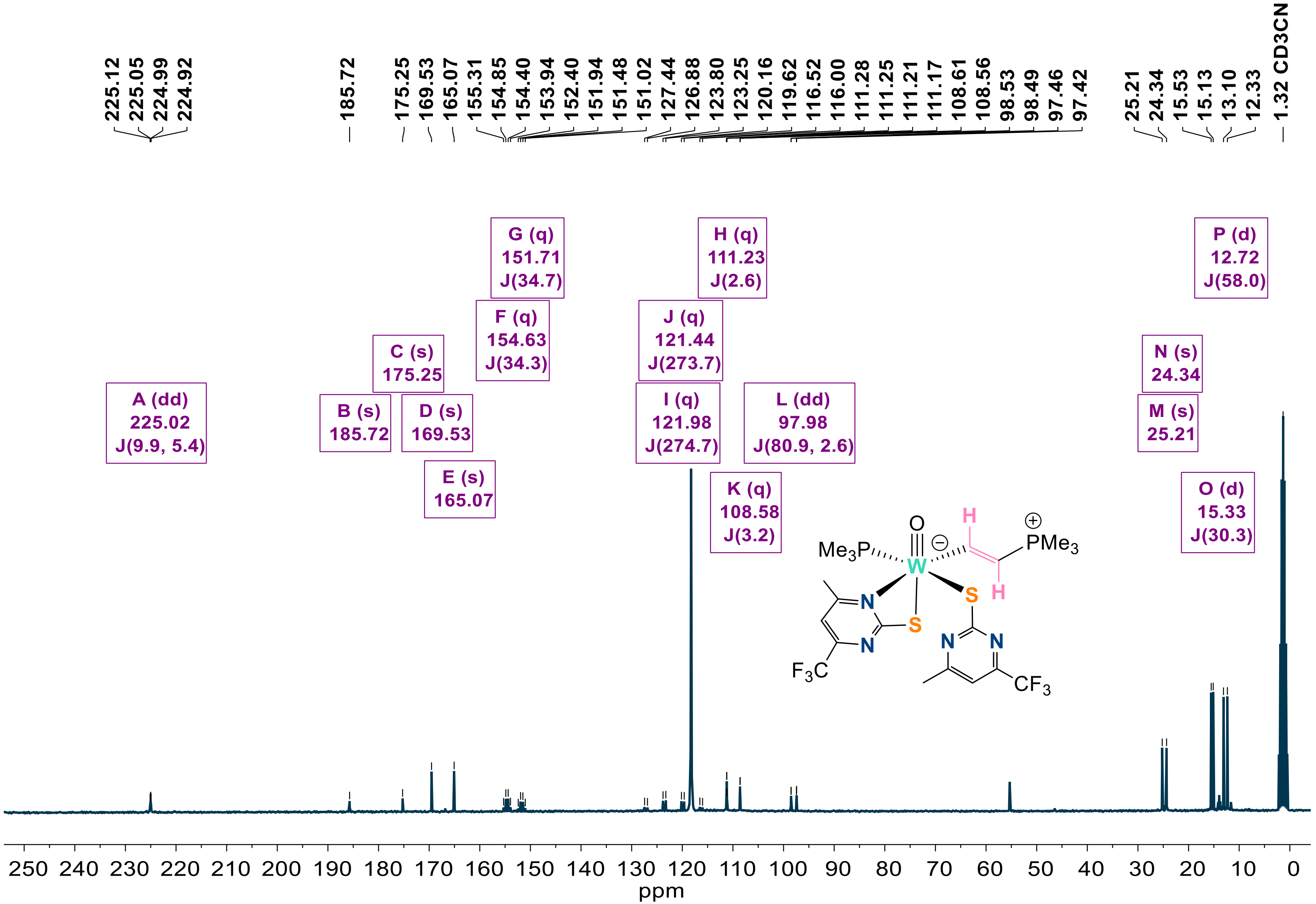

Figure S12. ^{13}C NMR spectrum of **3c** in CD_3CN.

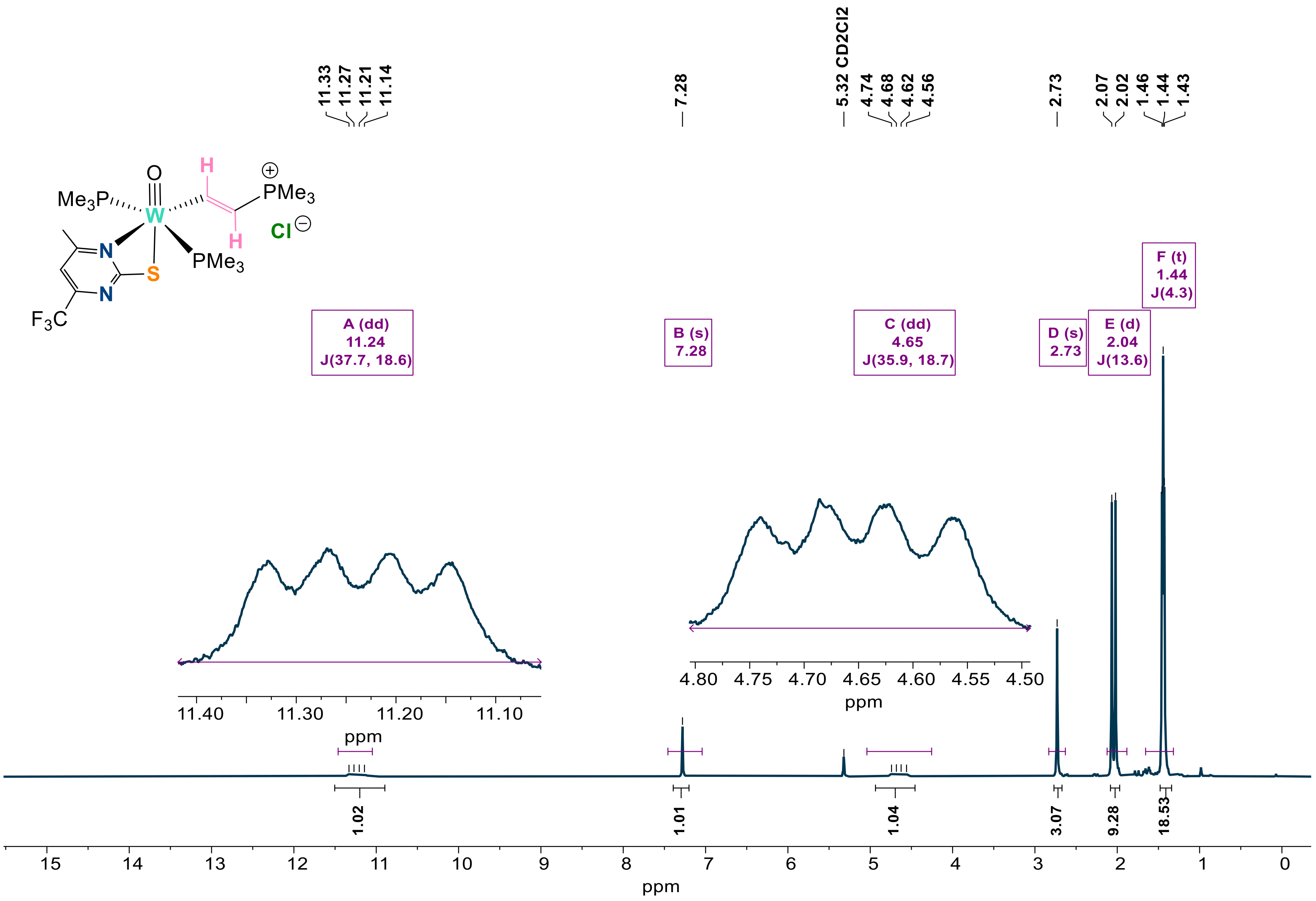

Figure S13. ^{1}H NMR spectrum of **4-Cl** in CD_2Cl_2.

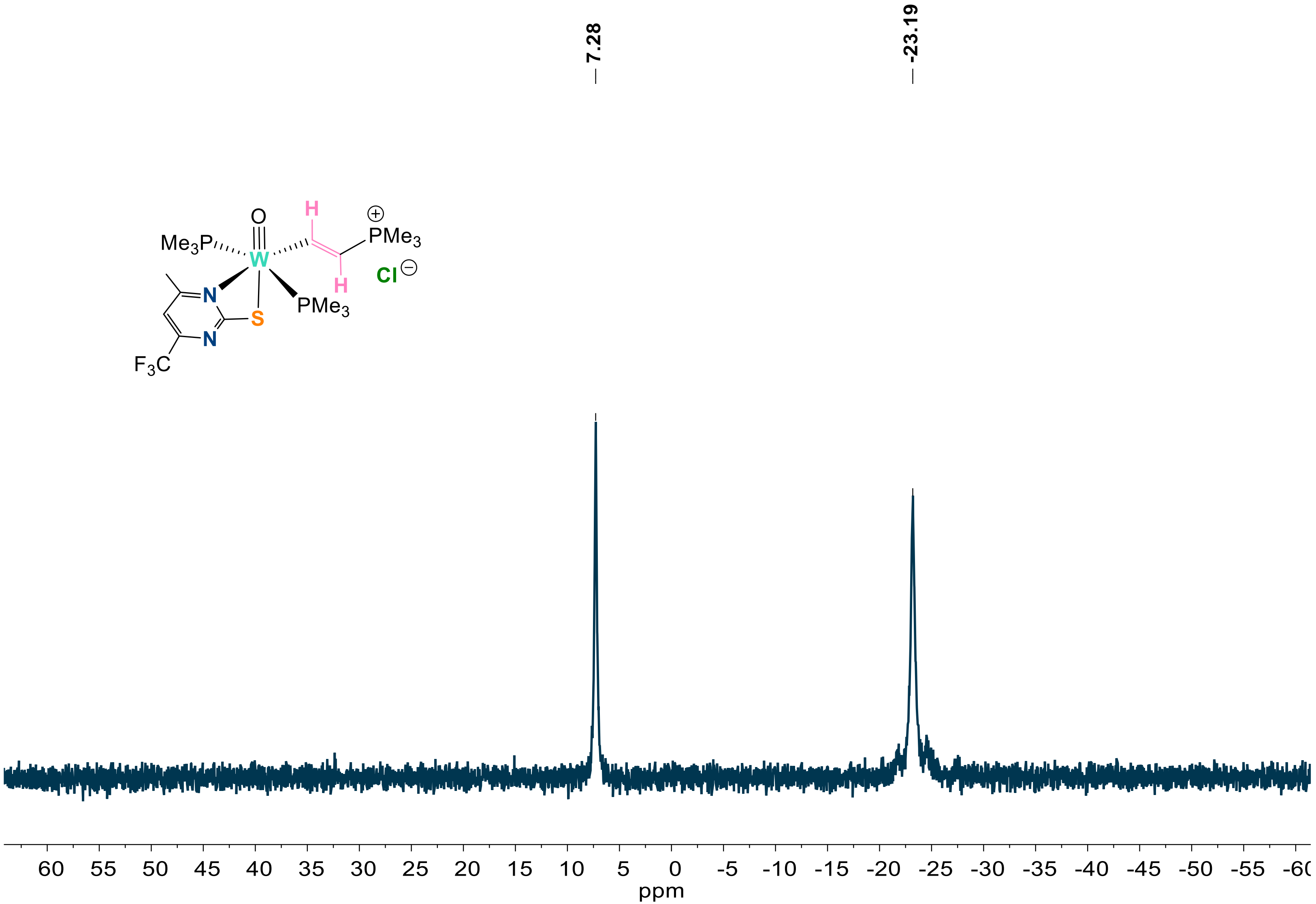

Figure S14. $^{31}P\{^{1}H\}$ NMR spectrum of **4-Cl** in CD_2Cl_2.

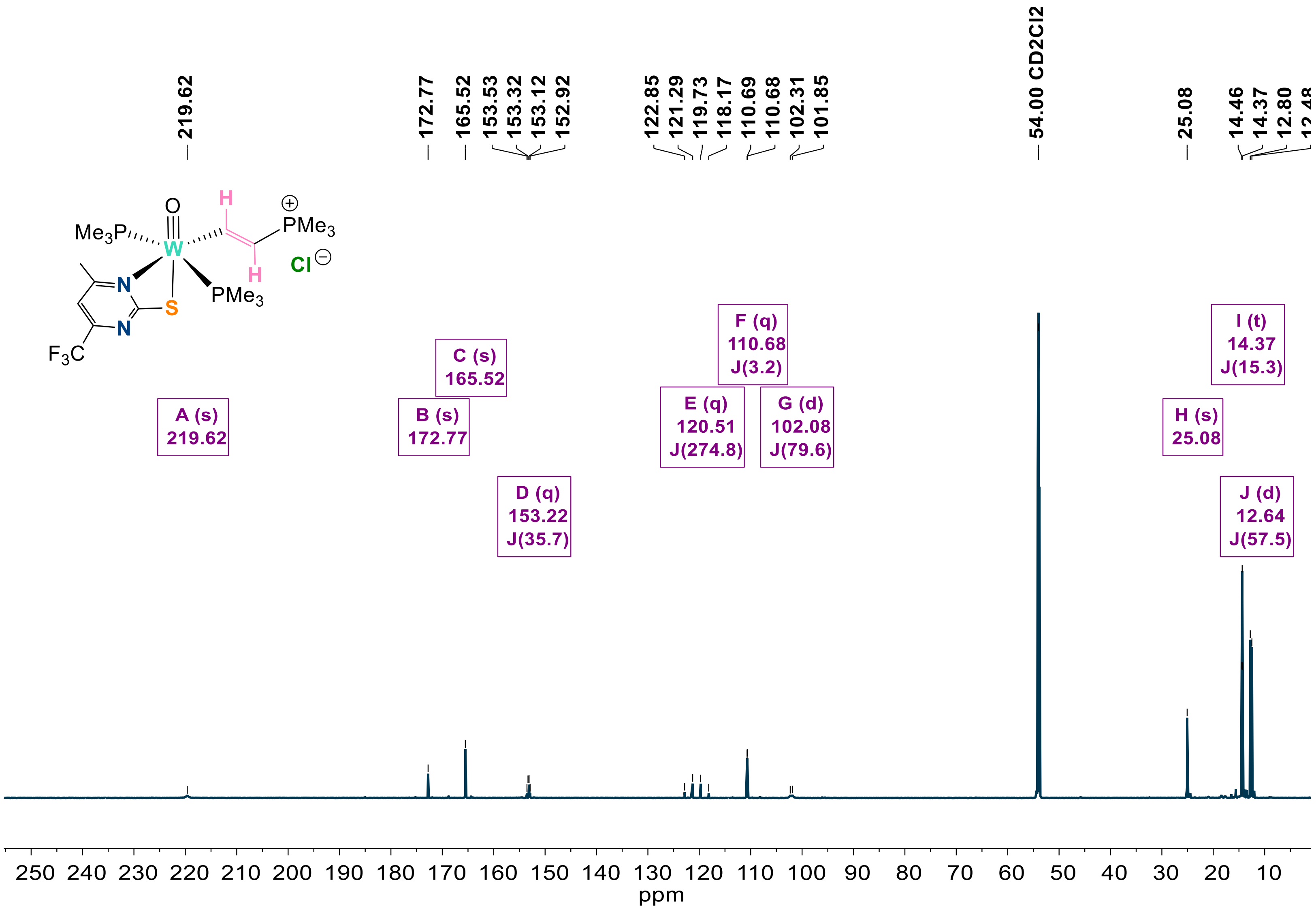

Figure S15. ^{13}C NMR spectrum of **4-Cl** in CD_2Cl_2.

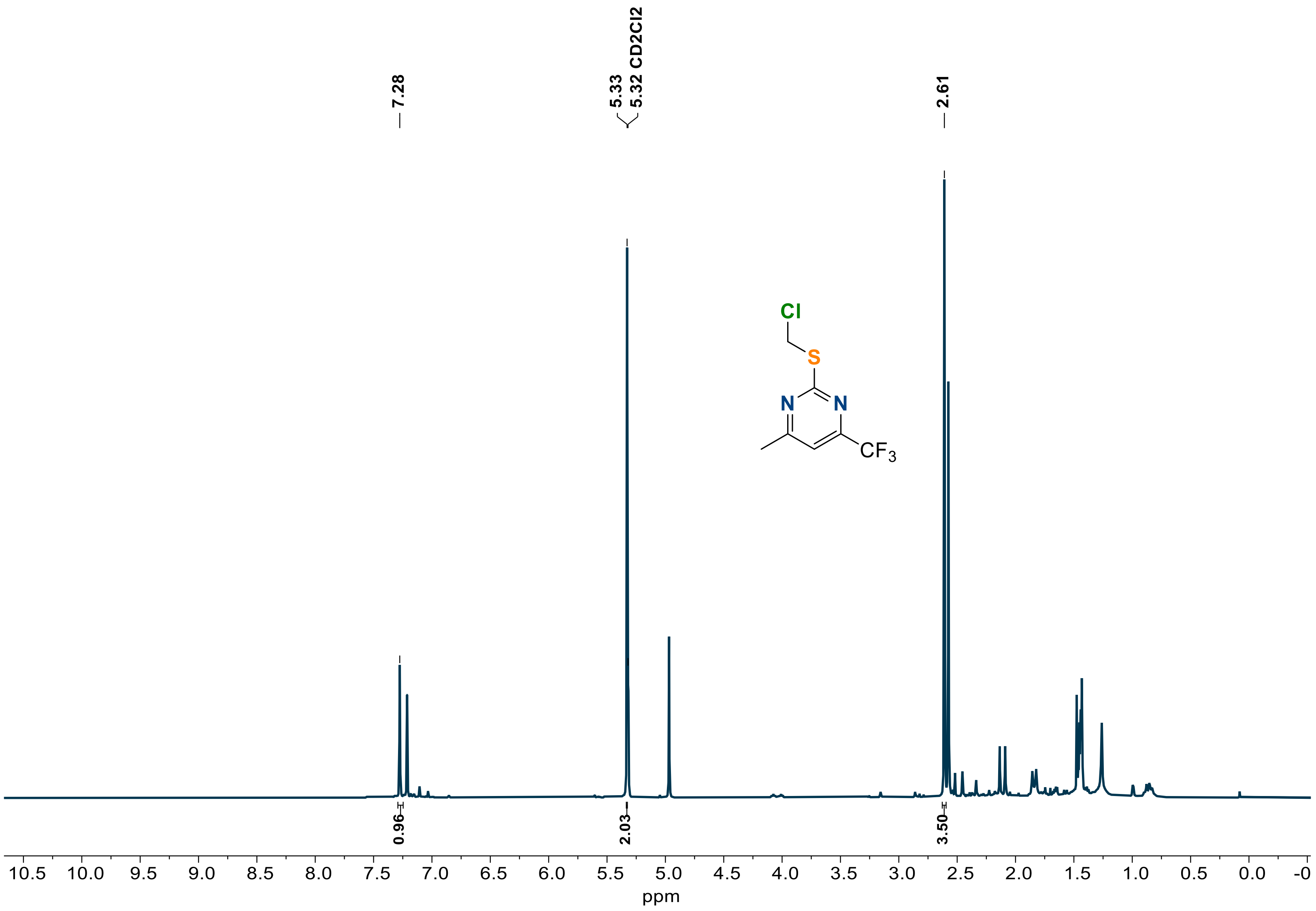

Figure S16. ^{1}H NMR spectrum of a mixture of $PymSCH_2Cl$ (peaks denoted) and $(PymS)_2CH_2$ in CD_2Cl_2.

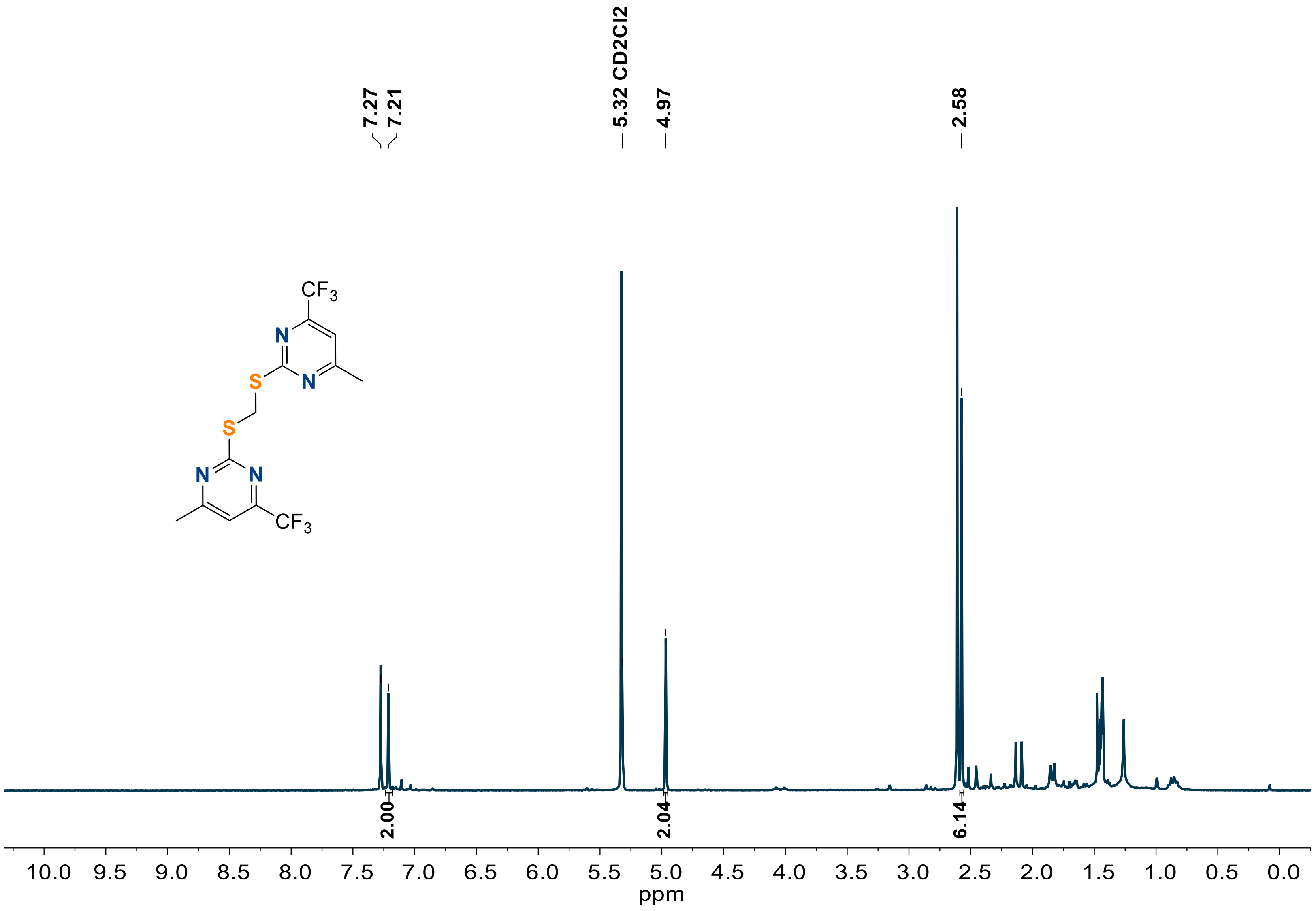

Figure S17. ^{1}H NMR spectrum of a mixture of $PymSCH_2Cl$ and $(PymS)_2CH_2$ (peaks denoted in CD_2Cl_2.

6 NMR Data of All Relevant Compounds

6.1 Carbonyl Complexes

Table S1. ^{1}H and $^{31}P\{^{1}H\}$ NMR data of carbonyl complexes.

**Compound**	**CD₂Cl₂**	**CD₃CN**
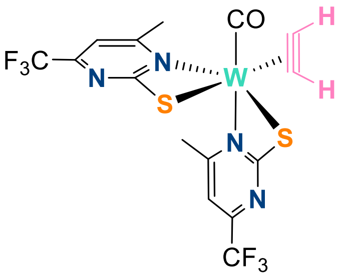<br>**1**	^{1}H NMR:<br>14.00 (s, 1H, C≡CH)<br>12.65 (s, 1H, C≡CH)<br>7.35 (s, 1H, pymH)<br>6.97 (s, 1H, pymH)<br>2.13 (s, 3H, CH_3)<br>1.39 (s, 3H, CH_3)	^{1}H NMR:<br>14.17(s, 1H, C≡CH)<br>12.92 (s, 1H, C≡CH)<br>7.57 (s, 1H, pymH)<br>7.18 (s, 1H, pymH)<br>2.13 (s, 3H, CH_3)<br>1.39 (s, 3H, CH_3)
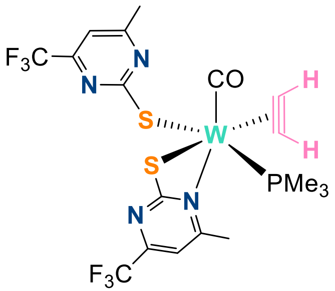<br>**1a**	^{1}H NMR:<br>12.50 (s, 1H, C≡CH),<br>12.14 (s, 1H, C≡CH),<br>7.28 (s, 1H, pymH),<br>7.03 (s, 1H, pymH)<br>3.08 (s, 3H, CH_3)<br>2.49 (s, 3H, CH_3),<br>1.08 (d, J = 8.8 Hz, 9H, PMe_3)<br>$^{31}P\{^{1}H\}$ NMR:<br>–27.43 (s(d), J = 236.2 Hz, $WPMe_3$)	^{1}H NMR:<br>12.62 (s, 1H, C≡CH),<br>12.31 (s, 1H, C≡CH),<br>7.41 (s, 1H, pymH),<br>7.16 (s, 1H, pymH)<br>3.03 (s, 3H, CH_3)<br>2.43 (s, 3H, CH_3),<br>1.04 (d, J = 9.2 Hz, 9H, PMe_3)<br>$^{31}P\{^{1}H\}$ NMR:<br>–26.14 (s(d), J = 238.1 Hz, $WPMe_3$)

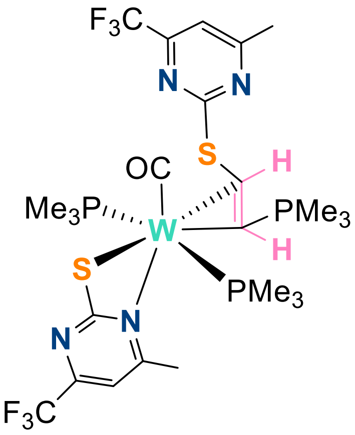<br>**1b''**	^{1}H NMR:<br>7.10 (s, 1H, pymH)<br>7.02 (s, 1H, pymH)<br>3.20 (dddd, J = 17.2, 12.8, 4.7, 1.6 Hz, 1H, CH)<br>2.69 (s, 3H, CH_3)<br>2.53 (s, 3H, CH_3)<br>1.78 (d, J = 12.8 Hz, 9H, PCH_3)<br>1.29 (d, J = 7.8 Hz, 9H, $WPCH_3$)<br>1.17 (d, J = 6.9 Hz, 9H, $WPCH_3$)<br>0.76 (dddd, J = 14.4, 7.5, 4.6, 2.3 Hz, 1H)<br>$^{31}P\{^{1}H\}$ NMR:<br>28.20 (d, J = 7.9 Hz, $CPMe_3$)<br>–9.50 (dd, J = 59.2, 3.7 Hz, $WPMe_3$)<br>–23.98 (d, J = 59.2 Hz, $WPMe_3$)	
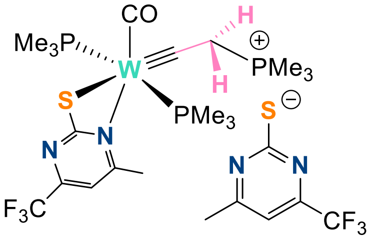<br>**2-PymS**	^{1}H NMR:<br>7.09 (s, 1H, pymH)<br>6.63 (s, 1H, pymH – anion)<br>4.07 (dt, J = 18.3, 4.2 Hz, 2H, CH_2)<br>2.40 (s, 3H, CH_3)<br>2.28 (s, 3H, CH_3 – anion)<br>2.11 (d, J = 14.0 Hz, 9H, PCH_3)<br>1.41 (t, J = 3.7 Hz, 18H, $WPCH_3$)<br>$^{31}P\{^{1}H\}$ NMR:<br>20.24 (m, $CPMe_3$)<br>–18.78 (d, J = 5.5 Hz, $WPMe_3$)	^{1}H NMR:<br>7.28 (s, 1H, pymH)<br>6.65 (s, 1H, pymH – anion)<br>3.64 (dt, J = 19.3, 4.7 Hz, 2H, CH_2)<br>2.40 (s, 3H, CH_3)<br>2.23 (s, 3H, CH_3 – anion)<br>1.93 (d, J = 14.5 Hz, 9H, PCH_3)<br>1.38 (t, J = 3.7 Hz, 18H, $WPCH_3$)<br>$^{31}P\{^{1}H\}$ NMR:<br>20.24 (t, J = 6.1 Hz, $CPMe_3$)<br>–19.12 (d, J = 6.1 Hz, $WPMe_3$)

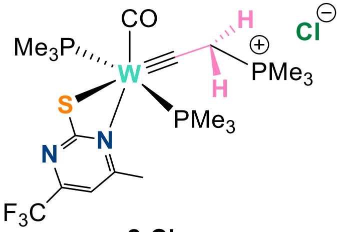<br><br>**2-Cl**	^{1}H NMR:<br>7.11 (s, 1H, pymH)<br>4.06 (dt, J = 19.9, 4.8 Hz, 2H, CH_2)<br>2.45 (s, 3H, CH_3)<br>2.13 (d, J = 14.5 Hz, 9H, PCH_3)<br>1.44 (t, J = 3.6 Hz, 18H, $WPCH_3$)<br>$^{31}P\{^1H\}$ NMR:<br>20.54 (t, J = 5.9 Hz, $CPMe_3$)<br>–18.81 (d, J = 5.7 Hz, $WPMe_3$)	^{1}H NMR:<br>7.29 (s, 1H, pymH)<br>3.66 (dt, J = 19.6, 4.7 Hz, 2H, CH_2)<br>2.43 (s, 3H, CH_3)<br>1.94 (d, J = 14.5 Hz, 9H, PCH_3)<br>1.41 (t, 18 H, $WPCH_3$)<br>$^{31}P\{^1H\}$ NMR:<br>21.30 (t, J = 5.6 Hz, $CPMe_3$),<br>–19.15 (d(dd), J = 277.9, 4.4 Hz, $WPMe_3$)

6.2 Oxo Complexes

Table S2. ^{1}H and $^{31}P\{^{1}H\}$ NMR data of oxo complexes.

**Compound**	**CD₂Cl₂**	**CD₃CN**
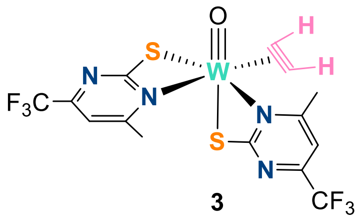<br>**3**	^{1}H NMR:<br>11.39 (s, 1H, C≡CH)<br>11.02 (s, 1H, C≡CH)<br>7.42 (s, 1H, pymH)<br>7.39 (s, 1H, pymH)<br>2.75 (s, 3H, CH_3)<br>2.26 (s, 3H, CH_3)	^{1}H NMR:<br>11.51 (s(d), J = 10.3 Hz, 1H, C≡CH)<br>11.20 (s(d), J = 11.9 Hz, 1H, C≡CH)<br>7.65 (s, 1H, pymH)<br>7.61 (s, 1H, pymH)<br>2.72 (s, 3H, CH_3)<br>2.25 (s, 3H, CH_3)
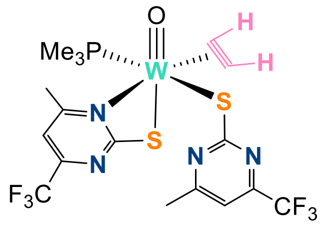<br>**3a**	^{1}H NMR:<br>10.54 (d, J = 18.7 Hz, 1H, C≡CH)<br>10.37 (d, J = 6.3 Hz, 1H, C≡CH)<br>7.03 (s, 1H, pymH)<br>6.93 (s, 1H, pymH)<br>2.75 (s, 3H, CH_3)<br>2.37 (s, 3H, CH_3)<br>1.61 (d, J = 10.0 Hz, 9H, PCH_3)<br>$^{31}P\{^{1}H\}$ NMR:<br>–11.00 (s(d), J = 249.5 Hz, $WPMe_3$)	^{1}H NMR:<br>10.79 (d, J = 18.9 Hz, 1H, C≡CH)<br>10.30 (d, J = 6.3 Hz, 1H, C≡CH)<br>7.20 (s, 1H, pymH)<br>7.18 (s, 1H, pymH)<br>2.70 (s, 3H, CH_3)<br>2.37 (s, 3H, CH_3)<br>1.59 (d, J = 10.3 Hz, 9H, PCH_3)<br>$^{31}P\{^{1}H\}$ NMR:<br>–9.41 (s(d), J = 249.5 Hz, $WPMe_3$)

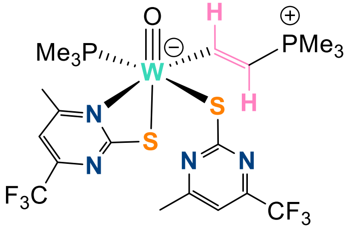 **3c**	^{1}H NMR:<br>11.41 (ddd(dddd), *J* = 38.4, 18.4, 1.7 Hz, 1H, WCH)<br>6.83 (s, 1H, pymH)<br>6.82 (s, 1H, pymH)<br>4.96 (ddd(dddd), *J* = 39.9, 18.4, 1.6 Hz, 1H, PCH)<br>2.63 (s, 3H, CH_3)<br>2.28 (s. 3H, CH_3)<br>1.74 (d, *J* = 13.1 Hz, 9H, PCH_3)<br>1.40 (d, *J* = 8.9 Hz, 9H, $WPCH_3$)<br>$^{31}P\{^1H\}$ NMR:<br>1.07 (d, *J* = 4.3 Hz, $CPMe_3$)<br>–23.47 (d, *J* = 4.2 Hz, $WPMe_3$)	^{1}H NMR:<br>11.23 (ddd(dddd), *J* = 37.8, 18.5, 1.8 Hz, 1H, WCH)<br>7.02 (s, 1H, pymH)<br>6.96 (s, 1H, pymH)<br>4.91 (ddd(dddd), *J* = 38.3, 18.5, 1.9 Hz, 1H, PCH)<br>2.66 (s, 3H, CH_3)<br>2.27 (s. 3H, CH_3)<br>1.69 (d, *J* = 13.6 Hz, 9H, PCH_3)<br>1.37 (d, *J* = 9.1 Hz, 9H, $WPCH_3$)<br>$^{31}P\{^1H\}$ NMR:<br>3.86 (d(dd), *J* = 45.2, 4.9 Hz, $CPMe_3$)<br>–23.52 (d(dd), *J* = 375.8, 4.5 Hz, $WPMe_3$)
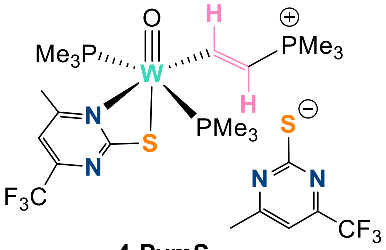 **4-PymS**		^{1}H NMR:<br>11.17 (m, WCH)<br>7.45 (s, 1H, pymH)<br>6.61 (s, 1H, pymH – anion)<br>4.66 (dd, *J* = 35.5, 18.9 Hz, 1H, PCH) 2.72 (s, 3H, CH_3)<br>2.22 (s, 3H, CH_3 – anion)<br>1.76 (d, *J* = 13.8 Hz, 9H, PCH_3)<br>1.41 (t, 18H, $WPCH_3$)<br>$^{31}P\{^1H\}$ NMR:<br>7.45 (bs, $CPMe_3$)<br>–23.02 (bs, $WPMe_3$)

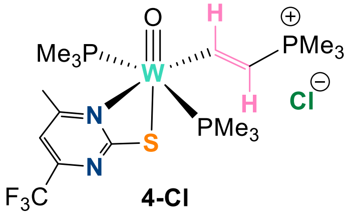 **4-Cl**	^{1}H NMR:<br>11.24 (dd, J = 37.7, 18.6 Hz, 1H, WCH)<br>7.28 (s, 1H, pymH)<br>4.65 (dd, J = 35.9, 18.7 Hz, 1H, PCH)<br>2.73 (s, 3H, CH_3)<br>2.04 (d, J = 13.6 Hz, 9H, PCH_3)<br>1.44 (t, J = 4.3 Hz, 18H, $WPCH_3$)<br>$^{31}P\{^{1}H\}$ NMR:<br>7.28 (bs, $CPMe_3$)<br>–23.19 (bs, $WPMe_3$)	^{1}H NMR:<br>11.17 (dd, J = 38.0, 18.8 Hz, 1H, WCH)<br>7.46 (s, 1H, pymH)<br>4.65 (dd, J = 35.5, 18.8 Hz, 1H, PCH)<br>2.72 (s, 3H, CH_3)<br>1.77 (d, J = 13.8 Hz, 9H, PCH_3)<br>1.41 (t, J = 4.5 Hz, 18H, $WPCH_3$)<br>$^{31}P\{^{1}H\}$ NMR:<br>6.51 (bs, $CPMe_3$)<br>–23.08 (bs, $WPMe_3$)
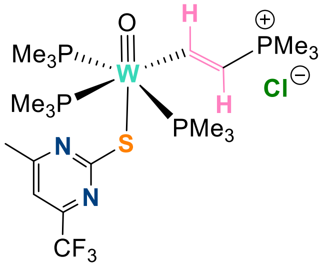 **4∗PMe$_3$**	^{1}H NMR:<br>11.06 (m, 1H, WCH)<br>7.30 (s, 1H, pymH)<br>5.76 (dd, J = 40.2, 18.8 Hz, 1H, PCH)<br>2.62 (s, 3H, CH_3)<br>1.99 (d, J = 13.5 Hz, 9H, PCH_3)<br>1.66 (d, J = 7.7 Hz, 9H, WCH3)<br>1.61 (t, J = 4.0 Hz, 18H, $WPCH_3$)<br>$^{31}P\{^{1}H\}$ NMR:<br>7.01 (bs, $CPMe_3$)<br>–27.39 (bs, $W(PMe_3)_2$)<br>–32.43 (bs, $WPMe_3$)	

6.3 Organic Compounds

Table S3. ^{1}H NMR data of organic compounds.

**Compound**	**CD_2Cl_2**	**CD_3CN**
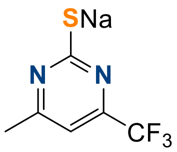	^{1}H NMR (+ Bu_4NCl):<br>6.56 (s, 1H, pymH)<br>2.24 (s, 3H, CH_3)	^{1}H NMR:<br>6.75 (s, 1H, pymH)<br>2.27 (s, 3H, CH_3)
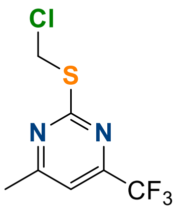	^{1}H NMR:<br>7.27 (s, 1H, pymH)<br>5.32 (s, 2H, CH_2)<br>2.60 (s, 3H, CH_3)	^{1}H NMR:<br>7.51 (s, 1H, pymH)<br>5.40 (s, 2H, CH_2)<br>2.56 (s, 3H, CH_3)
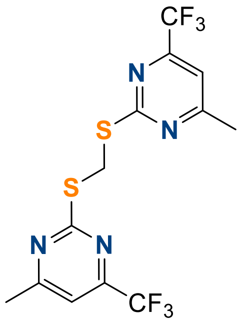	^{1}H NMR:<br>7.21 (s, 2H, pymH)<br>4.96 (s, 2H, CH_2)<br>2.57 (s, 6H, CH_3)	^{1}H NMR:<br>7.41 (s, 2H, pymH)<br>4.96 (s, 2H, CH_2)<br>2.55 (s, 6H, CH_3)

7 NMR Experiments

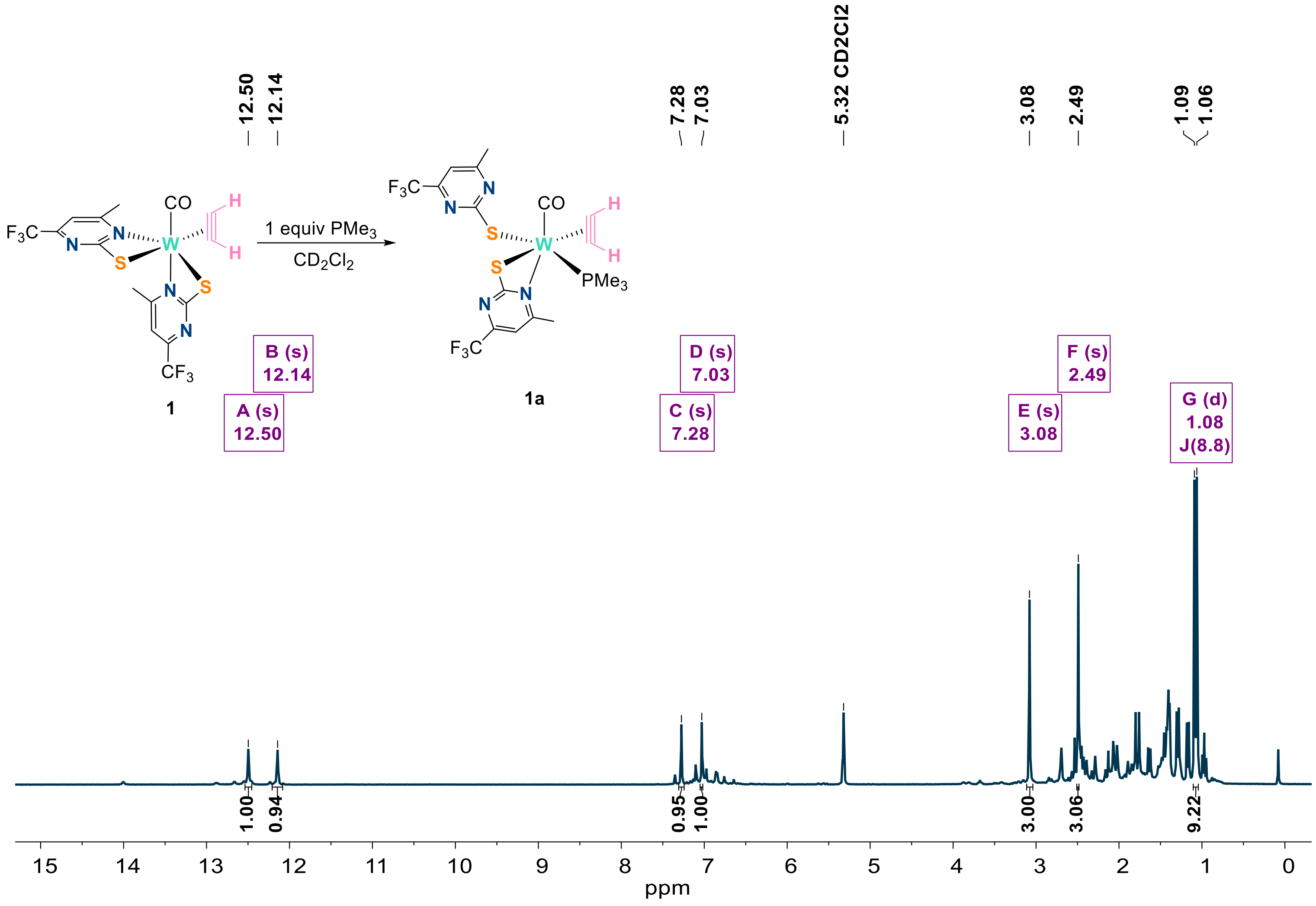

Figure S18. ^{1}H NMR spectrum of the reaction of **1** with 1 equiv of PMe_3 in CD_2Cl_2.

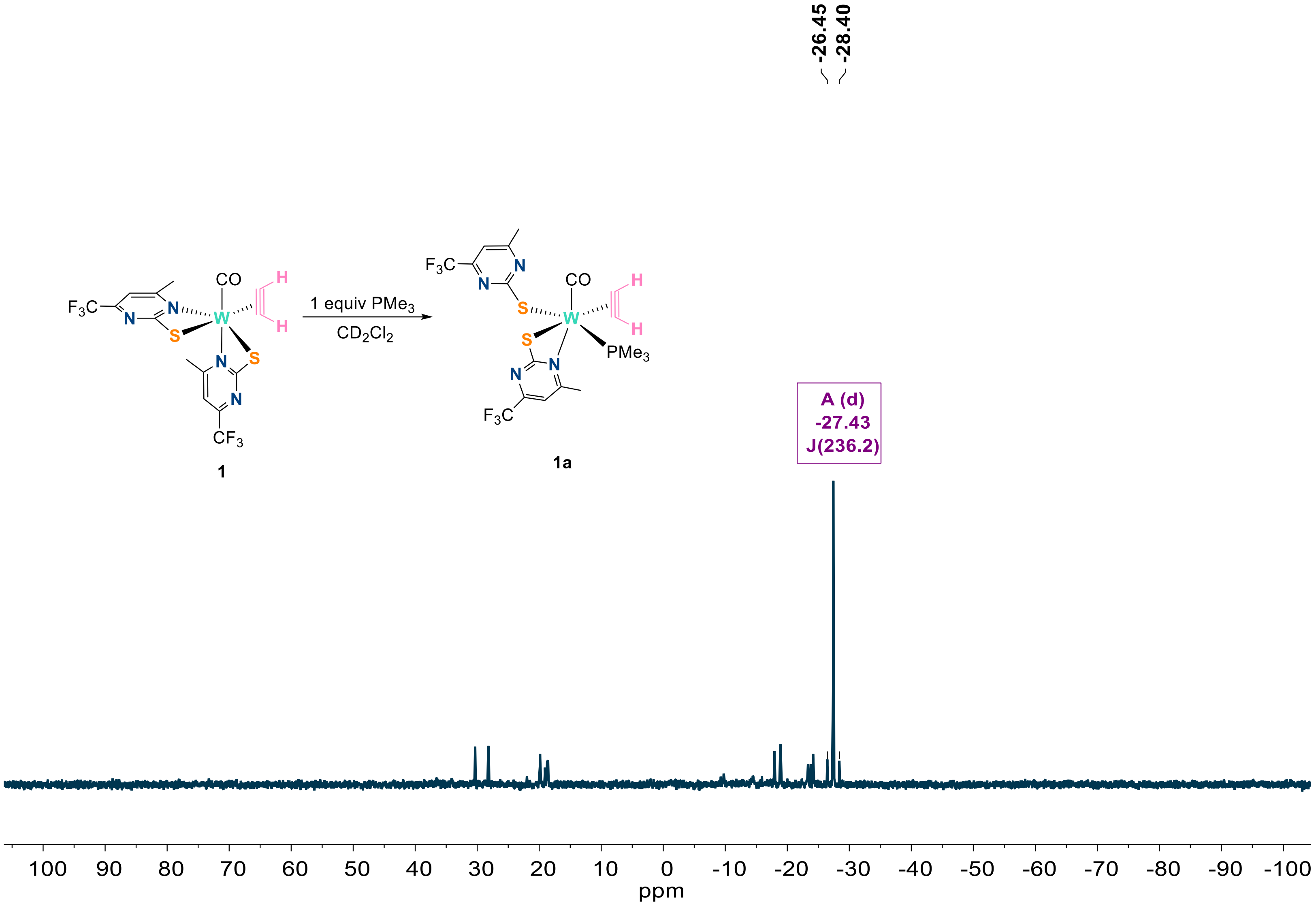

Figure S19. ${}^{31}P\{{}^{1}H\}$ NMR spectrum of the reaction of **1** with 1 equiv of PMe_3 in CD_2Cl_2.

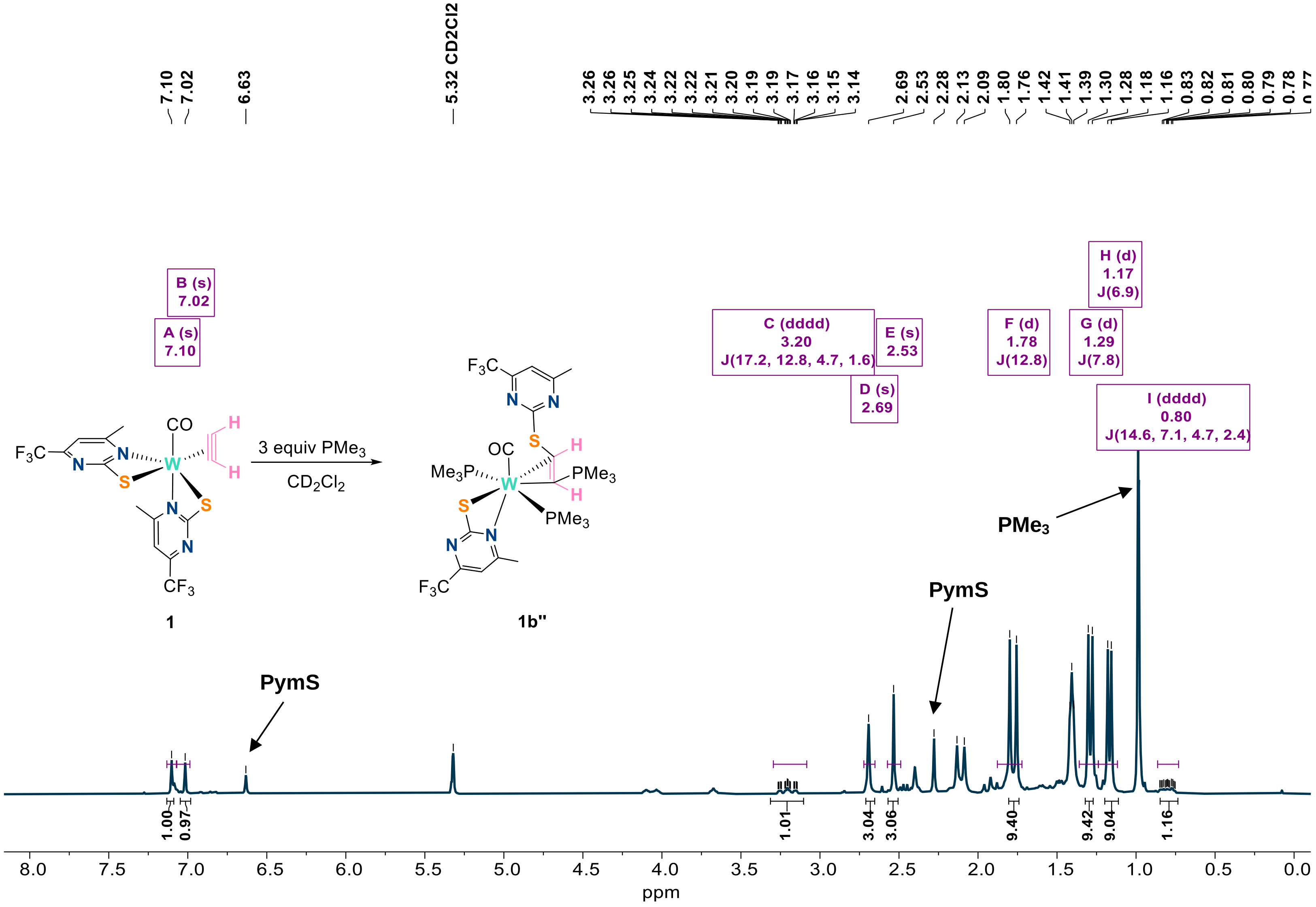

Figure S20. ^{1}H NMR spectrum of the reaction of **1** with 3 equiv of PMe_3 in CD_2Cl_2 recorded at room temperature.

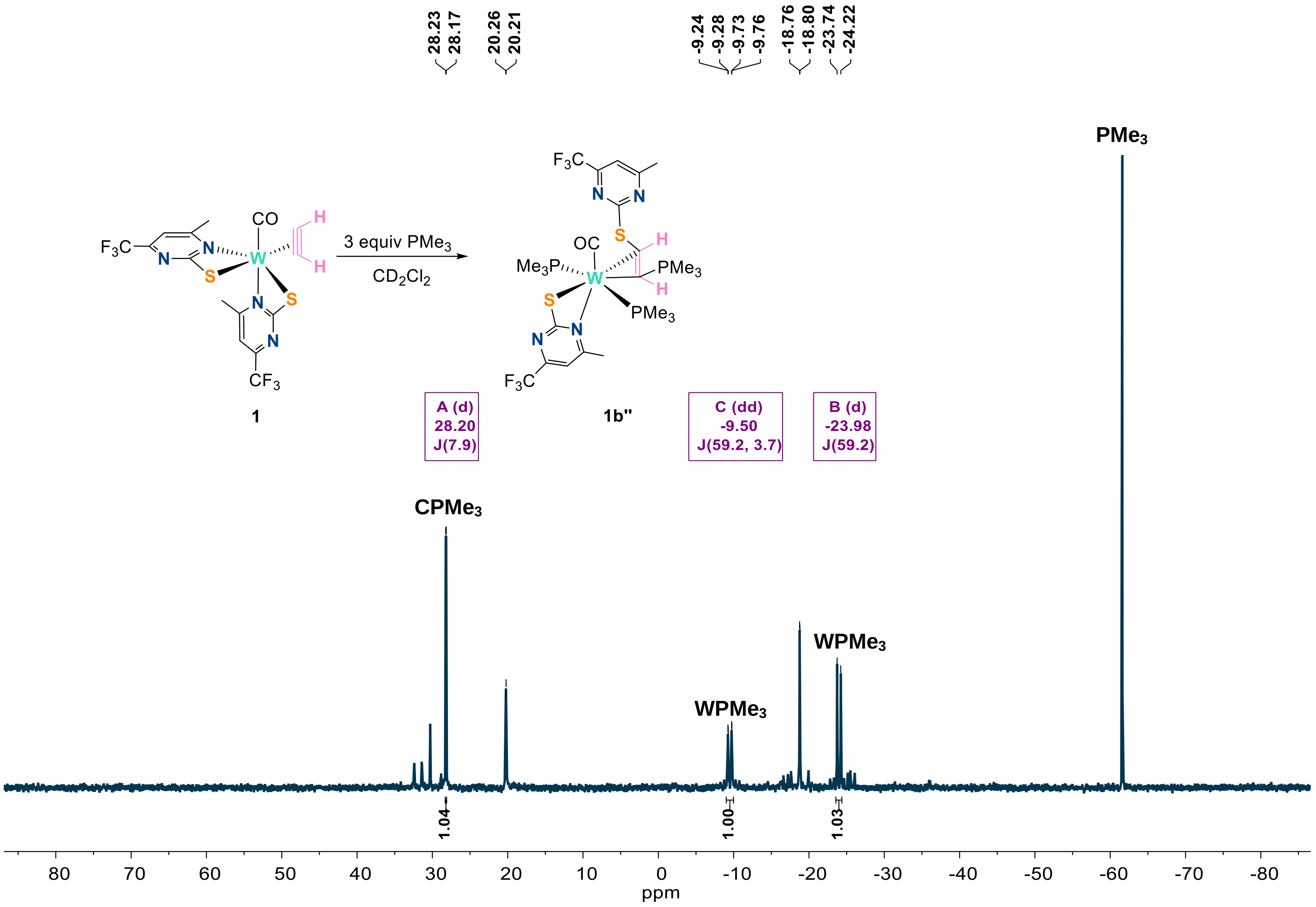

Figure S21. $^{31}P\{^{1}H\}$ NMR spectrum of the reaction of **1** with 3 equiv of PMe_3 in CD_2Cl_2 recorded at room temperature.

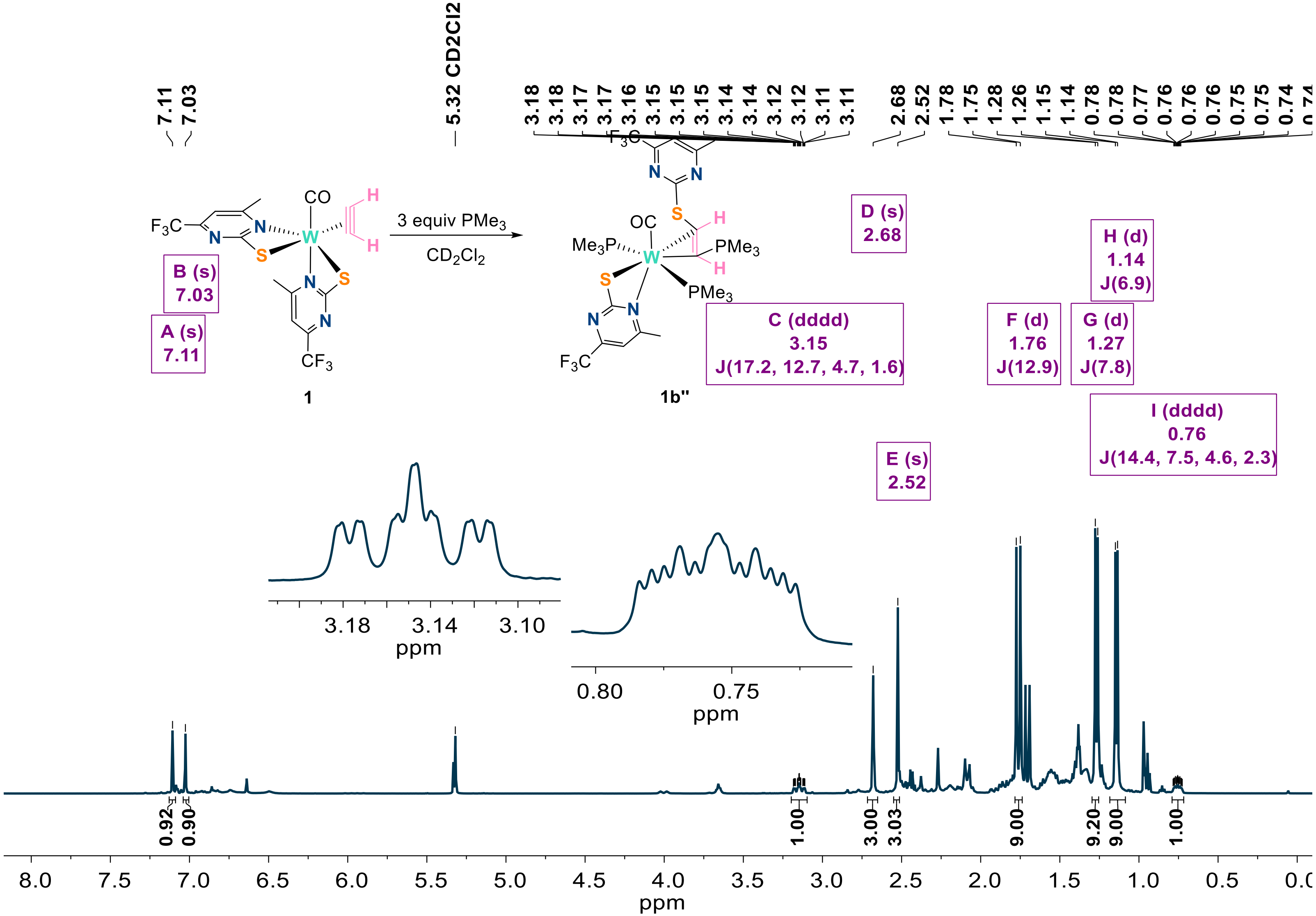

Figure S22. ^{1}H NMR spectrum of the reaction of **1** with 3 equiv of PMe_3 in CD_2Cl_2 recorded at 0 °C.

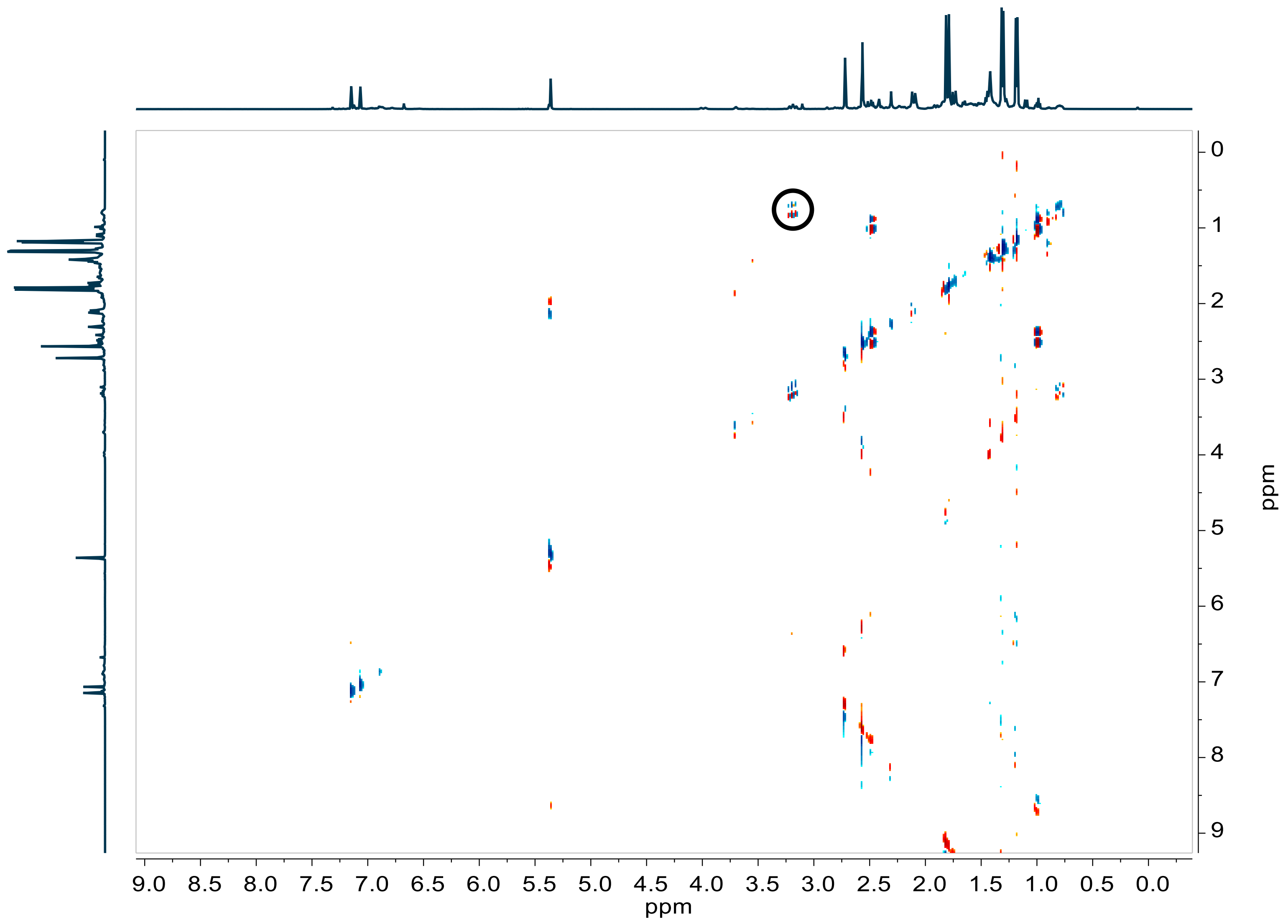

Figure S23. ^{1}H-^{1}H-correlated spectrum of the reaction of **1** with 3 equiv of PMe_3 in CD_2Cl_2 recorded at 0 °C.

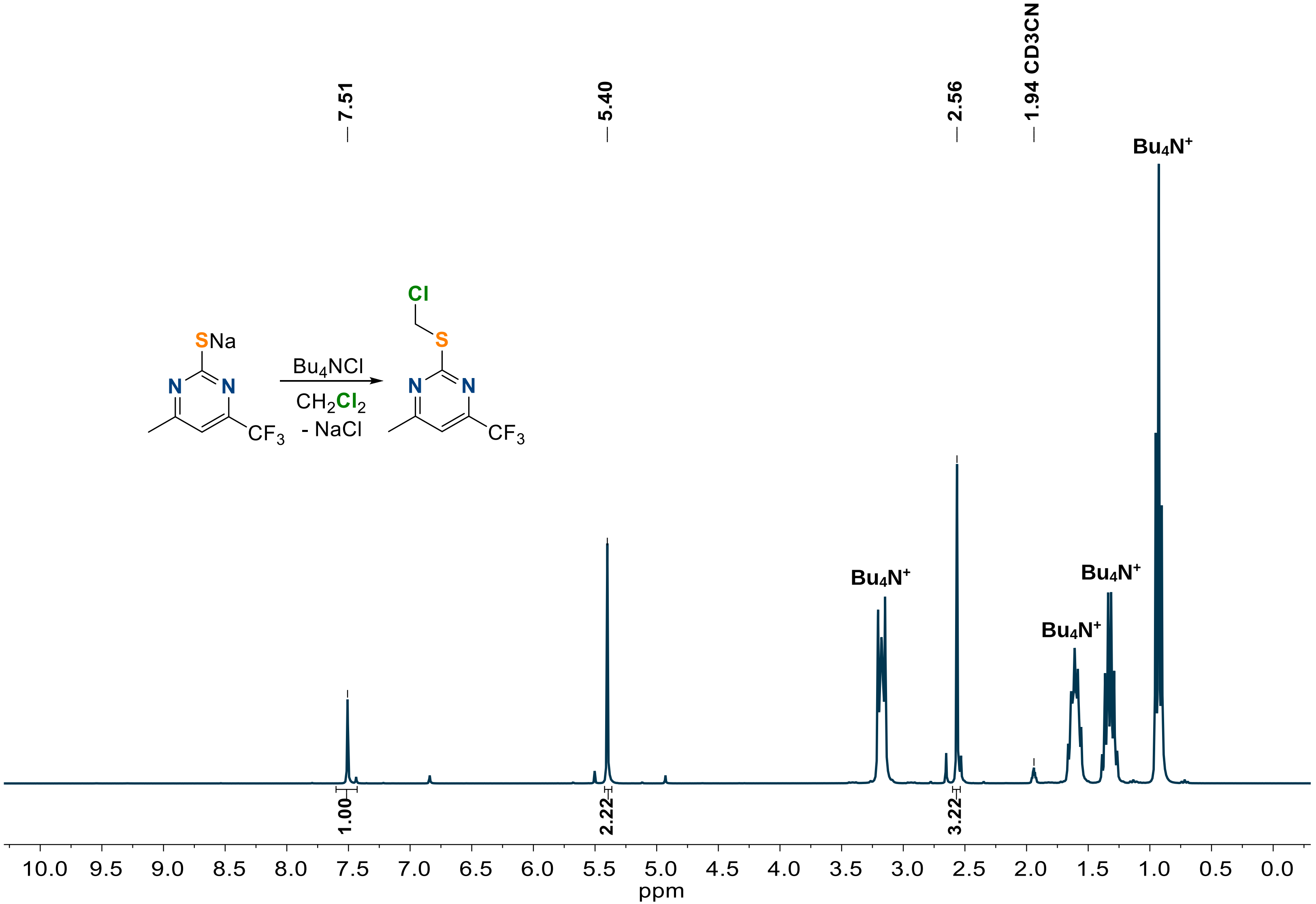

Figure S24. ^{1}H NMR spectrum of the reaction of Na(PymS) with $[Bu_4N]Cl$ and CH_2Cl_2 recorded in CD_3CN.

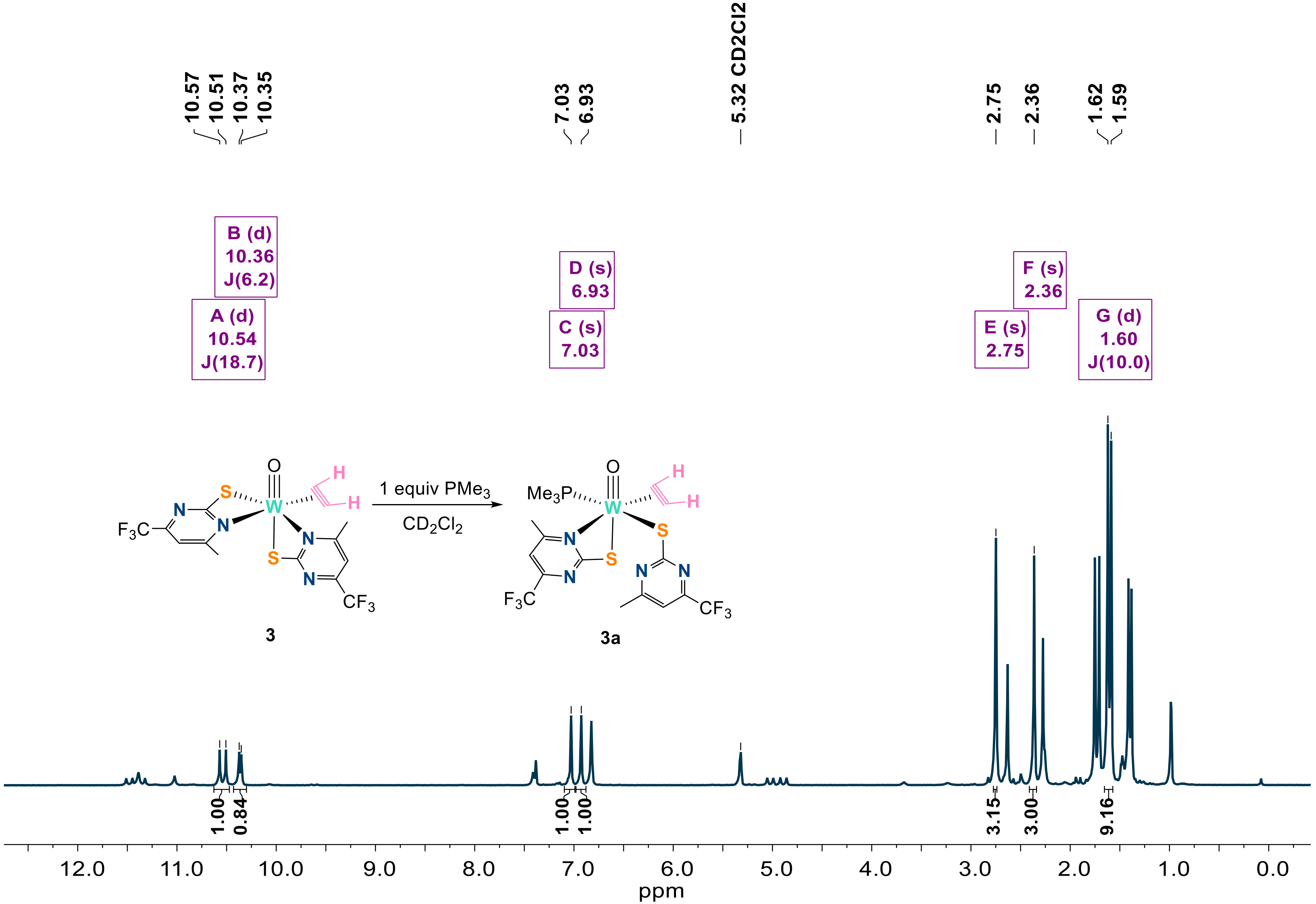

Figure S25. ^{1}H NMR spectrum of the reaction of **3** with 1.1 equiv of PMe_3 in CD_2Cl_2.

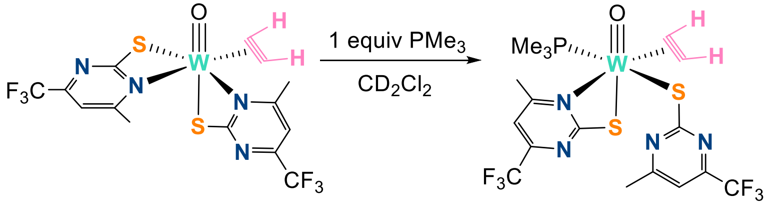

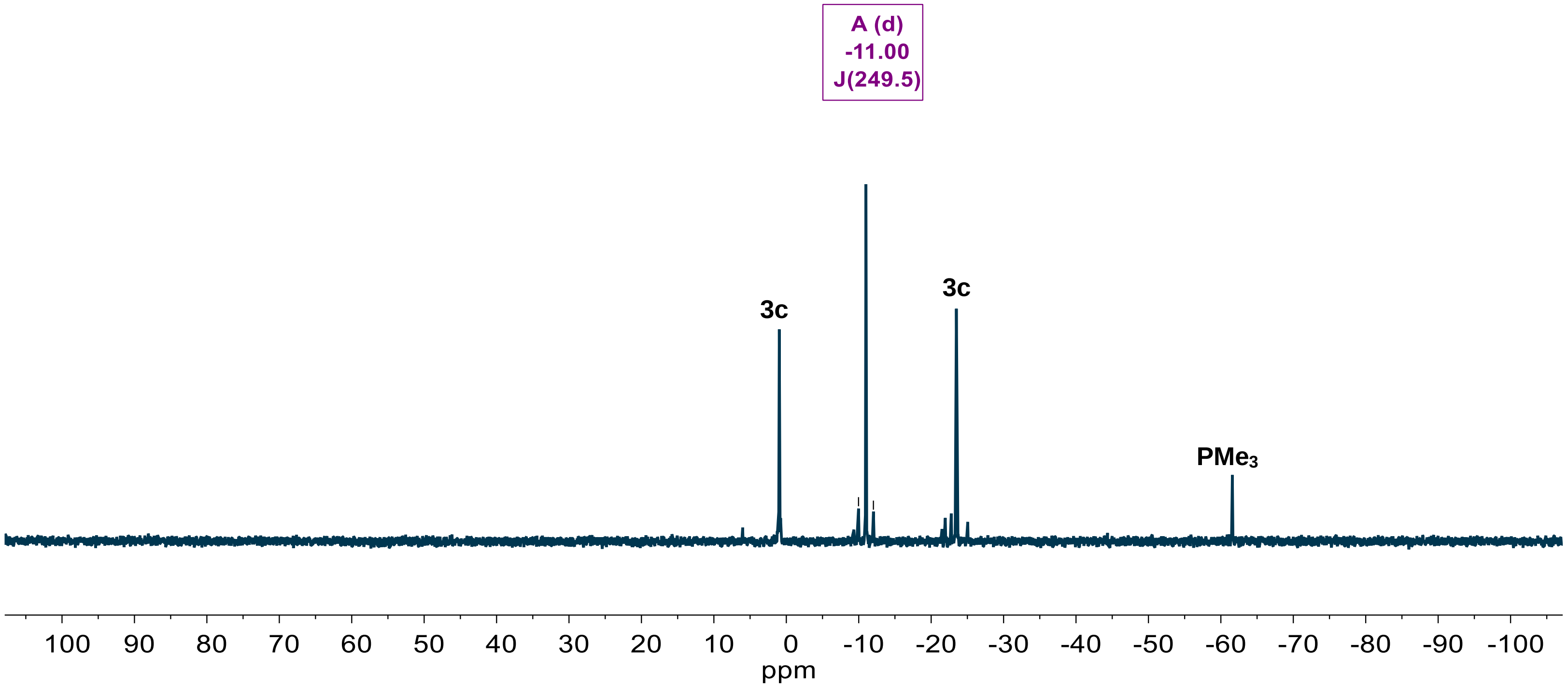

Figure S26. ${}^{31}P\{{}^{1}H\}$ NMR spectrum of the reaction of **3** with 1.1 equiv of PMe_3 in CD_2Cl_2.

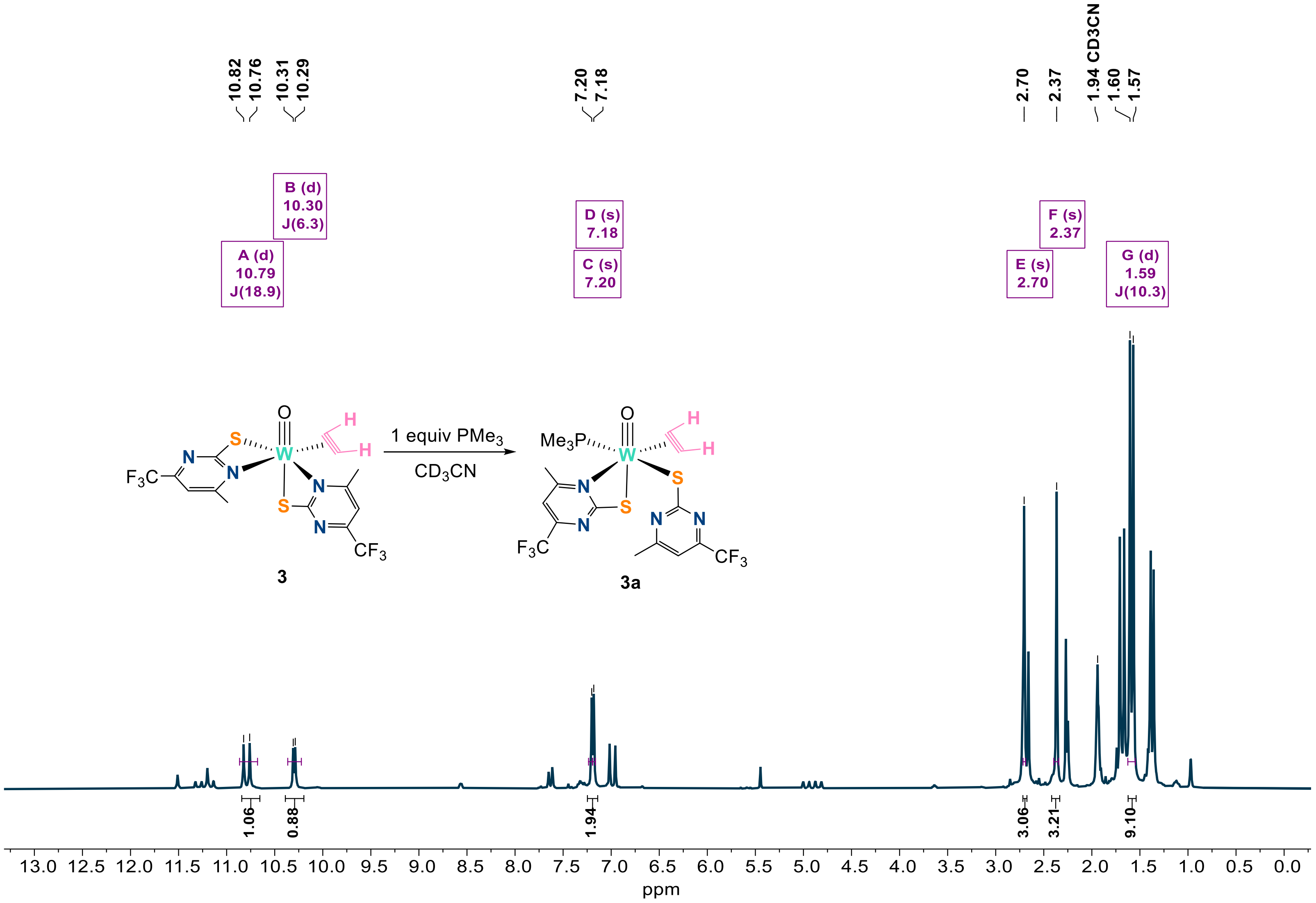

Figure S27. ^{1}H NMR spectrum of the reaction of **3** with 1.1 equiv of PMe_3 in CD_3CN.

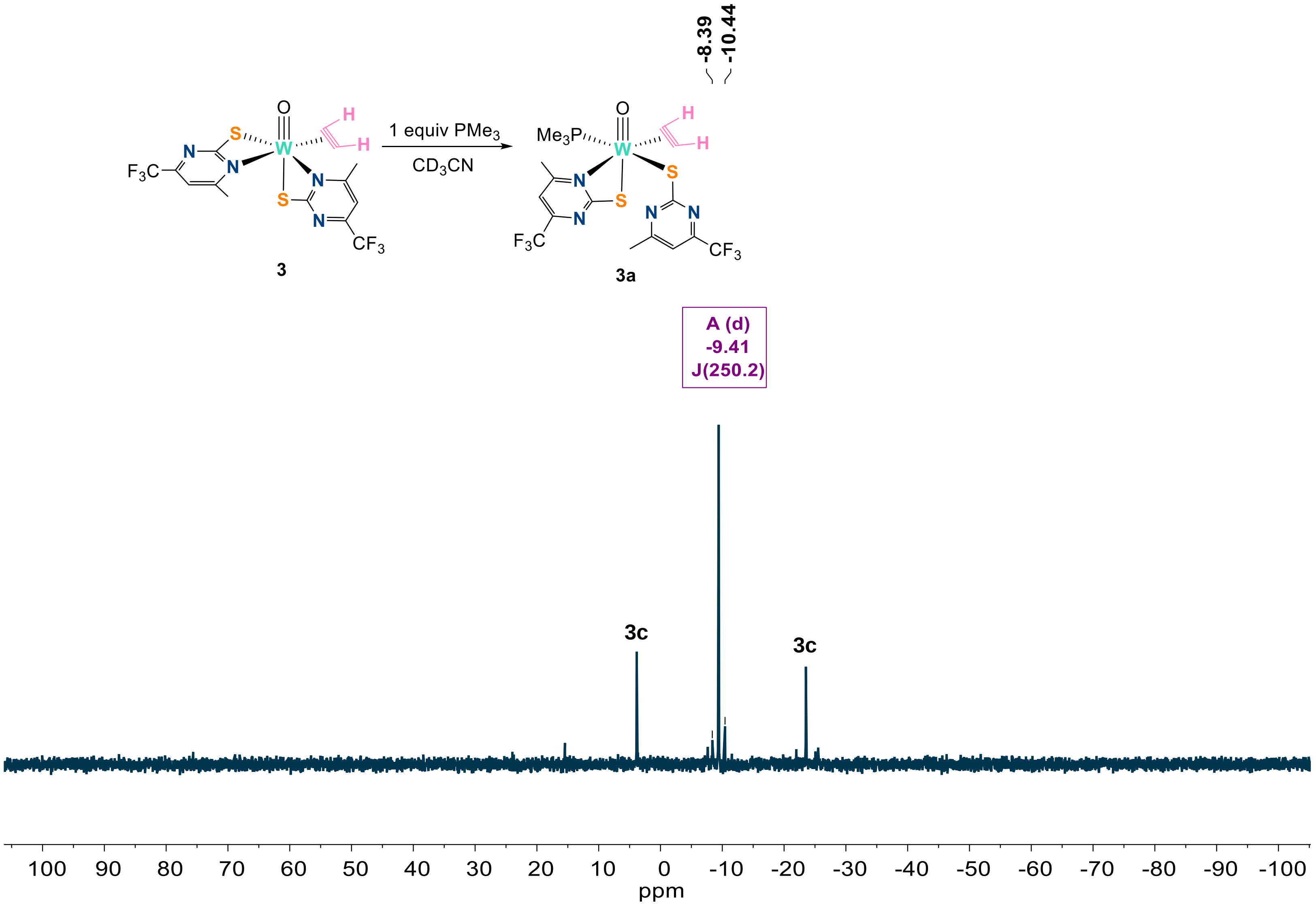

Figure S28. $^{31}P\{^{1}H\}$ NMR spectrum of the reaction of **3** with 1.1 equiv of PMe_3 in CD_3CN.

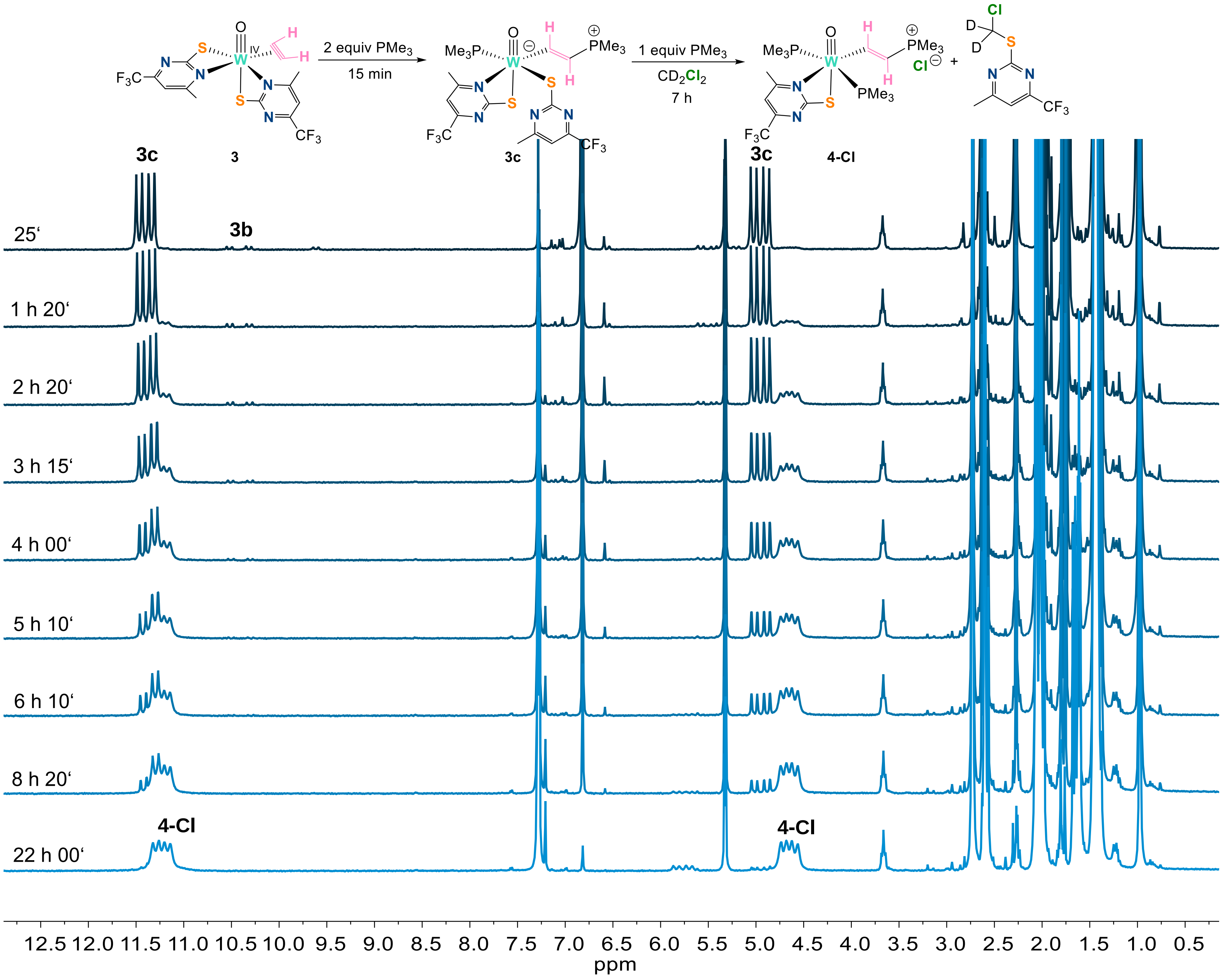

Figure S29. ^{1}H NMR spectra of the reaction of **3** with 3.3 equiv of PMe_3 in CD_2Cl_2.

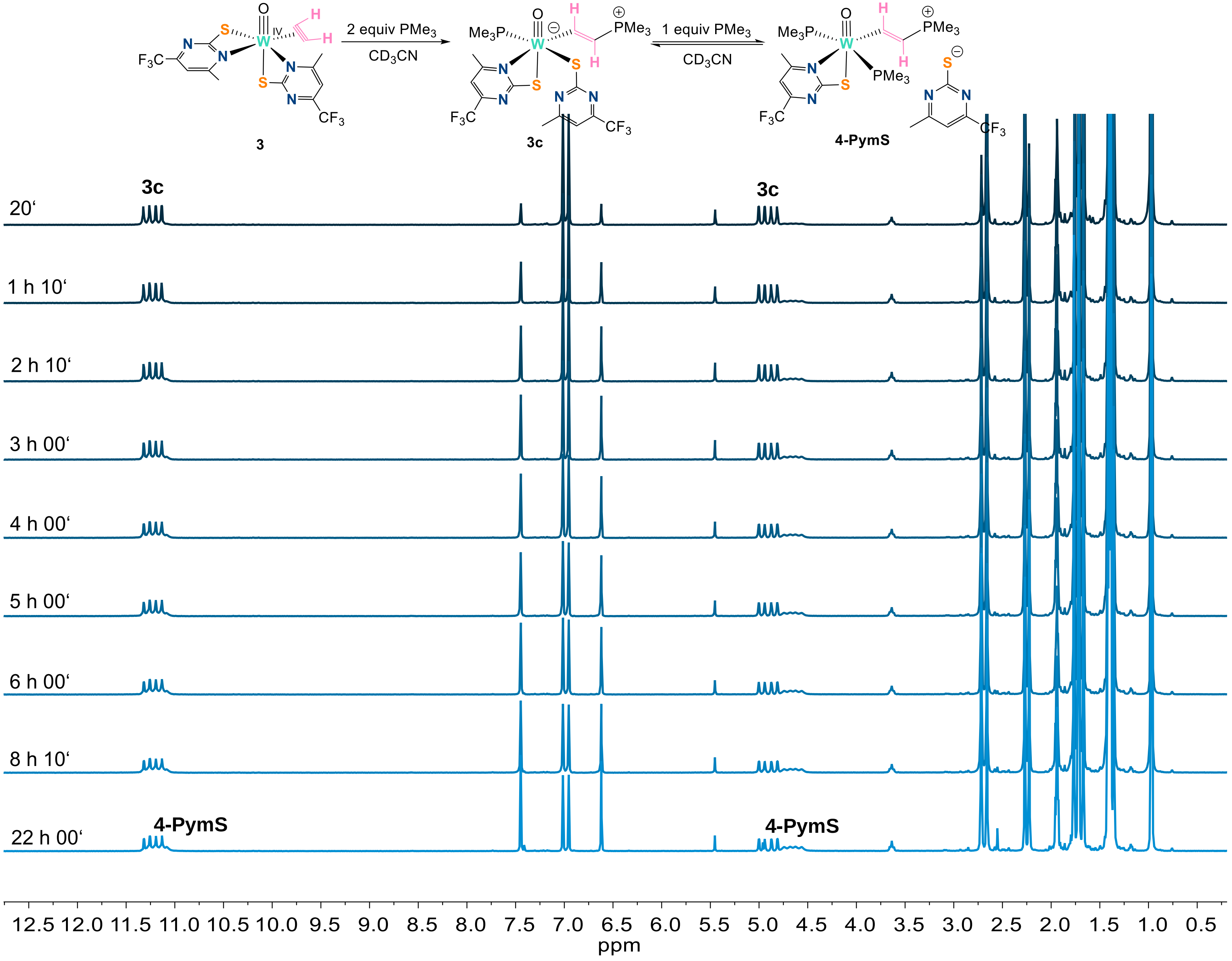

Figure S30. ¹H NMR spectra of the reaction of **3** with 3.3 equiv of PMe_3 in CD_3CN.

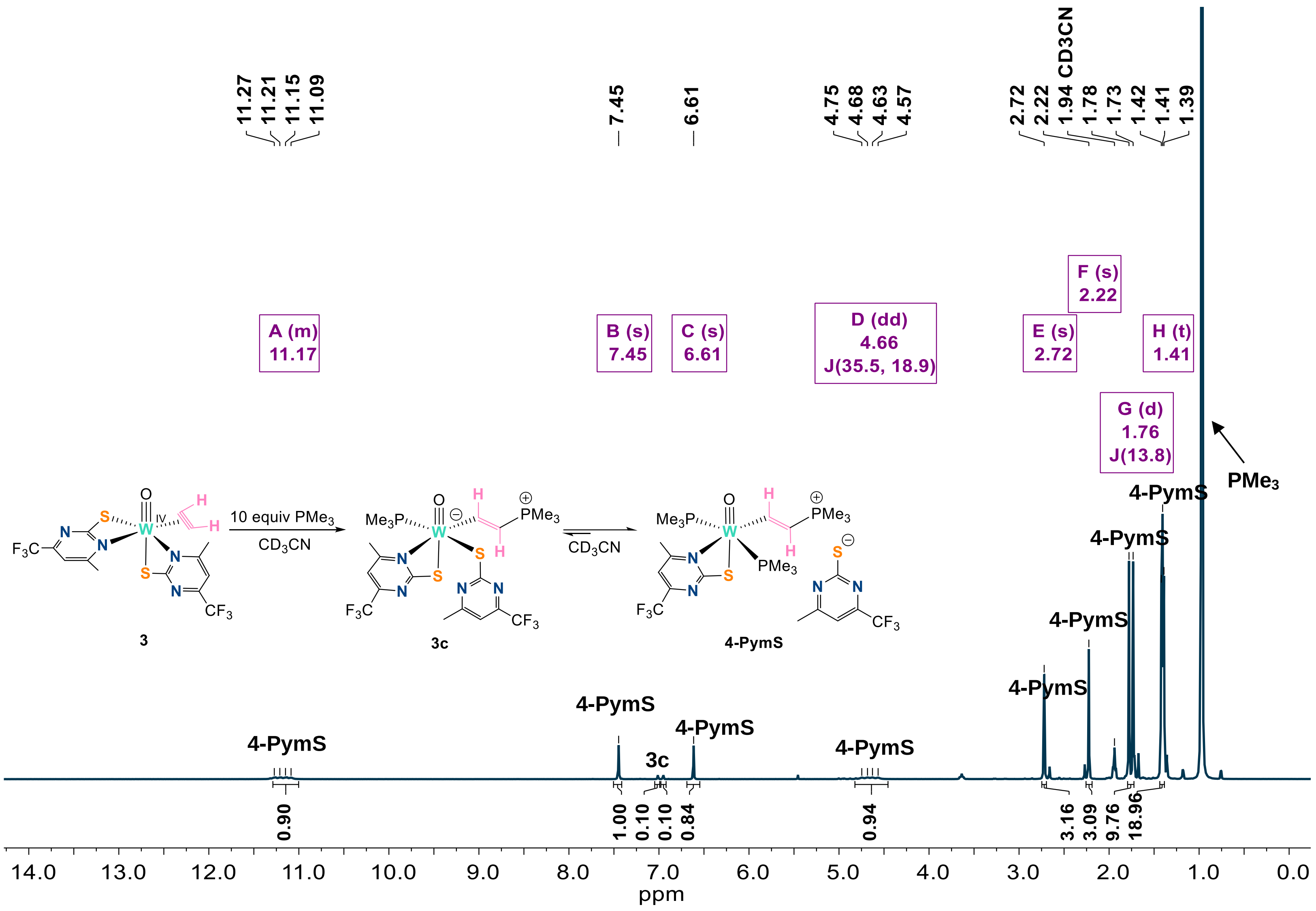

Figure S31. ^{1}H NMR spectra of the reaction of **3** with 10 equiv of PMe_3 in CD_3CN recorded after 4.5 h.

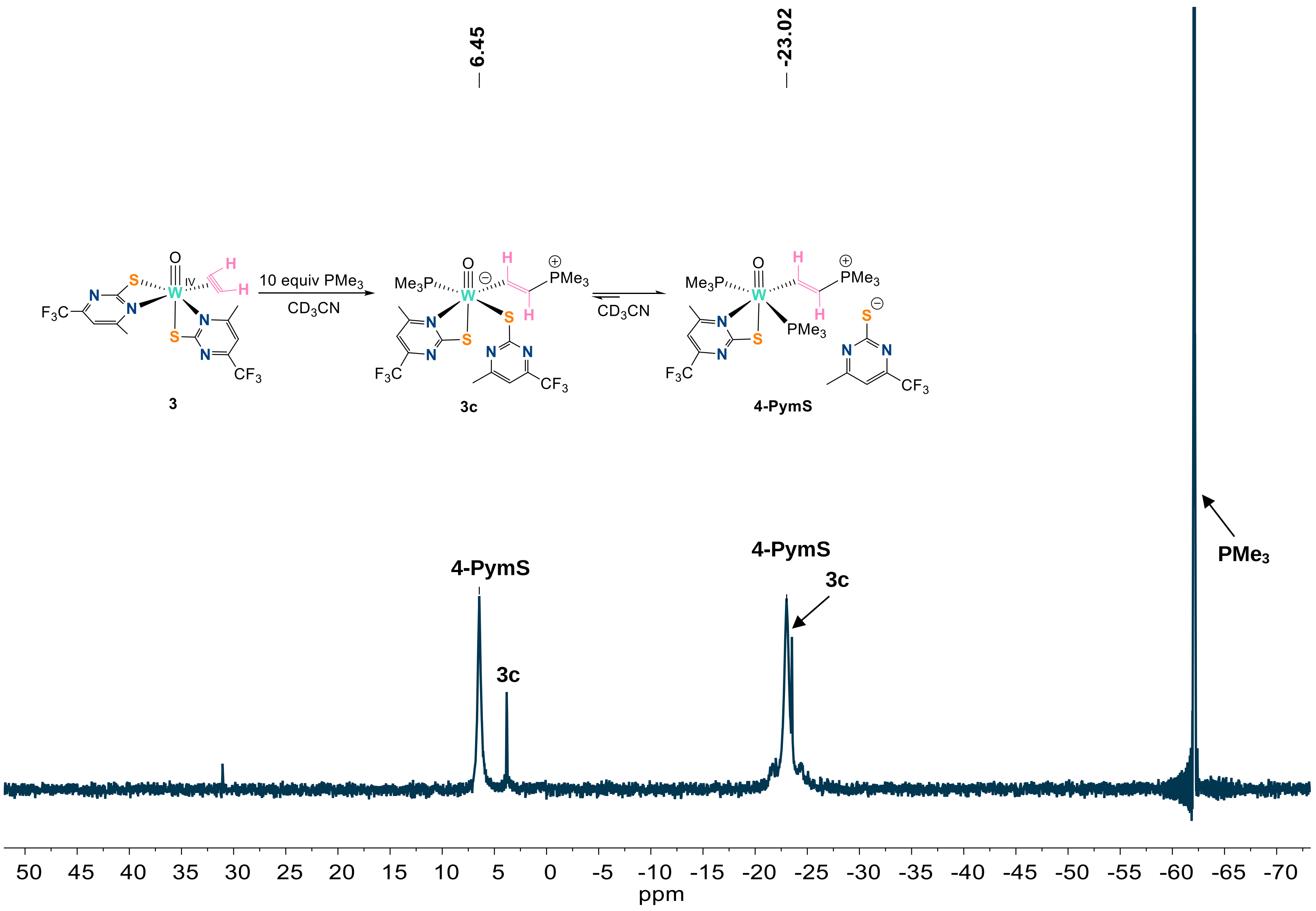

Figure S32. $^{31}P\{^{1}H\}$ NMR spectrum of the reaction of **3** with 10 equiv of PMe_3 in CD_3CN recorded after 4.5 h.

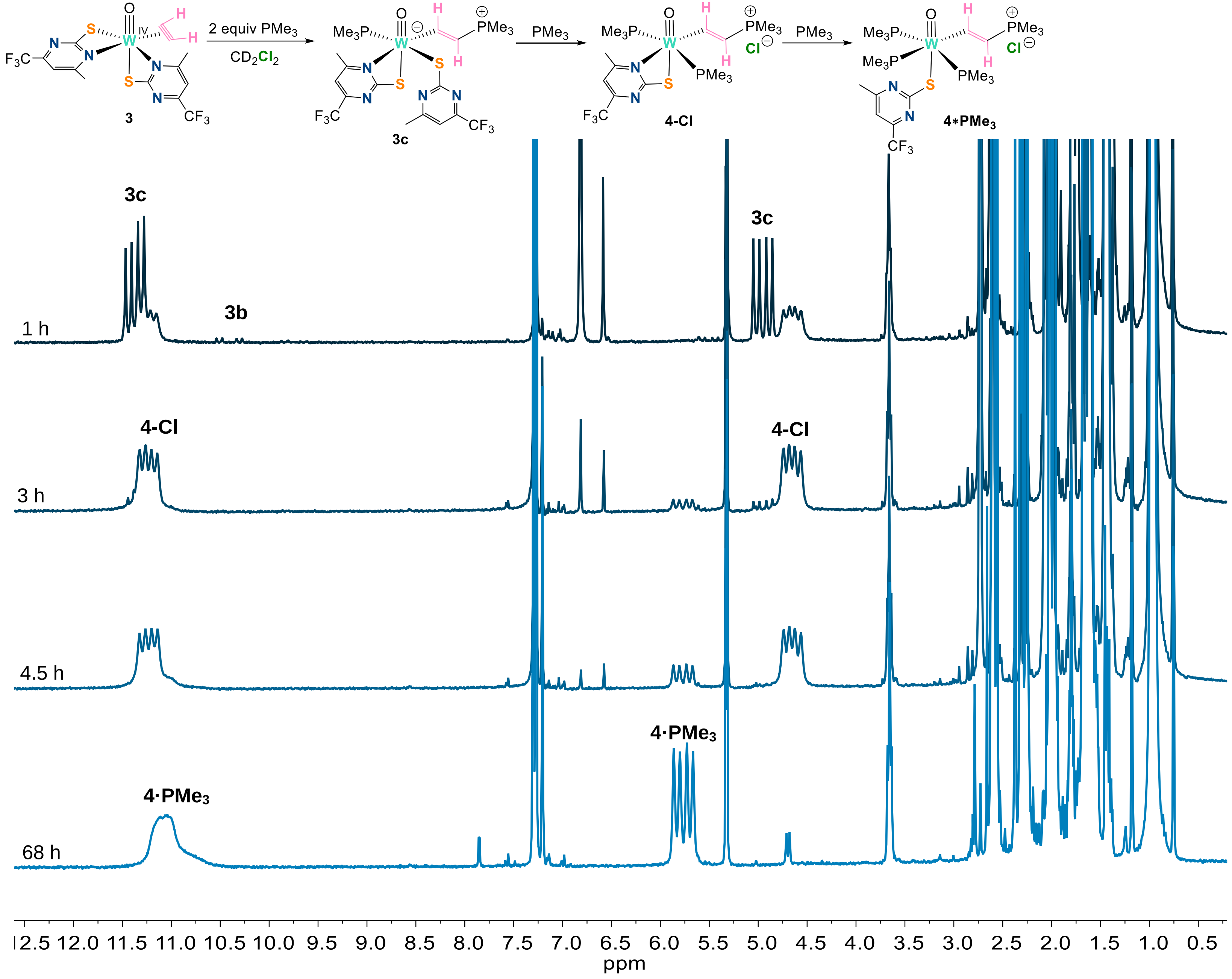

Figure S33. ^{1}H NMR spectra of the reaction of **3** with 10 equiv of PMe_3 in CD_2Cl_2.

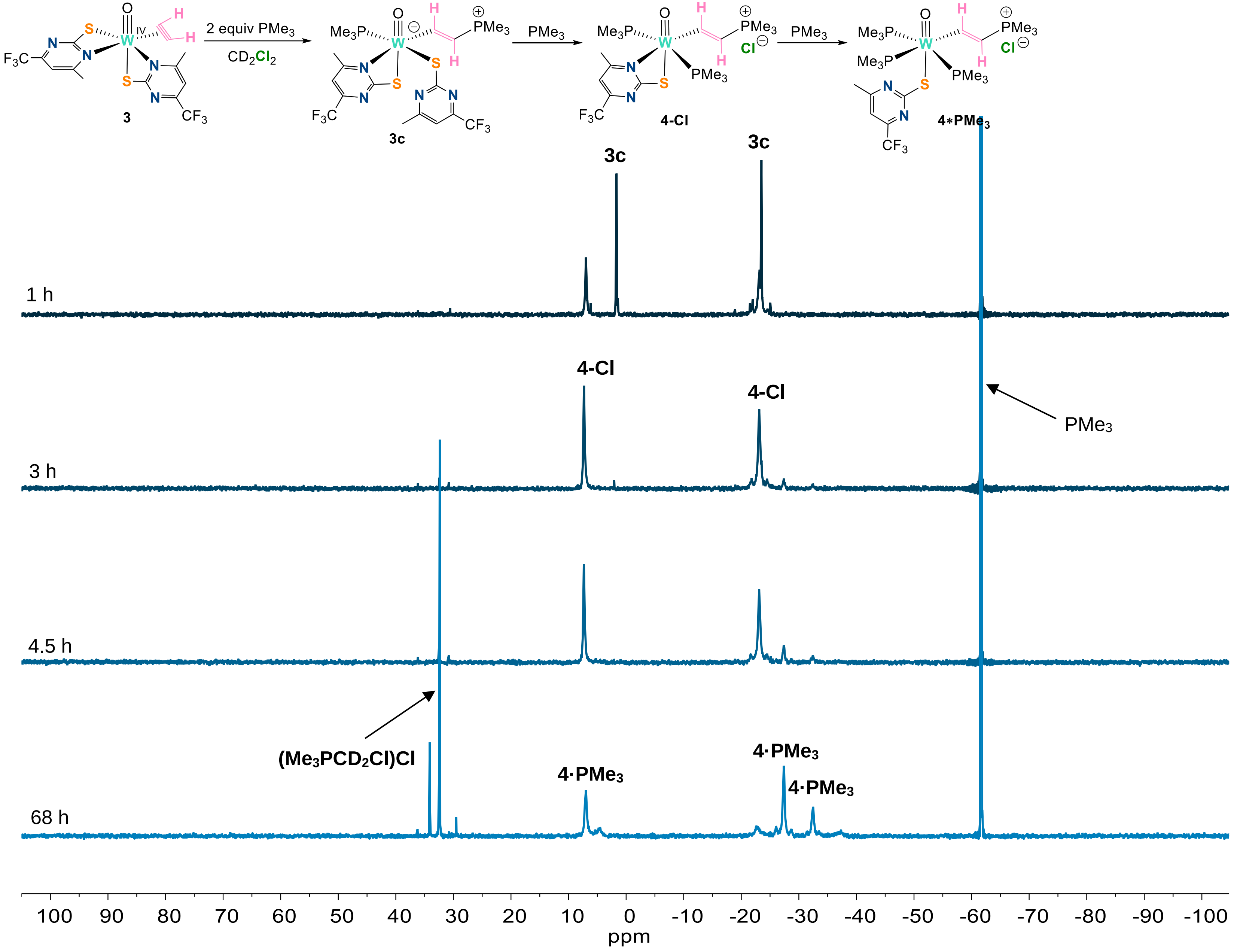

Figure S34. ${}^{31}P\{{}^{1}H\}$ NMR spectra of the reaction of **3** with 10 equiv of PMe_3 in CD_2Cl_2.

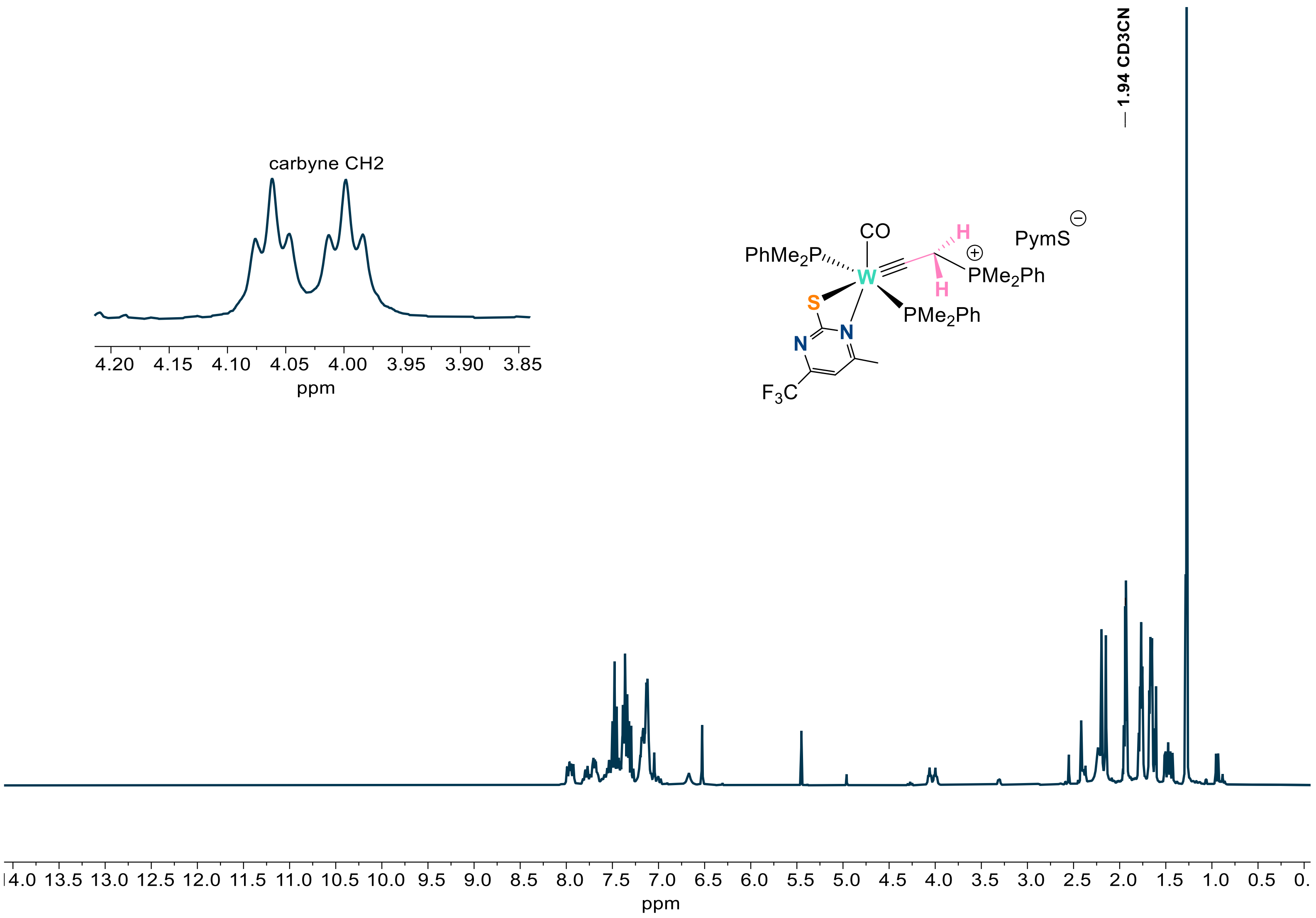

Figure S35. *In situ* ^{1}H NMR spectrum of the reaction of **1** with 4 equiv of PMe_2Ph in CD_3CN showing formation of the carbyne complex.

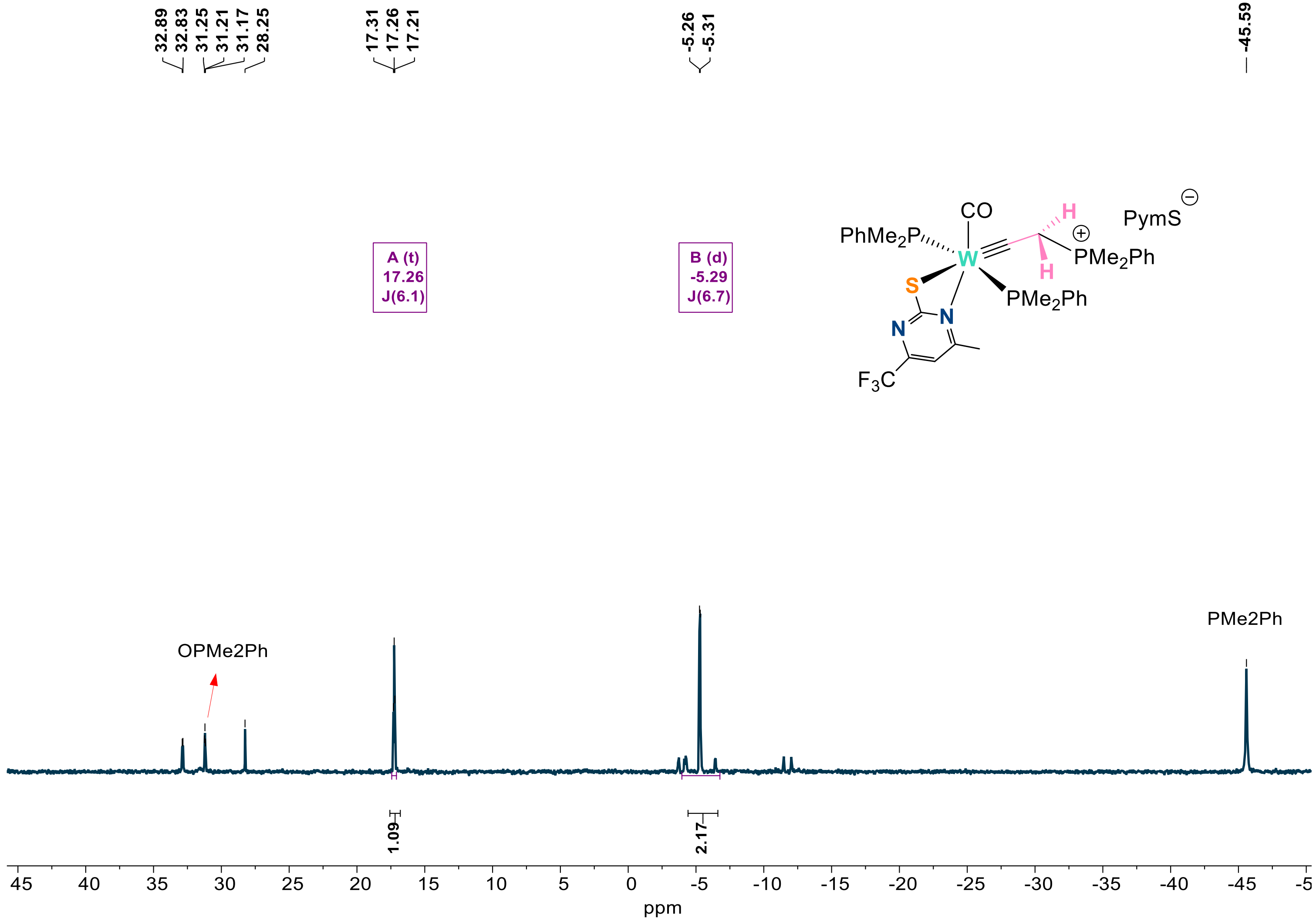

Figure S36. *In situ* $^{31}P\{^{1}H\}$ NMR spectrum of the reaction of **1** with 4 equiv of PMe_2Ph in CD_3CN confirming the formation of the depicted carbyne complex, free PMe_2Ph, $OPMe_2Ph$ and other undefined species.

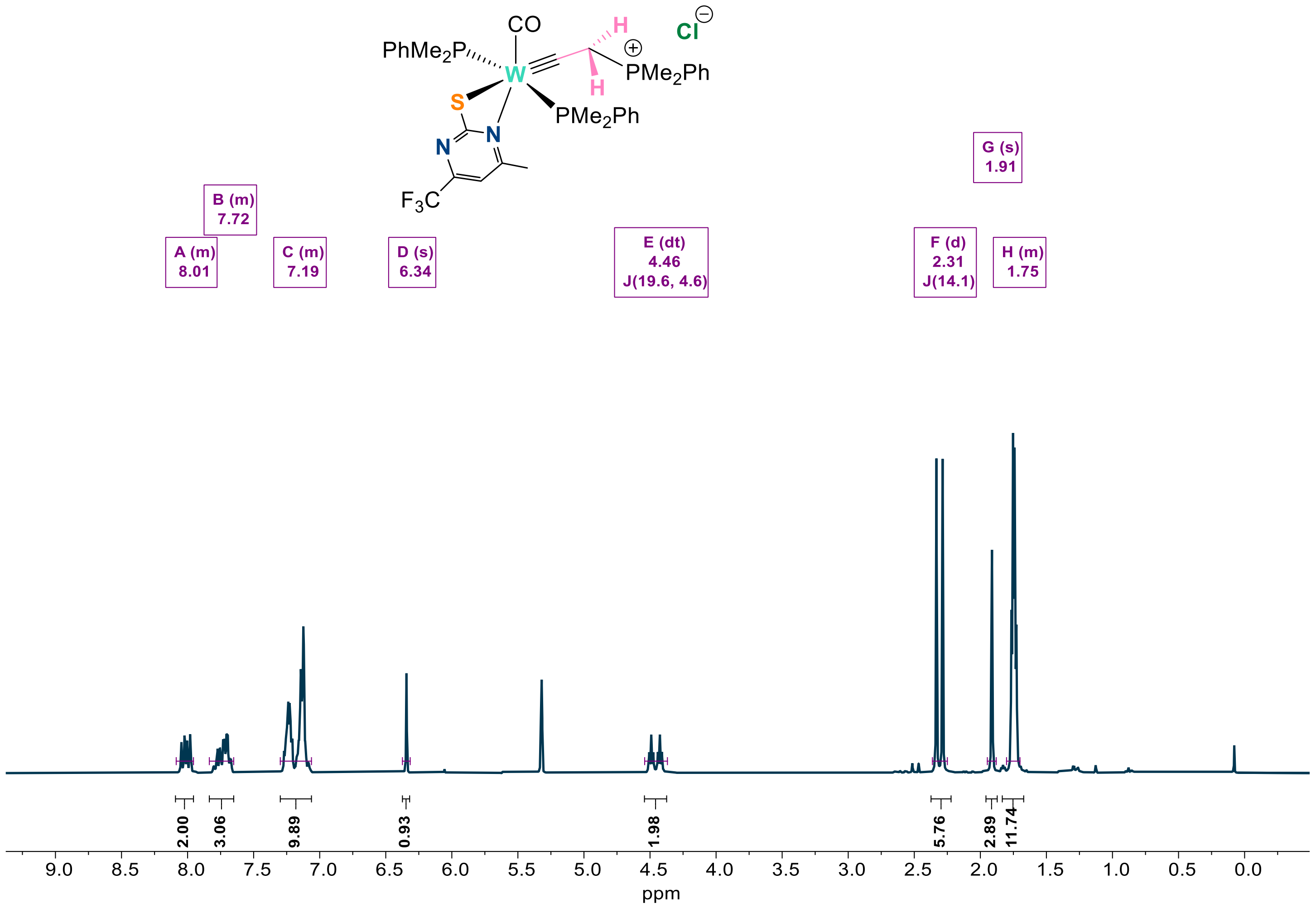

Figure S37. ^{1}H NMR spectrum of isolated [W(CO)(CCH$_2$PMe$_2$Ph)(PMe$_2$Ph)$_2$(PymS)]Cl in CD$_2$Cl$_2$.

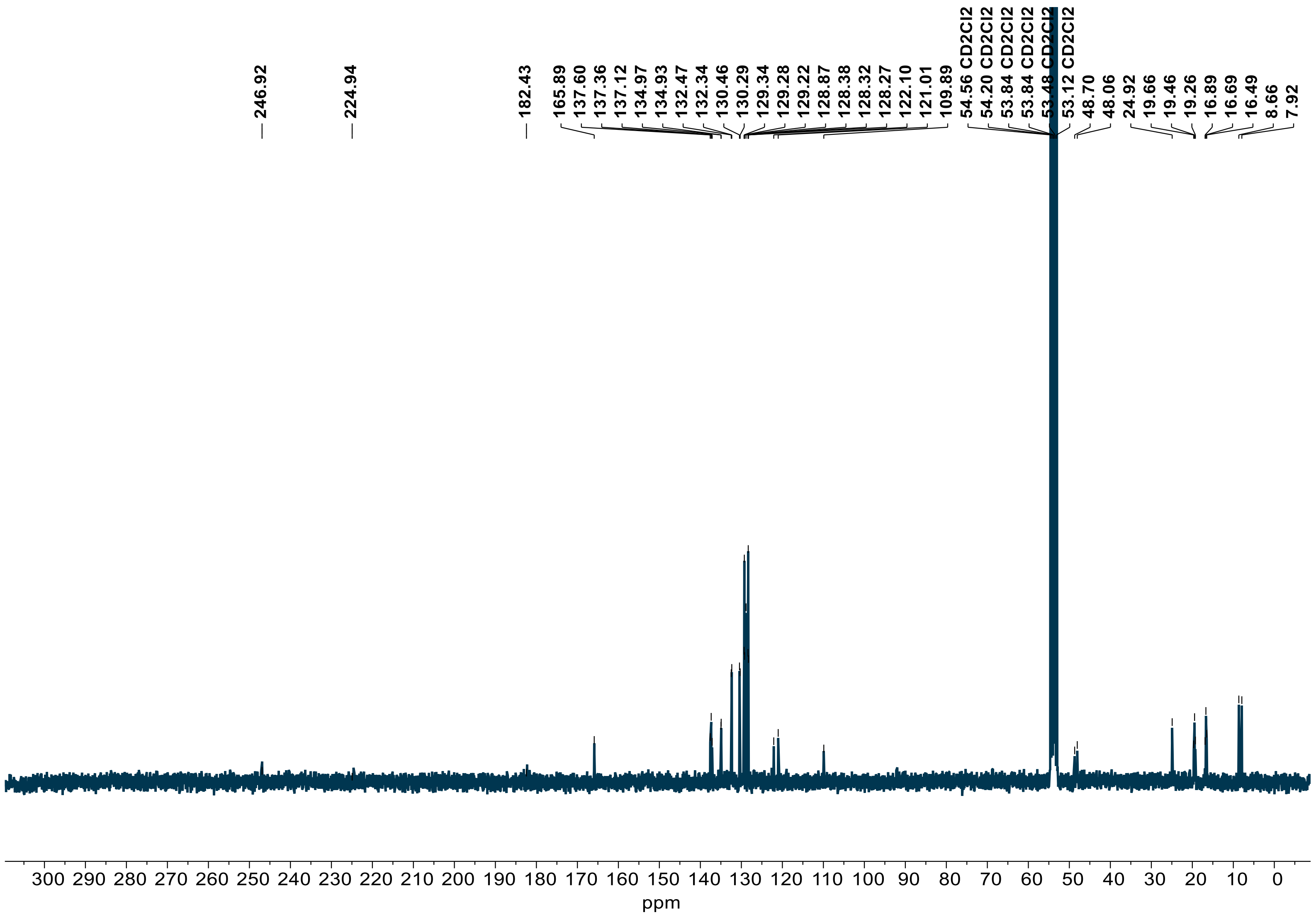

Figure S38. ^{13}C NMR spectrum of isolated [W(CO)(CCH$_2$PMe$_2$Ph)(PMe$_2$Ph)$_2$(PymS)]Cl in CD_2Cl_2.

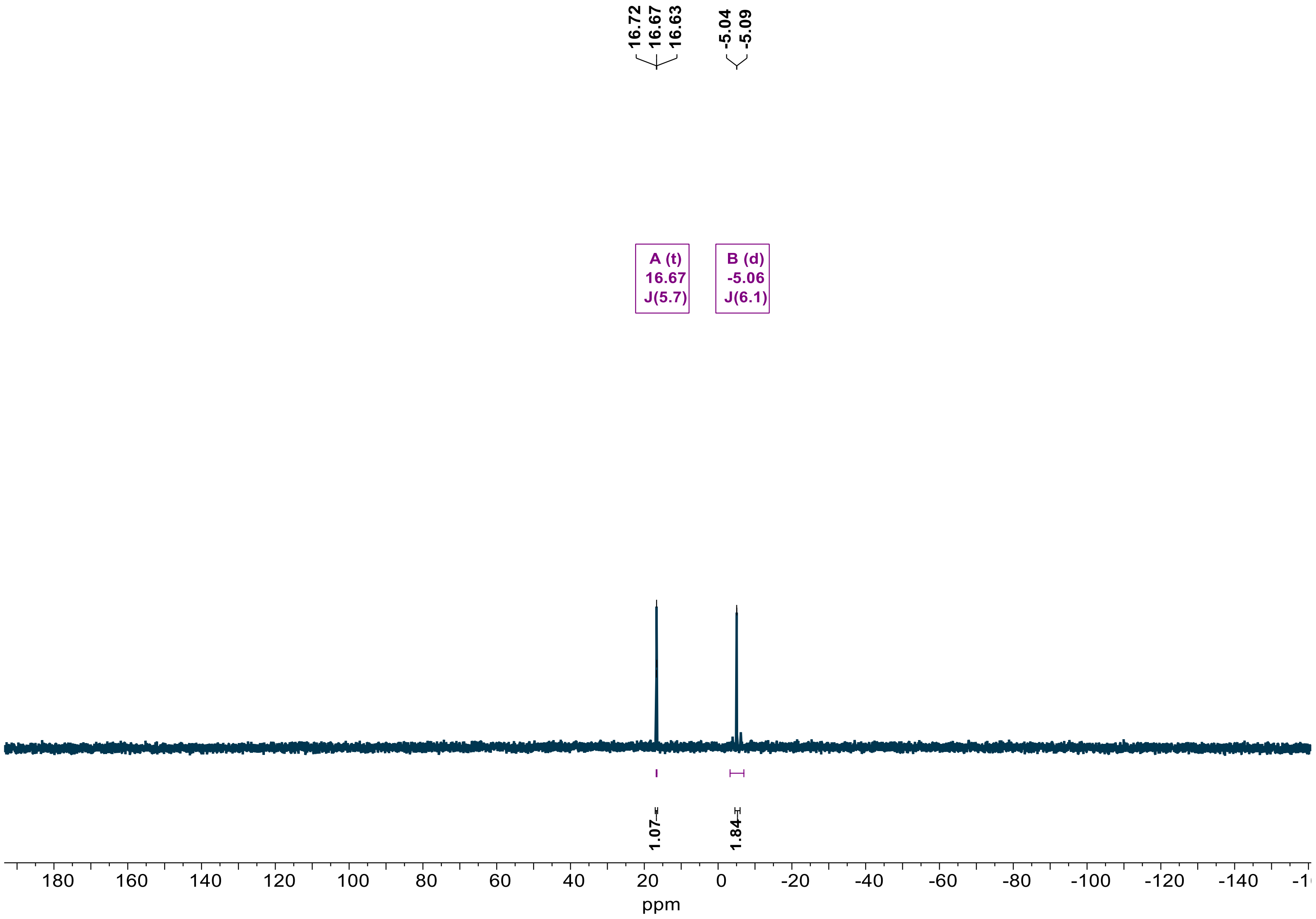

Figure S39. $^{31}P\{^{1}H\}$ NMR spectrum of isolated [W(CO)(CCH$_2$PMe$_2$Ph)(PMe$_2$Ph)$_2$(PymS)]Cl in CD_2Cl_2.

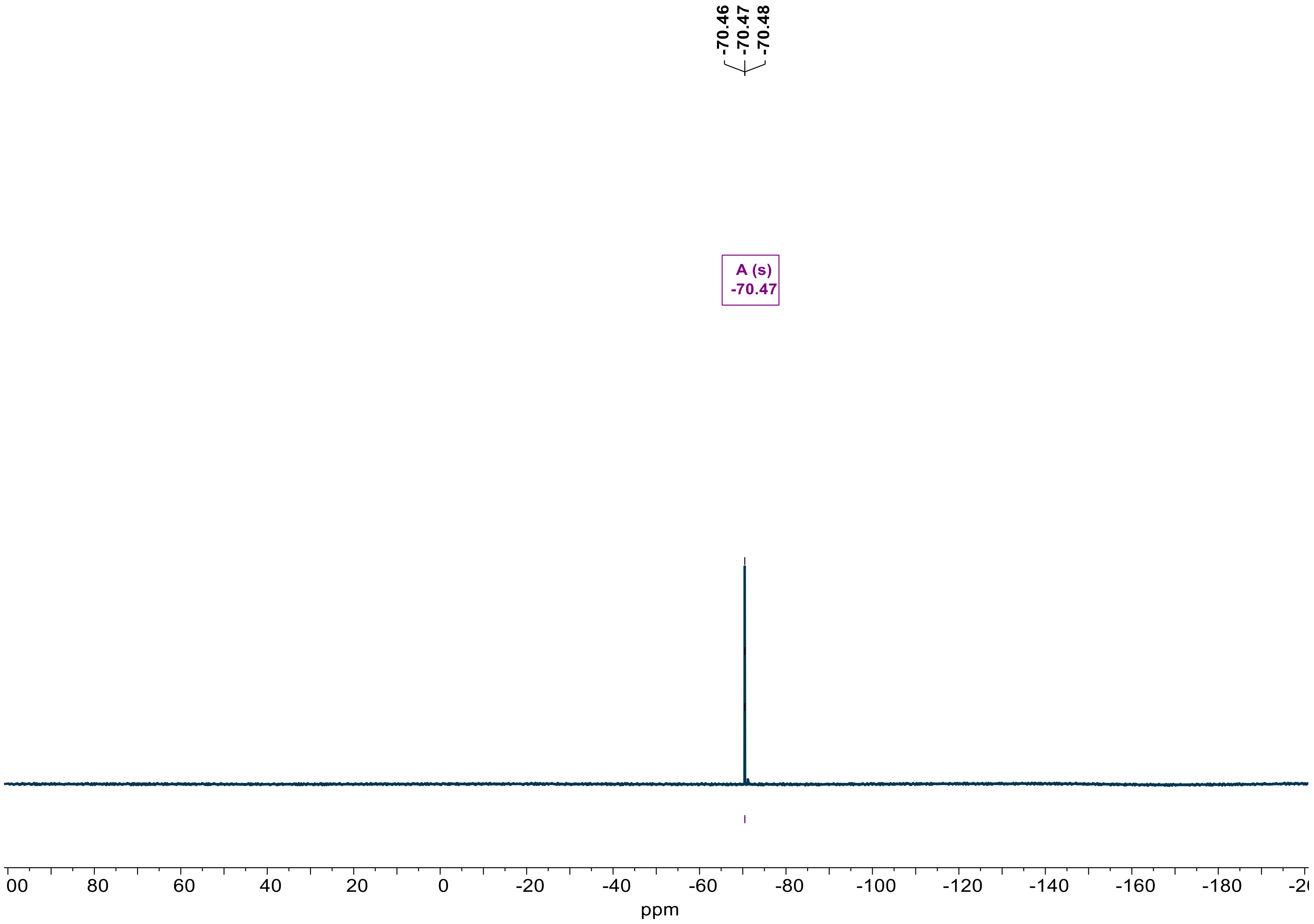

Figure S40. ^{19}F NMR spectrum of isolated [W(CO)(CCH$_2$PMe$_2$Ph)(PMe$_2$Ph)$_2$(PymS)]Cl in CD_2Cl_2.

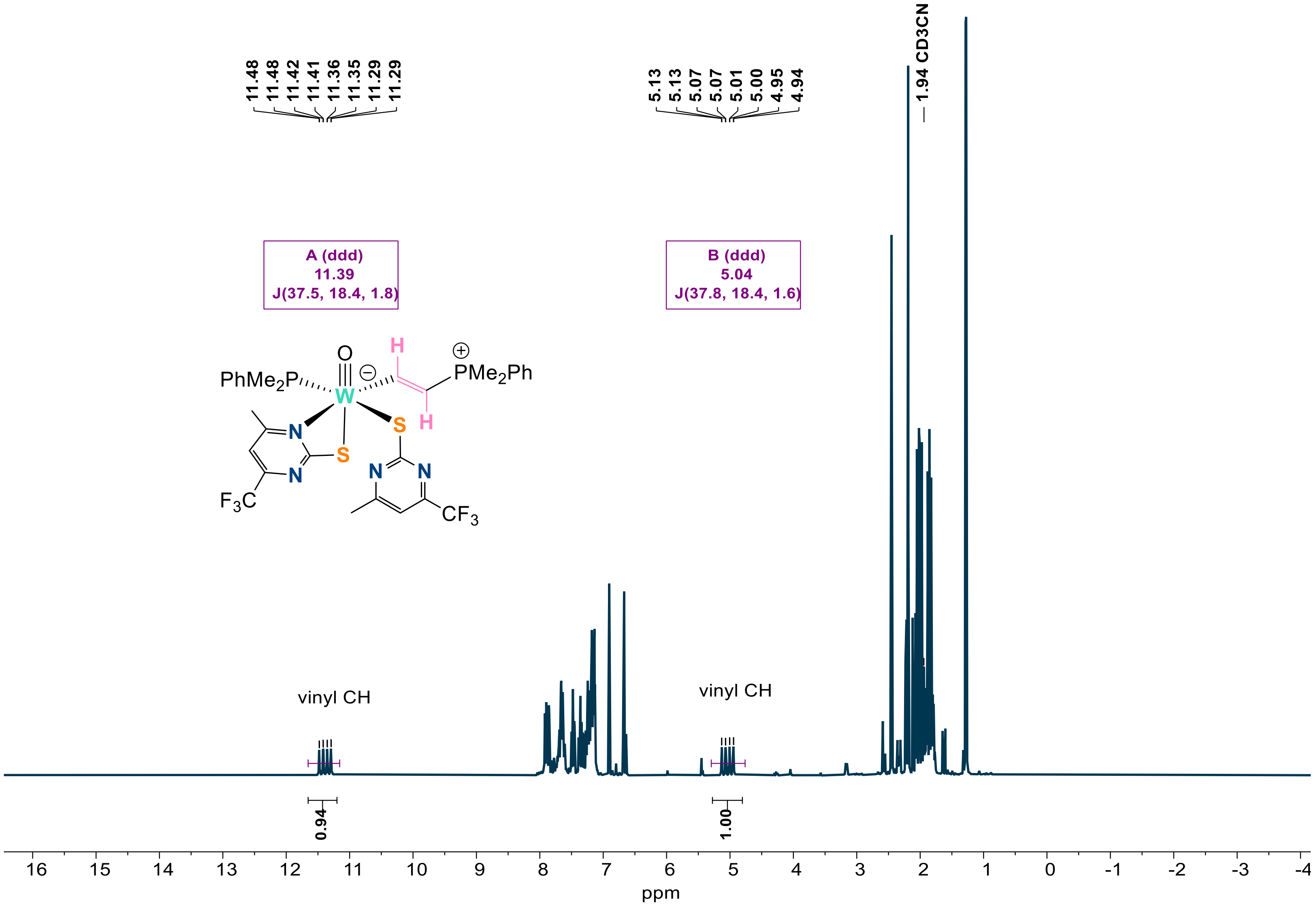

Figure S41. *In situ* ^{1}H NMR spectrum of the reaction of **3** with 4 equiv of PMe_2Ph in CD_3CN showing the formation of the η^1-vinyl compound.

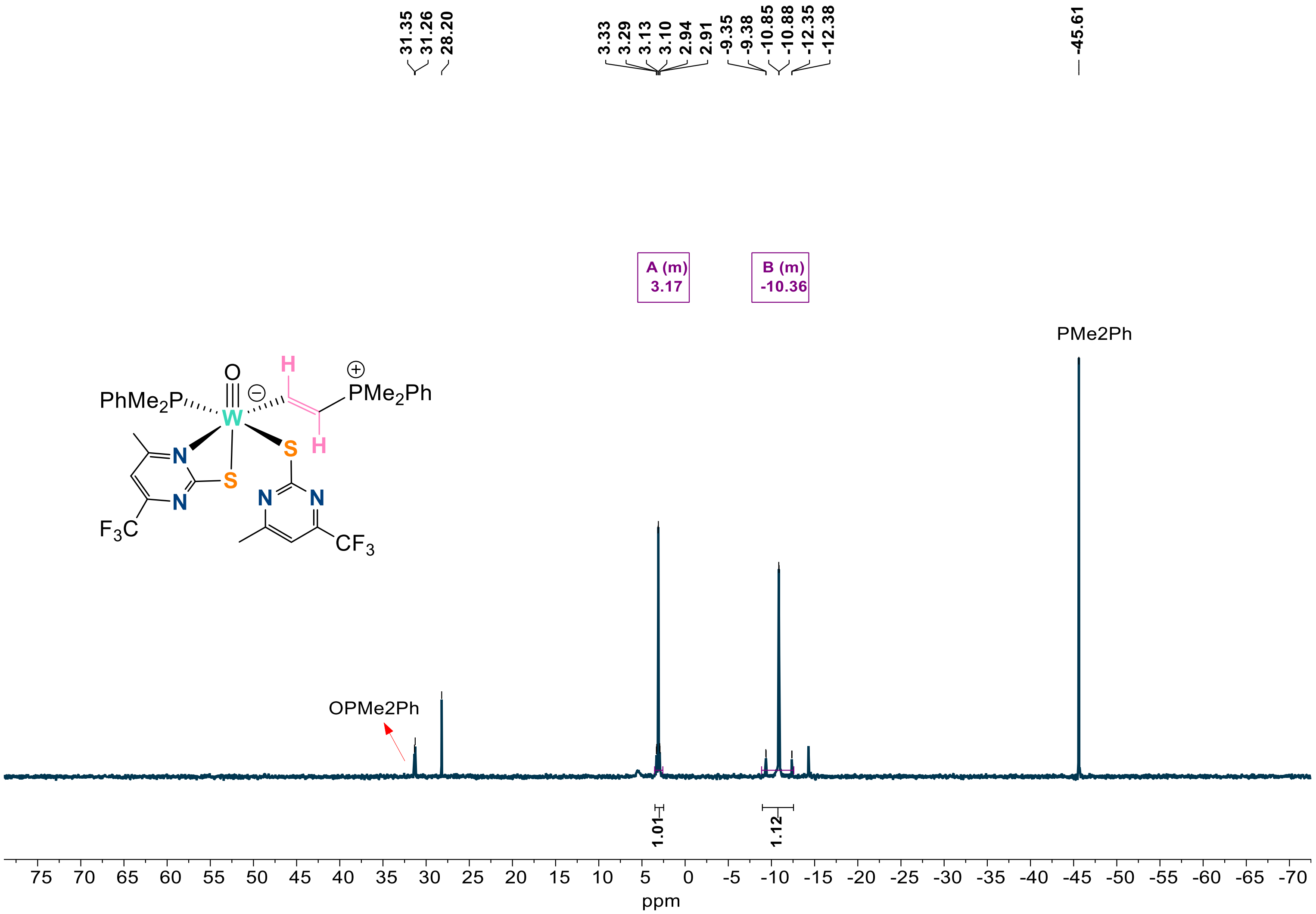

Figure S42. *In situ* $^{31}P\{^{1}H\}$ NMR spectrum of the reaction of **3** with 4 equiv of PMe_2Ph in CD_3CN showing the formation of the η^1-vinyl compound, free PMe_2Ph, $OPMe_2Ph$ and other undefined species.

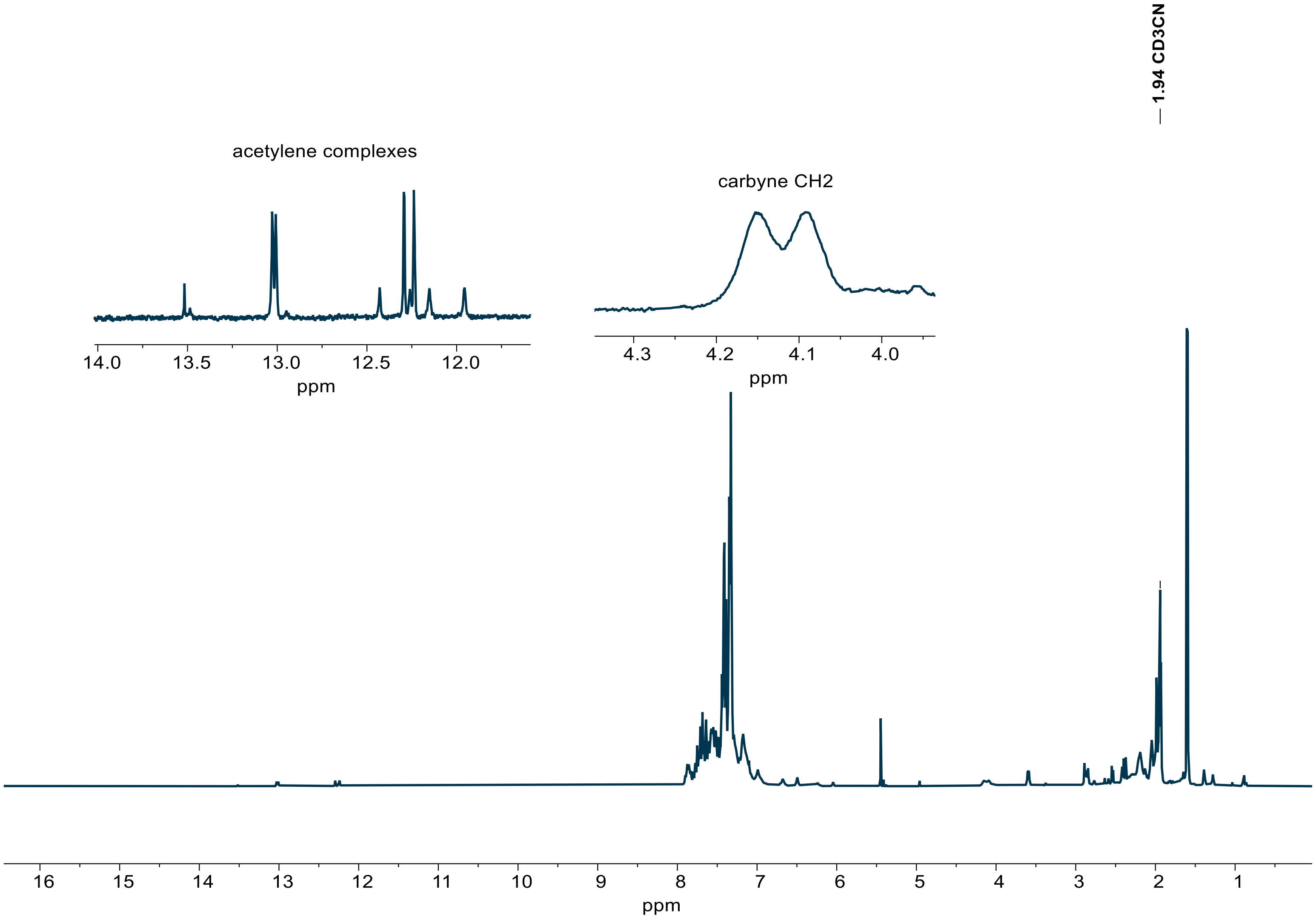

1.94 CD3CN
acetylene complexes
14.0
13.5
13.0
12.5
12.0
ppm
carbyne CH2
4.3
4.2
4.1
4.0
ppm
16
15
14
13
12
11
10
9
8
7
6
5
4
3
2
1
ppm

Figure S43. *In situ* ^{1}H NMR spectrum of the reaction of **1** with 4 equiv of $PMePh_2$ in CD_3CN showing several acetylene complexes and formation of the carbyne as a minor product.

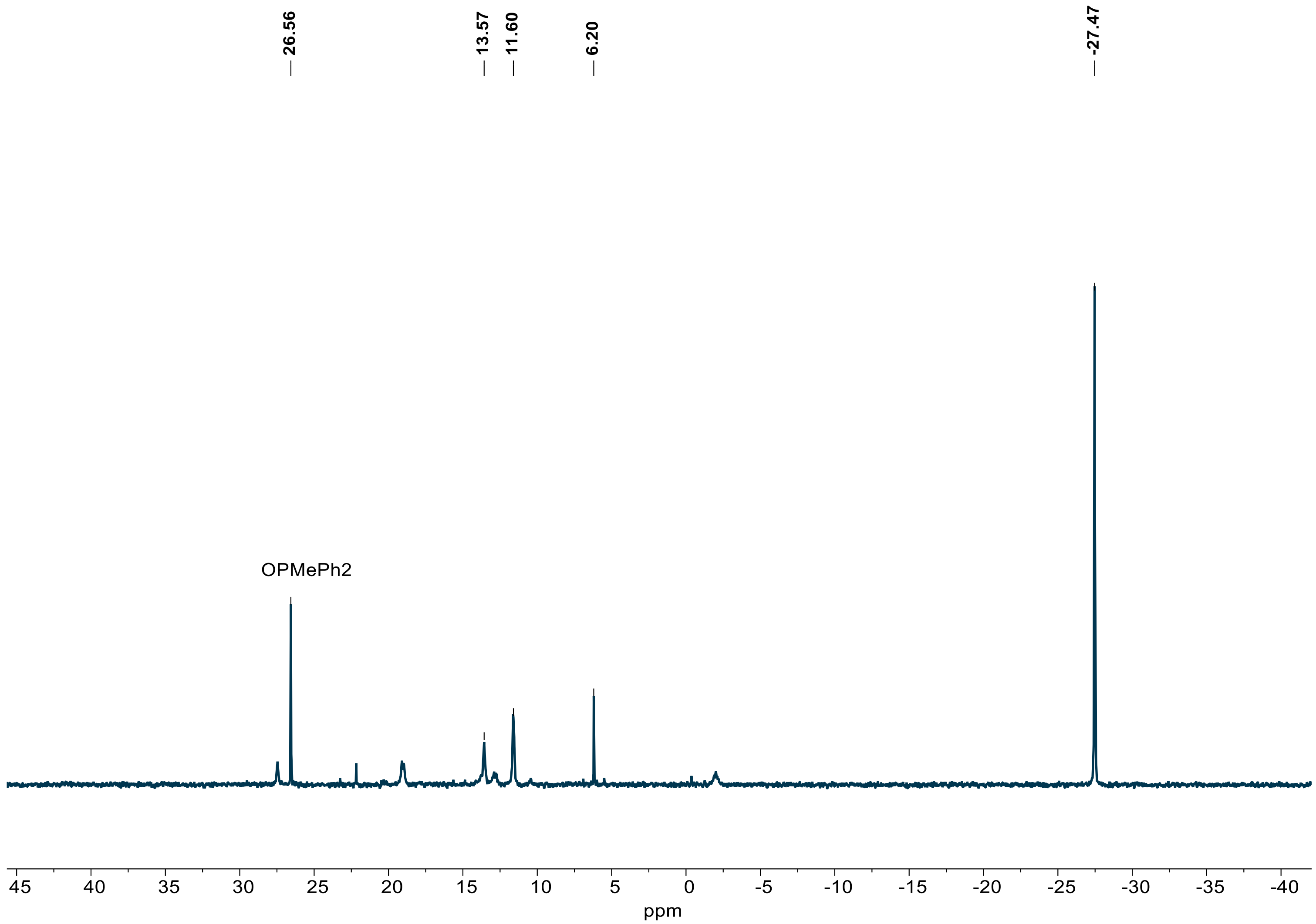

Figure S44. *In situ* $^{31}P\{^{1}H\}$ NMR spectrum of the reaction of **1** with 4 equiv of $PMePh_2$ in CD_3CN showing several undefined species, free $PMePh_2$ and $OPMePh_2$.

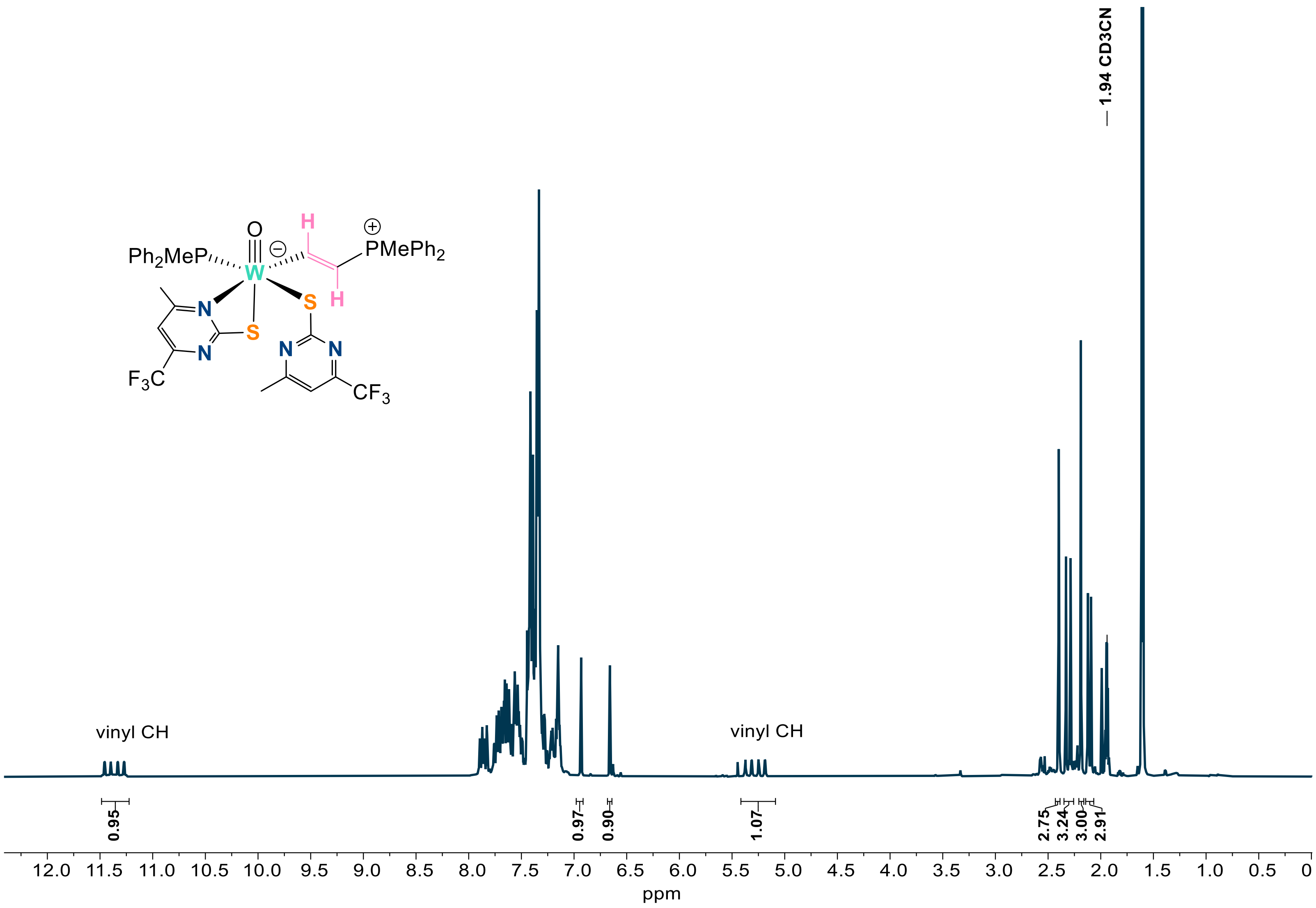

Figure S45. *In situ* ^{1}H NMR spectrum of the reaction of **3** with 4 equiv of $PMePh_2$ in CD_3CN confirming formation of the η^1-vinyl complex.

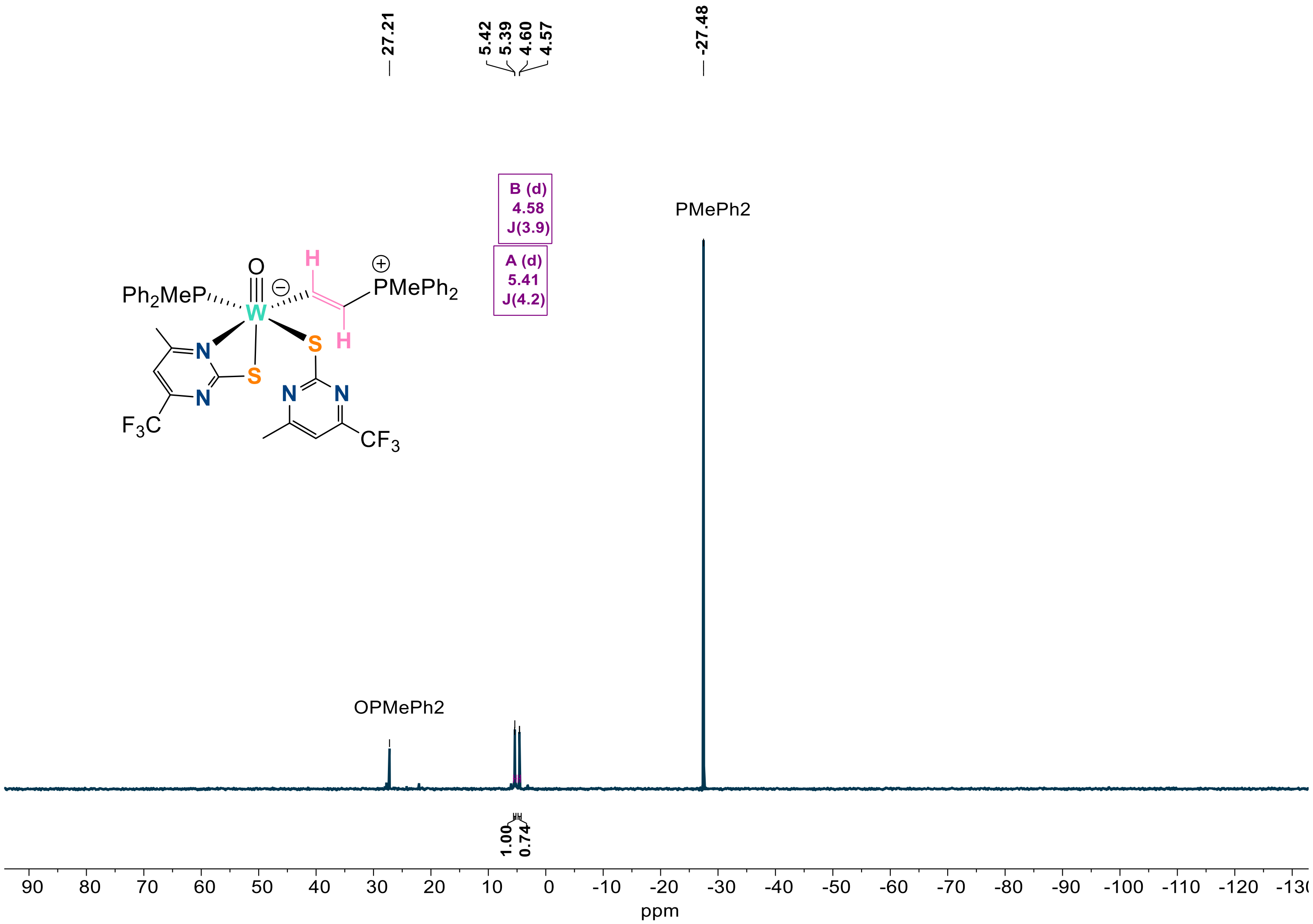

Figure S46. *In situ* $^{31}P\{^1H\}$ NMR spectrum of the reaction of **3** with 4 equiv of $PMePh_2$ in CD_3CN showing formation of the η^1-vinyl complex, free $PMePh_2$ and $OPMePh_2$.

8 X-Ray Crystallographic Data

Crystal Structure Determination–General. All the single crystal measurements were performed on a Bruker APEX-II CCD diffractometer at 100 K using Mo K_α radiation with a wavelength of 0.71073Å from an Incoatec microfocus sealed tube equipped with a multilayer monochromator. Absorption corrections were made semi-empirically from equivalents. The structures were solved by direct methods (SHELXS-97)[32] and refined by full-matrix least-squares techniques against F^2 (SHELXL-2014/6)[33]. A weighting scheme of $w = 1/[\sigma^2(F_o^2)+(aP)^2+bP]$ where $P = (F_o^2+2F_c^2)/3$ was used. The non-hydrogen atoms of the metal complexes were refined with anisotropic displacement parameters without any constraints. Except for compound **2** (vide infra), the positions of the H atoms of the ethyne ligands and of the ethenyl group were taken from a difference Fourier map, the C–H distances were fixed to 0.95 Å, and the H atoms were refined with a common isotropic displacement parameter without any constraints to the bond angles. The H atoms of the CH_2 groups were refined with common isotropic displacement parameters for the H atoms of the same group and idealized geometry with approximately tetrahedral angles and C–H distances of 0.99 Å. The H atoms of the methyl groups were refined with common isotropic displacement parameters for the H atoms of the same group and idealized geometries with tetrahedral angles, enabling rotations around the C–C bonds, and C–H distances of 0.98 Å. The H atoms of the aromatic rings were put at the external bisectors of the C–C–C angles at C–H distances of 0.95 Å and common isotropic displacement parameters were refined for the H atoms of the same ring. Crystal data, data collection parameters and structure refinement details are given in Tables S4–S8. Further refinement information, structure and bonding parameters, SHELXL .res and .hkl files are given in the deposited CIF file which is available free of charge from The Cambridge Crystallographic Data Centre (CCDC **2294570-2294573**).

Crystal Structure Determination of 3. Since twinning was detected a twin matrix (1 0 1 / 0 -1 0 / 0 0 -1) was applied and a scale factor was refined [0.1612(5)] between the two unequal components lowering R1 from 0.1415 to 0.0488. The H atoms of the ethyne ligands were put at the external bisectors of the W–C–C angles at C–H distances of 0.95 Å and one common isotropic displacement parameter was refined for the H atoms of the same ligand.

Table S4. Crystallographic data and structure refinement details of **1** and **2-Cl**

**Crystal data**	**1**	**2-Cl**
CIF data code	ME233	ME296
Empirical formula	$C_{15}H_{10}F_6N_4OS_2W$	$C_{18}H_{33}F_3N_2OP_3SW^{+}Cl^{-} \cdot CH_2Cl_2$
Formula weight	624.24	779.66
Crystal description	block, yellow	block, orange
Crystal size	0.42 x 0.39 x 0.37 mm	0.18 x 0.15 x 0.12 mm
Temperature	100 K	100 K
Crystal system	monoclinic	orthorhombic
Space group	P 2_1/c	P b c a
a	11.1341(6) Å	12.5006(6) Å
b	11.4816(6) Å	16.8432(8) Å
c	14.6617(9) Å	28.6716(12) Å
β	105.348(2)°	
Volume	1807.47(18) $Å^3$	6036.8(5) $Å^3$
Z	4	8
Calc. density	2.294 Mg/m^3	1.716 Mg/m^3
F(000)	1184	3072
Linear absorption coefficient μ	6.697 mm^{-1}	4.355 mm^{-1}
Max. and min. transmission	0.746 and 0.121	0.746 and 0.514
Unit cell determination	2.29° < Θ < 29.93°	2.16° < Θ < 28.71°
Reflections used	9003	9896
**Data collection**		
Θ range for data collection	1.90 to 27.00°	2.15 to 28.00°
Reflections collected / unique	38741 / 3941	218014 / 7298
Significant unique reflections	3626 with I > 2σ(I)	5518 with I > 2σ(I)
R(int), R(sigma)	0.0868, 0.0425	0.1193, 0.0503
Completeness to Θ_{max}	99.8%	100.0%
**Refinement**		
Data / parameters / restraints	3941 / 274 / 2	7298 / 321 / 0
Goodness-of-fit on F^2	1.089	1.042
Final R indices [I > 2σ(I)]	R1 = 0.0318, wR2 = 0.0794	R1 = 0.0286, wR2 = 0.0462
R indices (all data)	R1 = 0.0349, wR2 = 0.0807	R1 = 0.0508, wR2 = 0.0501
Weighting scheme param. a, b	0.0412, 2.6503	0.0000, 2.8226
Largest Δ/σ in last cycle	0.002	0.002
Largest diff. peak and hole	2.287 and -2.457 e/$Å^3$	0.830 and -1.021 e/$Å^3$
**CCDC no.**	**2294570**	**2294571**

Table S5. Crystallographic data and structure refinement details of **3** and **4-Cl**

**Crystal data**	**3**	**4-Cl**
CIF data code	ME290B	ME293
Empirical formula	$C_{14}H_{10}F_6N_4OS_2W$	$C_{17}H_{33}F_3N_2OP_3SW^+Cl^-$
Formula weight	612.23	682.72
Crystal description	block, yellow	block, purple
Crystal size	0.12 x 0.09 x 0.08 mm	0.13 x 0.11 x 0.08 mm
Temperature	100 K	100 K
Crystal system	monoclinic	orthorhombic
Space group	P 2_1/c	P b c a
a	22.109(2) Å	11.9724(9) Å
b	11.4589(12) Å	19.1556(15) Å
c	15.5638(15) Å	24.266(2) Å
β	110.286(5)°	
Volume	3698.5(7) Å^3	5565.2(8) Å^3
Z	8	8
Calc. density	2.199 Mg/m^3	1.630 Mg/m^3
F(000)	2320	2688
Linear absorption coefficient μ	6.543 mm^{-1}	4.526 mm^{-1}
Max. and min. transmission	0.745 and 0.445	0.746 and 0.509
Unit cell determination	2.66° < Θ < 25.00°	2.71° < Θ < 30.00°
Reflections used	6330	9713
**Data collection**		
Θ range for data collection	0.98 to 29.00°	2.17 to 30.00°
Reflections collected / unique	87643 / 9777	225980 / 8102
Significant unique reflections	7416 with I > 2σ(I)	6022 with I > 2σ(I)
R(int), R(sigma)	0.1091, 0.0962	0.0892, 0.0568
Completeness to Θ_{max}	99.4%	100.0%
**Refinement**		
Data / parameters / restraints	9777 / 517 / 0	8102 / 290 / 2
Goodness-of-fit on F^2	1.045	1.129
Final R indices [I > 2σ(I)]	R1 = 0.0488, wR2 = 0.0780	R1 = 0.0388, wR2 = 0.0646
R indices (all data)	R1 = 0.0781, wR2 = 0.0881	R1 = 0.0653, wR2 = 0.0726
Weighting scheme param. a, b	0.0000, 4.1110	0.0168, 4.6749
Largest Δ/σ in last cycle	0.001	0.002
Largest diff. peak and hole	1.454 and -1.681 e/Å^3	2.087 and -1.747 e/Å^3
**CCDC no.**	**2294572**	**2294573**

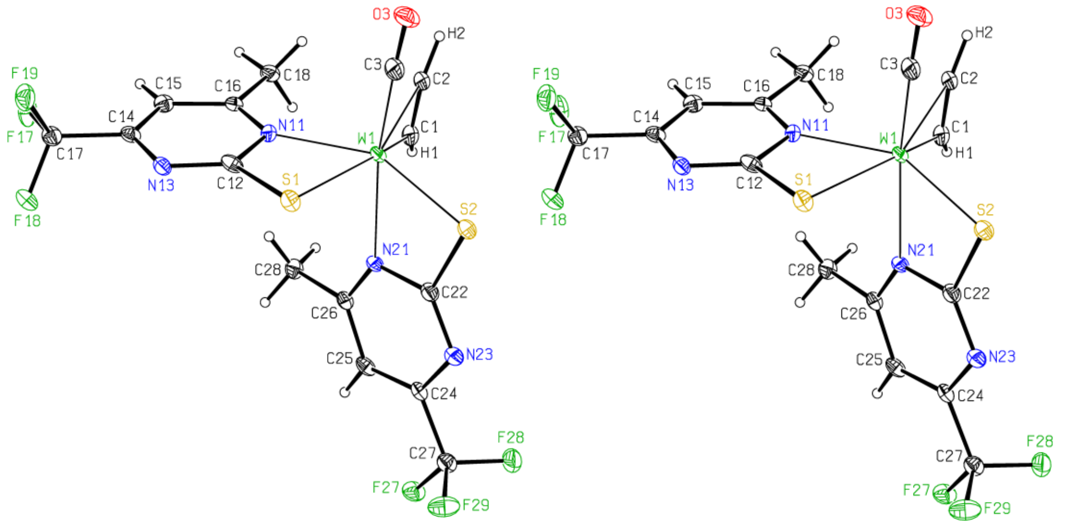

Figure S47. Stereoscopic ORTEP[9] plot of **1** showing the atomic numbering scheme. The probability ellipsoids are drawn at the 50% probability level. The H atoms are drawn with arbitrary radii.

Table S6. Selected bond lengths [Å] and angles [°] for **1**

W1–C1	2.017(4)	S2–W1–S1	86.10(4)
W1–C2	2.040(4)	C2–C1–H1	142.6(15)
W1–C3	1.983(5)	W1–C1–H1	145.1(14)
W1–N11	2.202(4)	C1–C2–H2	146.6(11)
W1–N21	2.257(3)	W1–C2–H2	143.3(11)
W1–S1	2.5842(11)	O3–C3–W1	177.5(4)
W1–S2	2.4001(12)	C12–S1–W1	81.03(15)
C1–C2	1.315(5)	C12–N11–C16	118.7(4)
C3–O3	1.146(6)	C12–N11–W1	104.0(3)
S1–C12	1.719(5)	C16–N11–W1	137.3(3)
S2–C22	1.747(5)	C22–S2–W1	84.21(15)
		C22–N21–C26	118.6(4)
C1–W1–S1	157.15(12)	C22–N21–W1	99.2(3)
C2–W1–S1	153.50(13)	C26–N21–W1	141.9(3)
N11–W1–S2	147.47(10)		
C3–W1–N21	161.12(18)	C1–C2–W1–C3	-179.9(3)
N11–W1–N21	95.74(13)	C2–C1–W1–C3	0.1(3)

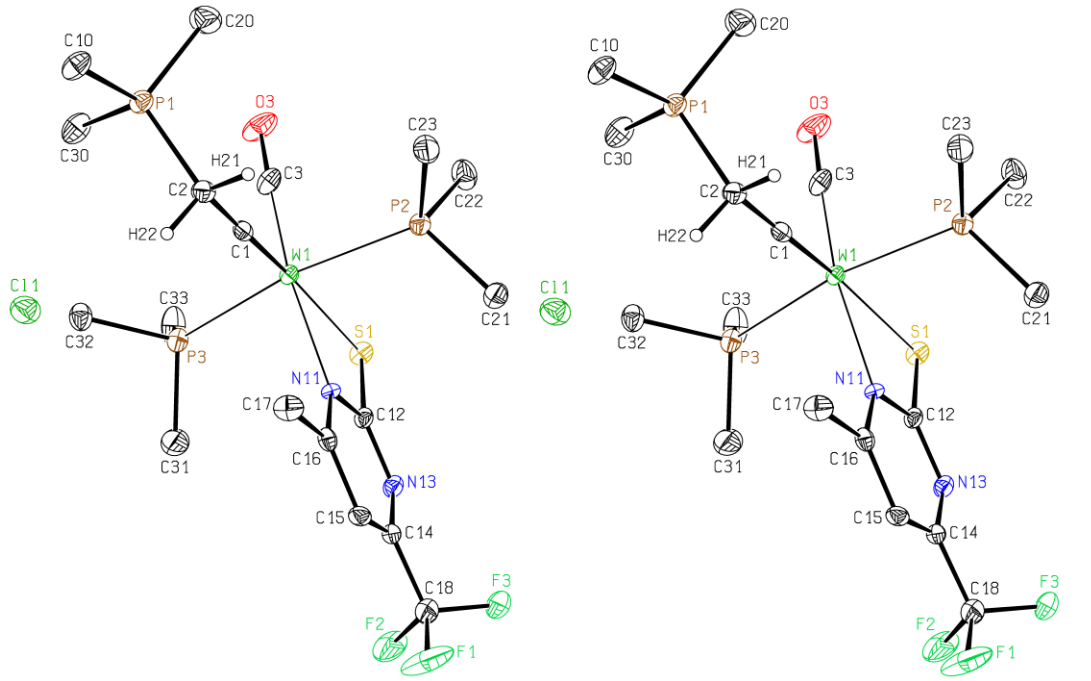

Figure S48. Stereoscopic ORTEP[9] plot of **2-Cl** showing the atomic numbering scheme. The probability ellipsoids are drawn at the 50% probability level. The H atoms of the ethylidyne group are drawn with arbitrary radii, the other H atoms were omitted for clarity.

Table S7. Selected bond lengths [Å] and angles [°] for **2-Cl**

W1–C1	1.793(3)
W1–C3	1.968(3)
W1–N11	2.204(2)
W1–P2	2.4666(9)
W1–P3	2.4915(8)
W1–S1	2.6780(8)
C1–C2	1.496(4)
C2–P1	1.796(3)
P1–C10	1.771(3)
P1–C30	1.774(3)
P1–C20	1.780(3)
C3–O3	1.165(4)
S1–C12	1.730(3)
C18–F3	1.323(4)
C18–F1	1.325(4)
C18–F2	1.330(4)
P2–C22	1.817(3)
P2–C21	1.817(3)
P2–C23	1.820(3)
P3–C32	1.814(3)
P3–C33	1.820(3)
P3–C31	1.821(3)

C1–W1–S1	169.14(9)
C3–W1–N11	163.80(11)
P2–W1–P3	167.33(3)
C2–C1–W1	178.6(3)
C1–C2–P1	114.0(2)
O3–C3–W1	177.6(3)
C12–S1–W1	78.88(10)
C16–N11–C12	118.9(2)
C16–N11–W1	135.0(2)
C12–N11–W1	106.04(18)

C1–W1–N11–C12	-178.77(19)
C1–W1–N11–C16	3.9(3)
C3–W1–S1–C12	179.4(3)
C1–C2–P1–C10	169.7(2)

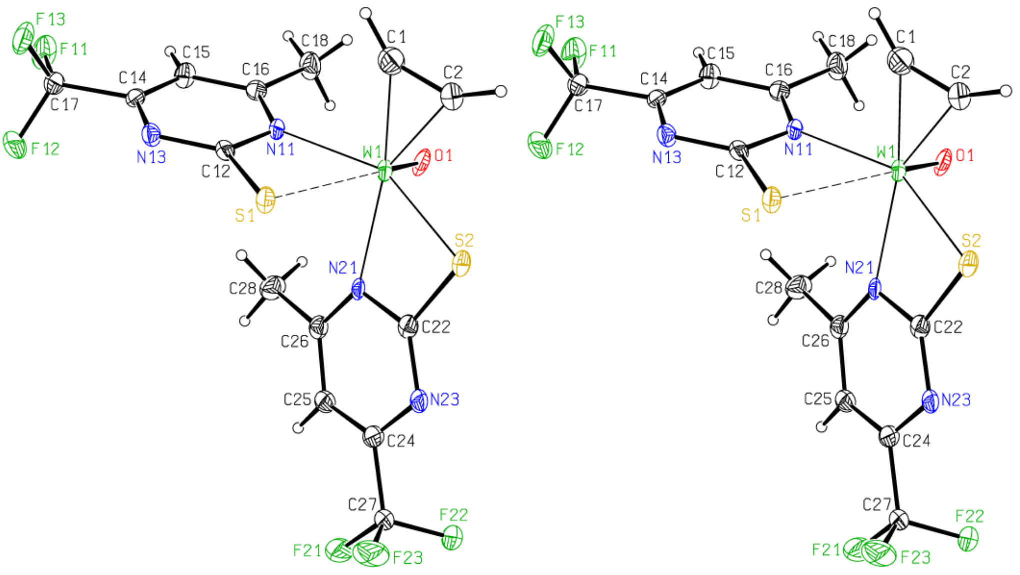

Figure S49. Stereoscopic ORTEP[9] plot of complex **A** of **3** showing the atomic numbering scheme. The probability ellipsoids are drawn at the 50% probability level. The H atoms are plotted with arbitrary radii. The rather long W–S distance is indicated by a dashed line.

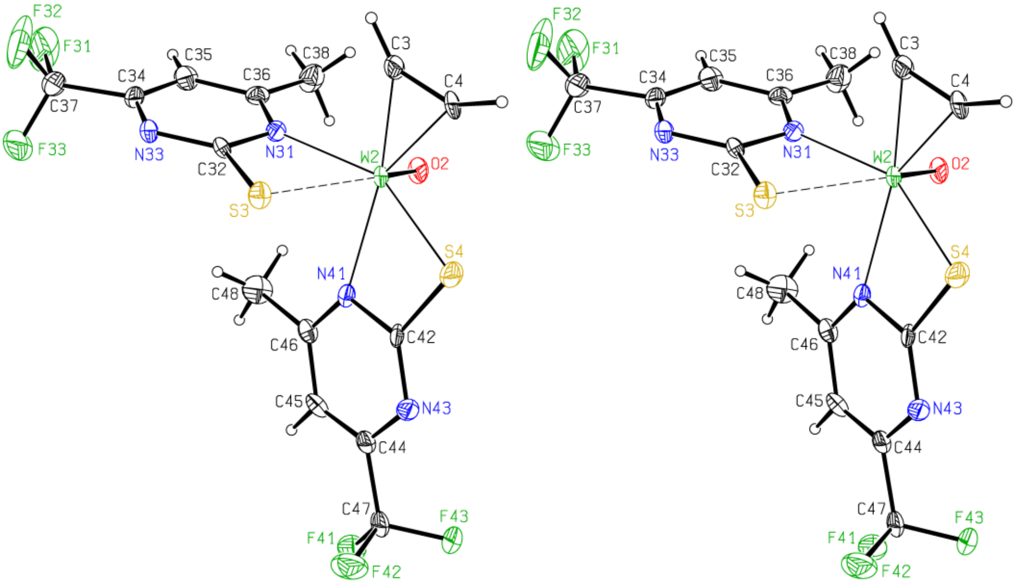

Figure S50. Stereoscopic ORTEP[9] plot of complex **B** of **3** showing the atomic numbering scheme. The probability ellipsoids are drawn at the 50% probability level. The H atoms are plotted with arbitrary radii. The rather long W–S distance is indicated by a dashed line.

Table S8. Selected bond lengths [Å] and angles [°] for **3**

W1–O1	1.718(5)	W2–O2	1.705(5)
W1–C1	2.088(8)	W2–C3	2.068(8)
W1–C2	2.074(8)	W2–C4	2.073(8)
W1–N11	2.200(6)	W2–N31	2.195(6)
W1–N21	2.273(6)	W2–N41	2.300(6)
W1–S1	2.6546(18)	W2–S3	2.6282(19)
W1–S2	2.4134(19)	W2–S4	2.420(2)
C1–C2	1.271(11)	C3–C4	1.257(11)
S1–C12	1.714(8)	S3–C32	1.716(8)
S2–C22	1.736(8)	S4–C42	1.735(7)
O1–W1–S1	151.83(18)	O2–W2–S3	151.71(18)
C1–W1–N21	166.7(3)	C3–W2–N41	167.1(3)
C2–W1–N21	146.1(3)	C4–W2–N41	146.5(3)
N11–W1–S2	149.55(16)	N31–W2–S4	149.39(17)
N11–W1–N21	94.2(2)	N31–W2–N41	93.0(2)
S1–W1–S2	89.61(6)	S3–W2–S4	89.86(7)
C12–S1–W1	79.6(2)	C32–S3–W2	79.8(2)
C12–N11–C16	119.8(6)	C32–N31–C36	119.6(6)
C12–N11–W1	105.8(5)	C36–N31–W2	135.4(5)
C16–N11–W1	134.4(5)	C32–N31–W2	105.0(5)
C22–S2–W1	84.2(3)	C42–S4–W2	84.9(3)
C22–N21–C26	118.1(6)	C42–N41–C46	118.5(6)
C22–N21–W1	99.2(4)	C42–N41–W2	98.8(4)
C26–N21–W1	142.5(5)	C46–N41–W2	141.7(5)
C1–C2–W1–O1	97.6(5)	C3–C4–W2–O2	96.0(5)
C2–C1–W1–O1	-91.2(5)	C4–C3–W2–O2	-92.2(5)
C1–C2–W1–N11	-3.9(6)	C3–C4–W2–N31	-3.2(6)
C2–C1–W1–N11	176.4(5)	C4–C3–W2–N31	177.1(5)
C1–C2–W1–S2	-157.0(5)	C3–C4–W2–S4	-157.4(5)
C2–C1–W1–S2	24.7(6)	C4–C3–W2–S4	24.5(6)
W1–N11–C12–S1	0.9(5)	W2–N31–C32–S3	1.7(5)
W1–S1–C12–N11	-0.8(4)	W2–S3–C32–N31	-1.4(4)
W1–N21–C22–S2	0.9(5)	W2–N41–C42–S4	4.6(5)
W1–S2–C22–N21	-0.8(5)	W2–S4–C42–N41	-4.3(5)

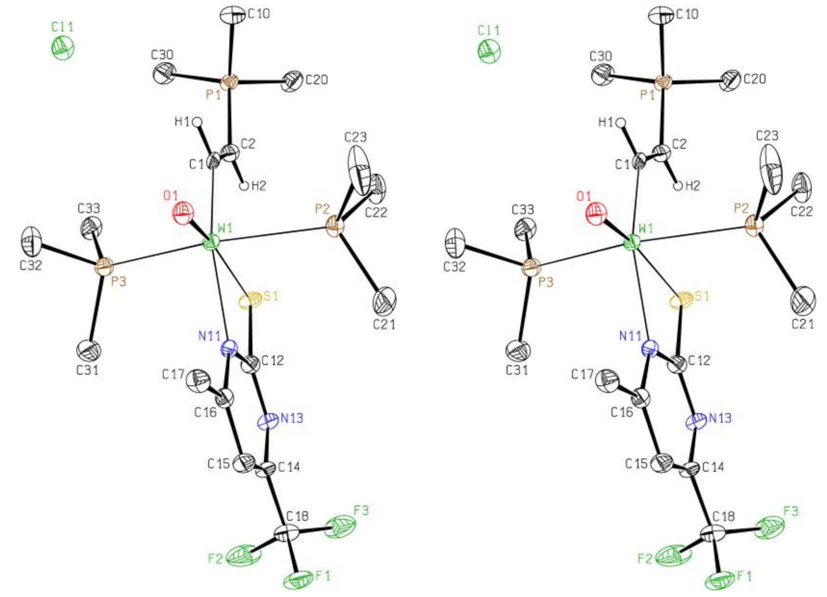

Figure S51. Stereoscopic ORTEP[34] plot of **4-Cl** showing the atomic numbering scheme. The probability ellipsoids are drawn at the 50% probability level. The H atoms of the ethenyl group are drawn with arbitrary radii, the other H atoms were omitted for clarity.

Table S9. Selected bond lengths [Å] and angles [°] for **4-Cl**

W1–O1	1.715(3)	C2–C1–W1	134.2(2)
W1–C1	2.074(3)	C2–C1–H1	114.1(7)
W1–N11	2.180(3)	W1–C1–H1	111.6(6)
W1–P2	2.5049(10)	C1–C2–P1	124.3(2)
W1–P3	2.4937(10)	C1–C2–H2	123.0(6)
W1–S1	2.6714(10)	P1–C2–H2	112.7(6)
C1–C2	1.351(5)	C12–S1–W1	79.08(14)
C2–P1	1.751(4)	C16–N11–C12	119.0(3)
P1–C10	1.773(4)	C16–N11–W1	134.4(3)
P1–C20	1.788(4)	C12–N11–W1	106.5(2)
P1–C30	1.789(4)		
P2–C23	1.797(5)	W1–C1–C2–P1	-177.51(18)
P2–C22	1.803(4)	C2–C1–W1–S1	-1.9(3)
P2–C21	1.811(5)	C2–C1–W1–O1	178.4(3)
P3–C33	1.808(4)	C1–W1–S1–C12	177.90(19)
P3–C32	1.816(4)	O1–W1–N11–C12	-178.8(3)
P3–C31	1.825(4)	O1–W1–N11–C16	-0.4(9)
S1–C12	1.719(4)		
O1–W1–S1	170.66(9)		
C1–W1–N11	146.80(12)		
P2–W1–P3	161.75(4)		

9 References

(1) Vidovič, C.; Peschel, L. M.; Buchsteiner, M.; Belaj, F.; Mösch-Zanetti, N. C. Structural Mimics of Acetylene Hydratase: Tungsten Complexes Capable of Intramolecular Nucleophilic Attack on Acetylene. *Chem. Eur. J.* **2019**, *25* (63), 14267–14272. DOI: 10.1002/chem.201903264

(2) Rodríguez, A.; Sousa-Pedrares, A.; García-Vázquez, J. A.; Romero, J.; Sousa, A. Electrochemical synthesis and characterization of zinc(II) complexes with pyrimidine-2-thionato ligands and their adducts with N,N donors. *Polyhedron* **2009**, *28* (11), 2240–2248. DOI: 10.1016/j.poly.2009.03.029

(3) Ehweiner, M. A.; Wiedemaier, F.; Belaj, F.; Mösch-Zanetti, N. C. Oxygen Atom Transfer Reactivity of Molybdenum(VI) Complexes Employing Pyrimidine- and Pyridine-2-thiolate Ligands. *Inorg. Chem.* **2020**, *59* (19), 14577–14593. DOI: 10.1021/acs.inorgchem.0c02412

(4) University of Karlsruhe and Forschungszentrum Karlsruhe GmbH. *TURBOMOLE*; University of Karlsruhe and Forschungszentrum Karlsruhe GmbH. http:// www.turbomole.com/

(5) Arnim, M. von; Ahlrichs, R. Performance of parallel TURBOMOLE for density functional calculations. *J. Comput. Chem.* **1998**, *19* (15), 1746–1757. DOI: 10.1002/(SICI)1096-987X(19981130)19:15<1746:AID-JCC7>3.0.CO;2-N

(6) Treutler, O.; Ahlrichs, R. Efficient molecular numerical integration schemes. *J. Chem. Phys.* **1995**, *102* (1), 346–354. DOI: 10.1063/1.469408

(7) Perdew, J. P.; Burke, K.; Ernzerhof, M. Generalized Gradient Approximation Made Simple. *Phys. Rev. Lett.* **1996**, *77* (18), 3865–3868. DOI: 10.1103/PhysRevLett.77.3865

(8) Grimme, S.; Anthony, J.; Ehrlich, S.; Krieg, H. A consistent and accurate ab initio parametrization of density functional dispersion correction (DFT-D) for the 94 elements H-Pu. *J. Chem. Phys.* **2010**, *132* (15), 154104. DOI: 10.1063/1.3382344

(9) Grimme, S.; Ehrlich, S.; Goerigk, L. Effect of the damping function in dispersion corrected density functional theory. *J. Comput. Chem.* **2011**, *32* (7), 1456–1465. DOI: 10.1002/jcc.21759

(10) Weigend, F.; Baldes, A. Segmented contracted basis sets for one- and two-component Dirac-Fock effective core potentials. *J. Chem. Phys.* **2010**, *133* (17), 174102. DOI: 10.1063/1.3495681

(11) Figgen, D.; Peterson, K. A.; Dolg, M.; Stoll, H. Energy-consistent pseudopotentials and correlation consistent basis sets for the 5d elements Hf-Pt. *J. Chem. Phys.* **2009**, *130* (16), 164108. DOI: 10.1063/1.3119665
(12) Eichkorn, K.; Treutler, O.; Öhm, H.; Häser, M.; Ahlrichs, R. Auxiliary basis sets to approximate Coulomb potentials - ERRATUM. *Chem. Phys. Lett.* **1995**, *242*, 652–660. DOI: https://doi.org/10.1016/0009-2614(95)00621-A
(13) Eichkorn, K.; Treutler, O.; Öhm, H.; Häser, M.; Ahlrichs, R. Auxiliary basis sets to approximate Coulomb potentials. *Chem. Phys. Lett.* **1995**, *240*, 283–290
(14) Eichkorn, K.; Weigend, F.; Treutler, O.; Ahlrichs, R. Auxiliary basis sets for main row atoms and transition metals and their use to approximate Coulomb potentials. *Theor Chem Acta* **1997**, *97* (1-4), 119–124. DOI: 10.1007/s002140050244
(15) Weigend, F. Accurate Coulomb-fitting basis sets for H to Rn. *PCCP* **2006**, *8* (9), 1057–1065. DOI: 10.1039/b515623h
(16) Plessow, P. Reaction Path Optimization without NEB Springs or Interpolation Algorithms. *J. Chem. Theory Comput.* **2013**, *9* (3), 1305–1310. DOI: 10.1021/ct300951j
(17) Kesharwani, M. K.; Brauer, B.; Martin, J. M. L. Frequency and zero-point vibrational energy scale factors for double-hybrid density functionals (and other selected methods): can anharmonic force fields be avoided? *J. Phys. Chem. A* **2015**, *119* (9), 1701–1714. DOI: 10.1021/jp508422u
(18) Klamt, A.; Schüürmann, G. COSMO: a new approach to dielectric screening in solvents with explicit expressions for the screening energy and its gradient. *J. Chem. Soc., Perkin Trans. 2* **1993** (5), 799–805. DOI: 10.1039/P29930000799
(19) Klamt, A.; Moya, C.; Palomar, J. A Comprehensive Comparison of the IEFPCM and SS(V)PE Continuum Solvation Methods with the COSMO Approach. *J. Chem. Theory Comput.* **2015**, *11* (9), 4220–4225. DOI: 10.1021/acs.jctc.5b00601
(20) Becke, A. D. Density-functional exchange-energy approximation with correct asymptotic behavior. *Phys. Rev. A* **1988**, *38* (6), 3098–3100. DOI: 10.1103/physreva.38.3098
(21) Becke, A. D. Density-functional thermochemistry. III. The role of exact exchange. *J. Chem. Phys.* **1993**, *98* (7), 5648–5652. DOI: 10.1063/1.464913
(22) Lee, C.; Yang, W.; Parr, R. G. Development of the Colle-Salvetti correlation-energy formula into a functional of the electron density. *Phys. Rev. B* **1988**, *37* (2), 785–789. DOI: 10.1103/physrevb.37.785

(23) Weigend, F.; Ahlrichs, R. Balanced basis sets of split valence, triple zeta valence and quadruple zeta valence quality for H to Rn: Design and assessment of accuracy. *PCCP* **2005**, *7* (18), 3297–3305. DOI: 10.1039/b508541a
(24) Weigend, F. A fully direct RI-HF algorithm: Implementation, optimised auxiliary basis sets, demonstration of accuracy and efficiency. *PCCP* **2002**, *4* (18), 4285–4291. DOI: 10.1039/b204199p
(25) Reed, A. E.; Weinstock, R. B.; Weinhold, F. Natural population analysis. *J. Chem. Phys.* **1985**, *83* (2), 735–746. DOI: 10.1063/1.449486
(26) Kollwitz, M.; Gauss, J. A direct implementation of the GIAO-MBPT(2) method for calculating NMR chemical shifts. Application to the naphthalenium and anthracenium ions. *Chem. Phys. Lett.* **1996**, *260*, 639–646. DOI: 10.1016/0009-2614(96)00897-4
(27) Ehweiner, M. A.; Ćorović, M. Z.; Belaj, F.; Mösch-Zanetti, N. C. Synthesis and Reactivity of Molybdenum and Tungsten Alkyne Complexes Containing 6-Methylpyridine-2-thiolate Ligands. *Helv. Chim. Acta* **2021**, *104* (11), e2100137. DOI: 10.1002/hlca.202100137
(28) Ehweiner, M. A.; Peschel, L. M.; Stix, N.; Ćorović, M. Z.; Belaj, F.; Mösch-Zanetti, N. C. Bioinspired Nucleophilic Attack on a Tungsten-Bound Acetylene: Formation of Cationic Carbyne and Alkenyl Complexes. *Inorg. Chem.* **2021**, *60* (12), 8414–8418. DOI: 10.1021/acs.inorgchem.1c00643
(29) Ehweiner, M. A.; Belaj, F.; Kirchner, K.; Mösch-Zanetti, N. C. Synthesis and Reactivity of a Bioinspired Molybdenum(IV) Acetylene Complex. *Organometallics* **2021**, *40* (15), 2576–2583. DOI: 10.1021/acs.organomet.1c00289
(30) Bondi, R.; Ćorović, M. Z.; Buchsteiner, M.; Vidovič, C.; Belaj, F.; Mösch-Zanetti, N. C. The Effect of Pyridine-2-thiolate Ligands on the Reactivity of Tungsten Complexes toward Oxidation and Acetylene Insertion. *Organometallics* **2021**, *40* (21), 3591–3598. DOI: 10.1021/acs.organomet.1c00472
(31) Chin, C. S.; Lee, M.; Oh, M.; Won, G.; Kim, M.; Park, Y. J. cis-Bis(alkenyl)iridium(III) Compounds by Apparent Insertion of Two Acetylenes into Two Ir−P Bonds: Crystal Structures of cis,trans-$[IrCl(-CHCH+PPh_3)_2(CO)(PPh_3)_2]^{2+}$ and $[Ir(OClO_3)(CH_3)(H_2O)(CO)(PPh_3)_2]^{+}$. *Organometallics* **2000**, *19* (8), 1572–1577. DOI: 10.1021/om0000459
(32) Sheldrick, G. M. A short history of SHELX. *Acta Cryst.* **2008**, *A64*, 112–122. DOI: 10.1107/S0108767307043930
(33) Sheldrick, G. M. Crystal structure refinement with SHELXL. *Acta Cryst.* **2015**, *71*, 3–8. DOI: 10.1107/S2053229614024218

(34) Johnson, C. K. *ORTEP. Report ORNL-3794.*, 1965
