# Supplementary material for: Understanding the Carbyne Formation from C2H2 Complexes
Source: J Am Chem Soc. 2024 Nov 15;146(47):32392–402. doi: 10.1021/jacs.4c07724 (PMC11613314; doi:10.1021/jacs.4c07724)
Supplement: Supplementary file 2 — ja4c07724_si_002.pdf [file ja4c07724_si_002.pdf]

Detailed DFT analysis of the mechanisms studied in the manuscript including alternative scenarios

This document is accompanying the manuscript:

Understanding the Carbyne Formation From C_2H_2 Complexes

*Miljan Z. Ćorović,‡ Madeleine A. Ehweiner,‡ Peter E. Hartmann,‡ Felix Sbüll, Ferdinand Belaj, A. Daniel Boese, Jesse Lepluart, Martin L. Kirk, Nadia C. Mösch-Zanetti**

1. Computational Details

All DFT calculations were performed with TURBOMOLE 7.4.1.[1–3] Geometries were optimized employing the PBE[4] functional together with the D3 dispersion correction using Becke-Johnson damping[5,6] and the dhf-SVP basis set.[7] To account for relativistic effects occurring for the central tungsten atom, the corresponding dhf-ecp (effective core potential) was utilized.[8] For speeding up the calculations, the resolution of identity (RI) approximation was utilized.[9–12] Transition states were located by using TURBOMOLE's woelfling-program,[13] followed by subsequent geometry optimization. Analytical normal modes were determined using TURBOMOLE's aoforce-program for confirmation of the stationary points and transition state search. After scaling of the frequencies,[14] the rigid-rotor-harmonic-oscillator (RRHO) approximation was used to calculate zero-point vibrational energies and thermal properties at room temperature (298 K).

To model solvent effects, geometries were reoptimized at the RI-PBE-D3BJ/dhf2-SVP level employing the COSMO-solvation model for dichloromethane ($\varepsilon_r = 8.930$).[15,16]

Single points of the transition states and minima were calculated with RI-PBE-D3BJ/dhf-TZVPP[7] and RIJK-B3LYP[17–19]-D3BJ/dhf2-TZVPP (with and without computing solvation effects), making use of the RIJK approximation for the B3LYP calculations.[18,20,21] Zero-point energies and thermal corrections for the COSMO-reoptimized structures and the single point calculations were taken from the RI-PBE-D3BJ/dhf-SVP gas phase calculations. The data reported in the main text and the remainder of this SI are the B3LYP-D3BJ/dhf2-TZVPP+COSMO data except otherwise noted. NBO-charges and Wiberg bond indices were calculated with the NBO implementation of TURBOMOLE[22] at the RIJK-B3LYP-D3BJ/dhf-TZVPP@RI-PBE-D3BJ/dhf-SVP level. NMR shifts were calculated using TURBOMOLE's mpshift program[23] at the RI-PBE-D3BJ/dhf-SVP level. This method has additionally been

verified to yield consistent results with calculations performed at both the RI-PBE-D3BJ/dhf-TZVPP and RIJK-B3LYP-D3BJ/dhf2-TZVPP level.

2. Detailed discussion of the computational investigations on the carbonyl-mechanism

In the following, we give a more detailed description of our computational studies on the mechanism of the transformation of complex **1** to **2-Cl**.

2.1. Initial steps of the mechanism until 1b

The pathways described in this section are summarized in Scheme 1, whereas the corresponding energy diagram is displayed in Diagram 1.

With **1a** presenting an experimentally identified candidate for a likely initial reaction intermediate, we started our investigations from there. Formation of **1a** requires two separate events, namely coordination of PMe_3 to the tungsten center of **1** and decoordination of the nitrogen atom of one of the PymS ligands. Initial decoordination appears highly unlikely due to the electron-deficiency of the resulting potential five-coordinate product, which is further corroborated by the fact that we were unable to optimize a corresponding structure as in all attempts the geometry reverted back to **1**.

An alternative initial attack at C_2H_2 is predicted to be kinetically disfavored. Due to the methyl group of the equatorially oriented PymS ligand (**PymS$_{eq}$**) blocking attack of PMe_3 from the "right side" (see Figure 1, A), attack can only take place at two sites, giving rise to **1a'$_1$@C_2H_2** (attack from "bottom", $\Delta G = +15.1$ kJ/mol) and **1a'$_2$@C_2H_2** (attack from "top", +9.0 kJ/mol). Formation of both hypothetical products is kinetically disfavored ($\Delta\Delta G^{TS} = +73.3$ and +71.9 kJ/mol, respectively), consistent with the experimental observations.

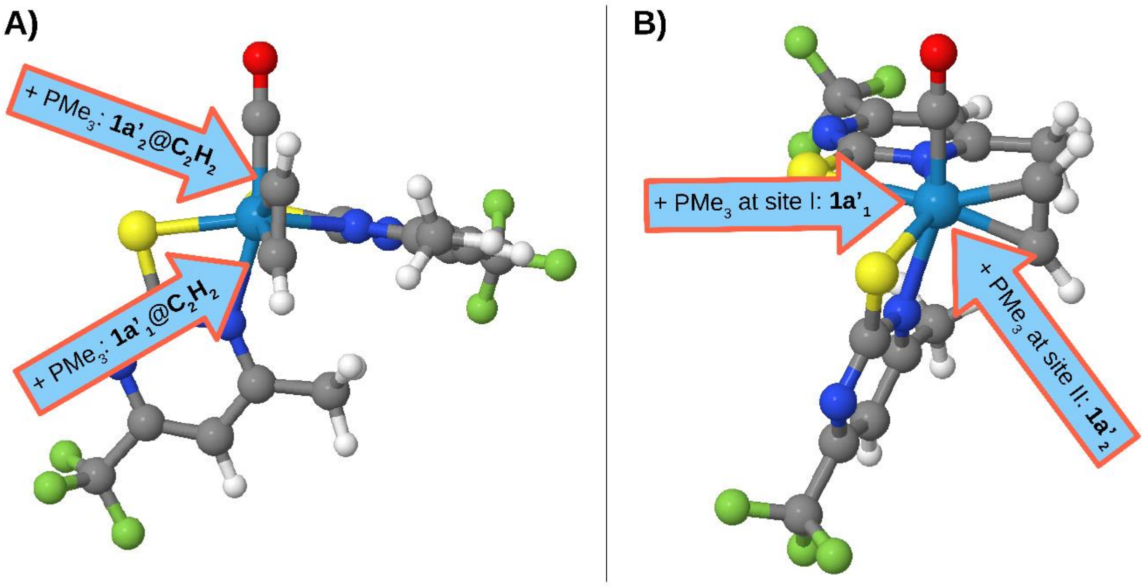

Figure 1. Investigated sites of attack/binding sites of PMe_3 (as indicated by arrows) at coordinated acetylene (A) and the tungsten (B) in **1**.

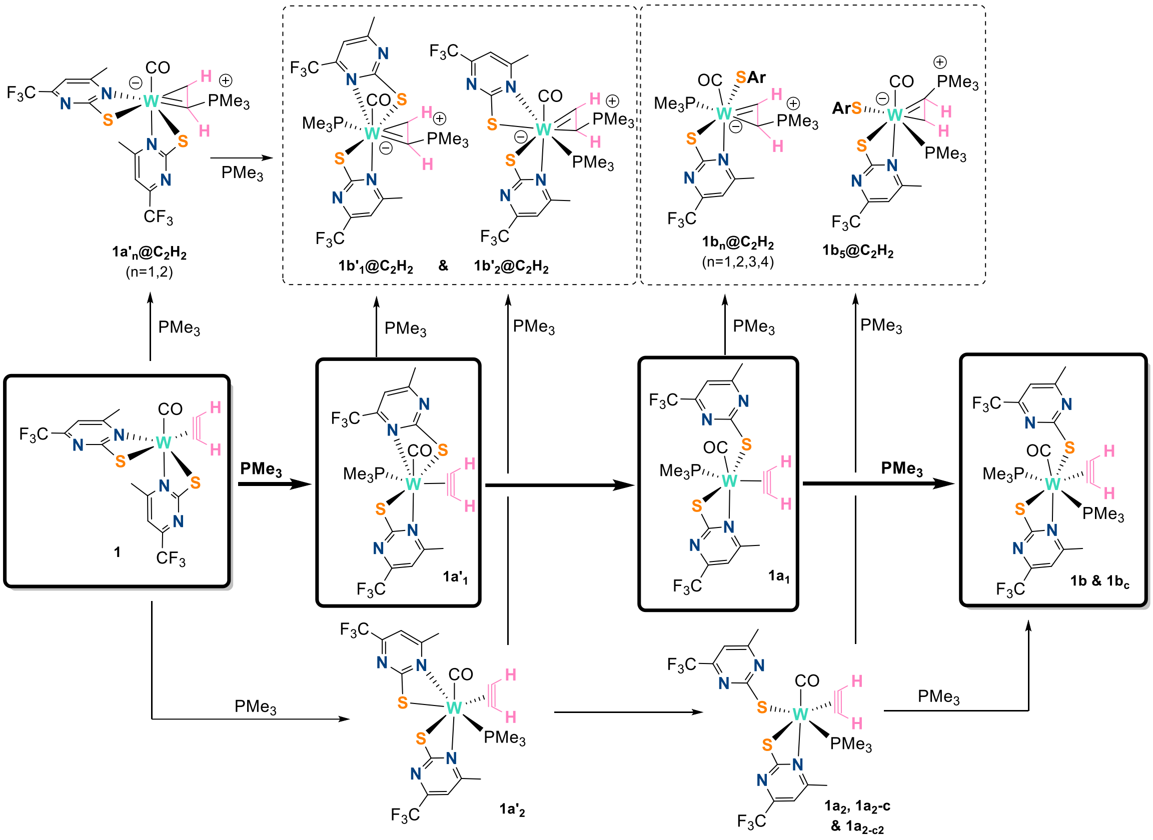

Scheme 1. Overview of the most important species and pathways discussed in this section. The favored pathway leading to **1b** is highlighted in bold.

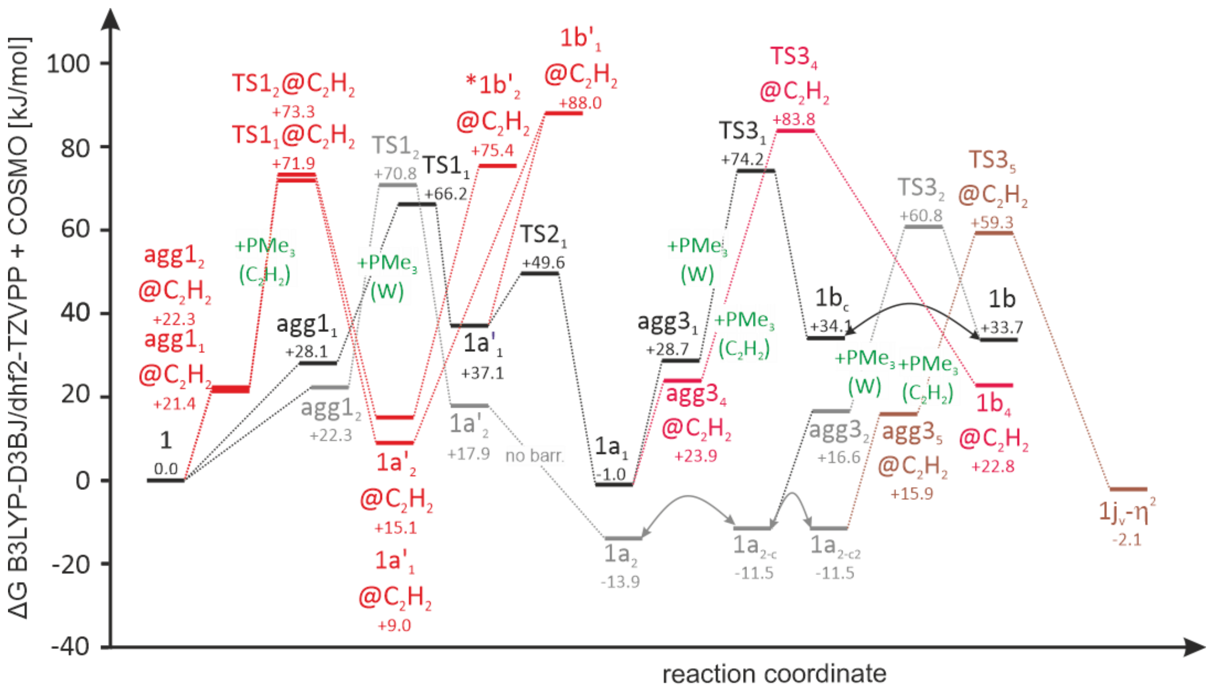

Diagram 1.Energy diagram of the carbonyl mechanism until **1b**. The value marked with an asterisk is the gas-phase free energy, as the COSMO geometry could not be obtained.

Thus, the first step of the mechanism is the attack of PMe_3 on the tungsten center of **1**, where several possible binding sites exist, yielding complexes **1a'$_n$**. The data indicate an attack *trans* to the C_2H_2 ligand (binding site I, Figure 1) to be kinetically favored ($\Delta\Delta G^{TS}$ = +66.2 kJ/mol), giving rise to complex **1a'$_1$** (called **1a'** in the main text), while attack *cis* to the C_2H_2 and *trans* to **PymS$_{eq}$** (binding site II) features a slightly higher energetic barrier ($\Delta\Delta G^{TS}$ = +70.8 kJ/mol), yielding complex **1a'$_2$**. Formation of both **1a'$_1$** (ΔG = +37.1 kJ/mol) and **1a'$_2$** (ΔG = +17.9 kJ/mol) is endergonic due to their seven-coordinate nature. Additionally, constitutional isomer **1a'$_3$** exists, which can, however, be neglected due to its very high energy (ΔG = +98.7 kJ/mol). Overall, this suggests that formation of **1a'$_1$** is the preferred pathway.

*2.1.1. Favored pathway from **1a'$_1$***

Attack of PMe_3 at the C_2H_2 ligand of **1a'$_1$** is not feasible due to the hypothetically formed products **1b'$_1$@C_2H_2** (attack from "bottom", compare Figure 1, +88.0 kJ/mol) and **1b'$_2$@C_2H_2** (attack from "top", +75.4 kJ/mol, gas phase value given, as COSMO structure could not be obtained) exhibiting very high energies.

However, **1a'$_1$** can easily convert to the more stable **1a$_1$** (ΔG = -1.0 kJ/mol, called **1a** in the main text) by decoordination of the nitrogen of **PymS$_{eq}$**. The small size of the barrier and the much higher stability of **1a$_1$** in comparison to **1a'$_1$** can easily be explained due to the reduction of the coordination number. The alternative decoordination of the axially oriented PymS ligand (**PymS$_{ax}$**) from **1a'$_1$** is thermodynamically disfavored and, hence, this possibility was excluded (**1a$_{1\text{-axdiss.}}$**, ΔG = +15.0 kJ/mol). These findings corroborate that the initial step of the mechanism is the formation of **1a'$_1$**, followed by partial decoordination of a PymS-ligand and formation of **1a$_1$**.

In **1a$_1$**, the next PMe_3 attacking the tungsten center has two possible binding sites *cis* to the C_2H_2 ligand, with the others being sterically blocked by the first PMe_3 molecule. Intermediate **1b$_c$** (ΔG = +34.1 kJ/mol) is formed from attack at binding site II ($\Delta\Delta G^{TS}$ = +75.2 kJ/mol), i.e., at the side of **PymS$_{ax}$** (which is still bound in a bidentate fashion). Attack at binding site III (Figure 2) between the C_2H_2 and the monodentate **PymS$_{eq}$** was ruled out, as it yields complex **1b$_{1\text{-III}}$**, which is thermodynamically highly disfavored (ΔG = +54.7 kJ/mol). Attack of PMe_3 from the side of the tungsten coordinated PMe_3 is sterically blocked.

Of **1b$_c$** there exists another conformer, **1b**, which differs only in the orientation of the monodentate PymS ligand: Both **1b$_c$** and **1b** exhibit a donor-acceptor interaction of the carbonyl carbon with one nitrogen of the monodentate 4,6-CF_3MePymS: Whereas in the case of **1b$_c$** the nitrogen next to the CH_3 group interacts with the CO ligand, for **1b** it is the one adjacent to the CF_3 group. Due to their similar energies (ΔG = +34.1 and +33.7 kJ/mol, respectively) and the relatively unobstructed trajectory of a potential rotation of the monodentate PymS ligand, the conformers are be expected to exist in an equilibrium.

Alternative pathways leading to an attack of PMe_3 at the C_2H_2 ligand of **1a₁** were also investigated. The latter exhibits four different potential sites for such an attack, giving rise to potential products $\mathbf{1b_n@C_2H_2}$ (Figure 2, n=1: right top; n=2: right bottom, n=3; left top; n=4: left bottom). Contrary to the oxo-mechanism from **3** to **4-Cl**, these pathways are both kinetically and thermodynamically highly disfavored (Table 1). Overall, these data therefore suggest that the mechanism commences from $\mathbf{1a_1}$ onward by formation of $\mathbf{1b_c}$/**1b**.

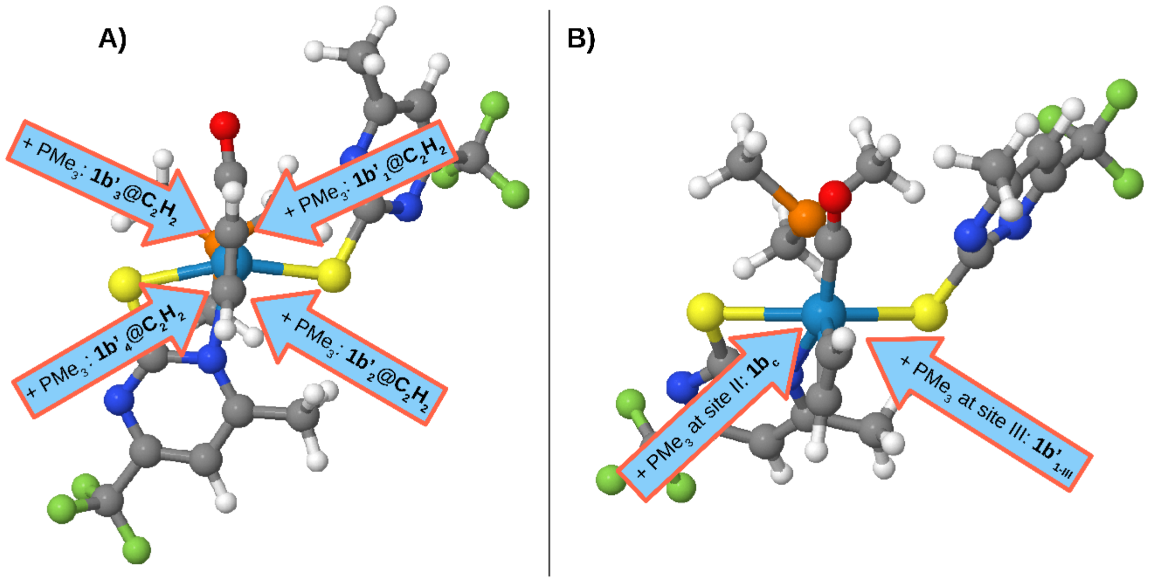

Figure 2. Investigated sites of attack/binding sites of PMe_3 (as indicated by arrows) at coordinated acetylene (A)

Table 1 Gibbs free energies of the hypothetical products $\mathbf{1b_n@C_2H_2}$ formed by attack of PMe_3 at C_2H_2 in $\mathbf{1a_1}$ and the associated transition states $\mathbf{TS3_n@C_2H_2}$ and pre-reaction agglomerates $\mathbf{agg3_n@C_2H_2}$. Additionally, the barrier heights ($\Delta\Delta G^{TS}$) associated with each transition state are given relative to the infinitely separated reactants.

N	$\mathbf{agg3_n@C_2H_2}$	$\mathbf{TS3_n@C_2H_2}$	$\Delta\Delta G^{TS}$	$\mathbf{1b_n@C_2H_2}$
1	n.a.	n.a.	n.a.	+46.3
2	+25.6	+83.8	+84.8	+26.9
3	n.a.	n.a.	n.a.	+45.9
4	+23.9	+83.8	+84.8	+22.8

2.1.2. *Disfavored pathway from* ***1a'₂***

In addition, we also investigated the hypothetical pathway starting from kinetically disfavored **1a'₂** (*vide supra*), as the energy difference between the two associated barriers of formation ($\Delta\Delta G^{TS}$ = +66.2 and +70.8 kJ/mol) are close enough to potentially allow the formation of **1a'₂** to occur as a side-pathway (Scheme 1). If formed, **1a'₂** would convert to $\mathbf{1a_2}$ (ΔG = -13.9 kJ/mol) by decoordination of the nitrogen of $\mathbf{PymS_{eq}}$ and, subsequently, by minor conformational

changes of the monodentate ligand, further to conformers **1a$_{2\text{-c}}$** and **1a$_{2\text{-c2}}$** (ΔG = -11.5 kJ/mol for both). Analogous to the favored pathway *via* **1a'$_1$**, decoordination of **PymS$_{ax}$** from **1a'$_2$** can be excluded due to the hypothetically resulting complex **1a$_{2\text{-axdiss.}}$** being thermodynamically disfavored (ΔG = +10.1 kJ/mol).

Subsequently, attack of PMe_3 at binding site I' of **1a$_{2\text{-c}}$** (cf. Figure 3) would then yield complex **1b** (ΔG = +33.7 kJ/mol, $\Delta\Delta G^{TS}$ = +72.3 kJ/mol). Similar to **1a$_1$**, attack of PMe_3 on **1a$_{2\text{-c}}$** (and its conformers) from the side of the tungsten-coordinated PMe_3 is sterically blocked. Likewise, attack at binding site III of **1a$_2$** (Figure 3) could also be ruled out again as it would yield high-energy complex **1b$_{2\text{-III}}$** (ΔG = +47.0 kJ/mol). Therefore, as **1b** and **1b$_c$** are expected to be in equilibrium, both the favored pathway *via* **1a'$_1$** and the disfavored pathway *via* **1a'$_2$** can ultimately lead to same species **1b$_c$**/**1b** (the two pathways are compared in Figure 4).

Interestingly, however, unlike the favored pathway, attack of PMe_3 at the coordinated C_2H_2 of **1a$_{2\text{-c2}}$** yielding **1j$_v$-η^2** (cf. Figure 3) is predicted to display a similar energy barrier as the attack at the tungsten center giving **1b** ($\Delta\Delta G^{TS}$ = +70.8 kJ/mol and $\Delta\Delta G^{TS}$ = +72.3 kJ/mol, respectively). The potential further transformations from **1j$_v$-η^2** onwards are described in section 2.2.5. and appear to lead to no further implications on the proposed main mechanistic route (further corroborated by the fact, that neither **1j$_v$-η^2** nor any of the potential intermediates further down the pathway are observable in experiment). This is due to the already disfavored nature of the pathway, which would lead to the formation of only minor amounts of **1a'$_2$** at most, and the subsequent competition between the further transformations to **1b** and **1j$_v$-η^2** (approximately equal amounts within the error of the method).

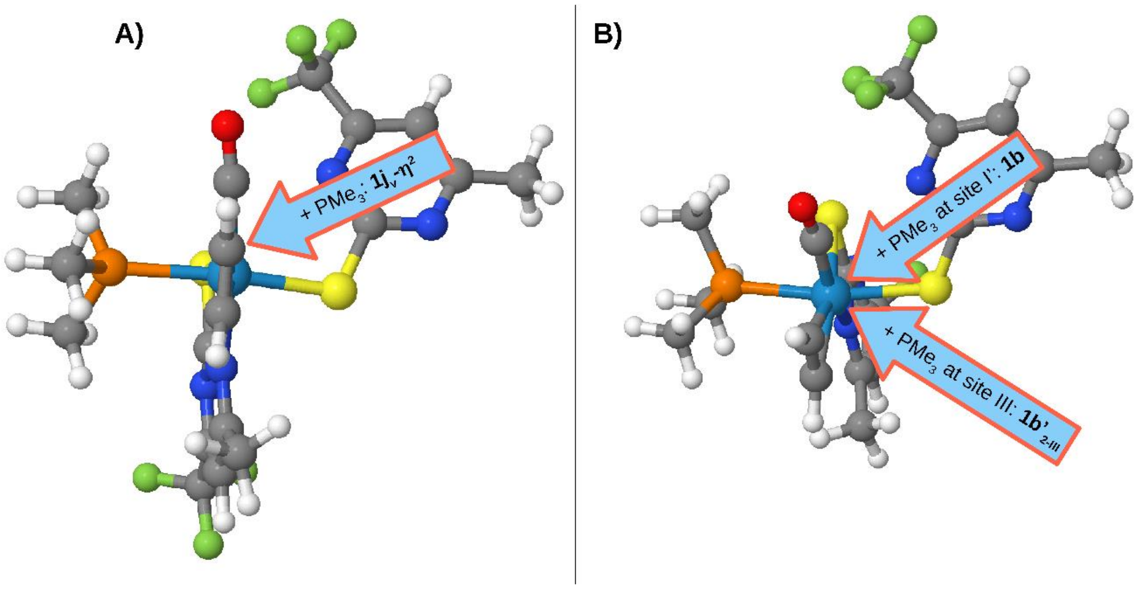

Figure 3 Investigated sites of attack/binding sites of PMe_3 (as indicated by arrows) at coordinated acetylene (A) and the tungsten (B) in **1a$_2$**. The labelling stays the same for conformers **1a$_{2\text{-c}}$** and **1a$_{2\text{-c2}}$** of **1a$_2$**.

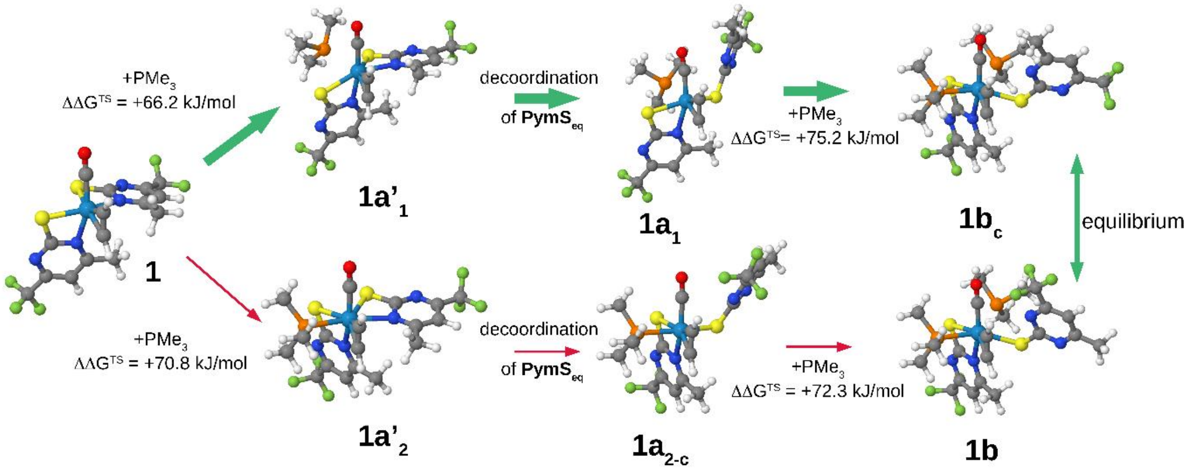

Figure 4 Overview of the favored pathway (green arrows) toward **1b** and the hypothetical alternative (red arrows).

2.2.Pathways from 1b: Carbyne formation and the experimental intermediate 1b''

Following the formation of **1b**, several possibilities arise how the mechanism could continue toward carbyne formation. Overall, four potential scenarios were identified and investigated in further detail (*vide infra*). Whereas scenarios I, II and IV could be ruled out (see the corresponding sections), scenario III (section 2.2.3) presents our final mechanistic proposal as presented in the main text.

I. Direct attack of PMe_3 at the acetylene ligand in **1b** (section 2.2.1.) followed by hydrogen shift towards the carbyne.
II. Partial decoordination of **PymS$_{ax}$** from **1b** yielding **1b$_{mono}$** and subsequent attack of PMe_3 at the acetylene ligand of the latter (section 2.2.2). This would be followed by ligand mediated (*via* a nitrogen of **PymS$_{eq}$**) hydrogen shift towards the carbyne.
III. loss of $PymS^-$ from **1b** and subsequent attack of the latter at the acetylene ligand (section 2.2.3). This would be followed by attack of PMe_3 at the newly formed tungstocyclopropene, yielding a tungstocyclopropane. Subsequent loss of PymS-anion by breakage of the C-S bond between the tungstocyclopropane and **PymS$_{eq}$** would be followed by ligand mediated (*via* the thiolate moiety of the **PymS$_{eq}$**-anion) hydrogen shift towards the carbyne.
IV. Partial decoordination of **PymS$_{ax}$** from **1b** and rearrangement of the coordination sphere yielding **1b$_{mono2}$**, followed by attack of a nitrogen of **PymS$_{eq}$** at the C_2H_2 ligand forming a tungstocyclopropene moiety (section 2.2.4). This would be followed by attack of PMe_3 at the newly formed tungstocyclopropene, yielding a tungstocyclopropane. Subsequent breaking of the C-N bond between the tungstocyclopropane and **PymS$_{eq}$** would be followed by ligand mediated (*via* a nitrogen of **PymS$_{eq}$**) hydrogen shift towards the carbyne.

2.2.1. *Scenario I: Direct attack of PMe_3 at **1b***

Looking for potential candidates for **1b''**, we first investigated the intuitive possibility of the formation of the corresponding η^2-vinyl complex **$1e_v$-η^2** (where the subscript "v" indicates the vertically aligned acetylene ligand, i.e., parallel to the axis of the W-CO bond) by addition of another molecule of PMe_3 to **1b**/**$1b_c$** (Scheme 2). However, all efforts to optimize **$1e_v$-η^2** failed as the corresponding configuration was unstable under geometry optimization. These findings suggest that this step is prevented due to steric hindrance, since the product would be too crowded to fit the additional PMe_3.

2.2.2. *Scenario II: Monodentate pathway via **$1b_{mono}$***

The pathways described in this section are summarized in Scheme 2, whereas the corresponding energy diagram is displayed in Diagram 2 Energy diagram of the monodentate pathway starting from **1b** described in **section** 2.2.2 Steps until 1b as described in section 2.1. are left in for comparison. The next possibility for continuation of the pathway would be decoordination of the nitrogen atom of the still bidentate PymS ligand **$PymS_{ax}$** of **1b**, yielding **$1b_{mono}$** ($\Delta G = +18.0$ kJ/mol). This route initially appeared entirely plausible, as chelate ligands of the employed type are known to exhibit a considerable degree of flexibility, and was, hence, investigated in further detail. However, compared to the energy necessary for the initial decoordination of **$PymS_{eq}$** from ($\Delta\Delta G^{TS} = +12.5$ kJ/mol), the energy barrier associated with the formation of **$1b_{mono}$** is much larger ($\Delta\Delta G^{TS} = +41.7$ kJ/mol). Together with the already discussed data on the disfavored initial decoordination of **$PymS_{ax}$** (*vide supra*), these findings indicate a high preference for **$PymS_{ax}$** to remain bound in a bidentate fashion. While, due to the difference in coordination number, **$1b_{mono}$** is predicted to be more stable than **1b** (**$1b_{mono}$**: $\Delta G = +18.0$ kJ/mol; **1b**: $\Delta G = +33.7$ kJ/mol), both **1b** and **$1b_{mono}$** are significantly less stable than their predecessors **$1a_1$** and **$1a_2$** ($\Delta G = -1.0$ and -13.9 kJ/mol).

Due to the reduction of steric crowding in **$1b_{mono}$** over **1b**, the C_2H_2 ligand of the former could be attacked by the third molecule of PMe_3, yielding the monodentate analogue of **1e-η^2**, **$1e_{mono,v}$-η^2** ($\Delta G = +35.7$ kJ/mol) *via* a comparably large energy barrier of +85.9 kJ/mol (again, the subscript "v" indicates the vertically aligned acetylene ligand, i.e., parallel to the axis of the W-CO bond). From the NMR data it appeared unlikely that **$1e_{mono,v}$-η^2** could be the observed short-lived reaction intermediate (as the two proton signals at 3.20 and 0.75 ppm do not fit with a typical η^2-complex, *vide supra*), which was confirmed by calculation of the chemical shifts of this complex (Table 2). Together with its comparably high calculated energy, this strongly suggest that **$1e_{mono,v}$-η^2** is not the observed species **1b''**.

Next, the further transformation towards the carbyne were investigated. In general, all calculated pathways of a hydrogen shift from an η^2-complex to a carbyne in this study (i.e., not limited to this subsection) yielded an η^1-complex as an intermediate, showing that, as expected *a priori*, this transformation encompasses several steps. Hence, pursuing the pathway from η^2-vinyl complex **$1e_{mono,v}$-η^2** onward further, the latter first transform into its corresponding η^1-

vinyl form. A minor conformational change with respect to the arrangement of the monodentate pyrimidine ligand in **1e$_{mono,v}$-η^2** yields **1e$_{mono,v,c}$-η^2** (ΔG = +34.6 kJ/mol). Breaking one W–C bond in **1e$_{mono,v,c}$-η^2** would give rise to high energy complex **1e$_{mono}$-η^1** (ΔG = +60.8 kJ/mol) *via* a comparably low barrier of formation ($\Delta\Delta G^{TS}$ = +42.8 kJ/mol). Subsequently, **1e$_{mono}$-η^1** could readily rearrange ($\Delta\Delta G^{TS}$ = +16.2 kJ/mol) to a form where the C–H bond of the W-bound carbon atom appears to exhibit agostic interactions with the metal center (**1e$_{mono,ago}$-η^1**, ΔG = +43.1 kJ/mol and its conformer, **1e$_{mono,ago,c}$-η^1**, ΔG = +47.7 kJ/mol). Both complexes **1e$_{mono}$-η^1** and **1e$_{mono,ago}$-η^1** can be ruled out as candidates for **1b''** due to their high energy and since the chemical shifts do not match the experimental data (Table 2).

While the pathway at hand did not feature a suitable candidate for **1b''**, we proceeded to investigate it further due to the possibility that **1b''** might also have been located off the main mechanistic pathway. Our calculations show that direct hydrogen shift from agostic η^1-complexes towards carbyne **1f$_{mono,c}$** would require overcoming a tremendous energy barrier ($\Delta\Delta G^{TS}$ = +161.9 kJ/mol), posing the question which feature of the reaction mechanism is responsible for facilitating this step. Upon closer inspection of the structure of **1e$_{mono,ago}$-η^1**, one of the nitrogen atoms of **PymS$_{eq}$** is located near the hydrogen atom which is to be transferred. This opens the possibility for a ligand-mediated hydrogen shift, where the hydrogen is first abstracted by the pyrimidine moiety ($\Delta\Delta G^{TS}$ = +58.4 kJ/mol), yielding highly unstable vinylidene species **1e$_{mono}$-H@N** (ΔG = +67.8 kJ/mol). **1e$_{mono}$-H@N** is too high in energy to present a probable candidate for **1b''**, which was confirmed by calculation of the chemical shifts (Table 2). Following the formation of **1e$_{mono}$-H@N**, transfer of the hydrogen to the respective carbon leads to the formation of **1f$_{mono}$** (ΔG = +15.5 kJ/mol) *via* an energy barrier of only +38.0 kJ/mol. Hence, the involvement of the pyrimidine moiety drastically brings down the energy barrier involved in the hydrogen transfer, suggesting a pivotal role in the reaction. This constitutes a thus far overlooked feature in mechanisms of carbyne complex formation, which led to the development of the final mechanistic proposal.

Coming back to the possibility of the observed intermediate **1b''** being located off the main mechanistic pathway, we also computed hydride **1e$_{hydride}$** and η^1-complexes **1e$_1$-η^1** and **1e$_2$-η^1**. The former could directly be excluded as a potential reaction intermediate due to its unreasonably high energy of +167.1 kJ/mol, moreover the calculated chemical shifts are inconsistent with the experimental shifts of **1b''** (Table 2) and lie in the expected range for a classical metal hydride (*vide supra*). The latter are analogues to **1e$_{mono}$-η^1**, where **PymS$_{ax}$** (the initially axially oriented PymS ligand) is coordinated in a bidentate fashion and, hence, could be formed from the corresponding reaction intermediates **1e$_{mono}$-η^1** and **1e$_{mono,ago}$-η^1** by recoordination of the respective PymS ligand. While **1e$_1$-η^1** and **1e$_2$-η^1** exhibit stabilities comparable to that of **1a$_1$** (**1e$_1$-η^1**: ΔG = +5.7 kJ/mol; **1e$_2$-η^1**: ΔG = -7.9 kJ/mol) and would therefore appear to be feasible candidates for **1b''** in terms of energy, also here the calculated NMR shifts are not consistent with experiment (Table 2). In addition, our calculations indicate that hydrogen shift in **1e-η^1** involving hydrogen abstraction by **PymS$_{eq}$** (analogous to the pathway *via* **1e$_{mono}$-H@N** outlined above) would not be feasible, as we were unable to obtain stable geometries for the required intermediates. This indicates that any carbyne formation from

species **1e$_1$-η^1** and **1e$_2$-η^1** would have to occur *via* **1e$_{mono}$-η^1**. Hence, species of the type of **1e$_1$-η^1** and **1e$_2$-η^1** indeed constitute a dead end regarding the formation of the experimentally observed carbyne product and were therefore not investigated further.

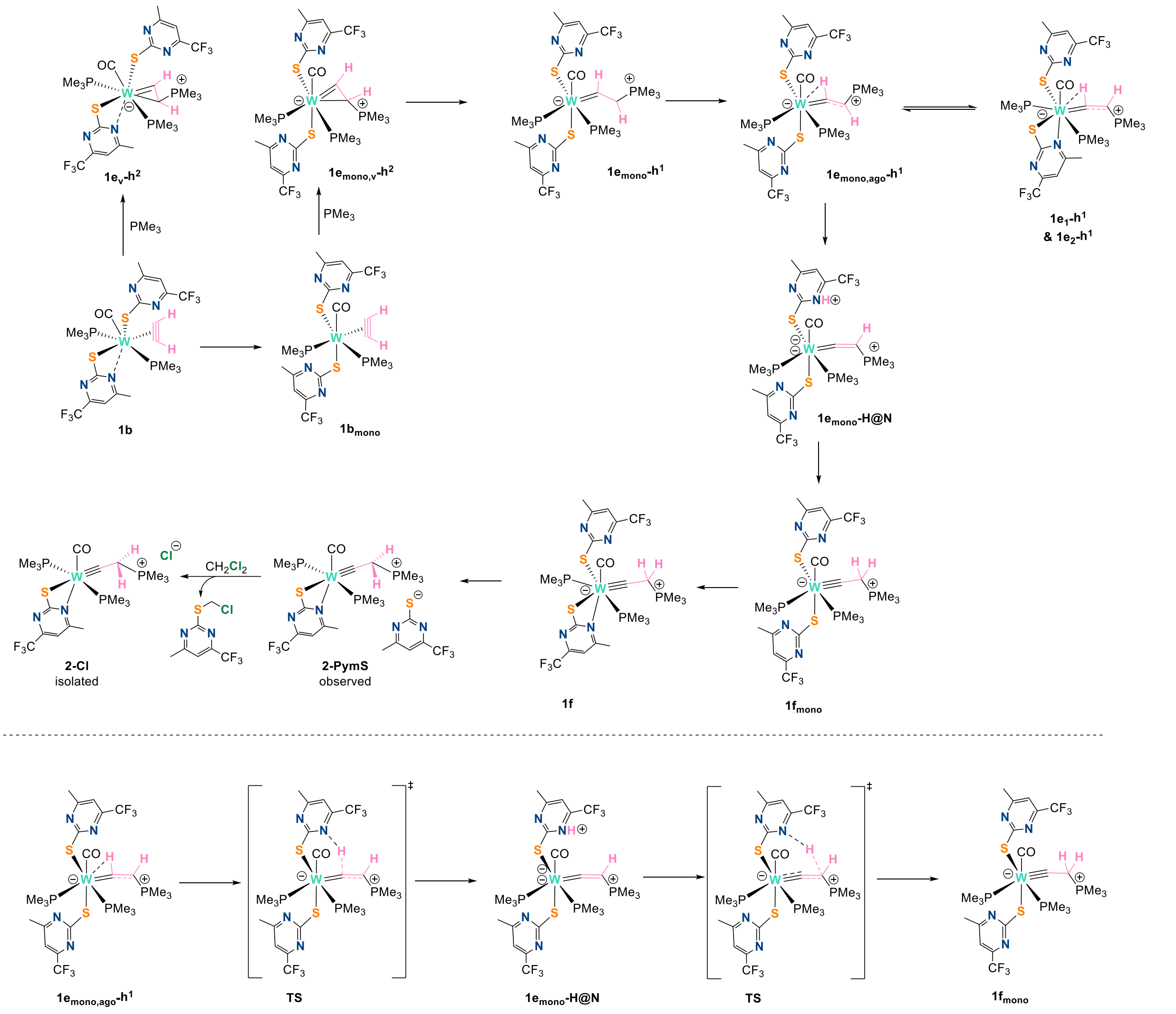

Scheme 2 Top: Overview of the investigated pathways starting from **1b** described in sections 2.2.1 and 2.2.2. Bottom: Hypothetical mechanism of the hydrogen shift involving species **1e$_{mono}$-H@N**.

Table 2 NMR shifts at PBE-D3BJ/dhf-SVP level

complex	ΔG	CH	CH	PymS-H	PymS-H	CH_3	CH_3	$P(CH_3)_3$	$P(CH_3)_3$	$P(CH_3)_3$
**exp. 1b''**	---	0.75	3.20	7.02	7.10	2.53	2.69	1.17	1.29	1.78
**1e$_{mono,v}$-η^2**	+35.7	2.39	8.13	6.38	6.46	2.01	2.35	1.13	1.23	1.87
**1e$_{mono}$-η^1**	+60.8	4.02	9.09	6.11	6.66	2.22	2.32	1.36	1.56	1.67
**1e$_{mono,ago}$-η^1**	+43.1	4.17	5.32	6.37	6.40	1.92	2.17	1.37	1.41	1.77
**1e$_{mono}$-H@N**	+67.8	3.51	16.35	5.40	6.30	1.90	2.09	1.04	1.43	1.64
**1e$_{hydride}$**	+167.1	-3.44	5.21	6.34	6.39	2.41	3.43	1.13	1.35	1.51
**1e$_1$-η^1**	+5.7	4.11	9.29	6.29	6.56	2.09	2.50	1.09	1.15	1.70
**1e$_2$-η^1**	-7.9	5.03	13.11	6.18	6.47	2.15	3.19	1.20	1.27	1.38

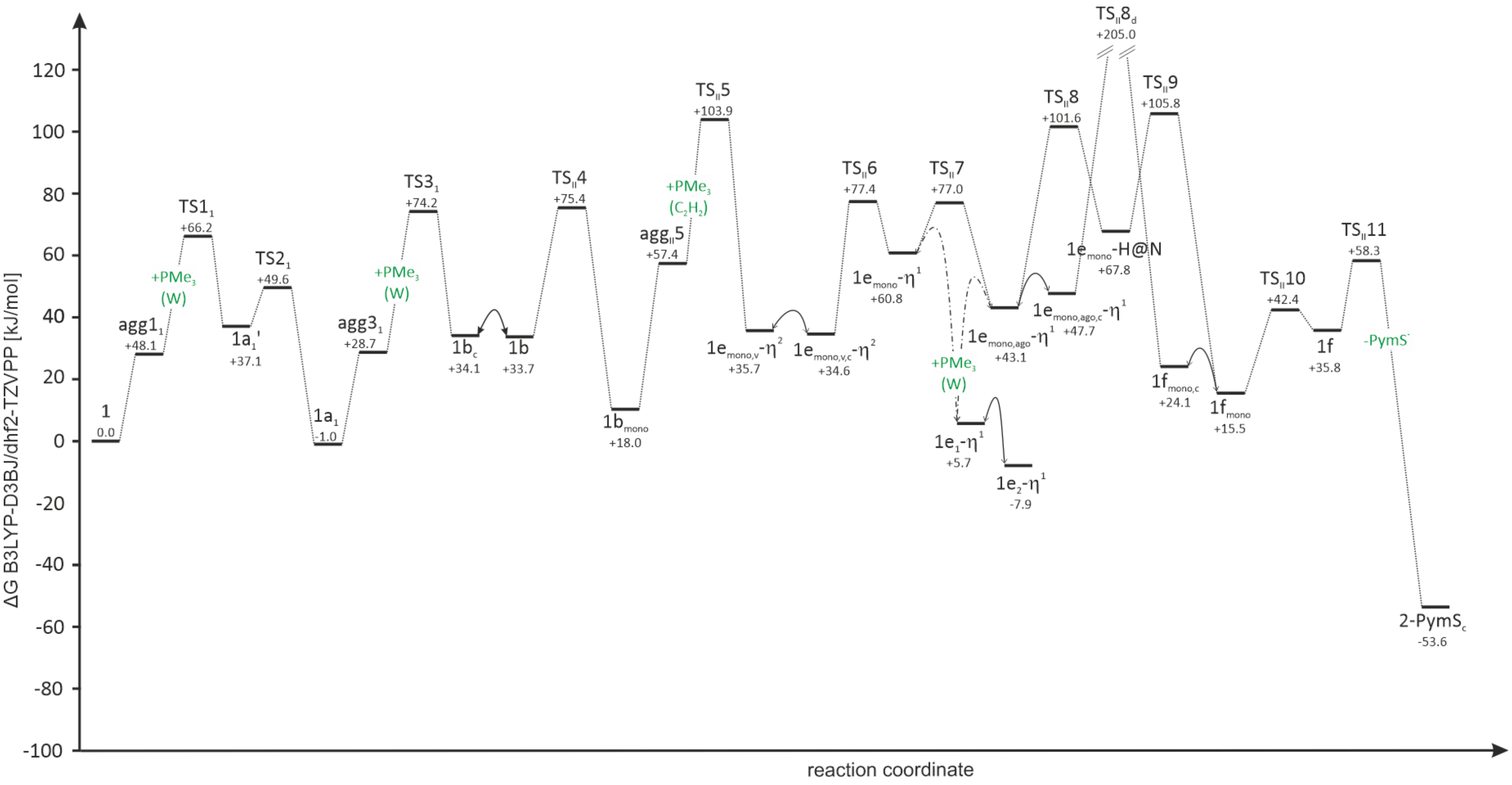

Diagram 2 Energy diagram of the monodentate pathway starting from **1b** described in section 2.2.2 Steps until **1b** as described in section 2.1. are left in for comparison.

Once **1f$_{mono}$** would be formed, the equatorial PymS ligand would need to be pushed out to proceed toward **2-PymS**. This could occur by recoordination of the nitrogen atom of **PymS$_{ax}$** to form **1f** and subsequent loss of 4,6-CF_3MePymS to form **2-PymS$_c$**, where the huge gain in energy upon formation of **2-PymS$_c$** would pull the equilibrium towards the side of the product ($\Delta\Delta G$ = -69.1 kJ/mol). Following carbyne formation, **2-PymS$_c$** could readily undergo transformation to its conformer **2-PymS** and further to **2-Cl** as described in section 2.2.3.

2.2.2.1. Issues with Scenario II

While the above outlined initially investigated mechanistic course of action at first appeared to provide a satisfactory explanation for the formation of the carbyne complex, it exhibits several issues of varying severity.

Firstly, a potential inconsistency regarding the final recoordination steps leading from **1f$_{mono}$** *via* **1f** to **2-PymS** arises. Considering the flexibility of the mono-coordinate **PymS$_{ax}$** in **1f$_{mono}$**, recoordination of **PymS$_{ax}$** in a manner rotated by 180° relative to the starting material appears plausible. This would yield **1f** and, ultimately, the final carbyne product in a configuration that does not match the obtained X-ray structure. This configuration of **PymS$_{ax}$** would be locked in, since loss of **PymS$_{eq}$** from **1f** can by and large be considered irreversible and the final carbyne products **2-PymS** and **2-Cl** should not exhibit partial decoordination of **PymS$_{ax}$** (as the resulting complexes would be five-coordinate, *vide supra*). While the fact that this configuration is not observed experimentally may well stem from recoordination of **PymS$_{ax}$** in the original configuration being favored, it is still worth noting that the monodentate scenario may potentially lead to ligand scrambling inconsistent with experiment (the corresponding calculations were omitted as the monodentate pathway was ruled out and, hence, not pursued further).

Secondly, the above discussed mechanistic pathway from **1b** to **2-PymS** does not feature a suitable candidate for the experimentally observed instable intermediate **1b''**. It is important to note that, while some discrepancies between the calculated and experimental NMR shifts are to be expected, especially regarding the aromatic protons of the PymS-ligands, the mismatches of all potential candidates for **1b''** listed in Table 2 are far outside anything that could be regarded as simply the error of the computational method.

Lastly, loss of **PymS$_{eq}$** from **1b** (barrierless) is highly favored over the partial decoordination of **PymS$_{ax}$** leading to the formation of **1b$_{mono}$** ($\Delta\Delta G^{TS}$ = +85.9 kJ/mol). The corresponding pathway is thoroughly discussed in section 2.2.3. and constitutes our final mechanistic proposal.

2.2.3. *Scenario III (**final mechanistic proposal**): Pathway via loss of PymS⁻ from **1b***

The issues arising from the monodentate pathway outlined in the previous section, especially the absence of any suitable candidate for **1b''**, prompted us to consider other pathways following the formation of **1b**. After reconsidering the NMR data of **1b''**, we grew to suspect the two intriguing proton signals mentioned above to stem from a tungstocyclopropane moiety, as both signals lie in a region (3.20 and 0.75 ppm) usually associated with protons bound to sp^3-hybridized carbons.

This prompted us to closely investigate a plethora of species of type **1w, 1x, 1y** and **1z** (Figure 5) which could hypothetically be formed would be consistent with the features of the short-lived reaction intermediate deduced from NMR. Their calculated chemical shifts are summarized in Table 3. Complexes of type **1w** feature a tungstocyclopropane moiety with both a PMe_3 and **PymS$_{eq}$** attached, where the latter is entirely decoordinated from the metal center and bound to the tungstocyclopropane *via* a nitrogen of the aromatic ring. Complexes **1x** also feature a tungstocyclopropane moiety with both a PMe_3 and **PymS$_{eq}$** (entirely decoordinated from the metal center) attached, where **PymS$_{eq}$** is bound by the sulfur atom. In complexes **1y**, the tungstocyclopropane moiety again exhibits both a bound PMe_3 and **PymS$_{eq}$** attached *via* a nitrogen atom of the aromatic ring, however, here **PymS$_{eq}$** remains coordinated to the metal center by the sulfur atom. Complexes **1z** bear analogy to complexes **1x** (PMe_3 and **PymS$_{eq}$** bound to the tungstocyclopropane, the latter by the sulfur atom), except that in **1z PymS$_{eq}$** is not decoordinated from the tungsten center but remains attached by one of the pyrimidine nitrogens.

Evidently, various isomers of these species are possible: (i) **PymS$_{eq}$** and PMe_3 can be bound in *cis* or *trans* fashion (subscripts "cis" and "trans"); (ii) the tungstocyclopropane moiety can be aligned either axially or equatorially [subscripts "h" (horizontal) and "v" (vertical)] and (iii) **PymS$_{eq}$** can attach either from the "top", i.e., from the direction of the carbonyl ligand, or from the "bottom", i.e., from the opposite direction of the carbonyl ligand [subscripts "1" (top) and "2" (bottom)]. In case binding of a nitrogen of a pyrimidine ring to the tungstocyclopropane occurs, this is possible *via* either the one next to the methyl or the CF_3 group of **PymS$_{eq}$** (subscripts "N-CH3" and "N-CF3").

For complexes **1w**, **1w$_{v1,trans,N-CH3}$** was investigated first (Figure 5), which proved unstable under geometry optimization. This indicates that other complexes of this type are unlikely to be reasonable candidates for **1b''** and, hence, they were not investigated further.

For species **1x**, a total of eight different isomers are possible (*vide* supra, Figure 5). The chemical shifts calculated for both **1x$_{h2,trans}$** (including its conformer **1x$_{h2,trans,c}$**) and **1x$_{h1,trans}$** are in good agreement with experiment (Table 3). Considering the thermodynamic stabilities, only **1x$_{h2,trans}$** (ΔG = -5.6 kJ/mol) exhibits a low-enough energy that is consistent with the observation of the short-lived reaction intermediate **1b''** in NMR, whereas **1x$_{h1,trans}$** can be ruled out (ΔG = +31.3 kJ/mol). The low energy of **1x$_{h2,trans}$** and good agreement of the calculated and experimental NMR shifts make it a prime candidate for **1b''**.

Regarding complexes **1y**, for axially oriented tungstocyclopropane, the sulfur atom of **PymS$_{eq}$** retains its place in the coordination sphere of the tungsten center, whereas for an equatorially aligned tungstocyclopropane moiety it must shift to an axial position (Figure 5), thereby

pushing **PymS$_{ax}$** in an equatorial position. Hence, whereas two pairs of complexes originate for axially oriented tungstocyclopropane (**1y$_{v1,cis}$** and **1y$_{v1,trans}$**; **1y$_{v2,cis}$** and **1y$_{v2,trans}$**), because of this rearrangement of the coordination sphere only one pair of complexes exists for the variant of featuring an equatorially aligned tungstocyclopropane (**1y$_{h2,cis}$** and **1y$_{h2,trans}$**) as **PymS$_{eq}$** cannot bind to the tungstocyclopropane from the direction of the carbonyl ligand. We started off by investigating both **1y$_{h2,trans,N\text{-}CH3}$** and **1y$_{h2,trans,N\text{-}CF3}$** to test for a preference regarding the pyrimidine nitrogens. Expectedly, the energy obtained for **1y$_{h2,trans,N\text{-}CH3}$** (ΔG = +4.2 kJ/mol) is much lower than the one for **1y$_{h2,trans,N\text{-}CF3}$** (ΔG = +29.5 kJ/mol). Hence, we restricted the further investigations of complexes **1y** to the ones of type **1y$_{N\text{-}CH3}$**, i.e., where **PymS$_{eq}$** is bound to the tungstocyclopropane moiety by the nitrogen neighboring the methyl group. Considering the thermodynamic stabilities and calculated NMR shifts (Table 3), only **1y$_{h2,trans,N\text{-}CH3}$** remains as a reasonable candidate for **1b''**.

For complexes **1z**, we first calculated the two complexes **1z$_{v2,trans,N\text{-}CH3}$** and **1z$_{v2,trans,N\text{-}CF3}$**. Their exceedingly large energies rule them out as candidates for **1b''** and indicate that also other complexes of this type are unlikely to be. Hence, the investigation of **1z** was not pursued further.

Overall, from our detailed screening only two complexes, namely **1x$_{h2,trans}$** and **1y$_{h2,trans,N\text{-}CH3}$**, remained as plausible candidates for **1b''**. Judging from their energies (ΔG = -5.6 kJ/mol and ΔG = +4.2 kJ/mol, respectively) a slight preference for **1x$_{h2,trans}$** might be deduced but such a rather small difference in energy may not be overinterpreted, especially without any knowledge of the involved energetic barriers yet. Upon comparing the agreement of calculated and experimental NMR shifts, the values for the tungstocyclopropane and PMe_3 protons are within the expected error of the method for both **1x$_{h2,trans}$** and **1y$_{h2,trans,N\text{-}CH3}$**, whereas the data for the aromatic and methyl hydrogens on the PymS ligands are a much better fit for **1x$_{h2,trans}$**. The experimental data display very similar shifts for each of the latter two groups of signals, hinting at relatively similar chemical environments for the two PymS ligands. While especially for the aromatic hydrogens a systematic relative shift of the calculated data can be observed, the relative distance between the two aromatic PymS hydrogen signals and the two methyl groups, respectively, are much more closer for **1x$_{h2,trans}$** than for **1y$_{h2,trans,N\text{-}CH3}$**. This can be attributed to the binding of **PymS$_{eq}$** to the tungstocyclopropane moiety by its aromatic nitrogen in **1y$_{h2,trans,N\text{-}CH3}$** having a much stronger impact on the chemical shifts of the protons of **PymS$_{eq}$** than the binding *via* its sulfur atom in **1x$_{h2,trans}$**.

From this analysis, **1x$_{h2,trans}$** can indeed be regarded as the more reasonable candidate for **1b''**, however, to confirm, both potential pathways leading to the final carbyne product *via* **1x$_{h2,trans}$** and **1y$_{h2,trans,N\text{-}CH3}$**, respectively, needed to be investigated. The pathway *via* **1y$_{h2,trans,N\text{-}CH3}$** could ultimately be disregarded due to the high energy required to form the initial intermediate **1b$_2$** (section 2.2.4.), whereas the pathway *via* **1x$_{h2,trans}$** presents our final mechanistic proposal as outline in the main text and will be discussed in the remainder of this section.

Formation of **1x$_{h2,trans}$** requires the migration of the monodentate PymS ligand to the coordinated C_2H_2, attack of PMe_3 on the C_2H_2 and turning of the (former) C_2H_2 ligand by 90°. The sequence needs to start with the with loss of the monodentate PymS ligand of **1b**, since direct attack of PMe_3 on the C_2H_2 ligand is sterically prevented (compare **1e$_v$-η^2**, section 2.2.1.) and formation of an isomer of **1b** in which the C_2H_2 ligand is bound in an equatorially aligned fashion is sterically prevented. Loss of the PymS ligand occurs barrierless and readily yields

ion pair **1b-PymS** (ΔG = +15.7 kJ/mol). Evidently, this step is strongly favored over the formation of **1b$_{mono}$** ($\Delta\Delta G^{TS}$ = +85.9 kJ/mol, section 2.2.2.). Depending on the location of the PymS$^-$ ion relative to the cationic remainder of the complex, the energy of **1b-PymS** can be lowered to +12.9 kJ/mol (**1b-PymS$_c$**) and +1.4 (**1b-PymS$_{c2}$**) kJ/mol.

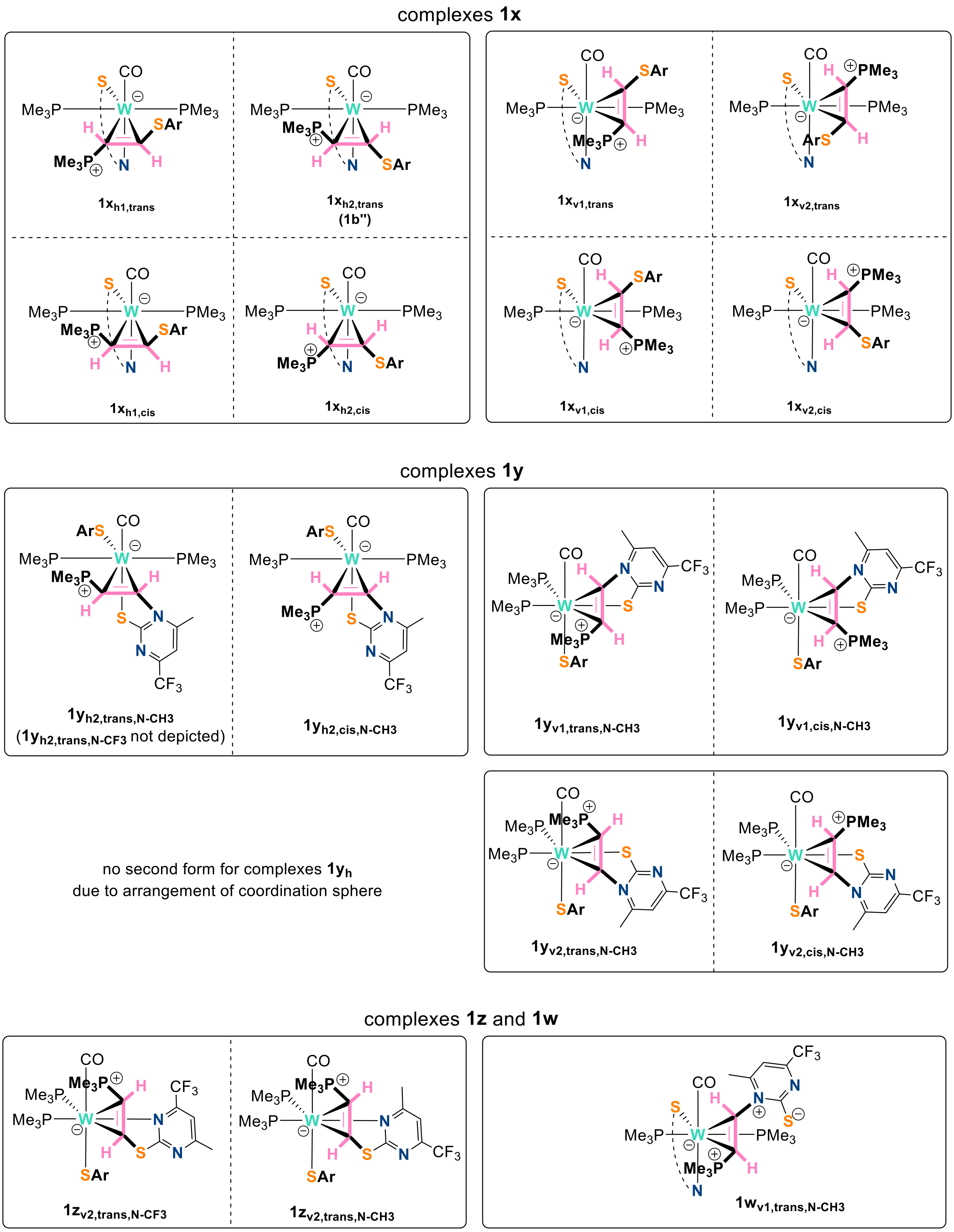

Figure 5 The various investigated candidates for experimentally observed short-lived intermediate **1b''**. Gibbs free energies are given in Table 3.

Table 3 NMR shifts at PBE-D3BJ/dhf-SVP level, the experimental data for **1b''** and plausible candidates for **1b''** are highlighted in bold, *: unstable geometry, loss of PymS ligand; $^{+}$: transforms to **1y**$_{v1,trans,N\text{-}CH3}$ under geometry optimization

complex	ΔG	CH	CH	PymS-H	PymS-H	CH$_3$	CH$_3$	P(CH$_3$)$_3$	P(CH$_3$)$_3$	P(CH$_3$)$_3$
**exp. 1b''**	**---**	**0.75**	**3.20**	**7.02**	**7.10**	**2.53**	**2.69**	**1.17**	**1.29**	**1.78**
**1w**$_{v1,trans,N\text{-}CH3}$	n.a.$^{+}$	n.a.$^{+}$	n.a.$^{+}$	n.a.$^{+}$	n.a.$^{+}$	n.a.$^{+}$	n.a.$^{+}$	n.a.$^{+}$	n.a.$^{+}$	n.a.$^{+}$
**1x**$_{h1,trans}$	+31.3	0.94	3.38	6.05	6.27	2.03	2.19	1.10	1.41	1.60
**1x**$_{h2,trans}$ **= 1b''**	**-5.6**	**1.10**	**2.81**	**6.14**	**6.50**	**2.20**	**2.36**	**0.97**	**1.32**	**1.79**
**1x**$_{h2,trans,c}$	**-2.6**	**1.09**	**2.92**	**6.17**	**6.54**	**2.23**	**2.35**	**0.97**	**1.33**	**1.83**
**1x**$_{h1,cis}$	+23.6	1.45	4.81	6.10	6.56	2.23	2.50	0.87	1.51	1.52
**1x**$_{h2,cis}$	+39.0	1.31	3.47	6.12	6.54	2.22	2.28	1.34	1.39	1.50
**1x**$_{v1,trans}$	+65.4	1.80	4.04	6.18	6.28	2.11	2.30	0.83	0.91	1.70
**1x**$_{v2,trans}$	+56.4	1.70	3.71	6.09	6.24	2.05	2.60	0.76	1.00	1.77
**1x**$_{v1,cis}$	+81.0	2.15	4.05	6.18	6.46	1.87	2.24	0.60	1.08	1.53
**1x**$_{v2,cis}$	n.a.*	n.a.*	n.a.*	n.a.*	n.a.*	n.a.*	n.a.*	n.a.*	n.a.*	n.a.*
**1y**$_{h2,trans,N\text{-}CH3}$	**+4.2**	**0.86**	**3.77**	**5.59**	**6.40**	**1.90**	**2.40**	**0.95**	**1.32**	**1.69**
**1y**$_{h2,trans,N\text{-}CF3}$	+29.5	0.99	4.20	5.31	6.37	1.61	2.40	0.97	1.30	1.70
**1y**$_{h2,cis,N\text{-}CH3}$	+28.1	0.95	4.37	5.75	6.52	1.97	2.31	1.00	1.35	1.50
**1y**$_{v1,trans,N\text{-}CH3}$	+70.8	3.42	4.04	5.41	6.41	1.66	1.96	1.22	1.37	2.04
**1y**$_{v1,cis,N\text{-}CH3}$	+48.4	-0.47	4.33	4.99	6.44	1.75	2.12	0.93	0.93	2.34
**1y**$_{v2,trans,N\text{-}CH3}$	+150.4	3.44	3.82	6.04	6.10	1.54	1.57	1.21	1.51	1.68
**1y**$_{v2,cis,N\text{-}CH3}$	+59.6	1.01	5.02	5.92	6.26	2.00	2.17	1.13	1.28	1.29
**1z**$_{v2,trans,N\text{-}CH3}$	+85.6	-0.11	3.22	6.30	6.33	2.14	2.48	1.29	1.36	1.54
**1z**$_{v2,trans,N\text{-}CF3}$	+121.7	1.05	1.98	6.13	6.46	2.06	2.14	1.36	1.48	1.57

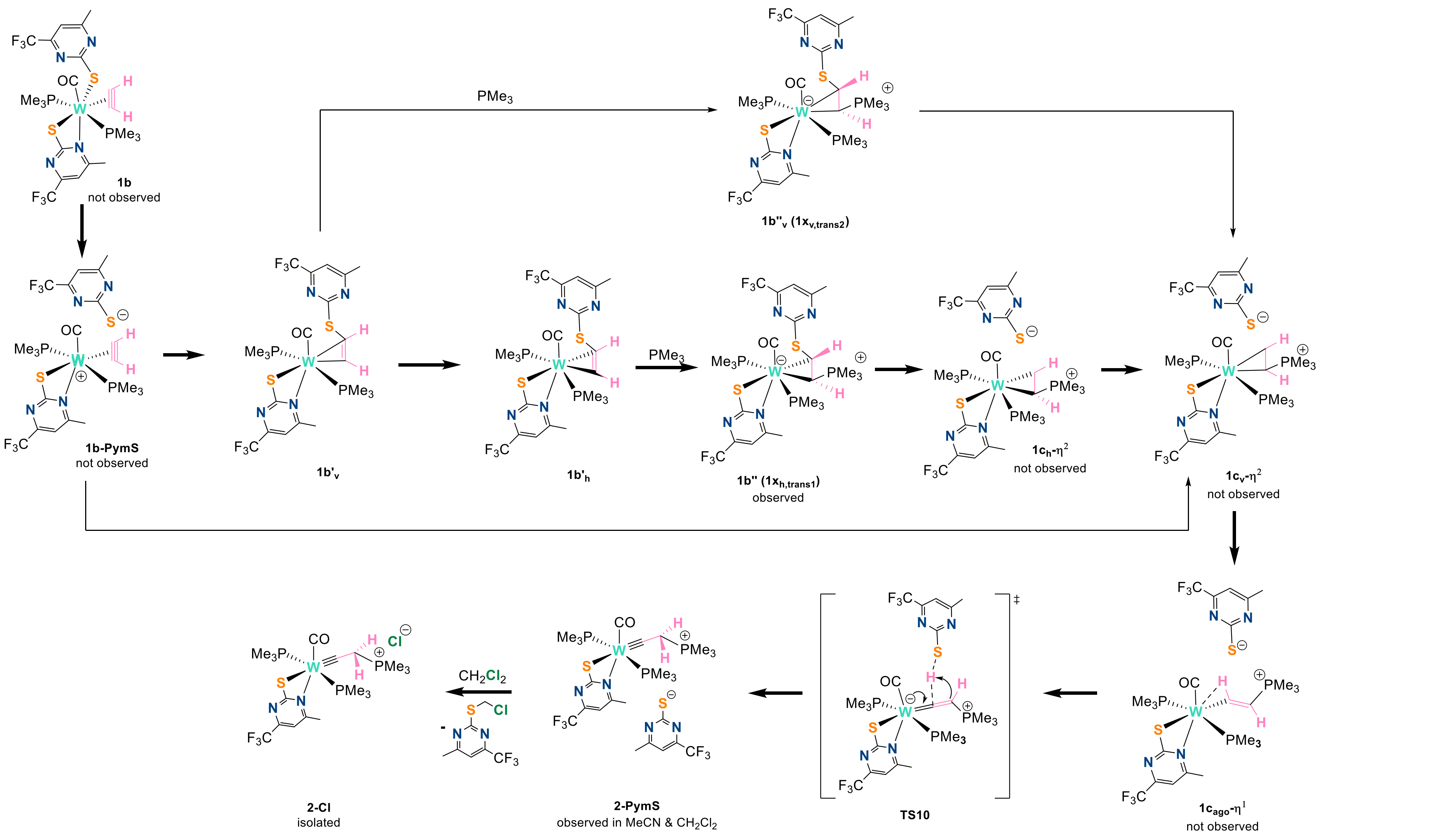

Scheme 3 Overview of the investigated pathways for our final mechanistic proposal starting from **1b** described in section 2.2.3. Favored pathway is marked with bold arrows.

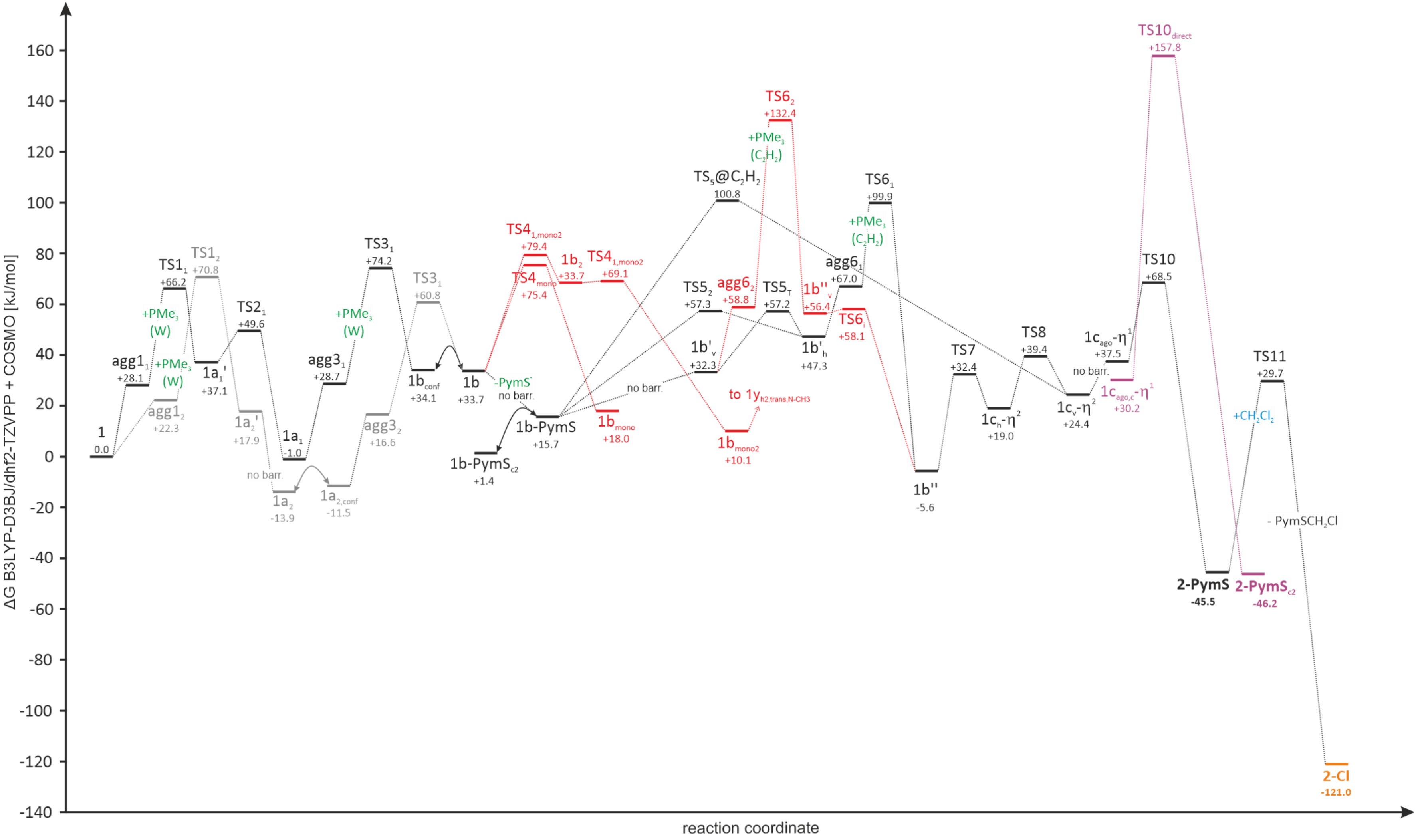

Diagram 3 Energy diagram of the pathways constituting our final mechanistic proposal as described in section 2.2.3. Steps until **1b** as described in section 2.1. are left in for comparison.

Attack of $PymS^-$ on the C_2H_2 ligand of **1b-PymS** exhibits no additional energetic barrier and yields **1b'**$_v$ ($\Delta G = +32.3$ kJ/mol), which features the newly formed tungstocyclopropene moiety bound in an axially aligned fashion (i.e., vertical, indicated by the subscript "v"). Contrary, attack of PMe_3 on **1b-PymS** (which would lead to **1c**$_v$**-η²**, *vide infra*) exhibits a large energy barrier of +85.1 kJ/mol. This indicates that $PymS^-$ here constitutes a much better nucleophile than PMe_3, which could be attributed to the cationic nature of the tungsten complex part in ion-pair **1b-PymS**. An alternative pathway leading from **1b-PymS** to **1b'**$_h$ ($\Delta G = +47.3$ kJ/mol), which features the tungstocyclopropene moiety bound in a horizontal (indicated by the subscript "h") is kinetically disfavored ($\Delta\Delta G^{TS} = +41.6$ kJ/mol). Of note, no transition state could be obtained for a direct shift of the PymS ligand from the metal center onto the acetylene ligand, i.e., as a single step process from **1b** to **1b'**$_v$ or **1b'**$_h$. Moreover, any attempt to optimize an analogue of **1b-PymS** in which the acetylene ligand is oriented horizontally failed, as the optimizations always lead to complex **1b'**$_h$. Together, these data indicate the mechanism from **1b** onward to proceed by formation of **1b'**$_v$.

After its formation, **1b'**$_v$ could subsequently be attacked by the third molecule of PMe_3, forming **1x**$_{\mathbf{v2,trans}}$ ($\Delta G = +56.4$ kJ/mol, Figure 5), the lowest energy isomer of **1x** which features the tungstocyclopropane moiety still in an axially aligned fashion. While subsequent transformation of **1x**$_{\mathbf{v2,trans}}$ to **1x**$_{\mathbf{h2,trans}}$ would be predicted to rapidly occur ($\Delta\Delta G^{TS} = +1.7$ kJ/mol), the formation of **1x**$_{\mathbf{v2,trans}}$ would require overcoming an exceedingly large barrier of +100.1 kJ/mol. Contrary, **1b'**$_v$ can easily transform further to **1b'**$_h$ ($\Delta G = +47.3$ kJ/mol) *via* rotation of the tungstocyclopropene moiety ($\Delta\Delta G^{TS} = +34.9$ kJ/mol). Attack of the third molecule of PMe_3 on **1b'**$_h$ is calculated to be much easier than on **1b'**$_v$, where the associated energy barrier is only about half the size ($\Delta\Delta G^{TS} = +52.6$ kJ/mol). Hence, the mechanism proceeds from **1b'**$_v$ *via* **1b'**$_h$ to **1x**$_{\mathbf{h2,trans}}$, where the formation of the latter is driven by the overall exergonicity of the transformation from **1b** onward. Having thus identified the identity of **1b''**, **1x**$_{\mathbf{h2,trans}}$ from here on out will be referred to as **1b''** for sake of simplifying the nomenclature.

In order to form the final product, expulsion of 4,6-CF_3MePymS^- from the tungstocyclopropane moiety of **1b''** is necessary, leading to the corresponding η^2-vinyl complex **1c-η²**. As previously mentioned (section 2.2.2), all calculated pathways of a hydrogen shift from an η^2-complex to a carbyne in this study (i.e., not limited to this subsection) yielded an η^1-complex as an intermediate, showing that, as expected *a priori*, this transformation encompasses several steps. Hence, **1c-η²** subsequently must first transform further into its η^1-form **1c**$_{\mathbf{ago}}$**-η¹** before it can undergo hydrogen shift to form carbyne **2-PymS**.

1c-η² again exists in two forms: **1c**$_h$**-η²**, which features a horizontally aligned tungstocyclopropane moiety, and **1c**$_v$**-η²**, in which the tungstocyclopropane moiety is oriented vertically. Similar to **1b''**, the horizontal isomer of **1c**$_h$**-η²** is thermodynamically preferred (**1c**$_h$**-η²**: $\Delta G = +19.0$; **1c**$_v$**-η²**: $\Delta G = +24.4$ kJ/mol). However, we were unable to obtain a transition state leading from **1c**$_h$**-η²** directly to **1c-η¹**, hence, **1c**$_h$**-η²** must first transform to **1c**$_v$**-η²**, which then converts barrierless into **1c**$_{\mathbf{ago}}$**-η¹**.

Following its formation, **1c**$_{\mathbf{ago}}$**-η¹** is poised to undergo hydrogen shift yielding carbyne **2-PymS** *via* a curiously low barrier of only +31.0 kJ/mol. The small size of this barrier could be attributed to the proximity of the sulfur atom of the $PymS^-$ anion in the associated transition state assisting the hydrogen transfer. Indeed, when recalculating the transition state without the

PymS⁻ anion located in proximity to the hydrogen being transferred, the barrier increases to an exorbitant +127.5 kJ/mol (the corresponding transformation occurs between a conformer of **$1c_{ago}$-η^1**, **$1c_{ago,c}$-η^1**, to a conformer of **2-PymS**, **2-PymS$_{c2}$**, compare Diagram 3). Complete removal of the PymS$^-$ anion and calculation of the cation only yields a similar result ($\Delta\Delta G^{TS}$ = +129.3 kJ/mol). These data suggest the pyrimidine moiety to play a pivotal assisting role in the hydrogen transfer rather than just being present as a spectator ligand in the reaction. The discarded alternative monodentate mechanisms outlined in sections 2.2.2. and 2.2.4. feature a similar role of the PymS-ligand aiding the hydrogen transfer necessary to form the carbyne, however, in these cases the aromatic nitrogen is involved rather than the sulfur. This finding presents a thus far overlooked feature in mechanisms of carbyne complex formation and may be at play also in the case of other, similar, reactions. For example, a similar mechanism might also be considered for carbyne formation involving the Tp' ligand in reference [24].

Interestingly, similar to the monodentate mechanism outlined in section 2.2.2, **$1c_{ago}$-η^1** exhibits agostic interactions between the tungsten center and the η^1-vinyl ligand. This interaction aids in the hydrogen transfer by weakening the C-H bond, allowing for easier deprotonation by the PymS$^-$ anion. This is reflected in the respective Wiberg bond indices: While in **$1c_v$-η^2** the bond index of the C-H_α bond is 0.872, for **$1c_{ago}$-η^1** it drops to 0.437, whereas the C-H_β bond index remains relatively unchanged (0.810 and 0.842, respectively).

Finally, following the formation of **2-PymS**, if CH_2Cl_2 is used as a solvent, the thiolate in **2-PymS** can undergo further reaction to **2-Cl** and 4,6-CF_3MePymSCH_2Cl. To obtain the height of the involved energy barrier for this step, we performed a potential energy surface (PES) scan for the analogous reaction of the PymS anion with CH_2Cl_2 from both the direction of the CH_3 and the CF_3 group, followed by transition state optimization. Attack from the side of the methyl group yielded an energy barrier of +81.7 kJ/mol, while attack from the side of the CF_3 group only requires +75.2 kJ/mol. Thus, the barrier for the transformation of **2-PymS** to **2-Cl** and 4,6-CF_3MePymSCH_2Cl was taken to be +75.2 kJ/mol. The comparably high amount of energy required for the reaction of the PymS$^-$ anion also rules out its conversion to 4,6-CF_3MePymSCH_2Cl and Cl^- already at the stage of **1b-PymS**, **1c-η^2** or **$1c_{ago}$-η^1**, i.e., before the formation of **2-PymS**, as in each case the energetic barriers described in the above discussion are significantly lower.

*2.2.3.1. Comments on the role of **1b''** and the order of attack at **1b-PymS***

Of note, formation of **$1c_v$-η^2** *via* **1b''** (in the following referred to as the indirect pathway) naively would appear to constitute a detour, as it involves first migration, i.e., addition, of the PymS ligand to C_2H_2, followed by attack of PMe_3 and then subsequent loss of PymS$^-$. This sparks the question, if **$1c_v$-η^2** could not be formed directly by attack of PMe_3 on the C_2H_2 ligand of **1b-PymS** (**TS_5@C_2H_2**) without the need of addition and loss of PymS$^-$ (in the following referred to as the direct pathway). Addition of PMe_3 to the acetylene ligand of **1b-PymS** is kinetically extremely disfavored ($\Delta\Delta G^{TS}$ = +85.1 kJ/mol, *vide supra*) over attack of PymS$^-$ [$\Delta\Delta G^{TS}$ = +16.6 kJ/mol (barrierless, ΔG between product and educt given)], thereby drastically favoring the formation of **1b'v** located in the indirect pathway. Despite this, as **1b-PymS** and

1b'$_h$ in the indirect pathway can be regarded to exist in equilibrium and the B3LYP-D3BJ/def2-TVPP+COSMO calculations predict the absolute heights of the highest involved transition states to be almost equal (**$TS_5@C_2H_2$**: ΔG = +100.8 kJ/mol; **$TS6_1$**: ΔG = +99.9 kJ/mol), the two possible routes could be expected to boast overall similar reaction rates. However, both the PBE-D3BJ/dhf-TZVPP+COSMO (**$TS_5@C_2H_2$**: ΔG = +85.9 kJ/mol; **$TS6_1$**: ΔG = +72.0 kJ/mol) and B3LYP-D3BJ/dhf-TZVPP gas-phase data (**$TS_5@C_2H_2$**: ΔG = +108.7 kJ/mol; **$TS6_1$**: ΔG = +69.8 kJ/mol) paint a very different picture, drastically favoring the indirect pathway. These findings indicate that the indirect route towards **$1c_v$-η^2** *via* **1b''** is likely to be the preferred one, however, the direct route cannot entirely be ruled out.

While in the indirect pathway **1b''** takes the role of a true reaction intermediate, in the direct pathway the experimental observation of **1b''** would be explained as a side product. This becomes clear upon examination of the energetic barriers from **$1c_v$-η^2** onward towards **2-PymS** (**TS10**: $\Delta\Delta G^{TS}$ = +31.0 kJ/mol) and back to **1b''** (highest barrier **TS8**: $\Delta\Delta G^{TS}$ = +15.0 kJ/mol), where the latter route, which essentially constitutes the backreaction of the indirect route from **1b''** to **$1c_v$-η^2**, is kinetically preferable (Diagram 3).

Interestingly, this indirect formation of the crucial η^2-intermediate with three PMe_3 moieties attached is also found for the discarded mechanism *via* **$1y_{h2,trans,N\text{-}CH3}$** (section 2.2.4). There, formation of the corresponding η^2-complex **$1e_{mono2,h}$-η^2** also preferentially occurs *via* initial attack of **$PymS_{eq}$** at the acetylene ligand, followed by attack of PMe_3 and subsequent breakage of the C-N bond of the tungstocyclopropene to **$PymS_{eq}$** instead of direct attack of PMe_3 on the acetylene. For both the final mechanistic proposal and the mechanism involving **$1y_{h2,trans,N\text{-}CH3}$** the energetic barrier of attack of PMe_3 is significantly lowered if the PymS-ligand first attaches to the acetylene ligand. This recurring sequence of mechanistic steps indicates an assisting catalytic role of the carbon-bound PymS-ligand not only in case of the hydrogen transfer but already in the mechanistic steps before.

2.2.3.2. Comments regarding the agreement with the experimental detection of complexes 1a and 1b''

Furthermore, the obtained data provide a good explanation for why species of type **$1a_n$** are the only intermediate observed in NMR until the formation of **1b''**. Firstly, all other intermediates *en route* feature comparatively high energies (Diagram 3). Secondly, the facts that the barrier associated with the formation of **$1b_{conf}$ /1b** is larger than the one that must be overcome for the formation of **$1a_1$'/$1a_1$**, and that any **$1a'_1$** formed by back-reaction from **$1a_1$** will rapidly revert to **$1a_1$** again will result in an accumulation of complex **$1a_1$** (this is reminiscent of the oxo-mechanism, *vide infra*). As for the observation of **1b''** in only trace amounts, while it constitutes a thermodynamic sink, all barriers lying to the right of **1b''** are lower in magnitude than the ones leading to its formation, preventing any accumulation of **1b''** in considerable amounts during the course of the reaction.

As a sidenote, the calculations curiously predict the backreaction from **$1b_{conf}$/1b** to **$1a_n$** to predominantly yield **$1a_2$** instead of the initially formed **$1a_1$**, as the associated barrier is lower ($\Delta\Delta G^{TS}$ = +27.1 and +40.1 kJ/mol, respectively). While loss of the PymS ligand from **1b** is kinetically preferred (barrierless), this does not exclude the possibility of formation of a small

amount of **1a$_2$** due to the barriers further down the mechanistic pathway exhibiting comparable or larger sizes, potentially leading to a mixture of **1a$_1$** and **1a$_2$** in the reaction vessel. However, as both **1a$_1$** and **1a$_2$** ultimately are converted back to the same species **1b**/**1b$_{conf}$** (*vide supra*), this does not hinder the further transformations towards the final product.

2.2.4. *Scenario IV: Pathways via 1y*

As outlined above (section 2.2.3), **1y**$_{\mathbf{h2,trans,N\text{-}CH3}}$ initially also represented a plausible candidate for the experimentally observed short-lived in intermediate **1b''**. While judging from the calculated energies and NMR shifts **1x**$_{\mathbf{h2,trans}}$ appeared to be the more likely contender, without any further investigation the calculated data would not allow for any definitive conclusion. Additional necessity for a closer inspection of a pathway incorporating **1y**$_{\mathbf{h2,trans,N\text{-}CH3}}$ arises due to the fact that insertion of an acetylene ligand into the W-N bond to a PymS ligand is known to occur for several other systems,[25] making it an overall reasonable mechanistic route to assume. The pathways described in this section are summarized in Scheme 4, whereas the corresponding energy diagram is displayed in Diagram 4.

Formation of **1y**$_{\mathbf{h2,trans,N\text{-}CH3}}$ requires both insertion of the acetylene ligand into the W-N bond to **PymS**$_{\mathbf{eq}}$ and partial decoordination of **PymS**$_{\mathbf{ax}}$ (by breaking the W-N bond) accompanied by rearrangement of the coordination sphere by shifting mono-coordinated **PymS**$_{\mathbf{eq}}$ to the axial position (i.e., opposite to the carbonyl ligand). Additionally, analogous to the formation of **1x**$_{\mathbf{h2,trans}}$, turning of the (former) C_2H_2 ligand by 90° must occur. Since formation of an isomer of **1b** in which the C_2H_2 ligand is bound in an equatorially aligned fashion (i.e., perpendicular to the W-CO bond) is sterically prevented (section 2.2.3), either one of the former events is required to occur first.

Initial partial decoordination of **PymS**$_{\mathbf{ax}}$ and rearrangement of the coordination sphere by shift of **PymS**$_{\mathbf{eq}}$ would lead to **1b**$_{\mathbf{mono2}}$, a constitutional isomer of **1b**$_{\mathbf{mono}}$ (compare section 2.2.2.) which features **PymS**$_{\mathbf{eq}}$ coordinated in the axial position and **PymS**$_{\mathbf{ax}}$ in an equatorial position opposite to the acetylene ligand. While **1b**$_{\mathbf{mono2}}$ displays a comparably low energy (ΔG = +10.1 kJ/mol), its formation was determined to have to occur *via* intermediate **1b**$_{\mathbf{2}}$, a high-energy (ΔG = +68.5 kJ/mol) constitutional isomer of **1b** in which **PymS**$_{\mathbf{eq}}$ is coordinated in a bidentate fashion instead of **PymS**$_{\mathbf{ax}}$. Similar to the transformation of **1b** to **1b**$_{\mathbf{mono}}$, which requires overcoming a barrier of +41.7 kJ/mol, the highest energetic barrier of the transformation of **1b** to **1b**$_{\mathbf{mono2}}$ is +45.3 kJ/mol, which is to be expected due to the severe geometric rearrangements necessary for both pathways. Alternative direct insertion of the acetylene ligand of **1b** into the W-N bond to **PymS**$_{\mathbf{eq}}$ gives rise to complexes **1g**$_{\mathbf{v1}}$ and **1g**$_{\mathbf{v2}}$ (attack of aromatic nitrogen at the "top" and the "bottom" of the acetylene ligand, i.e., the carbon atoms closer and further away from the CO ligand, respectively), which both exhibit a very high energy (**1g**$_{\mathbf{v1}}$: ΔG = +84.5 kJ/mol; **1g**$_{\mathbf{v2}}$: ΔG = +151.3 kJ/mol) and can thus be ruled out. Overall, compared to the barrierless conversion of **1b** to **1b-PymS** by loss of **PymS**$_{\mathbf{eq}}$, the latter process is clearly preferred, strongly favoring the mechanism *via* **1x**$_{\mathbf{h2,trans}}$ as outlined in section 2.2.3.

However, as the mechanistic route taken may also depend on the height of the barriers further down the pathway, we nevertheless continued the investigation of the mechanism *via* **1y**$_{\mathbf{h2,trans,N\text{-}CH3}}$. Attack of either PMe_3 or **PymS**$_{\mathbf{eq}}$ (which is now located in the axial position) on the vertically oriented acetylene ligand of **1b**$_{\mathbf{mono2}}$ is geometrically prevented, hence, rotation of the acetylene by 90° would have to occur first. This rotation can occur in two directions ($\Delta\Delta G^{TS}$ = +40.0 and +37.1 kJ/mol, respectively), giving rise to **1b**$_{\mathbf{mono2,h}}$ (ΔG = +36.8 kJ/mol).

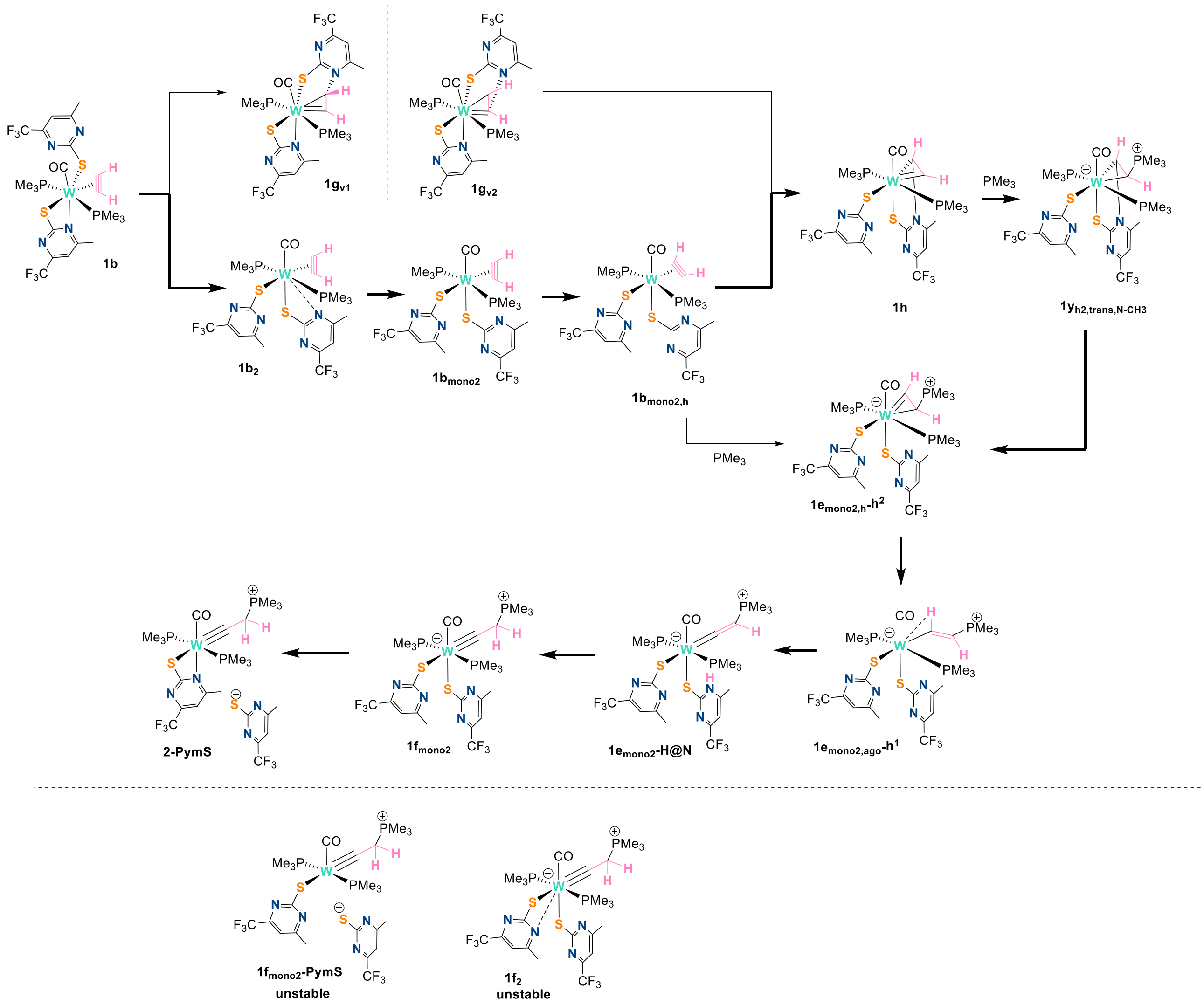

Scheme 4 Top: Overview of the investigated pathways starting from **1b** described in section 2.2.4., favored pathway is marked with bold arrows. Bottom: Hypothetical species **1f$_{mono2}$-PymS** and **1f$_2$** that could potentially have been formed from **1f$_{mono2}$** and, thus, played a role in the final steps but proved unstable under geometry optimization.

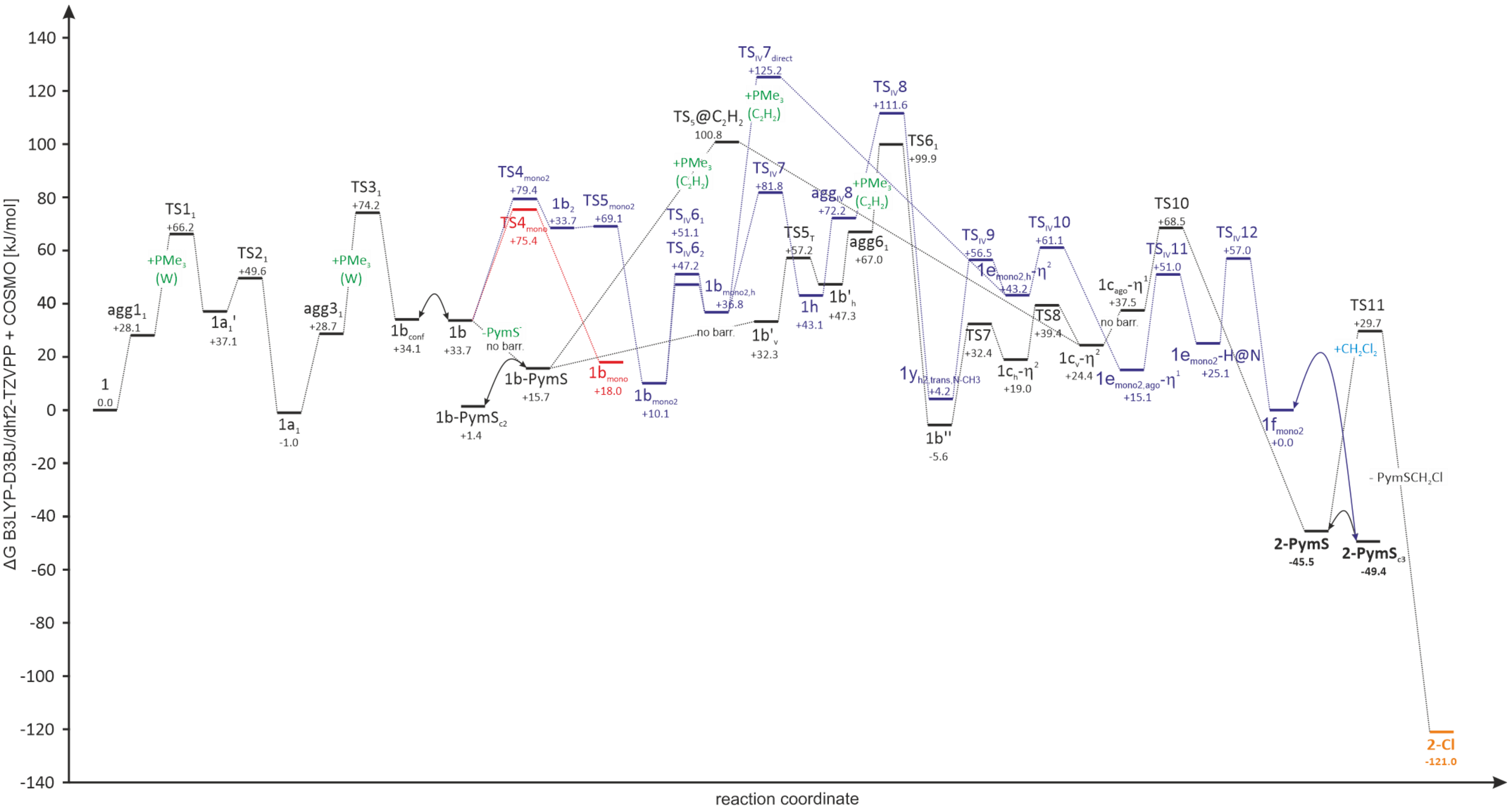

Diagram 4 Energy diagram of the pathways as described in section 2.2.4. (shown in violet). Steps until **1b** as described in section 2.1., the initial steps of the monodentate pathway outlined in section 2.2.2. and the pathways constituting our final mechanistic proposal (section 2.2.3) are left in for comparison.

Analogous to the mechanistic steps following the formation of **1b-PymS** in our final mechanistic proposal (section 2.2.3), the next step from **1b**$_{\mathbf{mono2,h}}$ must be attack of either PMe_3 or **PymS**$_{\mathbf{eq}}$ on the acetylene ligand (which is now enabled due to the latter being oriented horizontally). Also here, attack of PMe_3 leading directly to **1e**$_{\mathbf{mono2,h}}$**-η**$^{\mathbf{2}}$ (ΔG = +43.2 kJ/mol, a constitutional isomer of **1e**$_{\mathbf{mono,v}}$**-η**$^{\mathbf{2}}$, section 2.2.3.) displays a much higher energetic barrier ($\Delta\Delta G^{TS}$ = +88.4 kJ/mol) than attack of the PymS ligand ($\Delta\Delta G^{TS}$ = +44.3 kJ/mol) yielding complex **1h** (ΔG = +43.1 kJ/mol). The metallacyclopropene moiety of **1h** would then subsequently be attacked by PMe_3, thus forming **1y**$_{\mathbf{h2,trans,N\text{-}CH3}}$ (ΔG = +4.2 kJ/mol). Elimination of **PymS**$_{\mathbf{eq}}$ from the newly formed metallacyclopropane of **1y**$_{\mathbf{h2,trans,N\text{-}CH3}}$ then would lead to **1e**$_{\mathbf{mono2,h}}$**-η**$^{\mathbf{2}}$. Overall, similar to the mechanism *via* **1x**$_{\mathbf{h2,trans}}$, this sequence of steps leading to the formation of the required η^2-complex **1e**$_{\mathbf{mono2,h}}$**-η**$^{\mathbf{2}}$ that is poised to undergo hydrogen shift to the carbyne also constitutes a detour when compared to direct attack of PMe_3 at **1b**$_{\mathbf{mono2,h}}$ which leads to a lowering of the overall energy required, suggesting a catalytic role of the PymS-ligand (for a more detailed discussion see section 2.2.3.1.).

Importantly, the highest energy barrier that must be overcome in the pathway leading from **1b** to **1x**$_{\mathbf{h2,trans}}$ and further to η^2-complex **1c**$_{\mathbf{v}}$**-η**$^{\mathbf{2}}$ is +52.6 kJ/mol (attack of PMe_3 at **1b'**$_{\mathbf{h}}$), whereas the highest energy barrier occurring in the pathway leading to the formation of **1y**$_{\mathbf{h2,trans,N\text{-}CH3}}$ from **1b** and further to η^2-complex **1e**$_{\mathbf{mono2,h}}$**-η**^{2} is +68.5 kJ/mol (attack of PMe_3 at **1h**). Likewise, the absolute energies of the respective highest transition states involved are +99.9 and +111.6 kJ/mol, respectively. These data further indicate that the pathway *via* **1y**$_{\mathbf{h2,trans,N\text{-}CH3}}$ is disfavored over the one *via* **1x**$_{\mathbf{h2,trans}}$.

Following the formation of **1e**$_{\mathbf{mono2,h}}$**-η**$^{\mathbf{2}}$, the η^2-complex could readily convert further to its η^1-congener **1e**$_{\mathbf{mono2,ago}}$**-η**$^{\mathbf{1}}$, which again, similar to **1c**$_{\mathbf{ago}}$**-η**$^{\mathbf{1}}$ (section 2.2.3), exhibits agostic interactions between the tungsten center and the C_α-H bond. Subsequently, ligand mediated hydrogen shift to the (still monodentate) carbyne **1f**$_{\mathbf{mono2}}$ (ΔG = +0.0 kJ/mol) could occur, where the aromatic nitrogen of (still axially bound) **PymS**$_{\mathbf{eq}}$ would abstract the H_α.

The last step of this mechanism would then be expulsion of **PymS**$_{\mathbf{eq}}$ and recoordination of **PymS**$_{\mathbf{ax}}$ to yield the final carbyne product **2-PymS**$_{\mathbf{c3}}$ (ΔG = -49.4 kJ/mol). Several possibilities for this step arise. One pathway, analogous to the formation of **2-PymS**$_{\mathbf{c}}$ from **1f**$_{\mathbf{mono}}$ *via* **1f** (compare the monodentate pathway in section 2.2.2), would be reordination of **PymS**$_{\mathbf{ax}}$, yielding **1f**$_{\mathbf{2}}$ (Scheme 4), followed by decoordination of **PymS**$_{\mathbf{eq}}$. This route could, however, be ruled out, as **1f**$_{\mathbf{2}}$ proved unstable when attempting to optimize the geometry. Similarly, initial expulsion of **PymS**$_{\mathbf{eq}}$, yielding ion-pair **1f**$_{\mathbf{mono2}}$**-PymS** featuring a five-coordinate cationic complex (Scheme 4), could be discarded as no stable geometry for the ion-pair could be obtained. Therefore we expect a concerted mechanism where **PymS**$_{\mathbf{eq}}$ is expulsed simultaneously to the recoordination of **PymS**$_{\mathbf{ax}}$.

Overall, while the final steps of the mechanism *via* of **1y**$_{\mathbf{h2,trans,N\text{-}CH3}}$ also offer plausible explanation for the hydrogen shift leading to carbyne formation, the facts that the initial formation of **1b**$_{\mathbf{mono2}}$ is strongly disfavored over the generation of **1b-PymS** and that the subsequent steps boast much higher energetic barriers (Diagram 4) leads to the conclusion that pathway *via* **1y**$_{\mathbf{h2,trans,N\text{-}CH3}}$ can ultimately be ruled out.

2.2.5. *Pathway starting by attack of PMe₃ at **1a₂***

While formation of **1a'₂** presents a kinetically disfavored pathway, contrary to the favored pathway via **1a'₁**, the hypothetical subsequent attack of PMe_3 at the coordinated C_2H_2 of **1a₂** yielding **1j$_v$-η^2** displays a similar energy barrier as the attack at the tungsten center that would lead to **1b** (see section 2.1.2.). Despite its disfavored nature, this pathway is of interest as it opens a route to new intermediates which constitute the neutral analogues of the cationic species involved in our final mechanistic proposal, which can be used to gain additional insight into the factors at play in the reaction at hand (section 4.). The pathways described in this section are summarized in Scheme 5, whereas the corresponding energy diagram is displayed in Diagram 5.

Thus, **1j$_v$-η^2** constitutes the neutral analogue of **1c$_v$-η^2**, as the latter could theoretically be formed by ligand exchange of **PymS$_{eq}$** for PMe_3 in **1j$_v$-η^2**. However, such a ligand exchange would need to take place by initial addition of PMe_3 to **1j$_v$-η^2** (as direct loss of $PymS^-$ anion would yield an unstable five-coordinate complex), forming an intermediate of type **1e$_v$-η^2**, the geometry of which could not be optimized as it proved unstable (section 2.2.1). This would prevent direct conversion of **1j$_v$-η^2** to **1c$_v$-η^2**.

Following the formation of **1j$_v$-η^2**, it could readily convert further to its η^1-congener **1j$_{ago}$-η^1**, the neutral analogue of **1c$_{ago}$-η^1**, where, again, the latter could theoretically be formed from **1j$_{ago}$-η^1** by a ligand exchange of **PymS$_{eq}$** for PMe_3. Indeed, addition of PMe_3 to the tungsten center of **1j$_{ago}$-η^1** gives rise ($\Delta\Delta G^{TS}$ = +55.3 kJ/mol) to complex **1e$_3$-η^1** [a congener of **1e$_1$-η^1** and **1e$_2$-η^1** investigated within the context of the monodentate mechanism (section 2.2.2.)], which could undergo loss of $PymS^-$ anion to give **1c$_{ago}$-η^1**. Similar to **1c$_{ago}$-η^1**, **1j$_{ago}$-η^1** exhibits agostic interactions between the metal center and the C-H$_\alpha$ bond.

1j$_{ago}$-η^1 could also undergo ligand-mediated hydrogen shift towards carbyne **1k**, the neutral analogue of **2-PymS**. Here, contrary to the thiolate mediated hydrogen shift in the cationic mechanism (from **1c$_{ago}$-η^1** to **2-PymS**), the hydrogen would be transferred by a nitrogen atom of **PymS$_{eq}$**. However, the energy barrier that needs to be overcome for formation of **1k** ($\Delta\Delta G^{TS}$ = +82.7 kJ/mol) is considerably larger than the one for transformation to **1e$_3$-η^1** ($\Delta\Delta G^{TS}$ = +55.3 kJ/mol) and, foreseeably, also the one onward to **1c$_{ago}$-η^1**. It also is larger than the barrier for the subsequent hydrogen-shift in the cationic mechanism ($\Delta\Delta G^{TS}$ = +31.0 kJ/mol, **TS10** from **1c$_{ago}$-η^1** to **2-PymS**). The same picture is obtained when comparing the absolute energies of the involved transition states (Diagram 5). This presents another reason that the neutral pathway is kinetically not competitive with the cationic one constituting our main mechanistic proposal (section 2.2.3.). Moreover, none of the involved species could be detected experimentally (compare the calculated NMR shifts for **1e$_1$-η^1** and **1e$_2$-η^1**, the congeners of **1e$_3$-η^1** (Table 2, section 2.2.2.).

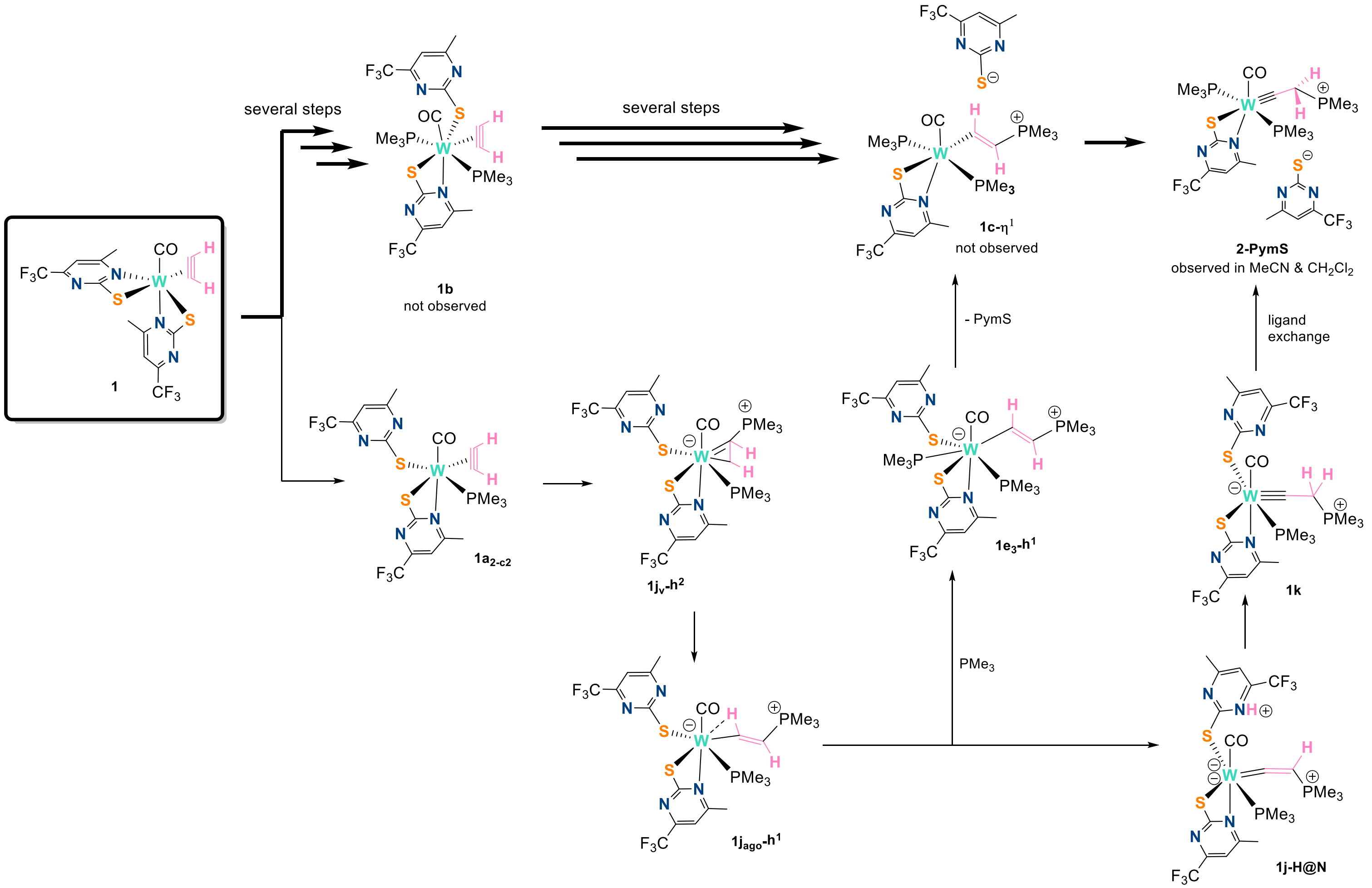

Scheme 5 Overview of the hypothetical neutral side-pathway starting from **1a₂** (section 2.2.5.) in comparison to our final mechanistic proposal (marked with bold arrows, section 2.2.3.).

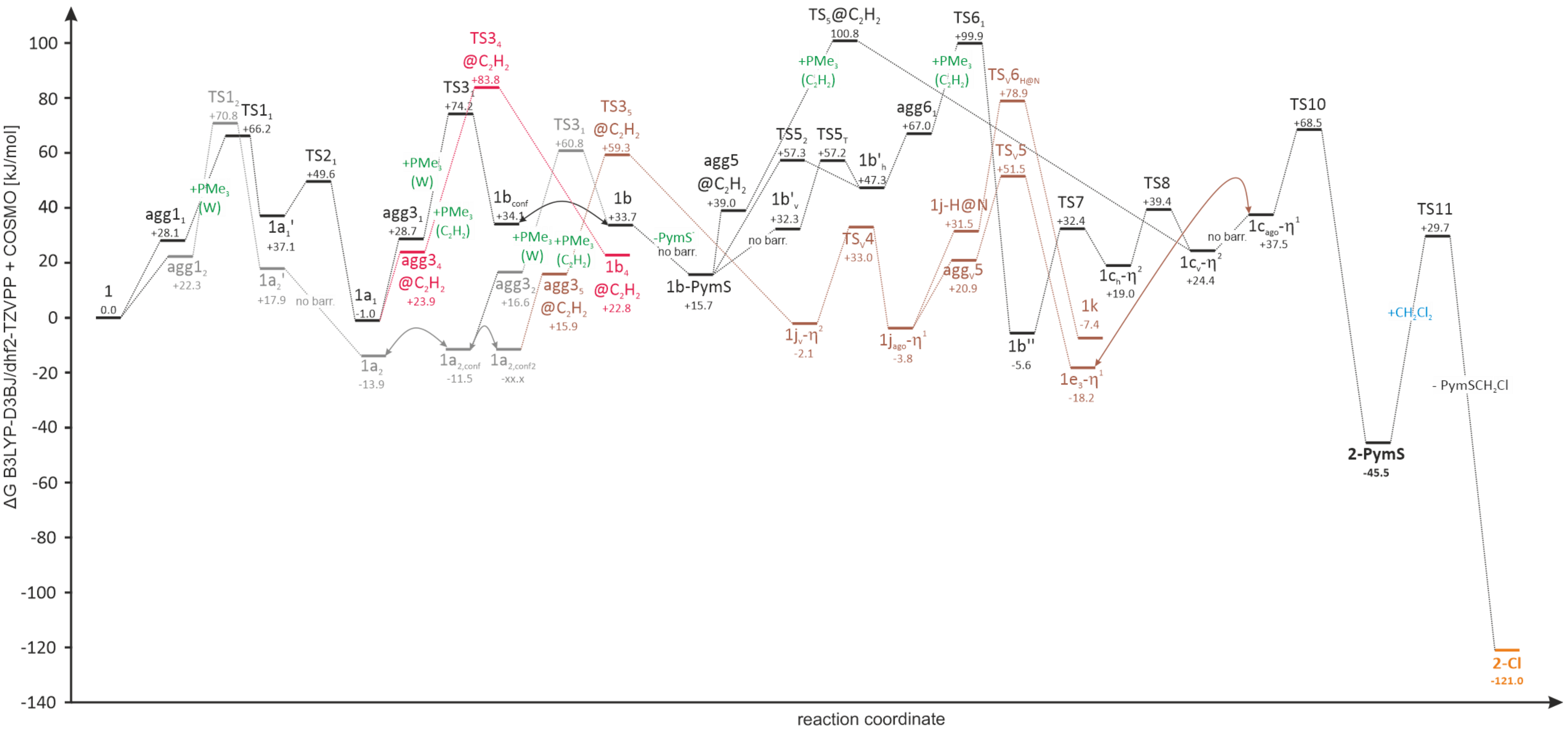

Diagram 5 Energy diagram of the hypothetical neutral side-pathway starting from **1a$_2$** (brown, section 2.2.5.), steps until **1b** as described in section 2.1. and the pathways constituting our final mechanistic proposal (section 2.2.3) are left in for comparison.

3. Detailed discussion of the computational investigations on the oxo-mechanism

In the following, we give a more detailed description of our computational investigations of the possible reaction pathways of the transformation of **3** to **4-Cl**. The pathways and corresponding energy diagrams described in this section are summarized in Scheme 6 and Diagram 6, which outline the pathways until the formation of **3c**, and Scheme 7 and Diagram 7, which depict the subsequent steps towards the formation of **4-PymS**.

With **3a** as the first experimentally identified candidate for a likely reaction intermediate, we started our investigations from there. Two steps are required for the formation of **3a**, namely coordination of PMe_3 to the tungsten center and decoordination of the nitrogen atom of one of the PymS ligands. Consequently, this leads to either an associative or dissociative mechanism, where, as could be expected, the latter was determined to be highly disfavored. Decoordination of the nitrogen of the equatorial PymS ligand of **3** (**$PymS_{eq}$**) yields high-energy five-coordinate complex **$3_{Ndiss.}$** (ΔG = +50.0 kJ/mol), while any attempt to optimize a complex featuring decoordination of the nitrogen of the axial PymS ligand of **3** (**$PymS_{ax}$**) failed since the geometries revert back to **3**.

Contrary, the associative mechanism is predicted to easily occur ($\Delta\Delta G^{TS}$ = +63.3 kJ/mol) with attack of PMe_3 proceeding from the "top" (Figure 6), i.e., from the direction of the oxo-ligand, leading to the formation of complex **$3a'_1$** (ΔG = +20.8 kJ/mol). Attack from the "bottom", i.e., from the direction of the sulfur atom of **$PymS_{ax}$** can be excluded, since it results in high-energy constitutional isomer **$3a'_2$** (ΔG = +57.4 kJ/mol).

Additionally, the alternative possibility of attack of PMe_3 at the acetylene ligand of **3** was also investigated. Complex **3** exhibits four potential sites for an attack of PMe_3, giving rise to hypothetical products **$3a'_n@C_2H_2$** (Figure 5, n=1: right top; n=2: right bottom, n=3; left top; n=4: left bottom). The data (Table 4) indicate that this possibility can be ruled out due to the associated transition states and products exhibiting a much higher energy than the ones resulting from attack at tungsten. Hence, the DFT calculations predict the first step in the mechanism to be the attack of PMe_3 at the tungsten center, yielding intermediate **$3a'_1$**.

In **$3a'_1$**, attack of the second molecule of PMe_3 at C_2H_2, hypothetically yielding **$3b'@C_2H_2$**, can be ruled out due to the corresponding barrier being too large ($\Delta\Delta G^{TS}$ = +102.3 kJ/mol) which can be attributed to the crowded hepta-coordinate nature of **$3a'_1$**. Additionally, formation of **$3b'@C_2H_2$** would be highly endergonic (ΔG = 69.1 kJ/mol) due to the additional PMe_3 increasing the crowding in the already seven-coordinate complex **$3a'_1$** even further.

However, once **$3a'_1$** is formed, decoordination of the nitrogen of **$PymS_{ax}$** is predicted to occur barrierless, yielding the experimentally observed intermediate **3a** (ΔG = -2.2 kJ/mol). The small size of the barrier and the much higher stability of six-coordinate **3a** in comparison to hepta-coordinate **$3a'_1$** can easily be explained due to the reduction of the coordination number. Importantly, the transformation of **3** to **3a** *via* **$3a'_1$** leads to an overall rearrangement of the coordination shell with regard to the position of the PymS ligands. Whereas in complex **3** one PymS ligand is aligned axially (**$PymS_{ax}$**) and one horizontally (**$PymS_{eq}$**), **$3a'_1$**, due to its hepta-

coordinate nature, features **PymS$_{eq}$** oriented at an angle in between (Figure 7). Subsequent decoordination of the nitrogen of **PymS$_{ax}$** then leads to **PymS$_{ax}$** being coordinated to the tungsten center in a now equatorial position (which becomes free due to the decoordination of said nitrogen, compare Figure 7), whereas **PymS$_{eq}$** consequently reorients to be bound in an axial fashion. It is emphasized here that the naming scheme of **PymS$_{ax}$** and **PymS$_{eq}$** originates from the orientations of the respective PymS ligands in starting material **3** and merely serves to provide an easy way to both talk about and track them throughout the course of the mechanism. Furthermore, no stable minimum corresponding to the alternative decoordination of **PymS$_{eq}$** could be obtained. While the experimental data do not allow for the determination which of the two PymS ligands becomes monodentate, we therefore propose that it is indeed **PymS$_{ax}$** which becomes monodentate and is subsequently replaced by PMe_3 (*vide infra*).

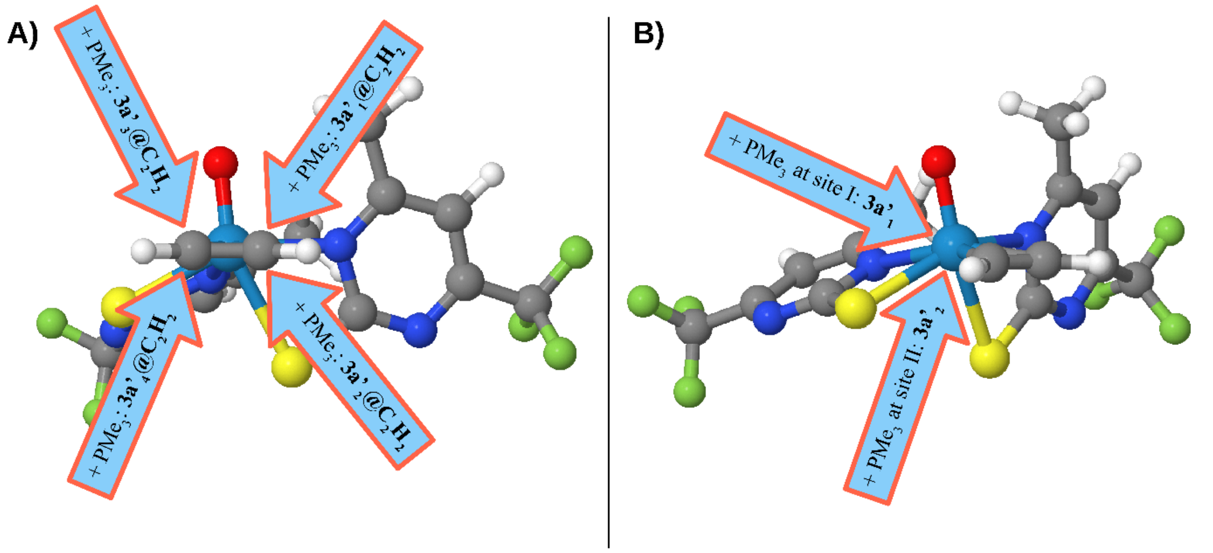

Figure 6 Investigated sites of attack/binding sites of PMe_3 (as indicated by arrows) at coordinated acetylene (A) and the tungsten (B) in **3**.

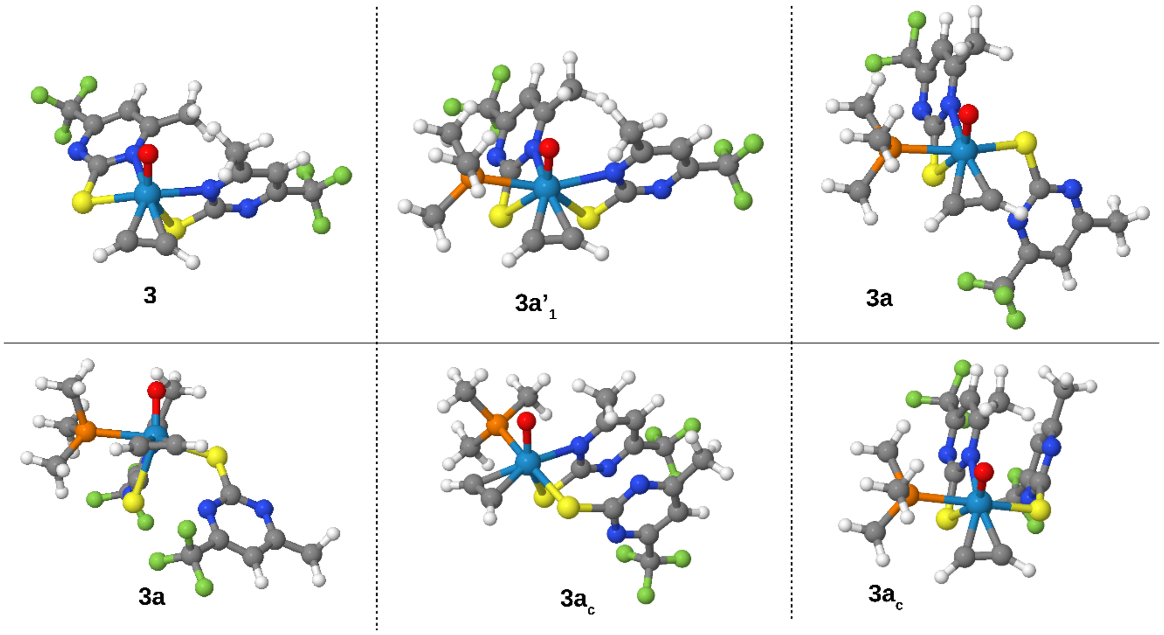

Figure 7 Comparison of the geometries of complexes **3a**, **3a'₁**, **3a** and **3a**$_c$. Two views of both **3a** and **3a**$_c$ are depicted for easier understanding of the geometric arguments outlined in the corresponding paragraphs.

Table 4 Gibbs free energies of the hypothetical products **3a'**$_n$**@**C_2H_2 formed by attack of PMe_3 at C_2H_2 in **3** and the associated transition states **TS1**$_n$**@**C_2H_2 and pre-reaction agglomerates **agg1**$_n$**@**C_2H_2. Additionally, the barrier heights ($\Delta\Delta G^{TS}$) associated with each transition state are given relative to the infinitely separated reactants.

n	**agg1**$_n$**@**C_2H_2	**TS1**$_n$**@**C_2H_2	$\Delta\Delta G^{TS}$	**3a'**$_n$**@**C_2H_2
1	+28.2	+88.2	+88.2	+40.4
2	+24.2	+91.3	+91.3	+39.1
3	+22.7	+86.0	+86.0	+42.4
4	+26.5	+94.0	+94.0	+54.2

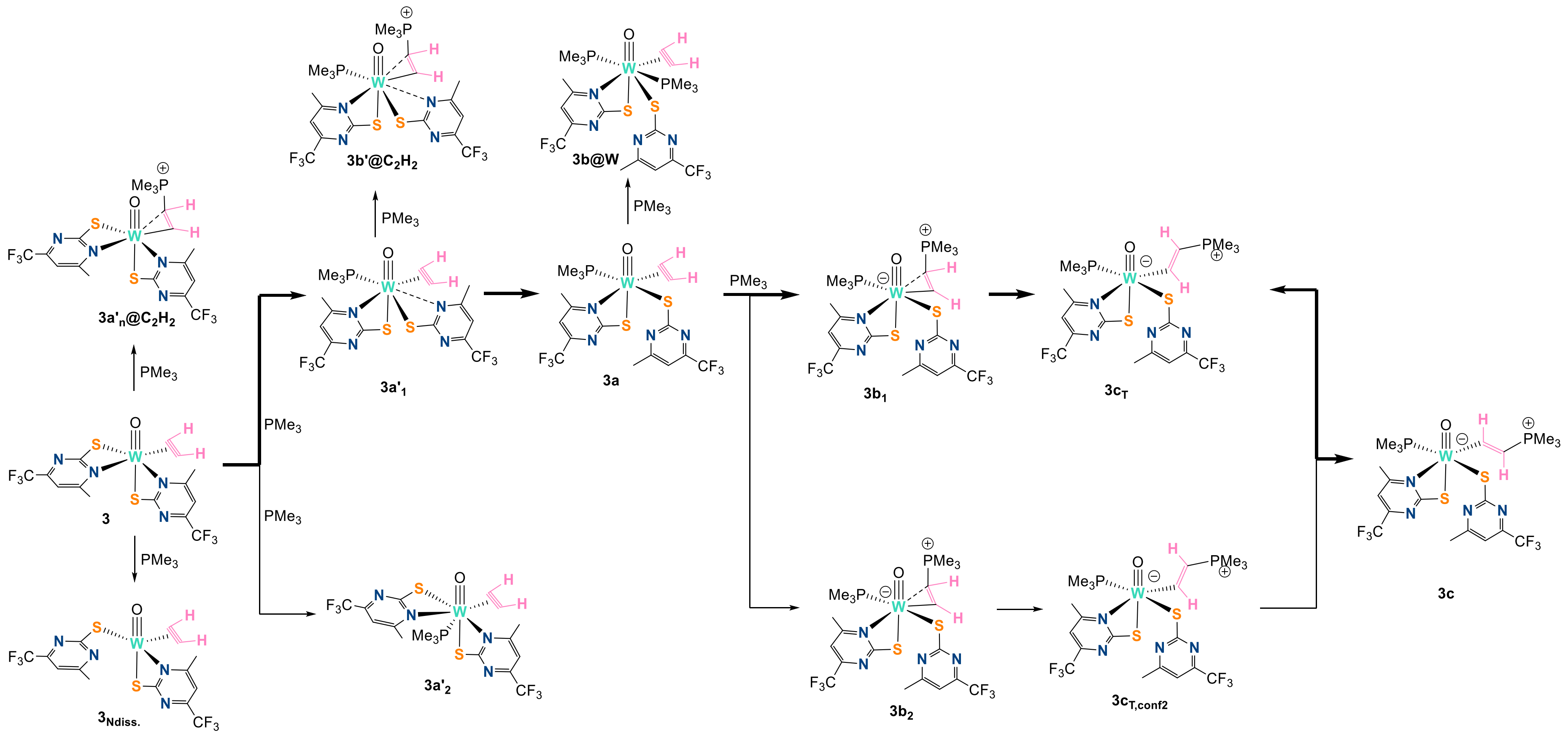

Scheme 6 Overview of the most important species and pathways discussed in this section until intermediate **3c**. The favored pathway is highlighted in bold.

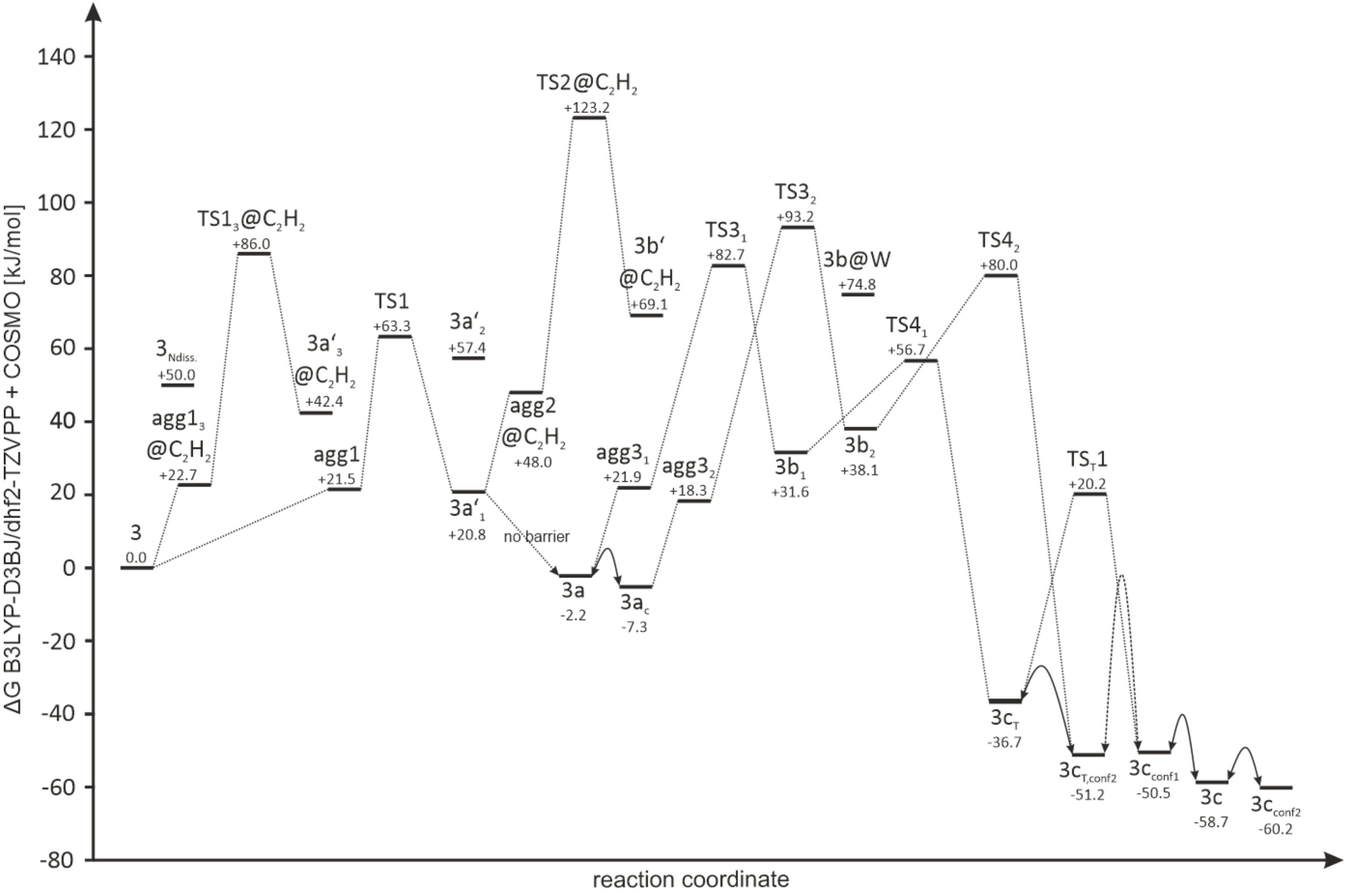

Diagram 6 Energy diagram of the oxo mechanism until intermediate **3c**.

In stark contrast to the mechanism of the carbonyl analogue, attack of a second PMe_3 at the tungsten center of **3a** is sterically highly disfavored, reflected in the high energy ($\Delta G = +74.8$ kJ/mol) of the corresponding hypothetical product **3b@W**. This is in line with the experimental data, as the observation of **3c** strongly suggests the nucleophilic attack to occur without coordination of a second PMe_3 to the tungsten center.

Therefore, the next step in the mechanism is attack of PMe_3 at the acetylene ligand, which, contrary to seven-coordinate **3a'$_1$**, becomes feasible for six-coordinate **3a**. While in **3a** attack of PMe_3 from the bottom is sterically blocked by **PymS$_{ax}$**, the latter exhibits a considerable degree of flexibility due to being bound in a monodentate fashion. This allows the formation of another conformation of **3a**, dubbed **3a$_c$** (Figure 7, $\Delta G = -7.3$ kJ/mol), which features **PymS$_{ax}$** tucked away from the coordinated C_2H_2. Therefore, PMe_3 can attack the acetylene ligand from four directions (compare Figure 6), yielding different potential configurations **3b$_n$** of the resulting η^2-vinyl complex (Figure 8, n=1: right top; n=2: right bottom, n=3; left top; n=4: left bottom). Attack from directions 1 and 2 would occur from the side of **PymS$_{ax}$** (yielding **3b$_1$** and **3b$_2$**, respectively), while attack along trajectories 3 and 4 would take place from the side of the already bound PMe_3 (yielding **3b$_3$** and **3b$_4$**, respectively). Calculating all four possibilities reveals that attack from directions 1 and 2 is both kinetically and thermodynamically preferable over attack along trajectories 3 and 4 (Table 5). This can be rationalized in terms of steric hindrance, as the already bound PMe_3 hinders attack along the latter and also raises the energy of the corresponding η^2-vinyl complexes. Overall, trajectory 1 features the lowest barrier ($\Delta\Delta G^{TS} = +85.0$ kJ/mol) and the lowest energy product **3b$_1$** ($\Delta G = +31.6$ kJ/mol, referred to as **3b** in the main text), and is, hence, favored over the three other possibilities.

The thus far described findings closely match the experimental observations, as the experimental observation of **3a** can readily be explained: The barrier associated with the formation of **3b$_1$** is larger than the one associated with the formation of **3a'/3a**, leading to an accumulation of **3a** in the reaction vessel (this is reminiscent of the carbonyl-mechanism, section 2.1.).

After the formation of η^2-vinyl complex **3b$_1$** (referred to as **3b** in the main text), addition of another molecule PMe_3 is sterically prevented. Therefore, **3b$_1$** is predicted to convert to its η^1-vinyl counterpart **3c$_T$** ($\Delta G = -36.7$ kJ/mol). In **3c$_T$**, the CF_3 group of the monodentate PymS ligand faces the η^1-vinyl moiety. Therefore, at least two more conformers of **3c$_T$** could exist (Figure 9): Turning the monodentate PymS ligand by 180° around an axis running through the C-S bond would yield **3c$_{T,c1}$**, while tucking it away from the coordinated C_2H_2 would give rise to **3c$_{T,c2}$**. While we were unable to optimize **3c$_{T,c1}$**, **3c$_{T,c2}$** features a considerably lower energy than **3c$_T$** of -49.2 kJ/mol and must be expected to exist in equilibrium with **3c$_T$**. Overall, the formation of **3c$_T$/3c$_{T,c2}$** from **3a** is driven by the exergonicity of this step.

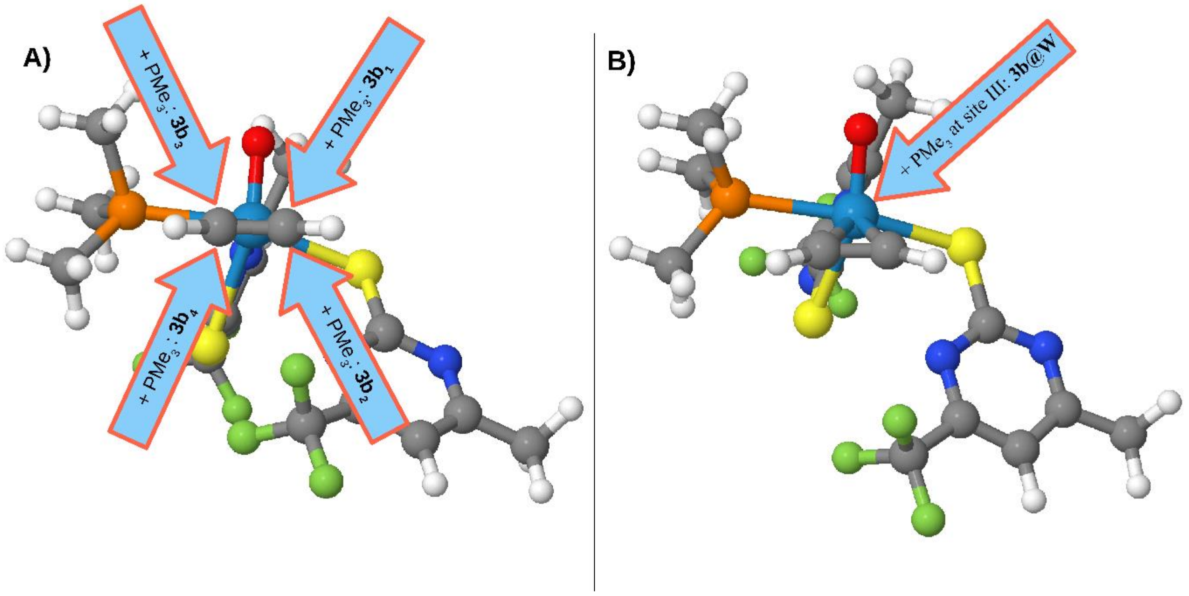

Figure 8 Investigated sites of attack/binding sites of PMe_3 (as indicated by arrows) at coordinated acetylene (A) and the tungsten (B) in **3a₁**.

Table 5 Gibbs free energies of complexes **$3b_n$** formed by attack of PMe_3 at C_2H_2 in **3a** and the associated transition states **$TS3_n$** and pre-reaction agglomerates **$agg3_n$**. Additionally, the barrier heights ($\Delta\Delta G^{TS}$) associated with each transition state are given relative to the infinitely separated reactants.

n	**$agg3_n$**	**$TS3_n$**	**$\Delta\Delta G^{TS}$**	**$3b_n$**
1	+21.9	+82.7	+85.0	+31.6
2	+18.3	+93.2	+100.5	+38.1
3	n.a.	n.a.	n.a.	+56.4
4	+29.0	+111.0	+113.3	+52.5

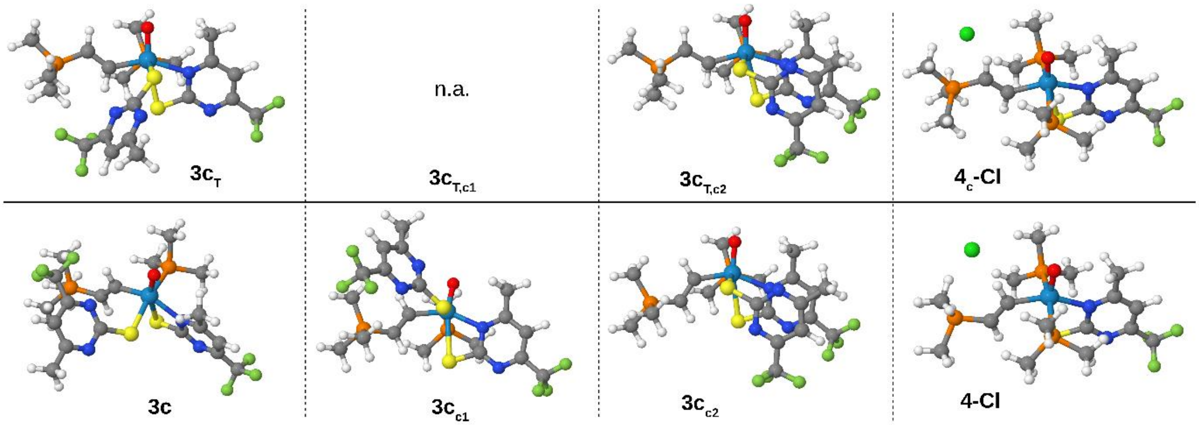

Figure 9: Top: Rendered 3D-structures of **3c$_{T}$** and its conformer **3c$_{T,c2}$** alongside carbyne **4$_{c}$-Cl**. Bottom: Rendered 3D-structures of **3c** and its conformers **3c$_{c1}$** and **3c$_{c2}$** alongside carbyne **4-Cl**.

Interestingly, after hypothetical formation of **3b$_{2}$** (disfavored over formation of **3b$_{1}$**, compare Table 5), the latter could directly convert to **3c$_{T,c2}$**. However, in addition to formation of **3b$_{2}$** being disfavored over **3b$_{1}$**, the barrier associated with the transformation of **3b$_{2}$** to **3c$_{T,c2}$** is also considerably higher ($\Delta\Delta G^{TS}$ = +41.9 kJ/mol) than the one for **3b$_{1}$** to **3c$_{T}$** ($\Delta\Delta G^{TS}$ = +25.0 kJ/mol). This completes the picture that the route *via* **3b$_{1}$** constitutes the favored pathway for formation of **3c$_{T}$**/**3c$_{T,c2}$**.

Importantly, both **3c$_{T}$** and **3c$_{T,c2}$** feature the η^1-vinyl group turned by 180° compared to the X-ray structure of product **4-Cl** (Figure 9), as indicated by the upper case subscript “T”. The corresponding complexes **3c**, **3c$_{c1}$** and **3c$_{c2}$** (compare Figure 9), which feature an orientation of the η^1-vinyl group akin to the X-ray structure, exhibit overall considerably lower energies than **3c$_{T}$** and **3c$_{T,c2}$** (Table 6). This suggests that the orientation of the η^1-vinyl group as determined by X-ray is the thermodynamically more stable one. However, despite our best efforts, all attempts to locate transition states leading from **3b$_{1}$** and **3b$_{2}$** to such a complex failed. Therefore, we calculated the energy required for the turning of the η^1-vinyl group, which were found to be low enough for the conformational change to be feasible to occur. Rotation of the η^1-vinyl group in **3c$_{T}$** yields **3c$_{c1}$** *via* a barrier of only +56.9 kJ/mol (if measured from the lowest energy conformer **3c$_{T,c2}$**, this barrier increases to +71.4 kJ/mol). Hence, depending on the heights of the energetic barriers of the remaining mechanistic steps, it may be either **3c** or **3c$_{T}$** which converts further into **4-PymS**.

Table 6 Gibbs free energies of **3c**, **3c$_{T}$** and their conformers

complex	ΔG	complex	ΔG
**3c**	-58.7	**3c$_{T}$**	-36.7
**3c$_{c1}$**	-50.5	**3c$_{T,c1}$**	n.a.

**$3c_{c2}$**	-60.2	**$3c_{T,c2}$**	-51.2

Proceeding to the final stages of the mechanism, several possibilities for the formation of **4-Cl** from **3c** or **$3c_T$** could occur. A dissociative mechanism where the monodentate pyrimidine ligand is pushed out from **$3c/3c_T$** can be discarded due to the electron-poor nature of the hypothetically resulting penta-coordinate species (in analogy with the initial dissociation of the nitrogen of a PymS ligand from **3** being highly unfavorable). Likewise, reaction of monocoordinated **$PymS_{ax}$** in its bound state with CH_2Cl_2 would not be in line with the experimental observation of ion pair **4-PymS**.

The experimental data can, however, very well be explained by an associative mechanism, where a third molecule of PMe_3 coordinates to the tungsten center of **3c** or **$3c_T$** first, forming intermediate **3d** or **$3d_T$**, respectively, without any additional energetic barrier. Once formed, **3d** undergoes loss of the thiolate ligand without any additional energetic barrier, thereby forming ion par **$4\text{-}PymS_{c2}$** and **4-PymS**, which only differ in the relative location of the decoordinated PymS ligand (**$PymS_{ax}$**). Likewise, any formed **$3d_T$** can undergo barrierless loss of the monocoordinated PymS ligand, forming **4_T-PymS**.

Formation of **$3d_T$** from **$3c_T$** displays a significantly higher energetic cost ($\Delta\Delta G^{TS}$ = +84.8 kJ/mol,] compared to the barrier that needs to be overcome for the rotation of the η^1-vinyl group converting **$3c_T$** to **$3c_{c1}$** ($\Delta\Delta G^{TS}$ = +56.9 kJ/mol). Since formation of **3d** from **3c** also displays a higher energetic cost ($\Delta\Delta G^{TS}$ = +116.3) compared to the back-conversion of **3c** to **$3c_T$** ($\Delta\Delta G^{TS}$ = +78.9 kJ/mol), **$3c_T$** and **3c** as well as their conformers can be regarded to exist in equilibrium. Hence, comparing the overall barrier heights relative to the lowest energy conformer **$3c_{c2}$** of the two transformations of **3c** to **3d** and **$3c_T$** to **$3d_T$** one obtains a $\Delta\Delta G^{TS}$ of +117.8 kJ/mol and +108.4 kJ/mol, respectively. These data suggest formation of a mixture of both **4-PymS** and **4_T-PymS**, where, due to the equilibrium strongly favoring **3c** due to its lower relative energy which supposedly leads to its accumulation during the reaction, a preference for the pathway leading from **3c** to **4-PymS** *via* **3d** can be argued. Moreover, since **4-PymS** and **4_T-PymS** differ only in the orientation of the η^1-vinyl group (the relative location of decoordinated **$PymS_{ax}$** ligand is the same) and exhibit approximately the same energy (**4-PymS**: ΔG = -38.9 kJ/mol, **4_T-PymS**: ΔG = -35.2 kJ/mol), the two forms can in any case be expected to equilibrate by direct interconversion *via* rotation of the η^1-vinyl group. Additionally, the back-reaction is also viable, where (upon their formation from **4-PymS** and **4_T-PymS**, respectively) **3d** and **$3d_T$** can also undergo loss of PMe_3 to restore **3c** and **$3c_T$**, respectively. Therefore, and due to the comparable energies of **$3c_{T,c2}$** (ΔG = -51.2), **$3c_{c2}$** (ΔG = -60.2), **4_T-PymS** (ΔG = -35.2) and **4-PymS** (ΔG = -38.9), the data overall predict the formation of a mixture of these four species in the reaction vessel. This is consistent with experimental observations, where no full conversion to **4-PymS/4_T-PymS** can be observed.

Using CH_2Cl_2 as a solvent, the $PymS^-$ anion in **4-PymS** can undergo further reaction to **4-Cl** and 4,6-CF_3MePymSCH_2Cl ($\Delta\Delta G^{TS}$ = +75.2 kJ/mol). As described for the carbonyl-

mechanism (section 2.2.3.), the corresponding reaction of the $PymS^-$ anion with CH_2Cl_2 was determined to require +75.2 kJ/mol. Thus, the barrier for the transformation of **4-PymS/4$_T$-PymS** to **4-Cl/4$_T$-Cl** and 4,6-CF_3MePymSCH_2Cl was taken to be +75.2 kJ/mol.

While it is entirely plausible a mixture of both **4-Cl/4$_T$-Cl** is formed initially, rotation of the η^1-vinyl group in **4$_T$-Cl** can commence *via* a low energetic barrier of +42.6 kJ/mol to give **4-Cl**. This rotation occurs even more readily than the corresponding transformation in **3c$_T$** ($\Delta\Delta G^{TS}$ = +56.9 kJ/mol), which can easily be rationalized by the rotation in **4$_T$-Cl** being less impaired by steric influences since the thiolate ligand is now replaced with PMe_3. This difference in the rotational barriers is further corroborated by NMR spectra of both **3c** and **4-Cl**, where the ethenyl protons of the latter display a much stronger dynamic behavior. Together with the energy gained by reorienting the η^1-vinyl ligand from **4$_T$-Cl** to **4-Cl** ($\Delta\Delta G$ = -15.6 kJ/mol), this provides an explanation why **4-Cl** is the conformer that crystallizes and is, thus, captured in the X-ray structure.

Overall, the reaction is driven towards formation of **4-Cl** due to the exergonic nature of the overall transformation from **3c** to **4-Cl**. However, the relatively large size of the energetic barriers that need to be overcome (around 110 kJ/mol) in the last steps of the mechanism well match the experimental observation that even after 22h the reaction of **3** with 3.3 equiv. of PMe_3 did not show full conversion.

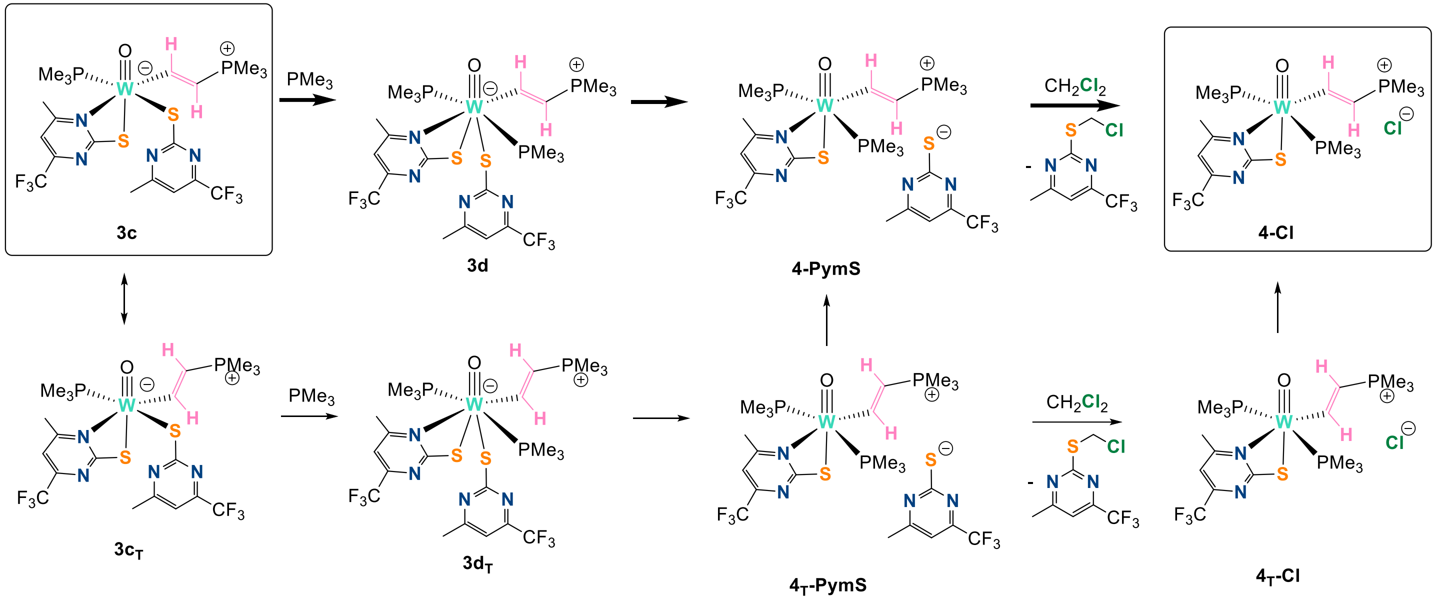

Scheme 7 Overview of the most important species and pathways leading to the formation of the final product **4-Cl** following the formation of intermediate **3c**. The favored pathway is highlighted in bold.

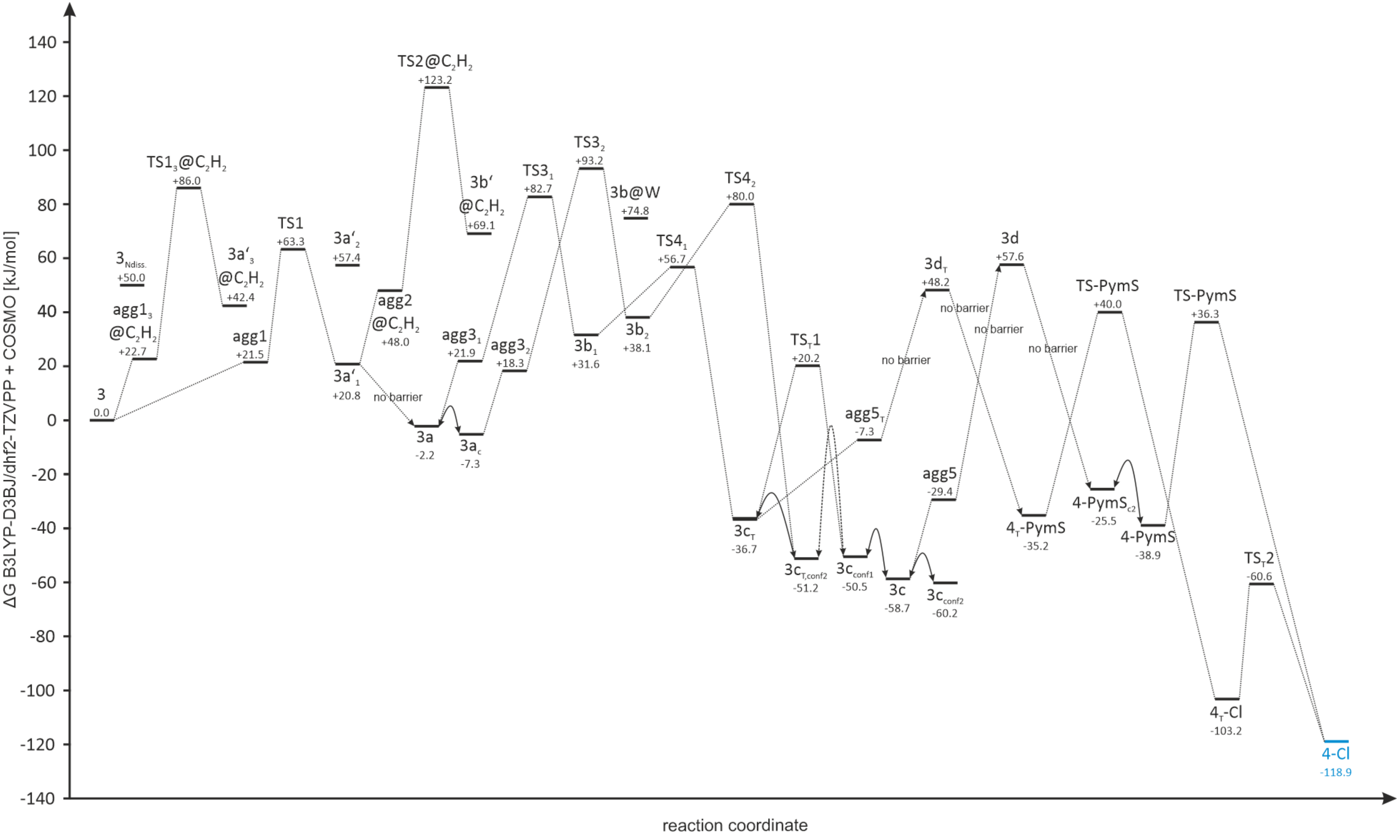

Diagram 7 Energy diagram of the full oxo mechanism as outlined in this section

4. Additional Details regarding "Discussion and Rationalization of the different behavior of the two congeneric mechanisms."

4.1. Why are the final products of the respective reactions thermodynamically preferable? What is the role of the cation in the carbonyl mechanism?

As detailed in the main text, oxo-species **3b** (η^2) and **3c** (η^2) display a different coordination sphere compared to carbonyl complexes **1c$_v$-η^2** and **1c$_{ago}$-η^1**. **3b** and **3c** are formed after reaction with the first molecule of PMe_3 and therefore contain one PMe_3 and one monodentate PymS ligand coordinated to tungsten each. Contrary, in **1c$_v$-η^2** and **1c$_{ago}$-η^1** the monodentate PymS ligand has already subsequently been replaced by the second PMe_3. Whereas the former coordination pattern results in neutral complexes, the latter renders them ion pairs containing cationic metal complexes (Figure 10). Since, intuitively, a tungsten center bound in a cationic complex would be expected to exhibit a diminished tendency toward electron donation, we were curious about the role the of cation formation in the carbonyl mechanism. Therefore, we also examined the hypothetical neutral analogues **1j$_v$-η^2**, **1j$_{ago}$-η^1** and **1k** (carbyne) of carbonyl complexes **1c$_v$-η^2**, **1c$_{ago}$-η^1** and **2-PymS** which could be obtained by changing the respective coordination pattern of the latter and also appear in the context of the hypothetical neutral side-pathway starting from **1a$_2$** (section 2.2.5.). Intriguingly, this revealed the tungsten centers in the cationic complexes to be moderately more electron rich (Table 7). In accordance, **1j$_{ago}$-η^1** exhibits a significantly less pronounced increase in W-C_α bond order than **1c$_{ago}$-η^1** (1.318 *vs.* 1.562) as well as a comparable reduction of the C_α=C_β double bond character (1.475 *vs.* 1.480) and linearization of the W-C_α-C_β bond angle (170.6° *vs.* 169.0°). **1j$_v$-η^2** and **1j$_{ago}$-η^1** are of similar stability ($\Delta\Delta G$ = +1.7 kJ/mol) and **1k** is only -3.6 kJ/mol more stable than **1j$_{ago}$-η^1**, indicating no particular preference for either the η^1, η^2 or carbyne forms. Hence, this triad of complexes (**1j$_v$-η^2**, **1j$_{ago}$-η^1**, **1k**) lies in between the trends discussed in the main text for the oxo and cationic carbonyl mechanism, where the η^1-form is either strongly favored or disfavored. This is consistent with only a slight decrease of the W-C=O bond order in both **1j$_v$-η^2** and **1k** compared to **1j$_{ago}$-η^1** (1.255 *vs.* 1.280 *vs.* 1.340) and the lower degree of electron donation onto the η^1-ligand.

Table 7 partial charges (NBO) of the tungsten center in the various investigated complexes

		η^2	η^1	$\Delta\Delta G$	η^1	carbyne	$\Delta\Delta G$
CO	cationic	-0.013 (**1c$_v$-η^2**)	-0.187 (**1c$_{ago}$-η^1**)	+13.1	-0.173 (**1c$_{ago}$$^+$-$\eta^1$**)	-0.089 (**2$^+$**)	-82.1
	neutral	+0.138 (**1j$_v$-η^2**)	+0.007 (**1j$_{ago}$-η^1**)	+1.7	+0.007 (**1j$_{ago}$-η^1**)	+0.036 (**1k**)	-3.6
oxo	cationic	---	---	---	+0.882 (**4$^+$**)	+0.756 (**4x$^+$**)	+71.2
	neutral	+1.020 (**3b**)	+1.000 (**3c**)	-90.3	---	---	---

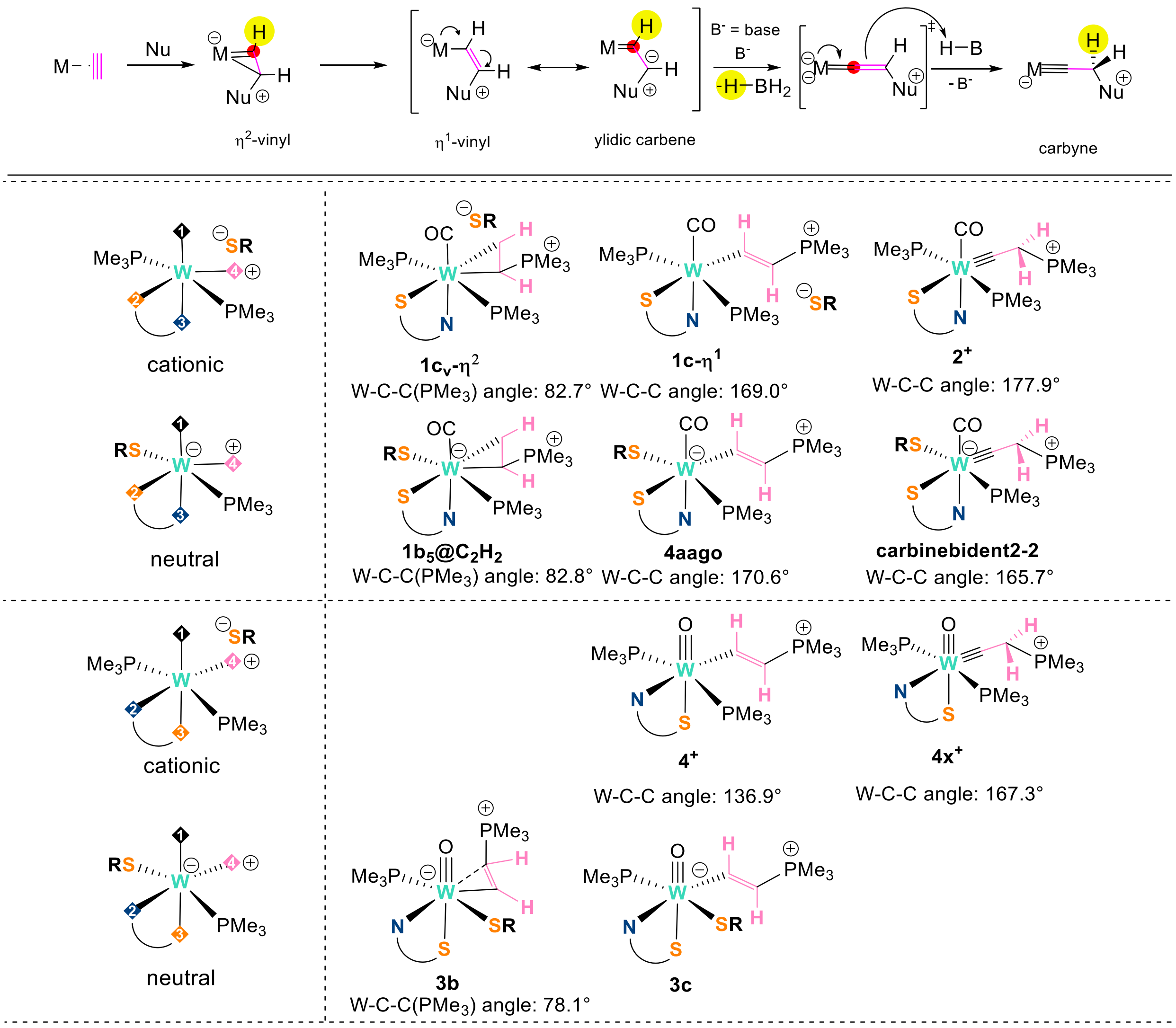

Figure 10 Top: Arrow pushing mechanism of the transformation of a general acetylene complex to the carbyne *via* a vinylidene transition state involving the two resonance structures of the η^1-vinyl species. Bottom: The various η^1-, η^2- and carbyne complexes investigated for the oxo and carbonyl mechanisms ordered by their respective coordination environment. W-C-C angles are given below structures.

4.2. Why is the hydrogen shift leading to the carbyne kinetically feasible?

The importance of the enhancement of the electron donation of the metal center due to the formation of the cation is further supported by the much less pronounced charge separation of the neutral η^1-congener $\mathbf{1j_{ago}}$-$\boldsymbol{\eta^1}$ (C_α: -0.286, C_β: -0.877).

5. Verification of the method employed to calculate NMR shifts

In order to verify our method used for the calculation of the NMR shifts (RI-PBE-D3BJ/dhf-SVP), we compared the corresponding chemical shifts obtained for complex **1** to the ones obtained at higher levels of theory (RI-PBE-D3BJ/dhf-TZVPP and RIJK-B3LYP-D3BJ/dhf-TZVPP, Table 8). The data indicate that, while the triple-zeta calculations somewhat increase the accuracy of the obtained data, all three methods expectedly exhibit systematic shifts which lie within an acceptable range. Therefore, calculation of the NMR shifts can be employed as a tool to narrow down the possible candidates for **1b''** and guide the further mechanistic investigations, however, it needs to be complemented by energetic considerations etc. to obtain a reliable conclusion (*vide supra*). Importantly, since especially the relative differences of the chemical shifts within each group of signals (C**H**, PymS-**H** and C$\mathbf{H}_3$) are consistent between the methods, calculation of the NMR shifts at the RI-PBE-D3BJ/dhf-SVP level already gives an accurate enough picture for the purpose at hand.

Table 8 NMR shifts of complex **1** as obtained at the various levels of theory in comparison to the experimentally determined values.

**Level of theory**	**CH**	**CH**	*Δδ (CH)*	PymS-**H**	PymS-**H**	*Δδ (PymS-H)*	CH$_3$	CH$_3$	*Δδ (CH$_3$)*
experiment	12.65	14.00	*1.35*	6.97	7.35	*0.38*	1.39	2.13	*0.74*
RI-PBE-D3BJ/dhf-SVP	10.64	11.32	*0.68*	6.16	6.62	*0.46*	0.65	1.51	*0.86*
RI-PBE-D3BJ/dhf-TZVPP	10.86	11.70	*0.84*	6.61	7.03	*0.42*	0.94	1.85	*0.91*
RIJK-B3LYP-D3BJ/dhf-TZVPP	10.91	11.68	*0.77*	6.66	7.05	*0.39*	1.02	1.79	*0.77*

References

(1) University of Karlsruhe and Forschungszentrum Karlsruhe GmbH. *TURBOMOLE*; University of Karlsruhe and Forschungszentrum Karlsruhe GmbH.

(2) Arnim, M. von; Ahlrichs, R. Performance of parallel TURBOMOLE for density functional calculations. *J. Comput. Chem.* **1998**, *19*, 1746–1757.

(3) Treutler, O.; Ahlrichs, R. Efficient molecular numerical integration schemes. *J. Chem. Phys.* **1995**, *102*, 346–354.

(4) Perdew, J. P.; Burke, K.; Ernzerhof, M. Generalized Gradient Approximation Made Simple. *Phys. Rev. Lett.* **1996**, *77*, 3865–3868.

(5) Grimme, S.; Anthony, J.; Ehrlich, S.; Krieg, H. A consistent and accurate ab initio parametrization of density functional dispersion correction (DFT-D) for the 94 elements H-Pu. *J. Chem. Phys.* **2010**, *132*, 154104.

(6) Grimme, S.; Ehrlich, S.; Goerigk, L. Effect of the damping function in dispersion corrected density functional theory. *J. Comput. Chem.* **2011**, *32*, 1456–1465.

(7) Weigend, F.; Baldes, A. Segmented contracted basis sets for one- and two-component Dirac-Fock effective core potentials. *J. Chem. Phys.* **2010**, *133*, 174102.

(8) Figgen, D.; Peterson, K. A.; Dolg, M.; Stoll, H. Energy-consistent pseudopotentials and correlation consistent basis sets for the 5d elements Hf-Pt. *J. Chem. Phys.* **2009**, *130*, 164108.

(9) Eichkorn, K.; Treutler, O.; Öhm, H.; Häser, M.; Ahlrichs, R. Auxiliary basis sets to approximate Coulomb potentials - ERRATUM. *Chem. Phys. Lett.* **1995**, *242*, 652–660.

(10) Eichkorn, K.; Treutler, O.; Öhm, H.; Häser, M.; Ahlrichs, R. Auxiliary basis sets to approximate Coulomb potentials. *Chem. Phys. Lett.* **1995**, *240*, 283–290.

(11) Eichkorn, K.; Weigend, F.; Treutler, O.; Ahlrichs, R. Auxiliary basis sets for main row atoms and transition metals and their use to approximate Coulomb potentials. *Theor Chem Acta* **1997**, *97*, 119–124.

(12) Weigend, F. Accurate Coulomb-fitting basis sets for H to Rn. *PCCP* **2006**, *8*, 1057–1065.

(13) Plessow, P. Reaction Path Optimization without NEB Springs or Interpolation Algorithms. *J. Chem. Theory Comput.* **2013**, *9*, 1305–1310.

(14) Kesharwani, M. K.; Brauer, B.; Martin, J. M. L. Frequency and zero-point vibrational energy scale factors for double-hybrid density functionals (and other selected methods): can anharmonic force fields be avoided? *J. Phys. Chem. A* **2015**, *119*, 1701–1714.

(15) Klamt, A.; Schüürmann, G. COSMO: a new approach to dielectric screening in solvents with explicit expressions for the screening energy and its gradient. *J. Chem. Soc., Perkin Trans. 2* **1993**, 799–805.

(16) Klamt, A.; Moya, C.; Palomar, J. A Comprehensive Comparison of the IEFPCM and SS(V)PE Continuum Solvation Methods with the COSMO Approach. *J. Chem. Theory Comput.* **2015**, *11*, 4220–4225.

(17) Becke, A. D. Density-functional exchange-energy approximation with correct asymptotic behavior. *Phys. Rev. A* **1988**, *38*, 3098–3100.

(18) Becke, A. D. Density-functional thermochemistry. III. The role of exact exchange. *J. Chem. Phys.* **1993**, *98*, 5648–5652.

(19) Lee, C.; Yang, W.; Parr, R. G. Development of the Colle-Salvetti correlation-energy formula into a functional of the electron density. *Phys. Rev. B* **1988**, *37*, 785–789.

(20) Weigend, F.; Ahlrichs, R. Balanced basis sets of split valence, triple zeta valence and quadruple zeta valence quality for H to Rn: Design and assessment of accuracy. *PCCP* **2005**, *7*, 3297–3305.

(21) Weigend, F. A fully direct RI-HF algorithm: Implementation, optimised auxiliary basis sets, demonstration of accuracy and efficiency. *PCCP* **2002**, *4*, 4285–4291.

(22) Reed, A. E.; Weinstock, R. B.; Weinhold, F. Natural population analysis. *J. Chem. Phys.* **1985**, *83*, 735–746.
(23) Kollwitz, M.; Gauss, J. A direct implementation of the GIAO-MBPT(2) method for calculating NMR chemical shifts. Application to the naphthalenium and anthracenium ions. *Chem. Phys. Lett.* **1996**, *260*, 639–646.
(24) Frohnapfel, D. S.; White, P. S.; Templeton, J. L. Insertion Products from Photolysis of Tp'$(CO)_3$WH and Alkynes. *Organometallics* **2000**, *19*, 1497–1506.
(25) Bondi, R.; Ćorović, M. Z.; Buchsteiner, M.; Vidovič, C.; Belaj, F.; Mösch-Zanetti, N. C. The Effect of Pyridine-2-thiolate Ligands on the Reactivity of Tungsten Complexes toward Oxidation and Acetylene Insertion. *Organometallics* **2021**, *40*, 3591–3598.
